# Enhanced N-directed electrophilic C–H borylation generates BN–[5]- and [6]helicenes with improved photophysical properties†

**DOI:** 10.1039/d1sc06513k

**Published:** 2022-01-04

**Authors:** Kang Yuan, Daniel Volland, Sven Kirschner, Marina Uzelac, Gary S. Nichol, Agnieszka Nowak-Król, Michael J. Ingleson

**Affiliations:** EaStCHEM School of Chemistry, The University of Edinburgh David Brewster Road Edinburgh EH9 3FJ UK michael.ingleson@edinburgh.ac.uk; Institut für Anorganische Chemie, Institute for Sustainable Chemistry and Catalysis with Boron Universität Würzburg Am Hubland 97074 Würzburg Germany agnieszka.nowak-krol@uni-wuerzburg.de; Institut für Organische Chemie & Center for Nanosystems Chemistry, Universität Würzburg Am Hubland 97074 Würzburg Germany

## Abstract

Helicenes are chiral polycyclic aromatic hydrocarbons (PAHs) of significant interest, *e.g.* in supramolecular chemistry, materials science and asymmetric catalysis. Herein an enhanced N-directed electrophilic C–H borylation methodology has been developed that provides access to azaborine containing helicenes (BN–helicenes). This borylation process proceeds *via* protonation of an aminoborane with bistriflimidic acid. DFT calculations reveal the borenium cation formed by protonation to be more electrophilic than the product derived from aminoborane activation with BBr_3_. The synthesised helicenes include BN-analogues of archetypal all carbon [5]- and [6]helicenes. The replacement of a CC with a BN unit (that has a longer bond) on the outer helix increases the strain in the BN congeners and the racemization half-life for a BN–[5]helicene relative to the all carbon [5]helicene. BN incorporation also increases the fluorescence efficiency of the helicenes, a direct effect of BN incorporation altering the distribution of the key frontier orbitals across the helical backbone relative to carbo-helicenes.

## Abstract 2

An enhanced N-directed electrophilic C–H borylation methodology has been developed that provides access to azaborine containing helicenes.

## Graphical abstract

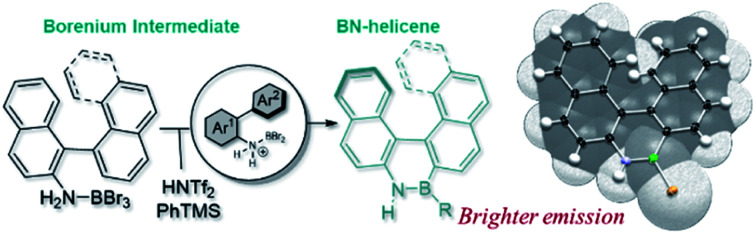

## Introduction

**Fig. 1:**
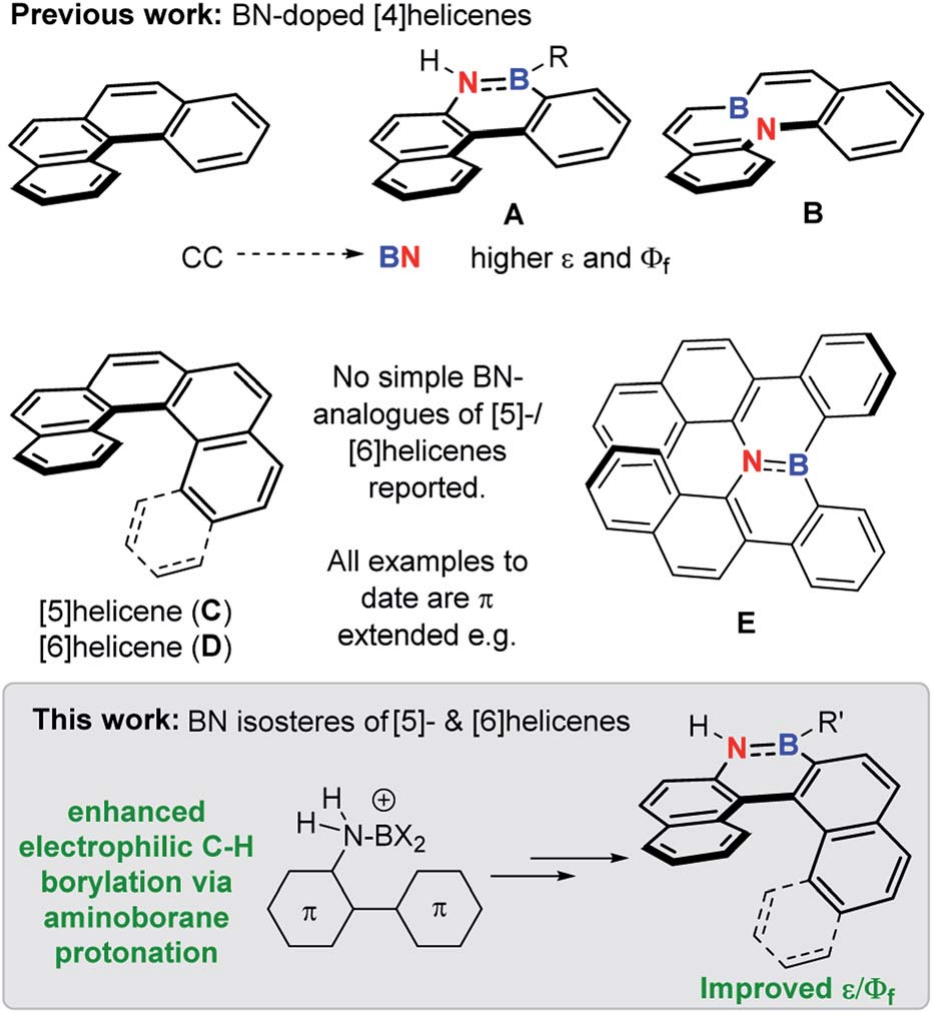
Top, previous work on BN-isosteres of [4]helicenes. Middle, [5]- and [6]helicene and a benzannulated BN analogue thereof. Bottom, this work.

The replacement of CC for isoelectronic BN units generates superficially similar molecules, but the BN congeners often have distinct properties.^1^ Due to this phenomenon BN incorporation into organic materials has received significant attention,^2^ with it now an established approach to modify physical and optoelectronic properties.^3^ This approach has been applied to helicenes, with BN incorporation within the helical backbone of [4]helicenes (*e.g.*A and B, Fig. 1) found to improve the fluorescence efficiency relative to the all carbon [4]helicene.^4^ However, the incorporation of BN units into the helical backbone of the higher [*n*]helicenes (*n* ≥ [5], necessary for configurational stability) is underdeveloped, with no simple BN analogues of the higher helicenes, [5]- and [6]helicene (C and D), reported to date to our knowledge (all examples published to date are π extended BN–helicenes, *e.g.*E).^5^ While four coordinate at boron BN units^6^ and other boron units^7^ have been incorporated into the backbone of higher helicenes, these do not contain helical cores isoelectronic to helicenes C and D.

The scarcity of BN-isosteres of the higher helicenes is presumably due to the synthetic challenge of incorporating BN units into these strained PAHs. Methods to form BN-PAHs predominantly build on Dewar's seminal work^8^ in which –N(R)H substituted PAHs are functionalised *via* electrophilic C–H borylation using haloboranes, often in the presence of an additional Lewis acid (*e.g.* AlCl_3_) and/or a base.^9^ Notable work extending Dewar's approach to access BN-containing higher helicenes comes from Hatakeyama and co-workers who reported benzo-fused aza (and oxa) bora-helicenes, *e.g.*E, and multi-helicenes.^5^ However, the synthesis of the BN helicenes reported to date requires forcing conditions, for example, A is formed by heating the amine precursor with excess BBr_3_ at 220 °C.^4*b*^ Furthermore, these reactions proceed *via* ill-defined boron electrophiles, and the optimal borylation conditions are often highly substrate/Lewis acid and base dependent.^10^ Thus generating an N-directed electrophilic C–H borylation method that proceeds under milder conditions for a range of challenging substrates would facilitate access to novel BN materials.

Herein we report a novel N-directed electrophilic C–H borylation method proceeding by protonation of the amino-borane to form borenium (three coordinate at boron) cations. This enables access to challenging to synthesise azaborine containing PAHs even at room temperature, including [5]- and [6]helicenes containing BN units in the helical backbone. These BN–helicenes have improved photophysical properties relative to the carbo[5]- and [6]helicenes, further highlighting the beneficial effects afforded by BN incorporation into helicenes.

## Results and discussion

### Synthetic studies

**Scheme 1:**
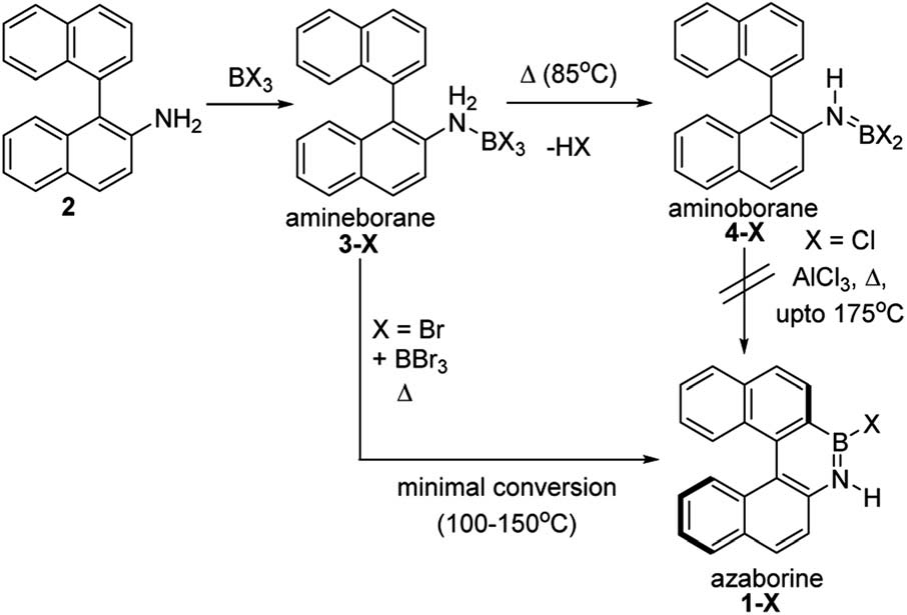
Attempts to form 1-X using previously reported borylation methods.

The BN–[5]helicenes 1-X (X = Br or Cl, Scheme 1), were targeted from amine 2. Amine 2 forms amineborane adducts 3-X on addition of BX_3_, with heating leading to dehydrohalogenation to form aminoboranes 4-X. The use of Dewar's conditions,^8^ specifically conversion of 3-Cl to aminoborane 4-Cl, addition of AlCl_3_ and then heating (up to 175 °C), led to a complex reaction mixture with no 1-Cl observed (by NMR spectroscopy, Fig. S3 and S4†). Many azaborines are made by addition of excess BBr_3_ to the amine and heating,^4*b*,11^ therefore excess BBr_3_ was added to 3-Br. However, heating under a range of conditions (at temperatures ranging from 85 °C to 150 °C) led to minimal conversion to the azaborine 1-Br even after prolonged reaction times (based on diagnostic resonances in the ^1^H and ^11^B NMR spectra, Fig. S5–S9†). Furthermore, heating 4-X/BBr_3_ at 150 °C for prolonged periods actually led to the formation of significant amounts of unidentified by-products and insoluble material. Therefore, these established N-directed borylation routes do not afford 1-X in any significant yield (all reactions afforded <30% 1-X by *in situ* NMR spectroscopy).

**Fig. 2:**
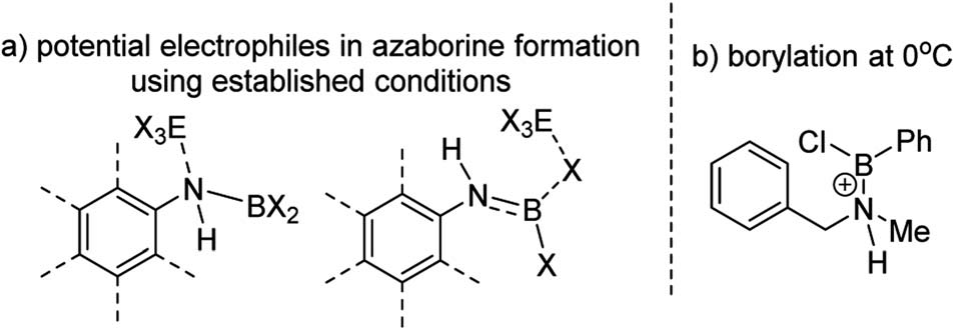
(a) Potential electrophiles in azaborine formation *via* established C–H borylation routes. (b) C–H borylation at 0 °C by a protonated aminoborane.

The challenge in accessing 1-X is presumably due to the increased strain in the BN–[5]helicene relative to other azaborines. We hypothesised that a more reactive borylating electrophile would enable higher yielding access to 1-X. The exact electrophiles in established high temperature *N*-directed electrophilic C–H borylation reactions currently are ill-defined and may originate from the Lewis acid EX_3_ (EX_3_ = AlCl_3_/BBr_3_) interacting with a B–X or N moiety of the aminoborane (Fig. 2a).^9*b*^ Notably, reactions that proceed by accessing borenium cations^12^ by protonation of aminoboranes can lead to facile N-directed C–H borylation, even at 0 °C (Fig. 2b).^13^ This is consistent with N protonation of oxazaborolidines with HNTf_2_ forming more reactive (as catalysts in Diels–Alder reactions) Lewis acids than congeners derived from binding of BBr_3_ or AlCl_3_ at N.^14^ Thus conditions that form [ArylNH_2_BX_2_]^+^, [5-X]^+^, derived from 3-X or 4-X, partnered with a suitable weakly coordinating anion were sought to generate enhanced N-directed C–H borylation.

**Scheme 2:**
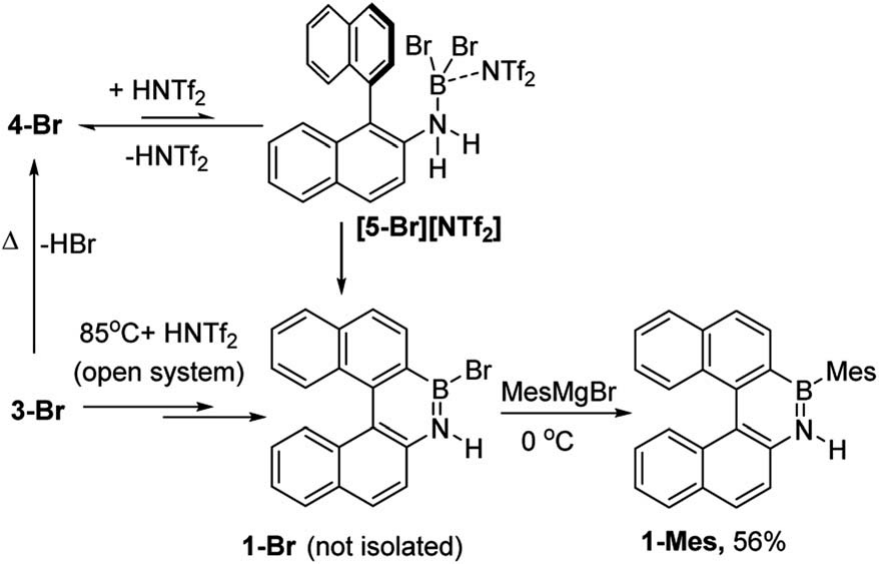
Formation of 1-Br using HNTf_2_ and heating in an open system (termed route i) and subsequent transformation into 1-Mes.

The simplest route to access [5-X]^+^ is by protonation of an aminoborane by a sufficiently strong Brønsted acid. Bis(trifluoromethanesulfonyl)imide ([NTf_2_]^−^) was selected as the counterion as it is weakly coordinating (including towards borenium cations),^15^ and the strong Brønsted acid, HNTf_2_, is commercially available. Notably, the addition of HNTf_2_ to a range of aminoboranes led to no observable protonation, indicating an extremely low basicity of these aminoboranes. However, presumably some protonation is occurring as heating 3-X/HNTf_2_ mixtures at 85 °C (a temperature at which dehydrohalogenation occurs to form the aminoborane 4-X) leads to formation of the azaborine 1-Br. The borylation proceeds in good yield starting from 3-Br when heated in an open-system, which is essential to facilitate loss of HBr and help drive the reaction to completion (see ESI Fig. S10 and S11†). As HNTf_2_ is not consumed in this reaction, its loading could be reduced (*e.g.* to 30 mol%) and after 6 h 1-Br is formed in *ca.* 90% conversion under these conditions (by *in situ* NMR spectroscopy, Fig. S11†). Installation of mesityl onto 1-Br by addition of MesMgBr at 0 °C enabled the isolation of 1-Mes (Scheme 2). 1-Mes is bench stable in solution for at least two months (by ^1^H NMR spectroscopy), and displayed a *δ*_11B_ = 39.5 comparable to related benzannulated azaborines.^16^1-Br also was converted to 1-Ph, however, 1-Ph proved unstable during work up and could not be isolated cleanly.

**Scheme 3:**
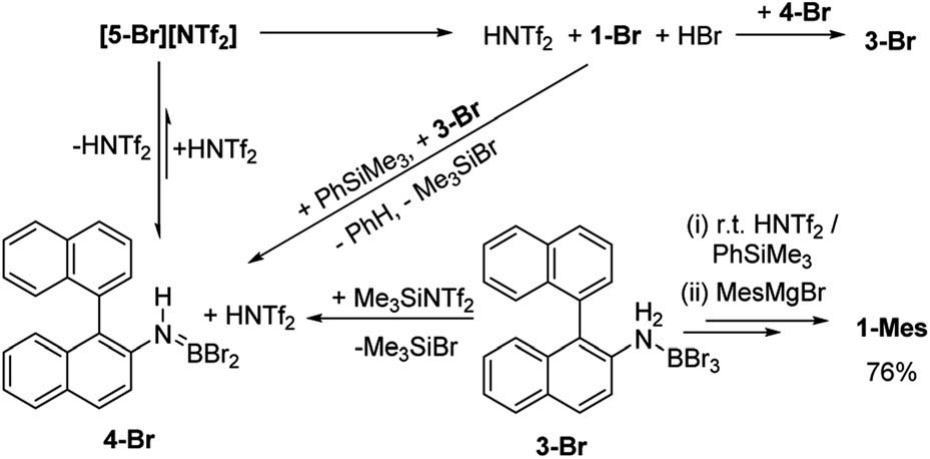
Me_3_SiNTf_2_ mediated, N-directed, C–H borylation (termed route ii).

As [5-Br][NTf_2_] is not observed we targeted its formation *via* an alternative route to support its intermediacy. Addition of Me_3_SiNTf_2_ (made by protodesilylation of PhSiMe_3_ with HNTf_2_) to 3-Br led to formation of Me_3_SiBr and 4-Br (Fig. S12 and S13†). While [5-Br][NTf_2_] could not be detected in this reaction either, the presence of these compounds supports the formation of [5-Br][NTf_2_], and indicates that it converts to HNTf_2_ and 4-Br (Scheme 3). Remarkably, this reaction mixture produces azaborine 1-Br at room temperature, with the by-product from S_E_Ar, HBr sequestered by 4-Br to reform 3-Br (under these room temperature closed vessel conditions). The reformed HNTf_2_ can be used to react with further PhSiMe_3_ to produce benzene and Me_3_SiNTf_2_, the latter reacts with more 3-Br to produce further 4-Br, Me_3_SiBr and HNTf_2_ which in turn produces more 1-Br. This cycle can be repeated until all 3-Br is converted into 1-Br, and this was achieved by sequential addition of multiple portions of PhSiMe_3_ until 1-Br is the major boron containing species (>90% conversion by *in situ* NMR spectroscopy – Fig. S12 and S13†). Ultimately upon addition of MesMgBr, 1-Mes can be isolated in 76% yield *via* this approach. Note the borylation of amineboranes (*e.g.*3-Br) cannot be performed by adding one portion of excess PhSiMe_3_ at the start of the reaction, as HNTf_2_ reacts rapidly with PhSiMe_3_, thus this combination instead produces benzene, the aminoborane (4-Br) and Me_3_SiNTf_2_. Note, the latter two combined do not react to generate a boron electrophile able to effect C–H borylation and form 1-Br.

Thus there are two related borylation methodologies that can be used: route (i) heat amineboranes + HNTf_2_ at 85 °C, and route (ii) at room temperature by combining amineboranes with Me_3_SiNTf_2_ and subsequent addition of portions of PhSiMe_3_. The key in both cases to enable high conversion to the azaborine is accounting for the sequestering of the HBr by-product from S_E_Ar by the aminoborane 4-Br (forming the amineborane 3-Br). The amineborane can be converted to the aminoborane either by heating in an open vessel (under inert atmosphere) or by adding PhSiMe_3_ portionwise. Both regenerate the key aminoborane/HNTf_2_ combination which effects C–H borylation. The importance of the latter combination has been confirmed by directly using these two species to effect C–H borylation which also proceeds at room temperature (see Fig. S12 and S13†).

**Scheme 4:**
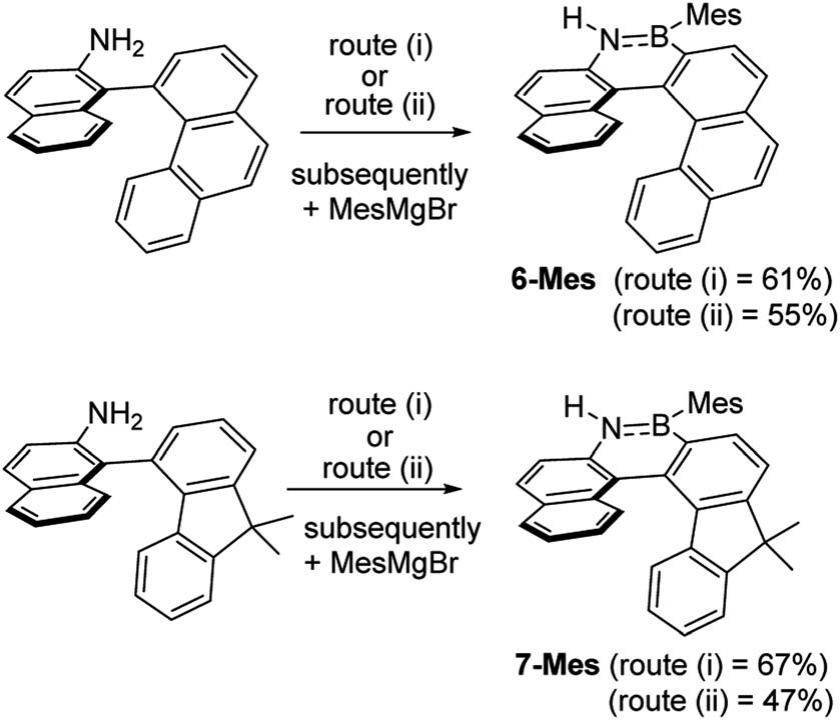
The formation of 6-Mes and 7-Mes by electrophilic C–H borylation.

**Scheme 5:**
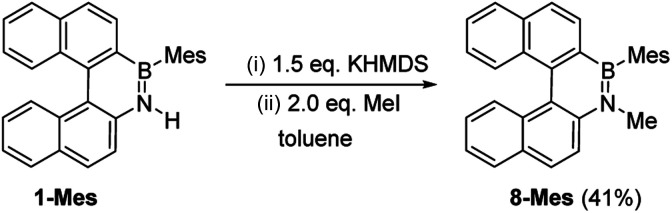
Synthesis of 8-Mes by functionalizing 1-Mes.

These borylation methodologies also could be used to form the 6-Br and 7-Br. These one-pot reactions were completed by mesityl protection using MesMgBr to afford bench stable 6-Mes and 7-Mes (Scheme 4). In contrast, attempts to make 7-Br from the respective amineborane using excess BBr_3_ under a range of conditions (including at 150 °C for 72 h), led to its formation only as a minor product (by *in situ* NMR spectroscopy – Fig. S18†). The mesityl protected helicenes were amenable to *N*-substituent transformations using standard azaborine functionalisation methodologies (Scheme 5).^1^ For example, deprotonation of 1-Mes with KHMDS and addition of MeI leads to formation of the N–Me analogue of 1-Mes, 8-Mes.

**Fig. 3:**
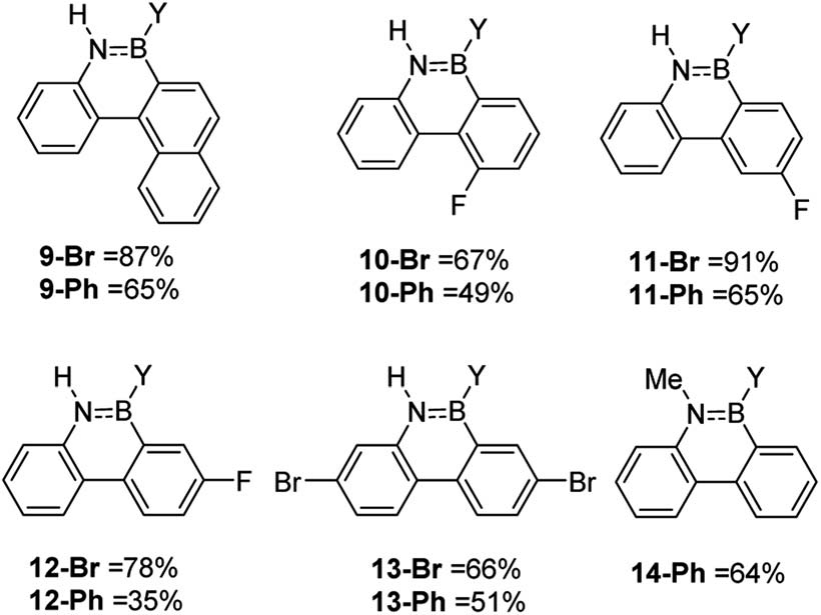
Select scoping study using challenging substrates. Y = Br or Ph.

While the formation of the BN–helicenes demonstrates the utility of these new borylation methods to access strained azaborines, its applicability to access other challenging (*e.g.* electronically deactivated towards S_E_Ar) substrates also was assessed. Conversion was assessed *in situ* and subsequently each azaborine was protected at boron and isolated as the B-Ph derivatives. In contrast to the unsuccessful borylation using Dewar's conditions (BCl_3_/AlCl_3_/Δ),^17^ azaborines 9-Y and 10-Y (Fig. 3) were readily accessible using the new methodologies. Furthermore, for 11-Br the regioselectivity was high with no borylation observed *ortho* to fluorine. This methodology also provides an efficient route to the dibrominated azaborines 13-Y, which were previously made *via* the borafluorene and an arylazide.^18^ Finally, this procedure is applicable to secondary amines, *e.g.*14, and to chloro analogues utilising BCl_3_ in place of BBr_3_ (Fig. S31–S34†). Although the chloro derivatives are formed in lower yield, presumably due to the lower electrophilicity of B–Cl *vs.* B–Br containing electrophiles.

**Scheme 6:**
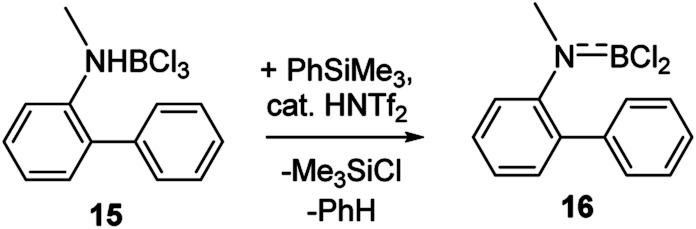
Dehydrochlorination mediated by Me_3_SiNTf_2_.

Finally, it should be noted that utilising combinations of substoichiometric HNTf_2_ and stoichiometric PhSiMe_3_ to effect amineborane dehydrohalogenation is also useful. Certain amineboranes do not undergo thermal dehydrohalogenation readily (*e.g.*15 does not at 140 °C), but using HNTf_2_/PhSiMe_3_ dehydrohalogenation proceeds to completion at room temperature to afford 16 (Scheme 6) after 7 h (effectively quantitatively by NMR spectroscopy – Fig. S35–S37†).

### Calculations on borylation using HNTf_2_ and BBr_3_ as activators

**Scheme 7:**
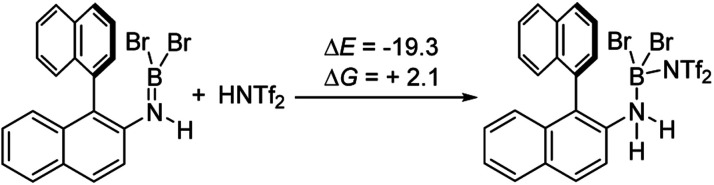
Energy change on protonation with HNTf_2_ (kcal mol^−1^).

It is notable that the HNTf_2_ mediated borylation methodology gives high yielding formation of BN–[5]/[6]-helicenes from the respective aminoboranes, even at room temperature. In contrast the use of only BBr_3_ resulted in low yielding (at best) formation of BN–[5]/[6]helicenes under a range of conditions (including forcing conditions). DFT calculations were performed at the M06-2X/6-311+G(d,p) level with a polarisable continuum model (PCM) DCM (this level has provided accurate thermodynamic data in previous calculations on directed electrophilic C–H borylation)^19^ to provide insight into this disparity. Note, herein we discuss just the [5]helicene system, but analogous results were found with the parent 2-amino-biphenyl system (see Fig. S87 and Scheme S1†), suggesting the outcomes are general for this type of aminoborane. Both borylation routes involving [NTf_2_]^−^ lead to an aminoborane and HNTf_2_ being present in solution, with the hypothesis being that protonation of the aminoborane with HNTf_2_ proceeds, but only to a small extent (thus is not observed by NMR spectroscopy). Consistent with this, the reaction of an aminoborane with HNTf_2_ was found to be slightly endergonic (Scheme 7), thus borenium equivalents will be accessible in solution from aminoboranes and HNTf_2_.

**Scheme 8:**
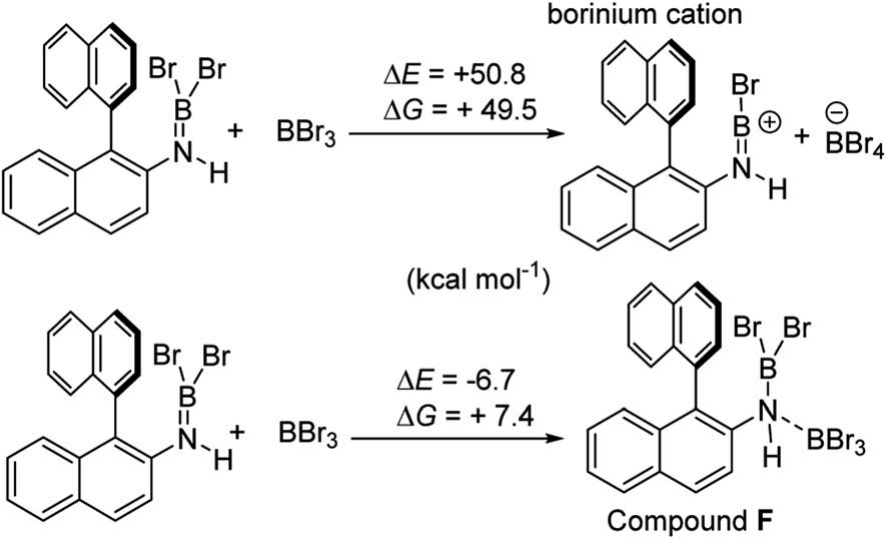
Energy change on BBr_3_ activation of aminoboranes.

Aryl N-directed C–H borylation with BBr_3_ generally only proceeds at high temperatures (as observed with the BN–helicenes herein). At these temperatures dehydrohalogenation of the amineborane to the aminoborane occurs. Therefore we attribute the difference in reaction outcomes when using HNTf_2_ and when using BBr_3_ to how these species activate the aminoborane. BBr_3_ could interact with the aminoborane by binding at N or by interacting with a Br moiety. Interaction of BBr_3_ with a Br in the aminoborane leads to borinium (two coordinate at boron) cation formation (Scheme 8, top), with no B–(μ-Br)–BBr_3_ species located as a minima in our hands. Notably, the formation of the borinium cation from the aminoborane and BBr_3_ is not energetically accessible, precluding its intermediacy during borylation using just BBr_3_.

**Fig. 4:**
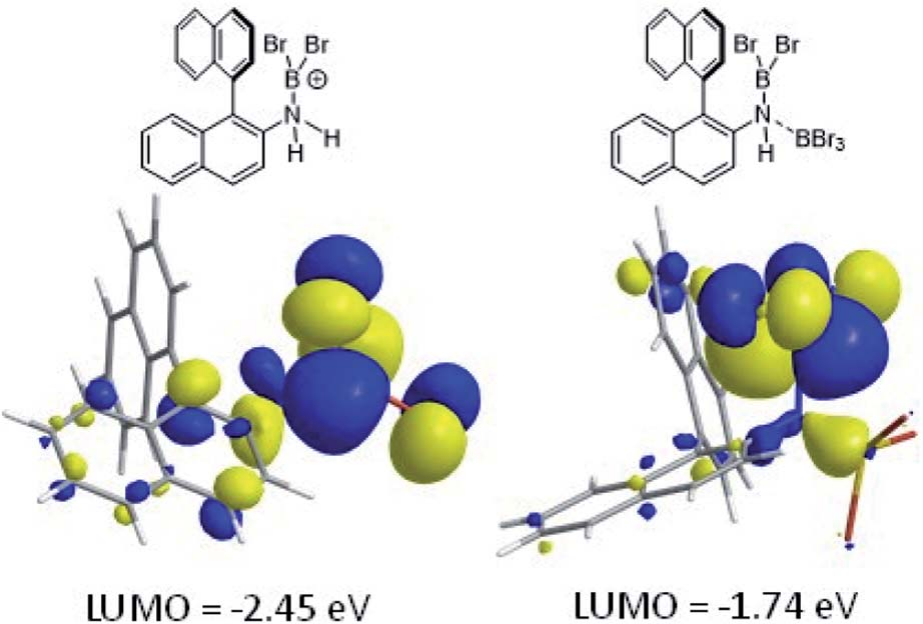
LUMO of, left, borenium cation G formed on protonation of an aminoborane, right, borenium equivalent F formed on addition of BBr_3_.

The coordination of BBr_3_ at N to form Lewis acid F is significantly less endergonic than borinium cation formation (Scheme 8, bottom), thus it is more feasible. However, while compound F is a functional borenium equivalent^12*b*^ it is significantly less electrophilic than bona-fide boreniums, *e.g.* [ArylNH_2_BBr_2_]^+^G, based on their relative LUMO energies (Fig. 4). Both LUMOs have significant character on the boron centre involved in forming the C–B bond during borylation, but for the functional borenium equivalent F, the LUMO is much (*ca.* 0.7 eV) higher in energy than the bona-fide borenium cation G. This is consistent with previous studies where protonolysis at N generates stronger Lewis acids relative to analogues formed by coordination of Lewis acids at N in oxazaborolidines.^14^ Therefore the enhanced borylation reactivity of the HNTf_2_ mediated system can be attributed to (a) an energetically viable protonation of the aminoborane, and (b) the much greater electrophilicity of the borenium cation [RNH_2_BBr_2_]^+^ (*e.g.*G) compared to the BBr_3_ adduct of the aminoborane (*e.g.*F).

**Scheme 9:**
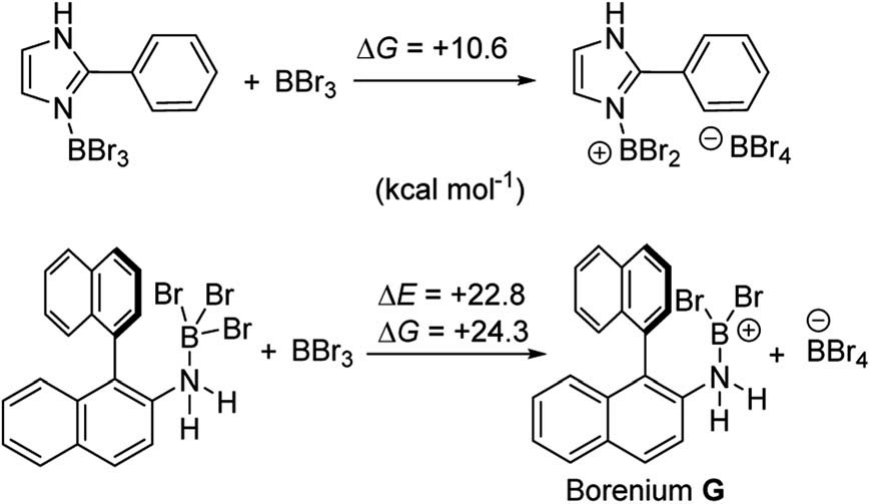
Energy change during generation of borenium cations from an imidazole-BBr_3_ adduct (top) and an amineborane (bottom) using BBr_3_.

It is also noteworthy to highlight the disparity between facile BBr_3_ mediated directed C–H borylation reactions (*e.g.* using N-heterocycles or pivaloyl as directing groups)^19,20^*versus* the much more challenging directed borylation to make 1-Br using just BBr_3_. In other systems, *e.g.* imidazole or pivaloyl directed borylation, formation of the borenium cation *via* halide abstraction from the Lewis base→BBr_3_ adduct using BBr_3_ is calculated to be energetically uphill by between +10–+13 kcal mol^−1^ (Scheme 9, top).^17,18^ Thus these borenium cations are readily accessible at room temperature. In contrast, bromide abstraction from 3-Br using BBr_3_ to form G is significantly more endergonic, at +24 kcal mol^−1^. This disparity is presumably due to the absence of π donation from the directing group (*e.g.* pivaloyl or an N-heterocycle) that will stabilise the borocation as previously discussed.^21^ In compound G the formally empty boron p_*z*_ orbital only is stabilised by π donation from two bromides. The relatively high energy of the borenium cation presumably contributes to the failure to form 1-Br from 2 using just excess BBr_3_ at room temperature, as the barrier to C–H borylation mediated by G will be >24.3 kcal mol^−1^.

### Discussion of the structures of BN–helicenes

**Fig. 5:**
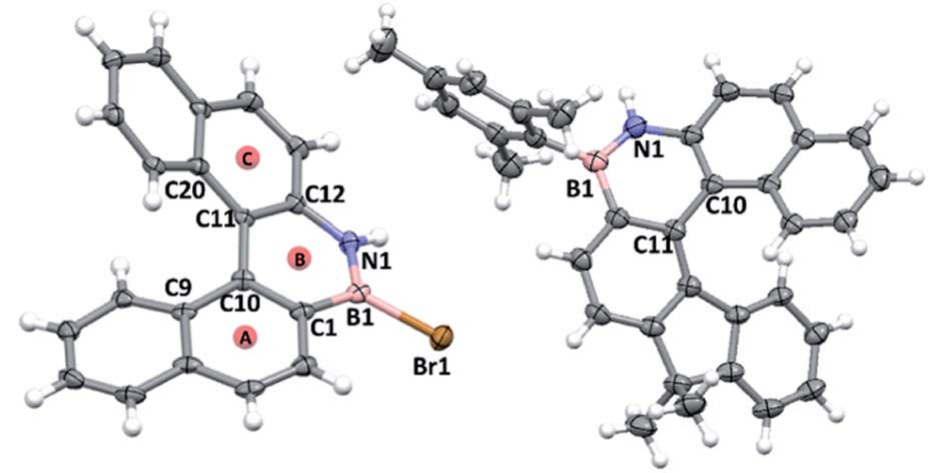
Left, solid state structure of 1-Br and right, solid state structure of 7-Mes, ellipsoids at 50%, red circles = calculated centroids.

1-Br crystallised as a racemate with a single helicene molecule in the asymmetric unit. Metrics within the azaborine unit are unremarkable and are comparable to B-aryl substituted azaborines (no B–Br azaborine structure has been reported to date to our knowledge).^22^ Comparison of the structure of 1-Br to that of the all carbon [5]helicene C is noteworthy.^23^ In 1-Br the C9–C10–C11–C20 and C1–C10–C11–C12 torsion angles (Fig. 5) are greater (34.6(5)° and 24.1(5)°, respectively) than the analogous angles in C (32.3(2)° and 20.2(2)°). Furthermore, the angles between the centroids A-B-C in 1-Br is 122.95°, while the comparable angle in C is 124.22°. In addition, the dihedral angle between the mean planes of the two terminal rings of 1-Br (58.88°) are significantly larger than that of the all carbon [5]-helicene C (47.30°). Note, similar trends are observed when comparing the metrics of the optimized geometries of 1-Mes and it carbo[5]helicene analogue (at the same level of theory), thus they are not due to packing effects. The origin of these disparities is attributed to the difference in key bond distances between the inner helix and the outer helix; for 1-Br C10–C11 (inner) = 1.472(5) Å, B1–N1 (outer) = 1.393(6) Å (*δ* = 0.079 Å) while the respective distances in C are: (inner) = 1.451(2) Å and (outer) = 1.348(2) Å (*δ* = 0.103 Å). The relatively long BN bond located at the centre of the outer helix in 1-Mes is a direct result of the weaker π bond in BN units *versus* that in CC, and this generates a slightly larger in-plane turn for the BN helicene relative to the carbon-analogue.

**Fig. 6:**
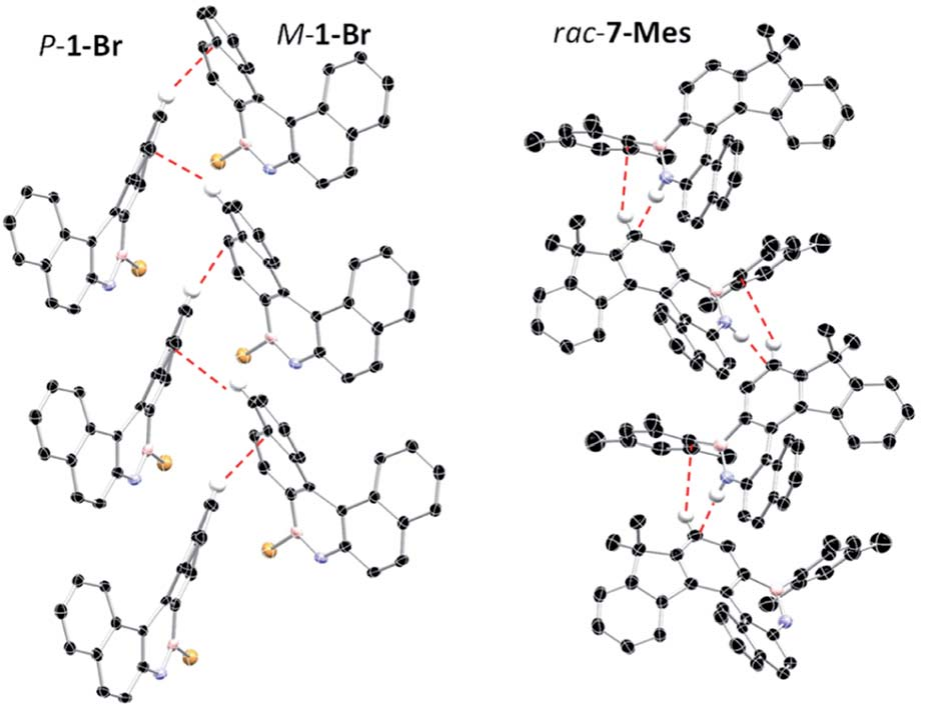
Packing diagrams of 1-Br (left) and 7-Mes (right). Dashed lines represent intermolecular interactions.

The solid state structures of 6-Mes and 7-Mes also were obtained, although the former contained significant disorder thus is not discussed (Fig. 5 and S41†). It should be noted that the calculated structures of 6-Mes and its carbo[6]helicene analogue have comparable trends to that discussed above for the BN and CC [5]helicene isosteres. The structure of 7-Mes has comparable key metrics to that of 1-Br, including similar BN and C10–C11 distances (for 7-Mes 1.409(3) Å and 1.465(2) Å, respectively). The sum of the torsion angles of the inner rim for 7-Mes is 71.30°, which is smaller than the all carbon 6-helicene D (88.08°)^24^ but is comparable to that of an aza-6-helicene that also contains a five-membered pyrrole unit (67.71°).^25^ The packing structures of *rac*-1-Br and *rac*-7-Mes are shown in Fig. 6. The extended structure of 1-Br contains adjacent columns of left handed and right handed helices, with the closest intermolecular distance between non-hydrogen atoms within a column being 3.248 Å. The adjacent *P*- and *M*-isomers are connected through intermolecular C–H⋯π interactions with a distance of 2.855 Å. In contrast to 1-Br, *rac*-7-Mes packed in an alternating fashion with *P*- and *M*-isomers interacting with each other through C–H⋯π and N–H⋯π interactions.

**Scheme 10:**
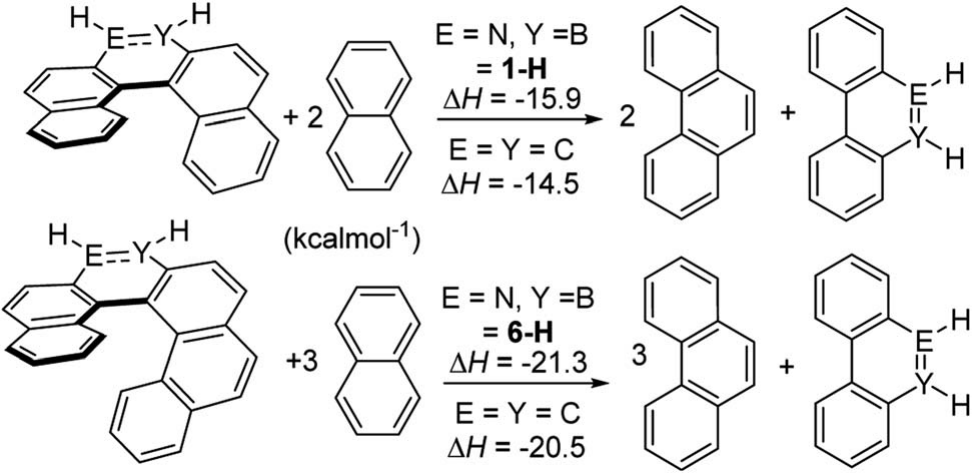
Calculated strain energy, values for carbon isosteres are from ref. 26.

To probe for consequences of the structural differences between the BN and CC helicene analogues discussed above the strain energy was calculated using a previously reported methodology.^26^ This was performed using the model complexes 1-H and 6-H to enable direct comparison to previously studied carbon analogues (with calculations performed at an identical level to previous work);^26^ note it has been shown previously that changing substituents on the outer helix (*e.g.* from 1-H to 1-Mes) has minimal effect on key steric modified parameters (*e.g.* strain energy/racemization barriers).^27^ These calculations (Scheme 10) showed that the BN–[5]- and [6]helicenes have approximately 1 kcal mol^−1^ greater strain energy than the carbon analogues.^26^ This is consistent with the greater torsion angles observed in 1-Br (large torsion angles relative to all carbon analogues also are observed along the inner helix for the calculated BN–helicenes) due to the smaller difference between inner and outer helix bond distances.

Another consequence of the different metrics/greater in plane turn in 1-Br relative to [5]helicene C is a greater barrier to racemization. The BN–[5]helicenes 1-H and 1-Mes were calculated to have higher barriers to *P*–*M* interconversion by *ca.* 1.5 kcal mol^−1^ than C and a 7-mesityl derivative (Fig. S92 and S93†). [5]Helicene has been determined previously to have a barrier to racemization of *ca.* 24 kcal mol^−1^.^26,27^ It is also important to note that 1-H and 1-Mes have effectively identical calculated barriers to racemization (*δ*Δ*E* = 0.2 kcal mol^−1^) confirming that there is minimal effect when changing a substituent on the outer helix. To confirm the *in silico* results, a single enantiomer of 1-Mes was isolated using enantioselective HPLC. This single enantiomer was kept in solution at 20 °C and monitored periodically by enantioselective HPLC (Fig S40†). This confirmed a slower racemization, for example a *ca.* 70 : 30 e. r. mixture was observed after 310 h and a racemate only formed after *ca.* one month. This is slower by approximately an order of magnitude than the rate of racemization for the [5]helicene C (which fully racemises within several days).^27,28^

### Optoelectronic properties

The photophysical properties of 1-Mes, 6-Mes and 7-Mes were investigated by UV-vis absorption and fluorescence spectroscopy.

**Fig. 7:**
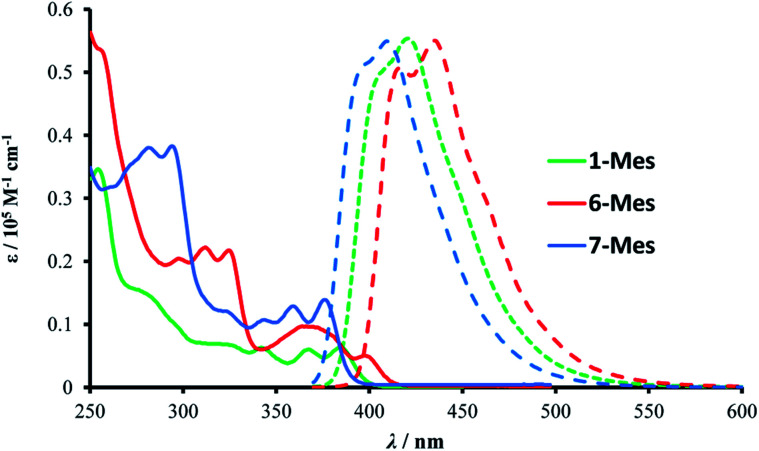
Absorption (solid line) and emission (dashed line) spectra of the three azaborines in dichloromethane (10^−5^ M).

As shown in Fig. 7 and Table 1, compound 1-Mes and 6-Mes displayed quite strong shoulder absorption bands centred at around 390 nm (387 nm for 1-Mes and 397 nm for 6-Mes), which is in stark contrast to the pristine carbohelicenes that exhibit very weak absorption bands at the longest wavelength (*e.g.* the molar extinction coefficient *ε* of [5]helicene C at 393 nm is 200 M^−1^ cm^−1^ and the *ε* of [6]helicene D at *ca.* 415 nm is *ca.* 300 M^−1^ cm^−1^).^29,30^ Similarly, compound 7-Mes also exhibited strong absorptions for the lowest energy transition centered at 374 nm. Due to the presence of a non-aromatic cyclopentadiene ring, the spectrum is hypsochromically shifted *vs.* those of 1-Mes and 6-Mes. Furthermore, unlike the all-carbon [5]- and [6]helicenes C and D, which are reported to have a *Φ*_PL_ of only 4%,^29^ all three azaborine helicenes studied herein exhibit stronger fluorescence with *Φ*_PL_ being at least five times higher than these carbohelicenes. Among the three azaborine compounds 7-Mes shows the highest luminescence quantum yield, which may be attributed to the fluorene unit as the latter has also been reported to improve the *Φ*_PL_ of carbohelicenes.^31^ The relatively small Stokes shifts (1090, 1320, and 1420 cm^−1^ for 1-Mes, 6-Mes, and 7-Mes, respectively) and vibrational structures of the emission spectra indicate their considerable rigidity.

**Table 1:** Optoelectronic properties of the three azaborine helicenes

	*λ*_abs_/nm [*ε*/10^5^ M^−1^ cm^−1^]	*λ*_PL_/nm	*Φ*_PL_ b	*E* ^Red^_1/2_ V^c^
1-Mes^a^	255 [0.345], 279 [0.152], 329 [0.063], 344 [0.059], 370[0.056], 387 [0.055]	404, 422	0.30	−2.788
6-Mes^a^	257 [0.531], 299 [0.203], 312 [0.222], 326 [0.213], 370 [0.096], 397 [0.053]	419, 436	0.21	−2.783
7-Mes^a^	283 [0.379], 295 [0.381], 325 [0.118], 345 [0.106], 361 [0.125], 374 [0.134]	395, 411	0.42	−2.923

a Absorption and emission spectra are of 10^−5^ M DCM solutions.

b Calculated using 9,10-diphenylanthracene as a standard.

c Reduction potentials were calibrated with ferrocene as an internal standard and referenced *vs.* Fc^+^/Fc.

In order to gain a deeper understanding of the impact of BN/CC replacement on the electronic properties of these helicenes, DFT and TD-DFT calculations were performed. Firstly, NICS(0) calculations revealed a much lower aromaticity of the BN-containing heterocycle in 1-Mes and 6-Mes relative to the comparable ring in the carbon analogues (see Fig. S94†), this is consistent with previous work on BN containing PAHs (including helicenes).^9,10^ The HOMO and LUMO energy for the BN helicenes were calculated and were comparable in trends to the experimental values. While comparison to the carbon isosteres revealed similar LUMO energies there is an increase in the energy of the HOMO (by *ca.* 0.2 eV) for 1-Mes and 6-Mes relative to H and I (see Fig. S95†).

**Fig. 8:**
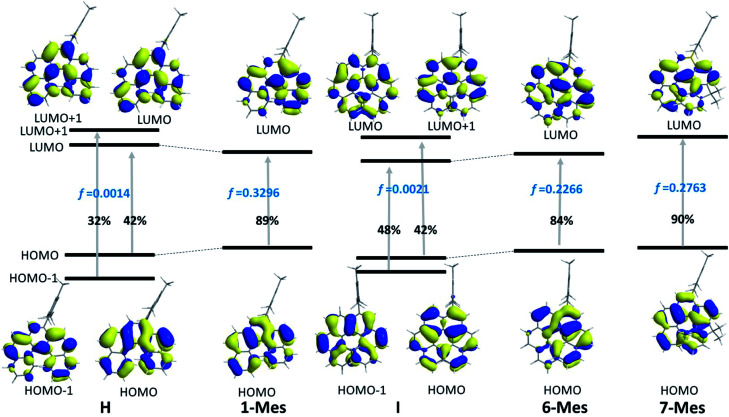
Molecular orbital diagrams (*iso* = 0.03) and the major compositions of S_0_ → S_1_ transitions and corresponding oscillator strength *f*.

The TD-DFT calculations taking 1-Mes and H (H = a mesityl functionalised all-carbon [5]helicene) as examples, revealed the lowest energy transition of 1-Mes is dominated by the HOMO→LUMO transition with the oscillator strength (*f*) being 0.3296 (Fig. 8). Notably, the incorporation of the BN unit alters the symmetry of the frontier orbitals (relative to the CC analogue H) with a significantly different orbital distribution throughout the helical backbone (note there is no contribution to the frontier orbitals from the mesityl group for any of these helicenes). Analogous to previous reports on all carbon–[5]helicenes, for H, the S_0_ to S_1_ transitions contains two major contributions: (i) HOMO to LUMO and (ii) HOMO−1 to LUMO+1 and has a low calculated oscillator strength for this transition (0.0014) in contrast to 1-Mes. Inspection of the frontier molecular orbitals of 1-Mes and H provides insights into this disparity: for H the S_0_–S_1_ transition is symmetry forbidden as previously discussed for carbo[5]helicenes.^30,32^ The replacement of a CC unit with a BN unit breaks the C_2_ symmetric distribution of the HOMO and LUMO across the helical backbone, and results in unsymmetric key frontier orbitals. Consequently, the S_0_–S_1_ transition for 1-Mes has a higher oscillator strength, thus stronger absorptions for the lowest energy transition and improved *Φ*_PL_ are observed for 1-Mes relative to C. TD-DFT calculations on 6-Mes and the all carbon analogue I revealed a similar trend to 1-Mes/H consistent with the reduction in symmetry and the unsymmetrical orbital distribution across the helical backbone for the HOMO and LUMO in 6-Mes. Compound 7-Mes also was predicted to have a large oscillator strength for the S_0_→S_1_ transition, which is in agreement with the experimental data. In order to examine for any potential impact of the mesityl group on the electronic transitions, DFT/TD-DFT calculations were performed on 1-H and 6-H in which the Mes groups are replaced with hydrogens (Fig. S88†). The calculated results for 1-H and 6-H are very similar to that of 1-Mes and 6-Mes, respectively, further indicating that the mesityl group has a minimal role in determining the key photophysical properties of these BN–helicenes.

Attempts were made to separate the enantiomers of 6-Mes and 7-Mes using chiral HPLC. In our hands, while 6-Mes could be resolved (Fig. S38†), 7-Mes could not, despite utilising a wide range of solvent and column conditions. As expected, 6-Mes reveals significantly higher tolerance to racemisation than its shorter homologue 1-Mes. Thus, the optically pure BN–[6]helicene can be handled without any special precautions as no deterioration of enantiomeric excess could be observed either at room temperature or somewhat elevated temperatures. To get more insight into its configurational stability, the sample of (*M*)-6-Mes in 1,2-dichlorobenzene was heated at 130–150 °C for 2–64 h followed by HPLC analysis of enantiomeric ratios. A barely visible shoulder started to arise after heating the sample at 150 °C for 16 h. To produce *ca.* 10% of the second enantiomer, the sample had to be kept at this temperature for over 2.5 days. The *P*–*M* interconversion process at 140 °C was significantly slower. To compare the stability of 6-Mes and carbo[6]helicene, which possesses the inversion barrier of *ca.* 36 kcal mol^−1^, the sample composition of carbo[6]helicene under given conditions was calculated based on the available thermodynamic parameters.^33^ This analysis indicates that this BN-isostere shows similar inversion barrier to that of all-carbon derivative (see Fig. S39†).

**Fig. 9:**
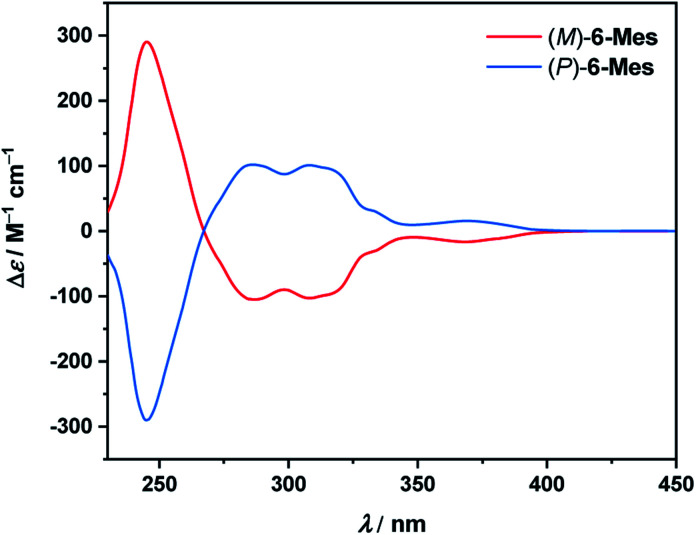
ECD spectra of (*M*)-6-Mes and (*P*)-6-Mes in CH_2_Cl_2_ (*c* = 2.2 × 10^−5^–2.5 × 10^−5^ M) at 20 °C.

The electronic circular dichroism (ECD) spectra of the resolved isomers of 6-Mes (Fig. 9) show a mirror-image relationship. A comparison of the experimental data with the TD-DFT simulated spectra (see Fig. S89†) allowed for the assignment of the absolute configuration of the first and second fractions as (*M*)- and (*P*)-enantiomers, respectively. The calculations reproduce reasonably well the general shape of the ECD spectrum of (*M*)-6-Mes. Enantiomer (*M*)-6-Mes exhibits negative Cotton effects (CEs) in the region from 267 to *ca.* 410 nm (Δ*ε* = −105 M^−1^ cm^−1^ at 286 nm, Δ*ε* = −103 M^−1^ cm^−1^ at 308 nm, Δ*ε* = −17 M^−1^ cm^−1^ at 369 nm) and an intense positive CD band at 245 nm (Δ*ε* = +290 m^−1^ cm^−1^). The absolute values of the absorption anisotropy factors (*g*_abs_) are typical of helicenes and amount to 4.9 × 10^−3^ at 245 nm, 5.1 × 10^−3^ at 286 nm and 5.0 × 10^−3^ at 308 nm. The weak lowest-energy CD band at 369 nm corresponds to |*g*_abs_| of 1.8 × 10^−3^. While the largest ECD of 6-Mes is comparable to that of carbo[6]helicene, the latter compound features somewhat higher maximal |*g*_abs_| value (9.2 × 10^−3^ in both MeCN and CH_2_Cl_2_ at 324 nm).^34,35^

## Conclusions

Protonation of aminoboranes by HNTf_2_ represents a new route to effect *N*-directed C–H borylation for accessing azaborines. In the systems studied herein it provides better outcomes than that achieved by the more established synthetic approach – activating aminoboranes with BBr_3_ and heating. This is attributed to the greater electrophilicity of borenium cations generated by aminoborane protonation relative to borenium cation equivalents generated by BBr_3_ coordination to N of the aminoborane. This HNTf_2_ mediated C–H borylation methodology thus enables access to azaborines otherwise challenging to access. This includes BN–[5] and [6]helicenes where the BN unit is located on the outer helix. BN incorporation slightly increases the strain present in these helicenes relative to all carbon analogues, but more notably it significantly enhances the photophysical properties (*ε* and *Φ*_F_), as observed previously for BN–[4]helicenes,^4^ while the absorption anisotropy factor (*g*_abs_) for a BN–[6]helicene is comparable to carbohelicene analogues. This work provides an example of BN incorporation into (configurationally stable) helically chiral organic frameworks leading to superior properties relative to all-carbon analogues along with a novel synthetic methodology that will facilitate access to azaborine materials.

## Data availability

Data associated with this article, including synthetic and computational details and compound characterization are available in the ESI.†

## Author contributions

MI and KY conceived the research concept and aims and analysed all data. KY performed all the synthetic work and the majority of the analytical and computational components of this project. DV investigated the separation of enantiomers of 6-Mes and 7-Mes and performed the configurational stability and ECD studies on 6-Mes (under the supervision of AN-K who also performed the simulations of the ECD spectra). SK performed the studies on the configurational stability of 1-Mes. GN and MU collected and solved all the crystal structures. Combined, KY, MI, DV and ANK drafted, reviewed and edited the manuscript.

## Conflicts of interest

There are no conflicts to declare.

## Supplementary Material

SC-013-D1SC06513K-s001

SC-013-D1SC06513K-s002
