# Supplementary material for: Enhanced N-directed electrophilic C–H borylation generates BN–[5]- and [6]helicenes with improved photophysical properties
Source: Chem Sci. 2022 Jan 4;13(4):1136–45. doi: 10.1039/d1sc06513k (PMC8790800; doi:10.1039/d1sc06513k)
Supplement: SC-013-D1SC06513K-s001 [file SC-013-D1SC06513K-s001.pdf]

Electronic Supplementary Material (ESI) for Chemical Science.
This journal is © The Royal Society of Chemistry 2022

Enhanced N-directed electrophilic C-H borylation generates BN-[5] and [6]-helicenes with improved photophysical properties

Kang Yuan, Daniel Volland, Sven Kirschner, Marina Uzelac, Gary S. Nichol,

Agnieszka Nowak-Król* and Michael J. Ingleson*

Table of Contents

S1. General description 2

S2. Synthesis of aniline precursors 4

S3. Synthesis of BN-helicenes 9

S4. Brief substrates scope assessment 28

S5. Resolution of (*rac*)-6-Mes and probing of racemisation barrier for 1-Mes and 6-Mes 46

S6. Crystal structures 51

S6. Electrochemical studies 54

S7. NMR spectra 56

S8. DFT calculations 77

S1. General description

All the experiments were performed under a nitrogen atmosphere in oven-dried glassware. Solvents were either obtained from an Inert PureSolv MD5 SPS or distilled from drying reagents: *ortho*-dichlorobenzene (CaH_2). All solvents were stored over activated 3 Å molecular sieves. Unless otherwise stated all chemicals were purchased from commercial sources and used as received. 2-Naphthylamine,[1] 1-bromonaphthalen-2-amine,[2] 4,4'-dibromo-2-nitrobiphenyl and 4,4'-dibromobiphenyl-2-amine[3] were prepared according to the literature procedures.

Column chromatography was performed using a CombiFlash NextGen 300+ AutoColumn or manually (40-63 μm silica).

Solution ^{1}H, $^{13}C\{^{1}H\}$, ^{11}B and $^{19}F\{^{1}H\}$ NMR spectra were recorded on 400 MHz and 500 MHz Bruker Spectrometers. ^{1}H and ^{13}C chemical shifts were referenced to residual solvent signals. ^{11}B and ^{19}F chemical shifts were referenced to external $BF_3{\cdot}OEt_2$ and hexafluorobenzene respectively. Resonances of carbon atoms directly bonded to boron atoms were not always observed due to quadrupolar relaxation effects.

UV-Vis absorption spectra were recorded on a SHIMADZU UV-1800 UV spectrophotometer. Emission spectra were recorded on a SHIMADZU RF-6000 spectro fluorophotometer. Solution quantum yields (Φ_{PL}) were calculated relative to 9,10-diphenylanthracene.[4]

High resolution mass spectrometry was performed at the Resource Centre for Advanced Mass Spectrometry based in the School of Chemistry at the University of Edinburgh.

Cyclic voltammetry (CV) measurements were performed under a N_2 atmosphere using a CH-Instrument 1110C Electrochemical/Analyzer potentiostat. Experiments were conducted using a 1 mM analyte solution with 0.1 M tetrabutylammonium hexafluorophosphate as the supporting electrolyte in THF with a scan rate of 100 mV s^{-1}. A glassy carbon electrode was used as the working electrode with platinum wires as the counter electrode and the reference electrode. All potentials were calibrated against the ferrocene/ferrocenium (Fc/Fc^{+}) redox couple.

---

1 Voth, S.; Hollett, J. W. and McCubbin, J. A. *J. Org. Chem.* 2015 **80** (5), 2545-2553.

2 Ortgies, S. and Breder, A. *Org. Lett.* 2015 **17** (11), 2748-2751.

3 Xu, R., Wang, Y., Duan, X., Lu, K., Micheroni, D., Hu, A. and Lin, W. *J. Am. Chem. Soc.* 2016 **138** (7), 2158-2161

4 Morris, J. V., Mahaney M. A. and J. R. Huber, *J. Phys. Chem.*, 1976, **80**, 969–974.

Investigation of the chiral resolution was performed on an analytical HPLC-system from JASCO equipped with a column oven (CO-4060), an autosampler (AS-4050), and a photodiode array detector (MD-4010).

Resolution of the enantiomers was carried out with a semi-preparative HPLC-setup from JASCO, equipped with an UV/vis-detector (UV-4070).

Circular Dichroism spectra were measured with a JASCO spectropolarimeter equipped with a Peltier temperature controller.

S2. Synthesis of aniline precursors

S2.1 General procedure 1: preparations of simple anilines

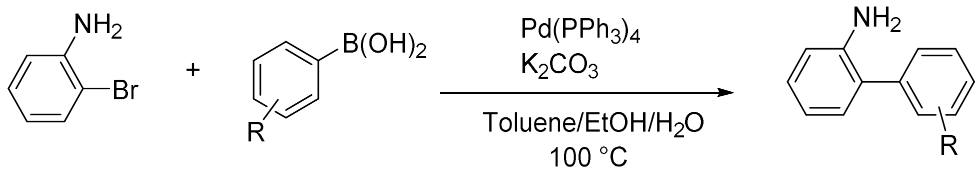

To a mixture of 2-bromoaniline (1 eq.), arylboronic acid (1.5 eq.), K_2CO_3 (4 eq.) and $Pd(PPh_3)_4$ (0.03-0.05 eq.) was added degassed toluene-ethanol-water mixed solvents (V:V:V 3:2:1). The mixture was heated to 100 °C (oil bath temperature) for 24 – 48 hours. Upon cooling, the mixture was quenched with water and extracted with ethyl acetate. The organic layer was dried with Na_2SO_4 and concentrated *in vacuo*. The product was purified by flash chromatography using a petroleum ether: dichloromethane eluent.

S2.1 Procedures for the preparation of sterically hindered anilines

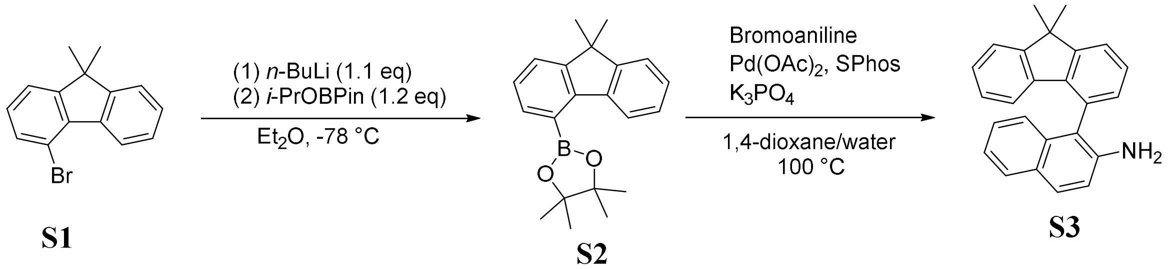

S1 **S2** **S3**

4-Bromo-9,9-dimethyl-9*H*-fluorene (S1): The compound was purchased from Fluorochem and was used as obtained. However, we could not find any reported NMR data of this compound. Thus, the NMR characterisation data are provided herein.

^{1}**H** NMR (500 MHz, $CDCl_3$) δ = 8.59 (ddd, *J* = 6.8, 3.6, 1.7 Hz, 1H), 7.52-7.50 (m, 1H), 7.46-7.44 (m, 1H), 7.41-7.38 (m, 3H), 7.20 – 7.11 (m, 1H), 1.49 (s, 6H).

^{13}**C{^{1}H}** NMR (126 MHz, $CDCl_3$) δ = 138.6, 137.7, 132.1, 128.2, 128.0, 126.9, 123.7, 122.5, 121.6, 117.2, 47.0, 27.5.

2-(9,9-dimethyl-9*H*-fluoren-4-yl)-4,4,5,5-tetramethyl-1,3,2-dioxaborolane (S2): To a solution of 4-bromo-9,9-dimethyl-9H-fluorene (**S1**) (2.73g, 10 mmol) in 25 mL Et_2O was added *n*-BuLi (6.88 mL, 1.6 M in hexane, 11 mmol) dropwise at -78 °C. The resulting mixture was stirred at -78 °C for two hours. Then *i*PrOBPin (2.45 mL, 12 mmol) was added slowly at -78 °C. The reaction was allowed to gradually warm to room temperature and stir overnight and a white suspension was obtained. The reaction was then quenched

with saturated NH_4Cl aqueous solution. The product was extracted with ethyl acetate. The organic layer was dried over $MgSO_4$ and concentrated in *vacuo*. The crude product was purified by column chromatography on silica gel (hexane: ethyl acetate 100:1 to 20:1). A total of 850 mg (26.5% yield) desired product was obtained (Rf = 0.45 in hexane: ethyl acetate 20:1). In addition, 1.76 g 4-bromo-9,9-dimethyl-9H-fluorene (**S1**) was recovered.

^{1}H NMR (500 MHz, $CDCl_3$) δ = 8.68-8.65 (m, 1H), 7.78 (dd, *J* = 7.4, 1.3 Hz, 1H), 7.51 (dd, J = 7.5, 1.3 Hz, 1H), 7.43-7.41 (m, 1H), 7.34-7.28 (m, 3H), 1.46 (s, 6H), 1.45 (s, 12H).

^{11}B NMR (128 MHz, $CDCl_3$) δ = 31.82.

^{13}C{^{1}H} NMR (101 MHz, $CDCl_3$) δ =154.2, 153.9, 144.0, 140.5, 135.1, 127.3, 126.9, 126.3, 125.3, 124.0, 122.3, 84.2, 46.1, 27.5, 25.1.

HRMS (ESI): Calcd for $C_{21}H_{26}BO_2^+$ $[M+H]^+$: 321.2020, found: 321.2019.

1-(9,9-dimethyl-9*H*-fluoren-4-yl)naphthalen-2-amine (S3): To a mixture of 1-bromo-2-naphthylamine (832 mg, 3 mmol), **S2** (1g, 2.5 mmol), K_3PO_4 (2.544 g, 12 mmol), palladium(II) acetate (70 mg, 0.312 mmol), SPhos (256 mg, 0.624 mmol) was added degassed 1,4-dioxane (12 mL) and water (1.2 mL). The reaction was stirred at 100 °C for 40 hours. The mixture was then diluted with water and extracted with ethyl acetate. The organic layer was dried over $MgSO_4$ and concentrated in *vacuo*. The crude product was purified by column chromatography on silica gel (hexane: ethyl acetate 100:1 to 5:1, Rf= 0.30 in hexane: ethyl acetate 5:1). The desired compound was obtained as a brown solid (700 mg, 67%).

^{1}H NMR (500 MHz, $CDCl_3$) δ = 7.82 (d, *J* = 8.8 Hz, 1H), 7.80 – 7.78 (m, 1H), 7.55 (d, *J* = 7.5 Hz, 1H), 7.47 (t, *J* = 7.5 Hz, 1H), 7.39 (d, *J* = 7.5 Hz, 1H), 7.24 – 7.19 (m, 4H), 7.18 – 7.11 (m, 2H), 6.87 (t, *J* = 7.6 Hz, 1H), 6.46 (d, *J* = 7.8 Hz, 1H), 3.55 (br, 2H), 1.57 (s, 3H), 1.56 (s, 3H).

^{13}C NMR (126 MHz, $CDCl_3$) δ = 155.2, 153.9, 141.1, 138.9, 138.2, 133.7, 131.4, 130.5, 129.2, 128.4, 128.1, 128.0, 127.2, 127.2, 126.7, 124.4, 122.5, 122.5 (two peaks overlap), 122.3, 122.3 (two peaks overlap), 118.5, 118.3, 46.6, 27.8, 27.7.

HRMS (ESI): Calcd for $C_{25}H_{22}N^+$ $[M+H]^+$: 336.1747, found: 336.1751.

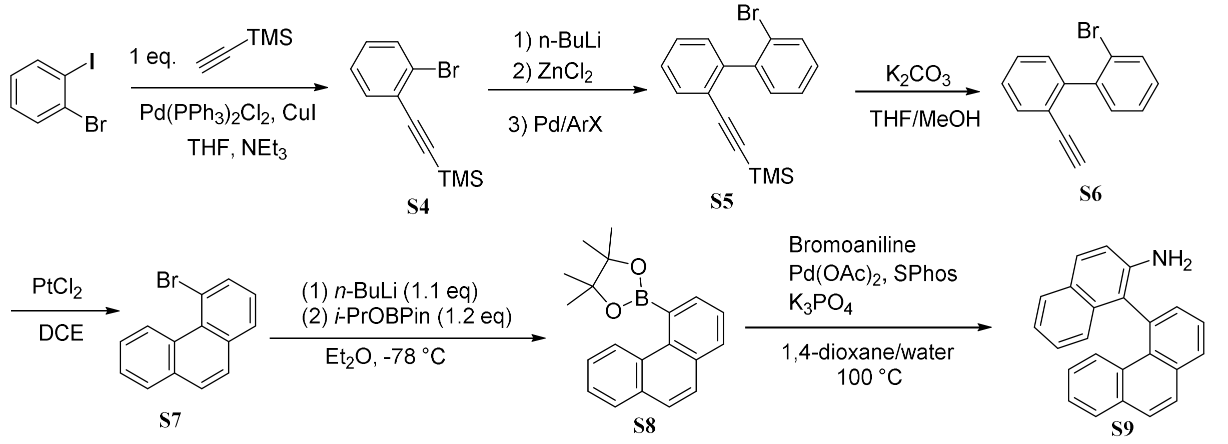

1-Bromo-2-[2-(trimethylsilyl)ethynyl]benzene (S4): To a solution of 1-bromo-2-iodobenzene (11.32 g, 40 mmol), $Pd(PPh_3)_2Cl_2$ (561 mg, 0.8 mmol) and CuI (456 mg, 2.4 mmol) in THF (50 mL) and triethylamine (10 ml) was added trimethylsilylacetylene (8.31 mL, 60 mmol). The reaction was stirred at room temperature for 4 days. Then saturated NH_4Cl aqueous solution was added to the reaction and the mixture was extracted with ethyl acetate. The organic layer was dried over $MgSO_4$ and concentrated in *vacuo*. The crude product was purified by silica gel column chromatography eluted with hexane to yield the desired product as a light-yellow oil. Yield 9.6 g, 95%:

^{1}**H** NMR (500 MHz, $CDCl_3$) δ = 7.57 (dd, *J* = 8.1, 1.2 Hz, 1H), 7.49 (dd, *J* = 7.7, 1.7 Hz, 1H), 7.24 (td, *J* = 7.6, 1.3 Hz, 1H), 7.15 (ddd, *J* = 8.0, 7.4, 1.7 Hz, 1H), 0.28 (s, 9H). The spectrum is in agreement with literature data.[5]

((2'-bromo-[1,1'-biphenyl]-2-yl)ethynyl)trimethylsilane (S5): To a solution of **S4** (5.064 g, 20 mmol) in 40 mL anhydrous THF was added *n*-BuLi (14.1 mL, 1.6 M, 23 mmol) dropwise at -78 °C. The mixture was stirred at -78 °C for 2 hours after the addition was completed. Then $ZnCl_2$ (2.726 g, 20 mmol) in 22 mL THF was added to the reaction slowly at -78 °C. The reaction was stirred at -78 °C for 40 minutes and at 0 °C for 30 minutes. Then $Pd(PPh_3)_4$ (770 mg, 0.67 mmol) and 1-bromo-2-iodobenzene (6.789 g, 24 mmol) were added to the reaction mixture. After the addition, the reaction was heated to 60 °C for 40 hours. The reaction was then quenched with saturated NH_4Cl aqueous solution and extracted with ethyl acetate. The organic layer was dried over $MgSO_4$ and concentrated in *vacuo*. The crude product was purified by silica gel column chromatography (hexane then hexane: DCM 5:1, Rf= 0.38 in hexane: DCM 5:1) to yield the desired product as a light-yellow oil (4.65 g, 71%).

---

5 Körner, C., Starkov, P., and Sheppard, T. D. *J. Am. Chem. Soc.* 2010 **132** (17), 5968-5969

^{1}H NMR (500 MHz, $CDCl_3$) δ = 7.60 (dd, *J* = 8.0, 1.2 Hz, 1H), 7.52 – 7.47 (m, 1H), 7.34 – 7.24 (m, 4H), 7.23 – 7.21 (m, 1H), 7.16 (ddd, *J* = 8.0, 6.9, 2.2 Hz, 1H), -0.04 (s, 9H).

^{13}C{^{1}H} NMR (126 MHz, $CDCl_3$) δ = 144.6, 141.8, 132.5, 131.9, 131.5, 129.5, 129.0, 128.3, 127.7, 126.9, 123.7, 123.0, 103.9, 98.3, -0.3.

HRMS (ESI): Calcd for $C_{17}H_{18}BrSi^+$ $[M+H]^+$: 329.0356, found: 329.0364.

2-Bromo-2'-ethynyl-1,1'-biphenyl (S6): A mixture of **S5** (6g, 18.2 mmol) and K_2CO_3 in THF (100 mL) and methanol (100 mL) mixed solvents was stirred at room temperature for 16 hours. The solvent was then removed and the obtained mixture was diluted with water and extracted with dichloromethane. The organic layer was dried over Na_2SO_4 and concentrated in *vacuo*. The desired compound was obtained as a white solid (4.53g, 97%) after silica gel column chromatography with hexane as the eluent.

^{1}H NMR (500 MHz, $CDCl_3$) δ = 7.67 (dd, *J* = 8.0, 1.2 Hz, 1H), 7.64 – 7.57 (m, 1H), 7.42 (td, *J* = 7.6, 1.5 Hz, 1H), 7.39 – 7.32 (m, 3H), 7.30 – 7.27 (m, 1H), 7.26 – 7.22 (m, 1H), 2.95 (s, 1H). The NMR data are in agreement with literature data.[6]

4-Bromophenanthrene (S7): The compound was prepared according to the reported procedure. The desired compound was obtained as a white solid after silica gel column chromatography with hexane as the eluent.

^{1}H NMR (400 MHz, $CDCl_3$) δ = 10.07 (m, 1H), 8.00 (dd, *J* = 7.6, 1.4 Hz, 1H), 7.92 – 7.88 (m, 1H), 7.85 (dd, *J* = 7.8, 1.4 Hz, 1H), 7.75 (d, *J* = 8.7 Hz, 1H), 7.70 – 7.62 (m, 3H), 7.38 (t, *J* = 7.7 Hz, 1H).The NMR data are in agreement with the literature data.[2]

4,4,5,5-Tetramethyl-2-(phenanthren-4-yl)-1,3,2-dioxaborolane (S8): To a solution of 4-bromophenanthrene (341 mg, 1.33 mmol) in 5 mL Et_2O was added *n*-BuLi (0.91 mL, 1.6 M in hexane, 1.46 mmol) dropwise at -78 °C. The resulting mixture was stirred at -78 °C for one hour. Then *i*PrOBPin (0.33 mL, 1.6 mmol) was added slowly at -78 °C. The reaction was allowed to gradually warm to room temperature and stirred overnight and a white suspension was obtained. The reaction was then quenched with saturated NH_4Cl aqueous solution. The product was extracted with ethyl acetate. The organic layer was dried over Na_2SO_4 and concentrated in *vacuo*. The crude product was purified by column

[6] Urbano, A., Del Hoyo, A. M., Martínez-Carrión, A., and Carreño, M. C. *Org. Lett.* 2019, **21**(12), 4623–4627.

chromatography on silica gel (hexane: ethyl acetate 100:1 to 4:1). The desired compound was obtained as a white solid (350 mg, 87%).

^{1}H NMR (400 MHz, $CDCl_3$) δ = 8.56 – 8.54 (m, 1H), 7.92 (dd, *J* = 7.9, 1.4 Hz, 1H), 7.90 – 7.87 (m, 1H), 7.84 (dd, *J* = 7.0, 1.4 Hz, 1H), 7.75 – 7.70 (m, 2H), 7.63 – 7.51 (m, 3H). The NMR data are in agreement with literature data.[7]

1-(phenanthren-4-yl)naphthalen-2-amine (S9): To a mixture of 1-bromo-2-naphthylamine (1.64 g, 6.2 mmol), **S8** (1.876g, 6.2 mmol), K_3PO_4 (5.3 g, 25 mmol), palladium (II) acetate (139 mg, 0.62 mmol) and SPhos (509 mg, 1.24 mmol) was added degassed 1,4-dioxane (20 mL) and water (2 mL). The reaction was stirred at 100 °C for 40 hours. The mixture was then diluted with water and extracted with ethyl acetate. The organic layer was dried over $MgSO_4$ and concentrated in *vacuo*. The crude product was purified by column chromatography on silica gel (hexane: ethyl acetate 100:1 to 5:1). The desired compound was obtained as a white solid (Yield: 840 mg, 41%).

^{1}H NMR (500 MHz, $CDCl_3$) δ = 8.02 (dd, *J* = 7.9, 1.6 Hz, 1H), 7.91 – 7.76 (m, 6H), 7.75 – 7.69 (m, 1H), 7.48 (dd, *J* = 7.2, 1.5 Hz, 1H), 7.39 (ddd, *J* = 7.9, 6.9, 1.1 Hz, 1H), 7.23 (ddd, *J* = 8.1, 6.4, 1.6 Hz, 1H), 7.18 – 7.09 (m, 3H), 7.01 (ddd, *J* = 8.6, 6.9, 1.6 Hz, 1H), 3.54 (br, 2H).

^{13}C{^{1}H} NMR (126 MHz, $CDCl_3$) δ = 140.8, 134.5, 134.3, 133.6, 133.1, 132.6, 131.0, 129.9, 129.6, 129.1, 128.6, 128.6, 128.1, 128.0, 128.0, 127.1, 126.8, 126.6, 126.5, 125.9, 124.6, 122.6, 122.6, 118.5.

HRMS (ESI): Calcd for $C_{24}H_{18}N^{+}$ $[M+H]^{+}$: 320.1434, found: 320.1433.

[7] Full, J., Panchal, S. P., Götz, J., Krause, A. M. and Nowak-Król, A. *Angew. Chem., Int. Ed.* 2021, **60**(8), 4350–4357

S3. Synthesis of BN-helicenes

S3.1 General Procedure 2: preparation of amine-borane adducts.

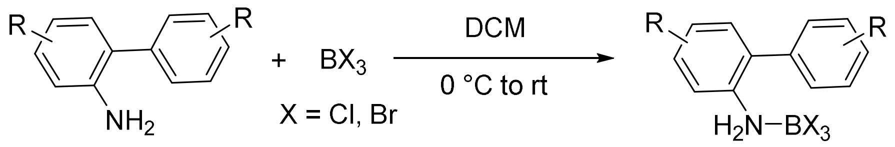

To a solution of BX_3 (X = Cl, Br) (1.3 eq., 1 M in dichloromethane) was added the corresponding aniline (1 eq., 0.4-0.6 M in dichloromethane) at 0 °C. The mixture was stirred at 0 °C for 30 minutes and then was brought to room temperature for another 30 minutes. Then the solvent was removed in *vacuo* and the residue was washed with pentane. The purity of the obtained solids is typically > 95% (by NMR spectroscopy) and was used without further purification.

S3.2 Attempted borylation with BX_3 to access BN-5-helicene

S3.2.2 Borylation with BCl_3 and $AlCl_3$ (Dewar's conditions)

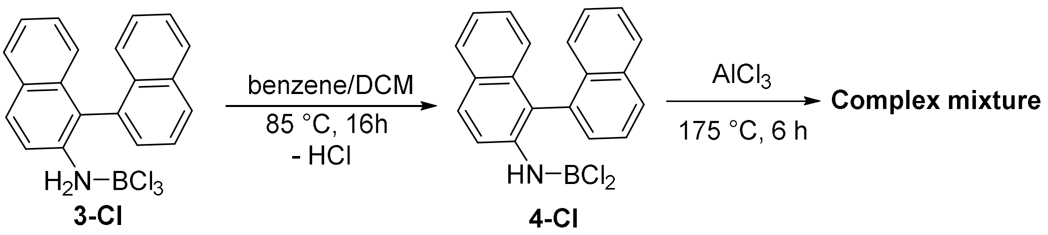

A solution of the amine-borane adduct **3-Cl** in benzene/dichloromethane (3 mL/1mL) mixed solvents was heated to 80 °C under a dynamic N_2 flow for 16 hours. Then a small portion of the reaction mixture was taken for NMR studies and the data indicated the clean formation of the aminoborane **4-Cl**. The solvent was then removed and a white solid **4-Cl** was obtained. To the aminoborane **4-Cl** was added $AlCl_3$ (10 mg). The mixed solids were heated to 175 °C and the aminoborane **4-Cl** melted. After 6 hours a dark mixture was obtained. The mixture was dissolved in DCM for NMR studies. The ^{11}B NMR spectrum indicates no formation of the azaborine **1-Cl**. Note: compound **1-Cl** is not reported in literature. We found that strain has little influence on the ^{11}B chemical shift for helicene containing azaborine units, for example, for **1-Br** and **11-Br**, the ^{11}B NMR chemical shifts are found at 33.5 ppm. Thus δ_{11B} of **1-Cl** should be similar to that of **9-Cl** (δ_{11B} = 34.6).

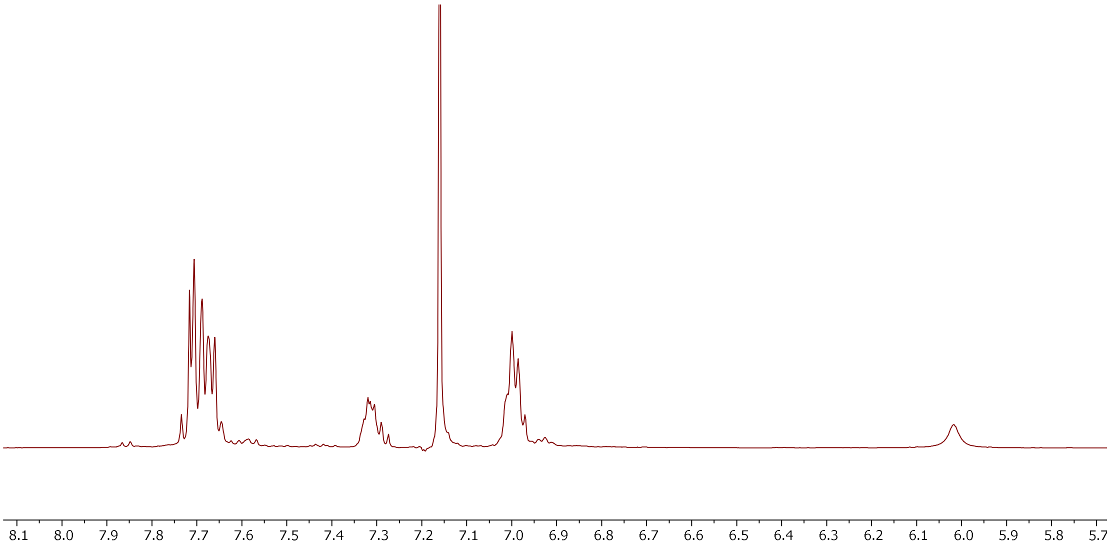

Figure S1. Crude ^{1}H NMR spectrum of **4-Cl** in benzene/dichloromethane mixed solvents.

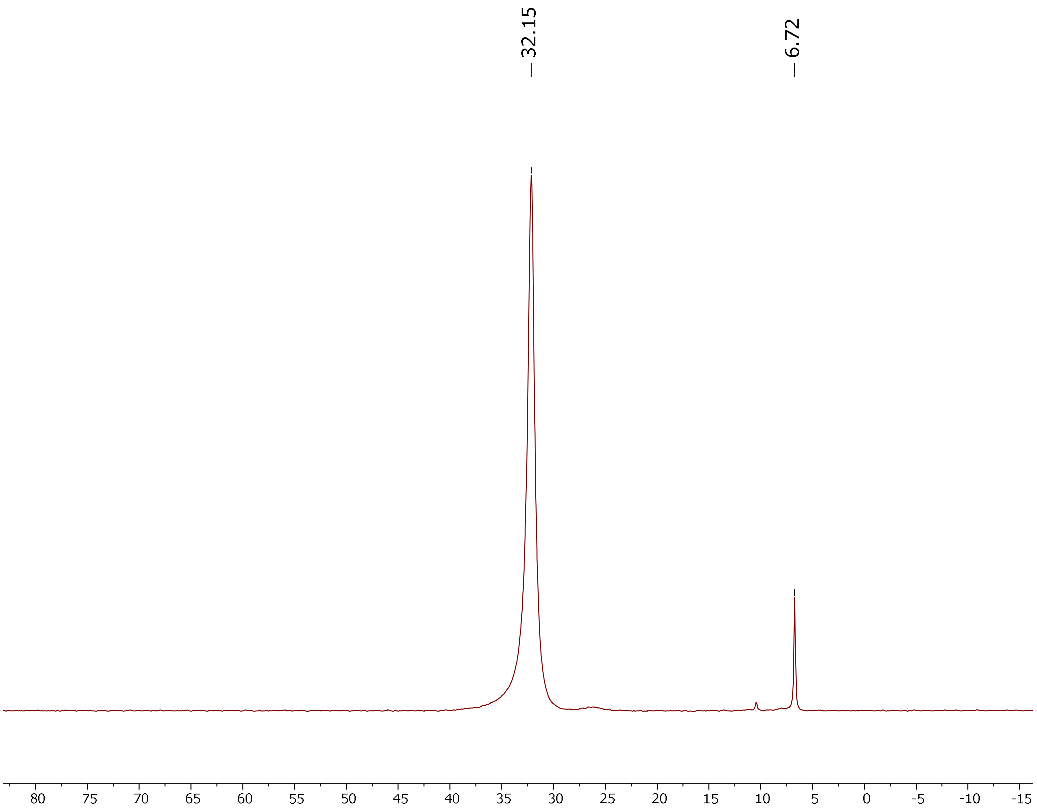

Figure S2. Crude ^{11}B NMR spectrum of **4-Cl** in benzene/dichloromethane mixed solvents, small amount of the amine-borane adduct **3-Cl** presents as an impurity.

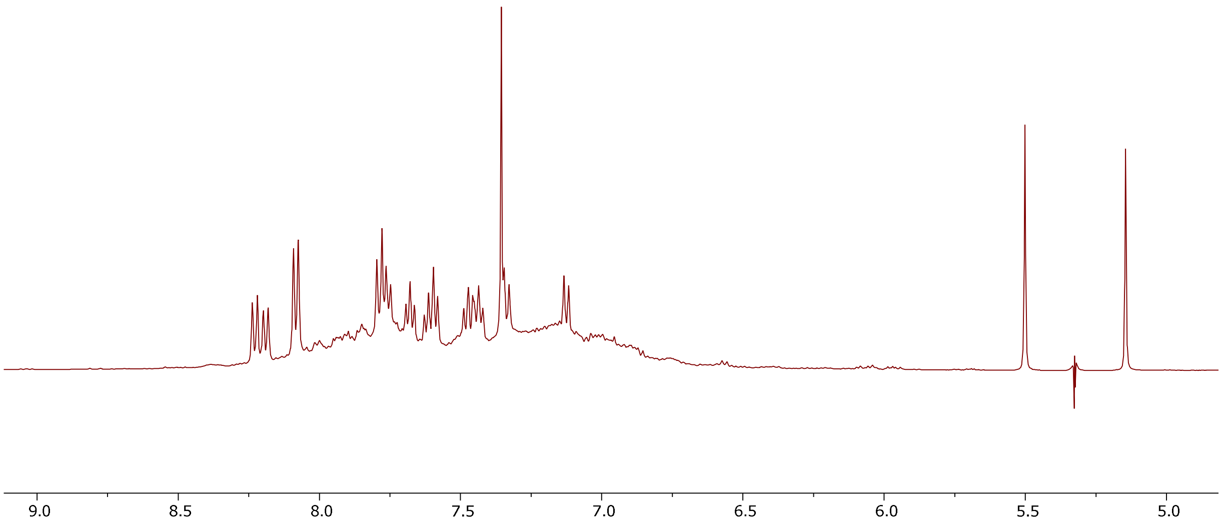

Figure S3. Crude ^{1}H NMR spectrum of **4-Cl** and $AlCl_3$ reaction mixture (in dichloromethane).

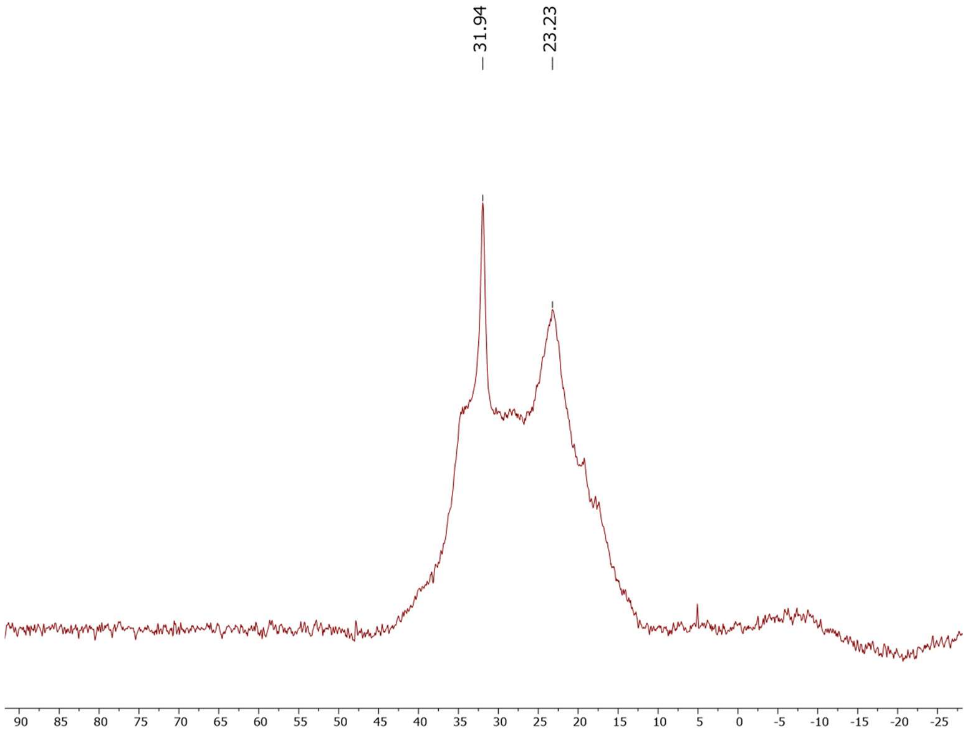

Figure S4. Crude ^{11}B NMR spectrum of **4-Cl** and $AlCl_3$ reaction mixture (in dichloromethane). Note B-Cl azaborines typically resonate at 34.6 ppm, confirming **1-Cl** is not formed.

S3.2.3 Borylation with excess BBr_3

S3.2.3a Borylation in an open system (under the flow of N_2)

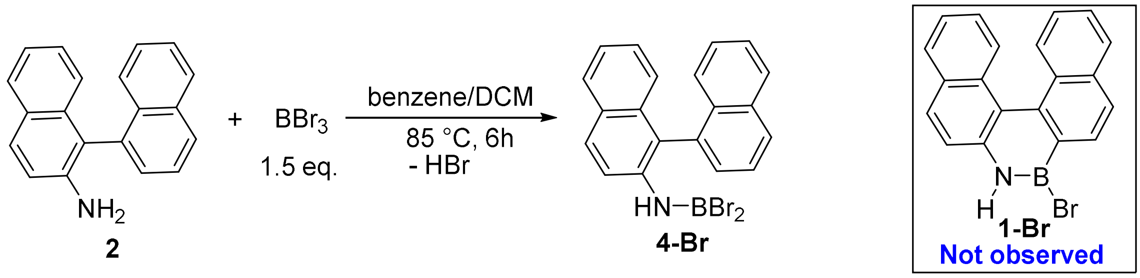

To a solution of amine **2** (54 mg, 0.2 mmol) in 2.5 mL benzene was added BBr_3 (0.3 mL, 1M in DCM) in one portion. The resulting mixture was stirred at 0 °C for 5 minutes and then at room temperature for 5 minutes. After that, the reaction was heated to 85 °C for 6 hours. Then 0.5 mL of the reaction mixture was taken for NMR studies. Both ^{1}H and ^{11}B NMR spectra indicate the formation of the dehydrobromination product **4-Br** as the major product. No azaborine **1-Br** was produced based on the ^{11}B NMR spectrum.

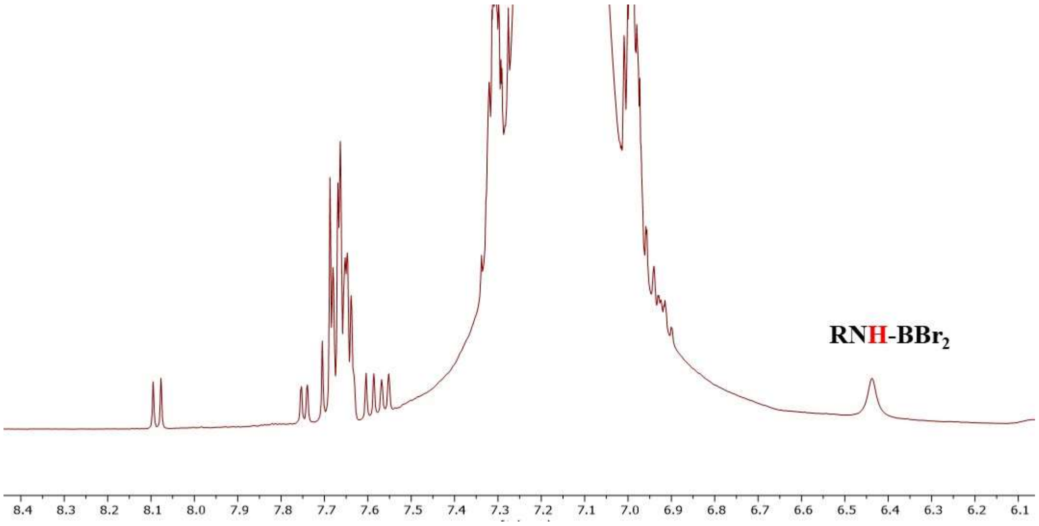

Figure S5. In-situ ^{1}H NMR spectrum (in benzene) for the reaction of amine **2** and BBr_3 under thermal conditions. The formation of the dehydrobromination product **4-Br** was confirmed by the characteristic RNH-BBr_2 resonance (and δ_{11B}, vide infra).

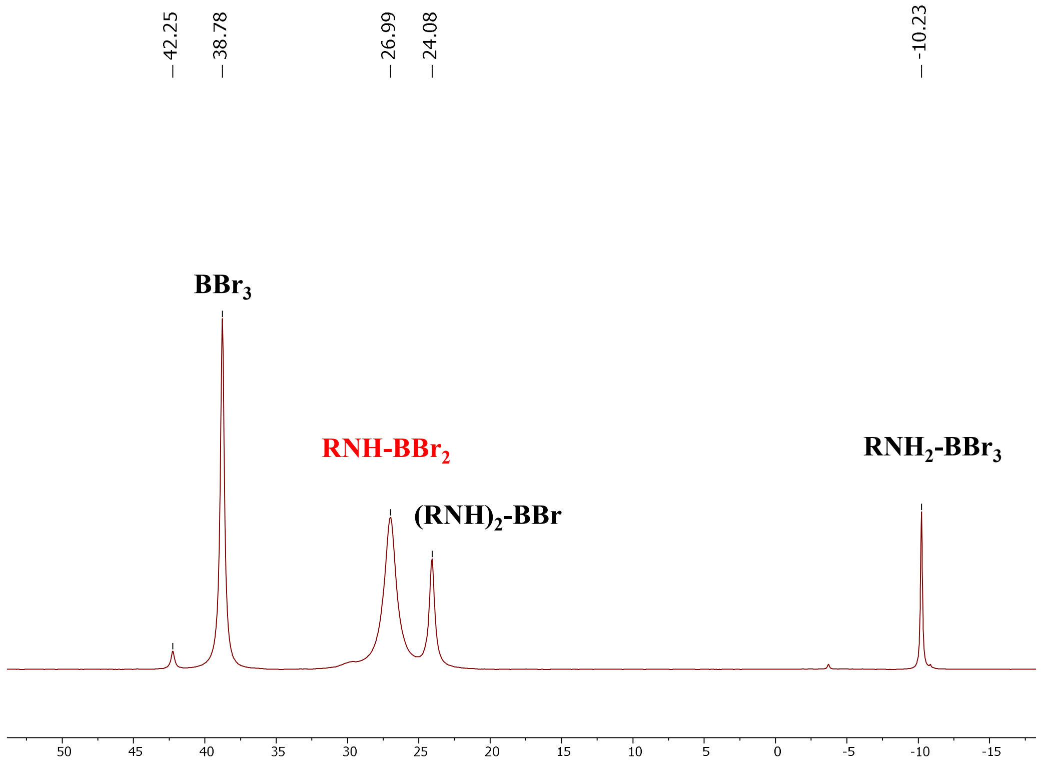

Figure S6. In-situ ^{11}B NMR spectrum (in benzene) for the reaction of amine **2** and BBr_3 under thermal conditions. The ^{11}B resonance at 26.99 ppm indicates the formation of the dehydrobromination product **4-Br**. No azaborine **1-Br** (^{11}B = ~ 33 ppm) formed under these reaction conditions.

S3.2.3b Borylation in a closed system

The borylation reactions in a closed system were performed in two different solvents (dichloromethane and ortho-dichlorobenzene). For both reactions in different solvents, only a small amount of desired product was formed.

(1) In dichloromethane

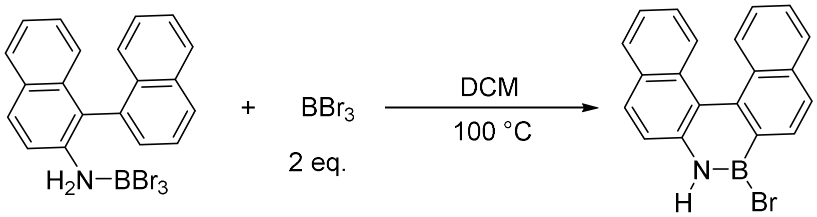

3-Br **1-Br**

To a J-Young NMR tube charged with the amine-borane adduct **3-Br** (26 mg, 0.05 mmol) was added 0.6 mL DCM and 2 eq. BBr_3 (0.1 mL, 1 M in DCM). The tube was sealed and heated to 100 °C (heating block temperature). After 21 hours, only a small amount of azaborine **1-Br** was produced based on the ^1H and ^{11}B NMR spectra. Extending the reaction time (48 hours at 100 °C) led to more azaborine **1-Br** (~ 30%) but unidentified by-products formed as well.

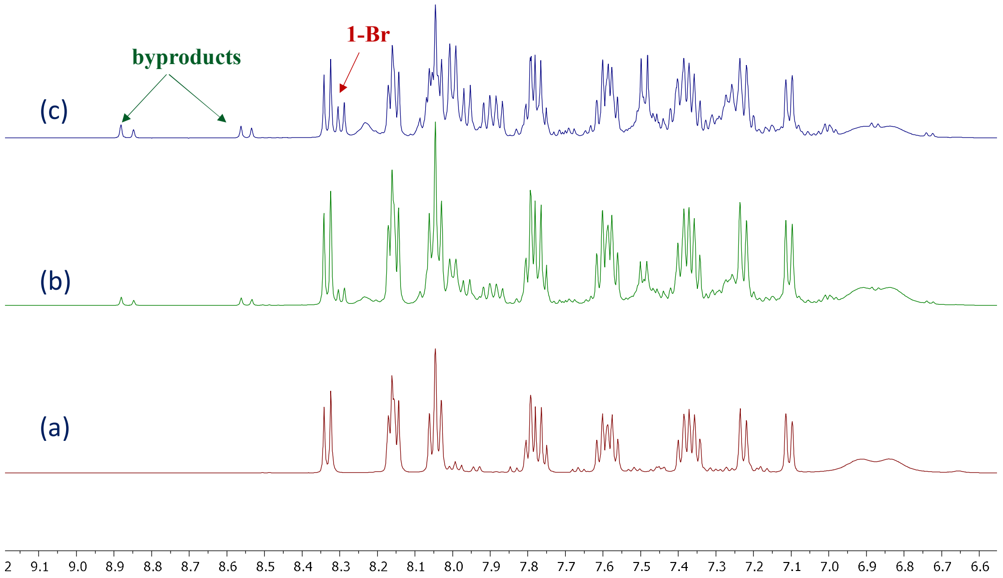

Figure S7. In-situ ^1H NMR spectra for the borylation reaction of **3-Br** with excess BBr_3 in dichloromethane: (a) the mixture of **3-Br** and 2 eq. BBr_3; (b) 100 °C, 21 hours; (c) 100 °C, 48 hours.

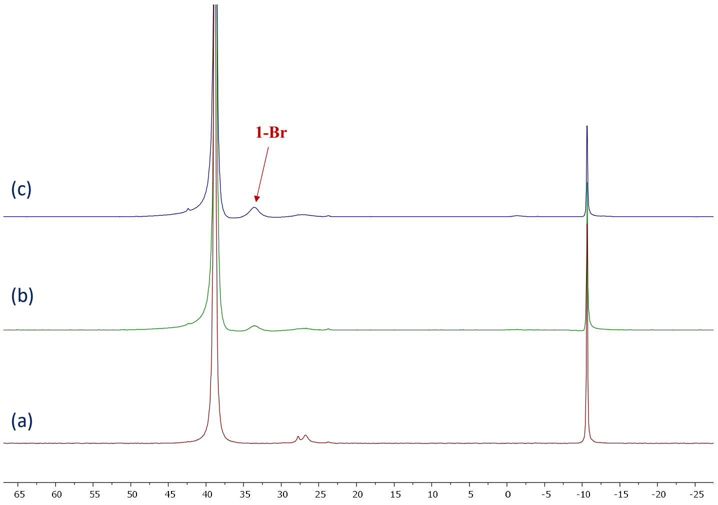

Figure S8. In-situ ^{11}B NMR spectra for the borylation reaction of **3-Br** with excess BBr_3 in dichloromethane: (a) the mixture of **3-Br** and 2 eq. BBr_3; (b) 100 °C, 21 hours; (c) 100 °C, 48 hours.

(2) In *ortho*-dichlorobenzene

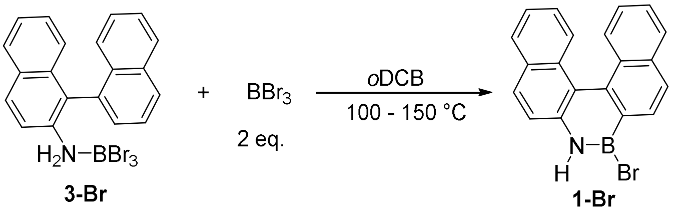

To a J-Young NMR tube charged with the amine-borane adduct **3-Br** (26 mg, 0.05 mmol) was added 1 mL *ortho*-dichlorobenzene (*o*DCB) and extra BBr_3 (0.5 mL, 0.2 M in *o*DCB). The tube was sealed and heated to 100 °C. After 40 hours, only a trace amount of azaborine **1-Br** was produced based on the ^1H NMR spectrum. Increasing the reaction temperature to 150 °C led to a**3-Br**:**1-Br** ratio of 2:1 after 24 hours. However, the ratio of **3-Br**:**1-Br** remains the same for an extra 24 hours heating and a significant amount of unidentified by-products formed. Upon cooling, dark blue precipitate formed in the NMR tube. The precipitate could not be dissolved in dichloromethane. Thus based on the ratio of **3-Br** : **1-Br** and the presence of unidentified by-products the conversion to **1-Br** is < 30%.

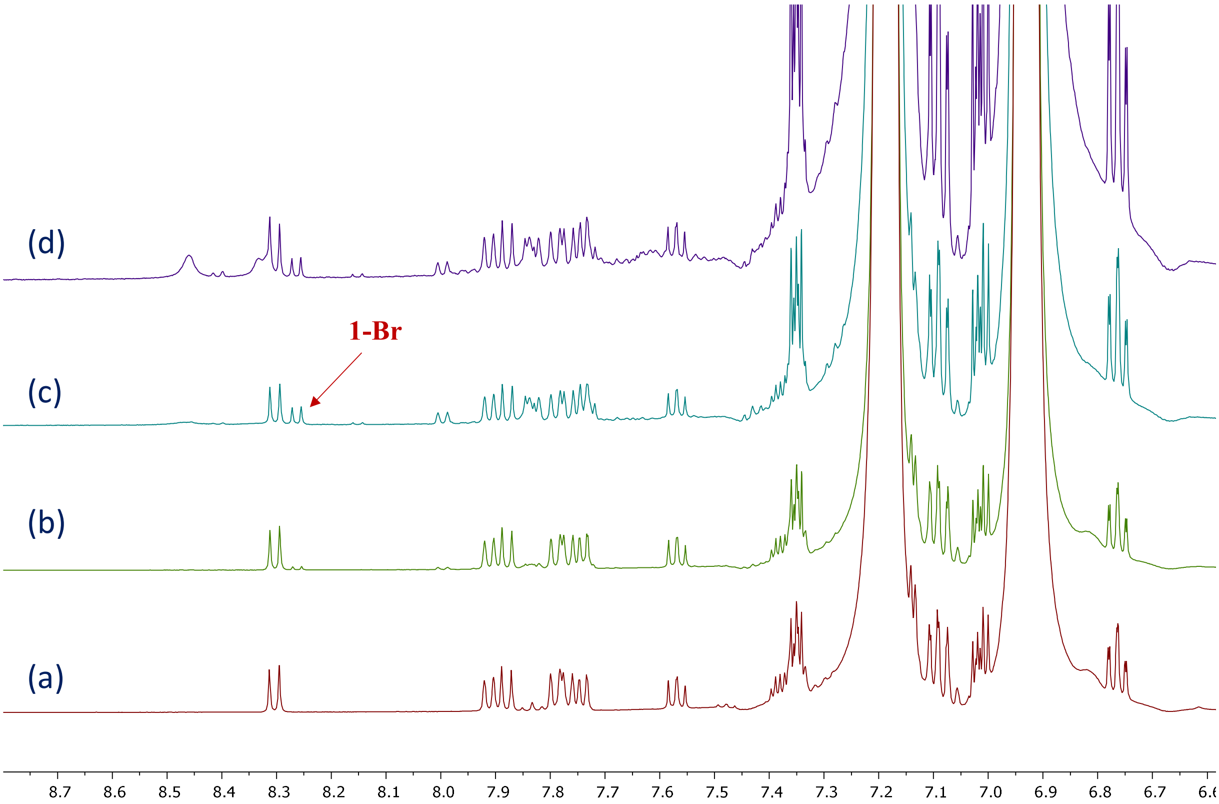

Figure S9. In-situ ^{1}H NMR spectra for the borylation of **3-Br** with excess BBr_3 in *o*DCB: (a) the mixture of **3-Br** and 2 eq. BBr_3; (b) 100 °C, 40 hours; (c) 150 °C, 24 hours; (d) 150 °C, 48 hours.

S3.3 Proton catalysed borylation for the synthesis of BN-helicenes

S3.3.1 Synthesis of 1-Mes

(a) Synthesis of 1-Mes via the open system procedure (route (i))

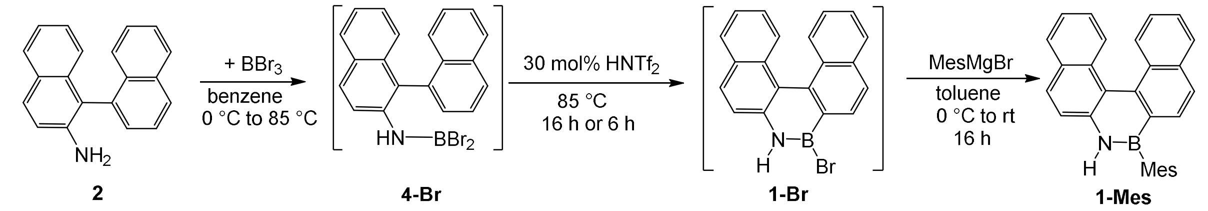

Large-scale Reaction

To a solution of the amine **2** (600 mg, 2.2 mmol, 1 eq.) in 10 mL benzene was added BBr_3 (3.3 mL, 1 M in dichloromethane, 1.5 eq.) in one portion at 0 °C. The reaction was stirred at 0 °C for 30 minutes and then at room temperature for 5 minutes. The solution then was heated to 85 °C for 5 hours under a dynamic flow of N_2. Then $HNTf_2$ (30 mol%) was added directly to the reaction and the resulting mixture was heated to 85 °C for 16 hours. The solvent and excess BBr_3 were removed after the heating. The resultant solid was dissolved in toluene. To the solution was added 2-mesitylmagnesium bromide (1.5 eq., 1 M in Et_2O) at 0 °C. The solution was allowed to gradually warm to room temperature and stirred overnight. The reaction was then quenched with 1 M HCl aqueous solution. The product was extracted with Et_2O and the organic layer was dried over Na_2SO_4 and concentrated in *vacuo*. The crude product was purified by column chromatography on silica gel (hexane: DCM 100:1 to 4:1). The desired compound was obtained as a light-yellow solid (500 mg, 56%).

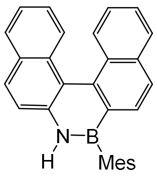

1-Mes

^{1}H NMR (500 MHz, $CDCl_3$) δ = 8.18 (d, *J* = 8.6 Hz, 1H), 8.03 (d, *J* = 8.6 Hz, 1H), 7.96 – 7.90 (m, 3H), 7.87 – 7.81 (m, 2H), 7.77 (dd, *J* = 8.2, 1.4 Hz, 1H), 7.55 (t, *J* = 7.5 Hz, 1H), 7.45 (d, *J* = 8.5 Hz, 1H), 7.40 (t, *J* = 7.4 Hz, 1H), 7.29 – 7.24 (m, 2H), 6.98 (s, 1H), 6.96 (s, 1H), 2.39 (s, 3H), 2.23 (s, 3H), 2.04 (s, 3H).

^{11}B NMR (160 MHz, $CDCl_3$) δ = 38.81.

^{13}C{^{1}H} NMR (126 MHz, $CDCl_3$) δ = 140.8, 140.7, 138.4, 138.1, 137.9, 135.4, 132.2, 130.9, 130.6, 130.0, 129.7, 129.2, 128.4, 128.1, 128.1, 127.4, 127.3, 127.0, 126.6, 125.1, 124.1, 124.0, 119.8, 117.3, 23.11, 23.05, 21.4.

HRMS (ESI): Calcd for $C_{29}H_{25}NB^{+}$ $[M+H]^{+}$: 398.2075, found: 398.2069.

Small-scale Reaction (in-situ NMR monitoring)

To a solution of the amine **2** (54 mg, 0.2 mmol, 1 eq.) in 10 mL benzene was added BBr_3 (0.3 mL, 1 M in dichloromethane, 1.5 eq.) in one portion at 0 °C. The reaction was stirred at 0 °C for 5 minutes and then at room temperature for 5 minutes. The solution then was heated to 85 °C for 6 hours under a dynamic flow of N_2. Then $HNTf_2$ (30 mol%) was added directly to the reaction and the resulting mixture was heated to 85 °C for 6 hours. The solvent and excess BBr_3 were removed after the heating. The resultant solid was re-dissolved in dichloromethane for NMR spectroscopy studies. Both ^{1}H and ^{11}B NMR spectra indicate a clean formation of the desired product **1-Br** (NMR Yield > 90%).

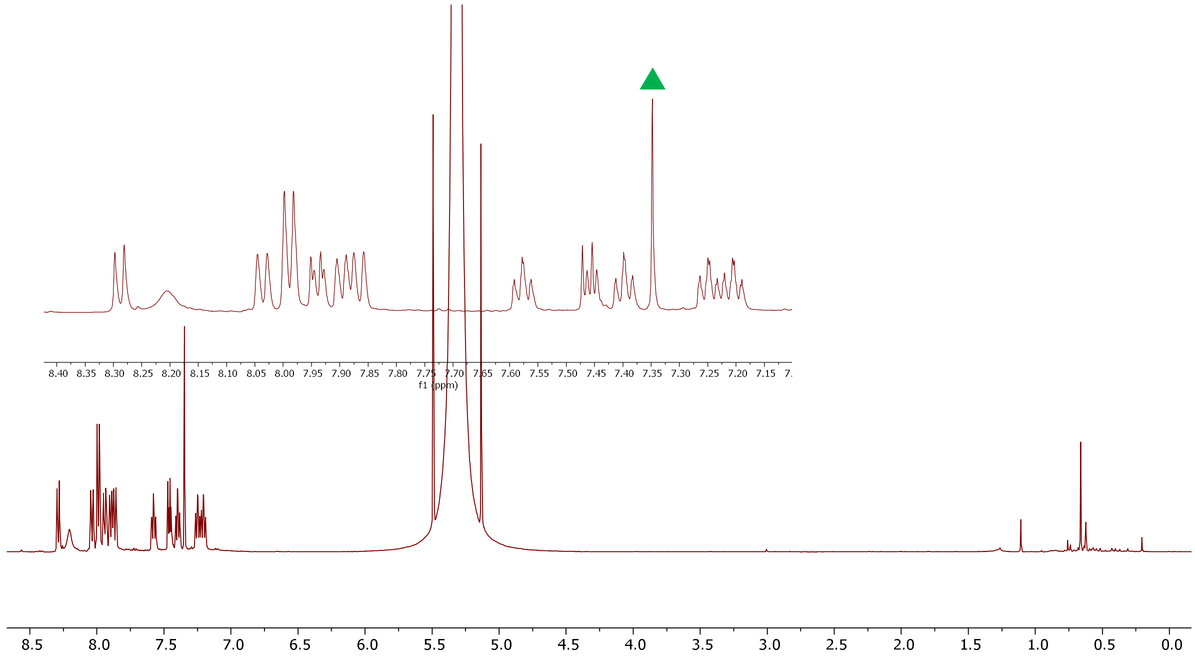

Figure S10. ^{1}H NMR spectrum of **1-Br** (crude sample) prepared via route (i). The green triangle denotes the residual benzene solvent.

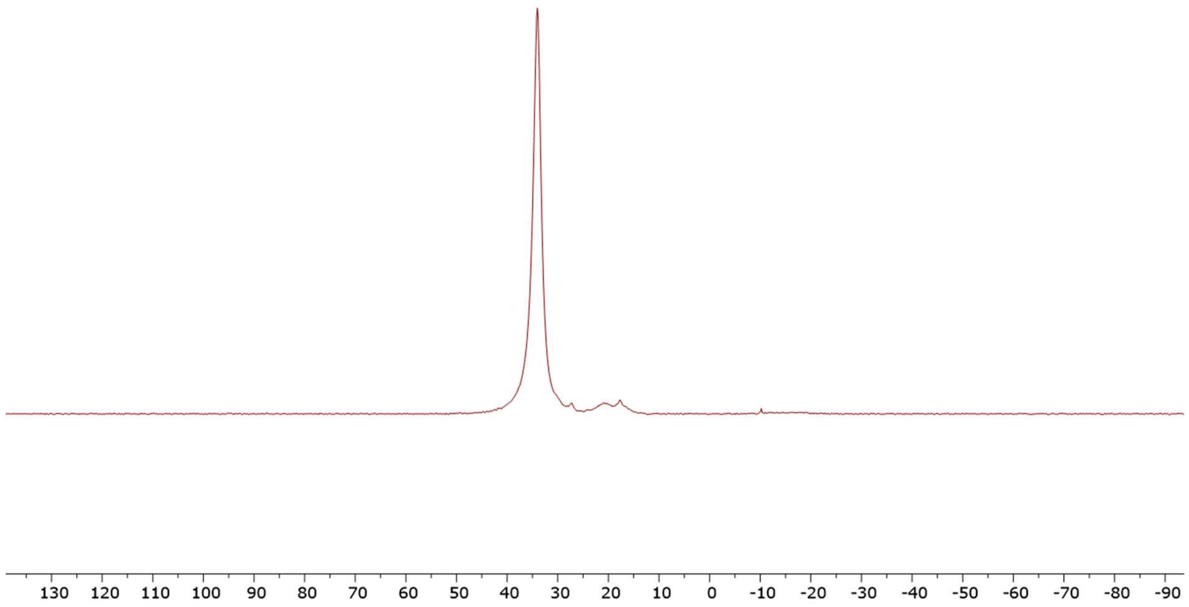

Figure S11. ^{11}B NMR spectrum of **1-Br** (crude sample) prepared via route (i).

(b) Synthesis of 1-Mes with PhTMS and $HNTf_2$ (route(ii))

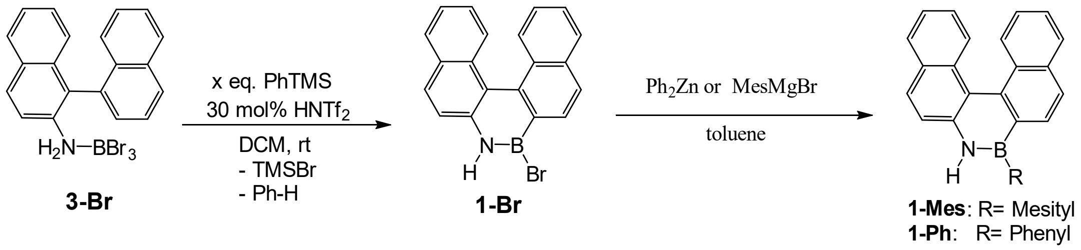

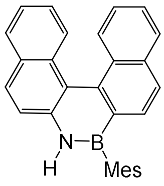

1-Mes

To a solution of **3-Br** (104 mg, 0.2 mmol) in dichloromethane (1.5 mL) was added PhTMS (34 µL, 0.2 mmol) and $HNTf_2$ (18 mg, 0.06 mmol). The sample was kept at room temperature and the reaction progress was monitored by NMR spectroscopy over time (See Figure S12-13 for in-situ NMR spectra). In order to accelerate the reaction, 10 µL PhTMS was added to the mixture 24 hours after the first addition. This was repeated until **3-Br** was fully consumed. The NMR reaction mixture containing predominantly **1-Br** was transferred to an ampoule. Both dichloromethane and the TMSBr byproduct were removed under vacuum. The obtained solid was dissolved in toluene. To the solution was added 2-mesitylmagnesium bromide (MesMgBr) (0.3 mL, 1 M in

Et_2O) at -78 °C. The solution was allowed to gradually warm to room temperature and stirred overnight. The reaction was then quenched with HCl aqueous solution (3 mL, 1M). The mixture was extracted with Et_2O and the organic layer was dried over Na_2SO_4 and concentrated in *vacuo*. The crude product was purified by column chromatography on silica gel (hexane: DCM 100:1 to 4:1). The desired compound was obtained as a light-yellow solid (60 mg, 76%). The NMR data are in agreement with that obtained from the open system procedure (S3.3.1a).

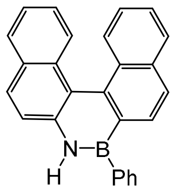

1-Ph

Synthesis of **1-Ph** from **1-Br**: To a toluene solution (3 mL) containing crude **1-Br** (0.1 mmol, generated as above) was added Ph_2Zn (2 mL, 0.2 M in toluene) at 0 °C. The reaction was allowed to gradually warm to room temperature and stirred overnight. The reaction was then quenched with 1 M HCl aqueous solution and the mixture was extracted with Et_2O. The organic layer was dried over Na_2SO_4 and concentrated in *vacuo*. Purification of the crude product was attempted by column chromatography on silica gel (best conditions = hexane: DCM 100:1 to 4:1). The desired compound **1-Ph** was obtained as an oil (12 mg, 34%). However, due to the limited stability of **1-Ph**, it is very difficult to get clean NMR spectra of **1-Ph**. Thus only ^1H and ^{11}B NMR data are reported here.

^{1}**H** NMR (500 MHz, $CDCl_3$) δ = 8.26 (d, J = 8.2 Hz, 1H), 8.18 – 8.12 (m, 1H), 8.01 – 7.97 (m, 2H), 7.95 – 7.90 (m, 4H), 7.86 – 7.85 (m, 2H), 7.58 – 7.52 (m, 3H), 7.50 (d, J = 8.6 Hz, 1H), 7.41-7.38 (m, 1H), 7.29-7.23 (m, 3H).

^{11}**B** NMR (160 MHz, $CDCl_3$) δ = 36.77.

HRMS (ESI): Calcd for $C_{26}H_{19}NB^+$ $[M+H]^+$: 356.1605, found: 356.1572.

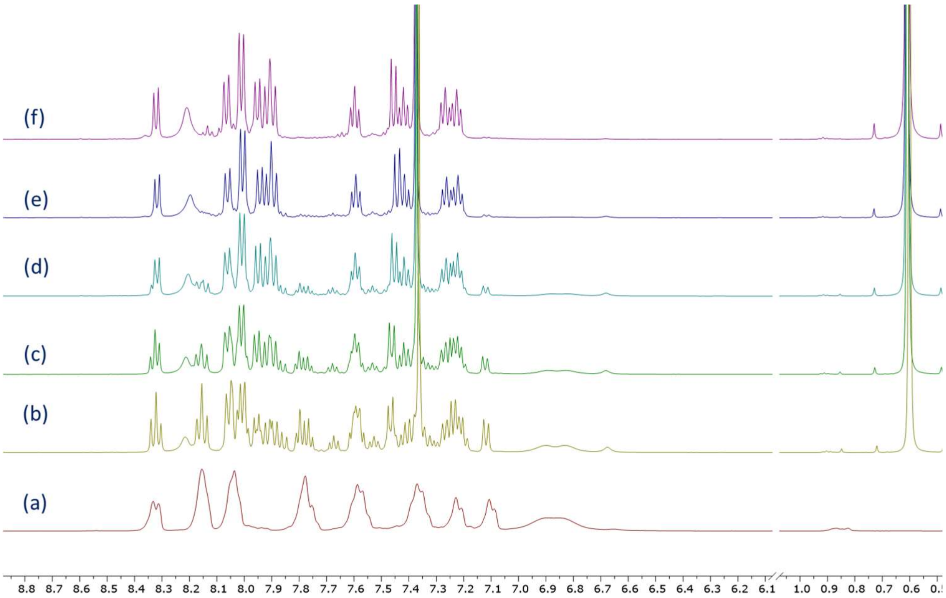

Figure S12. In-situ ^{1}H NMR spectra for the dehydrobromination and borylation reactions of **3-Br** with 30 mol% $HNTf_2$ in dichloromethane: (a) **3-Br**; (b) 24 hours after the addition of 1 eq. of PhTMS and 30 mol% $HNTf_2$; (c) = (b) + 0.28 eq. PhTMS, 24 hours; (d)= (c) + 0.28 eq. PhTMS, 24 hours; (e) = (d) + 0.28 eq. PhTMS, 24 hours; (f) = (e) + 0.14 eq. PhTMS, 24 hours.

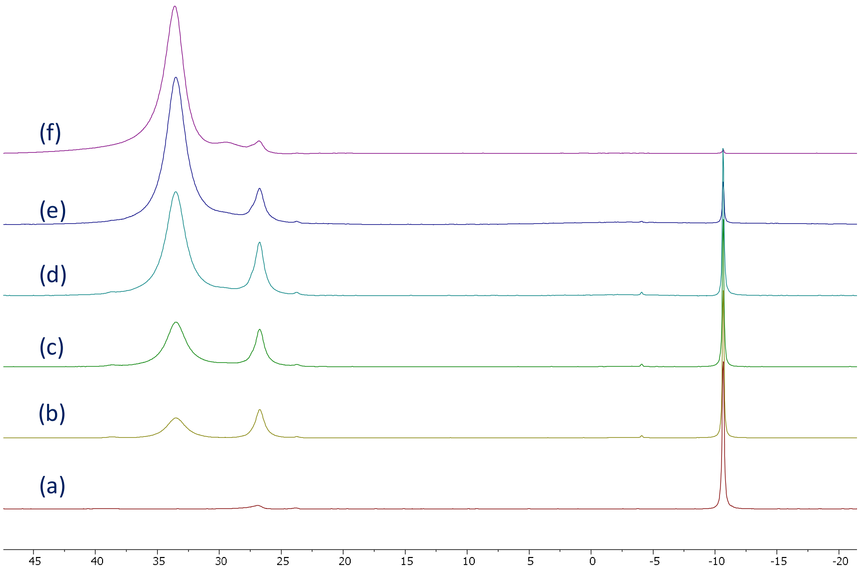

Figure S13. In-situ ^{11}B NMR spectra for the dehydrobromination and borylation reactions of **3-Br** with 30 mol% $HNTf_2$ in dichloromethane: (a) **3-Br**; (b) 24 hour after the addition of 1 eq. of PhTMS and 30 mol% $HNTf_2$; (c) = (b) + 0.28 eq. PhTMS, 24 hours; (d) = (c) + 0.28 eq. PhTMS, 24 hours; (e) = (d) + 0.28 eq. PhTMS, 24 hours; (f) = (e) + 0.14 eq. PhTMS, 24 hours.

S3.3.2 Synthesis of 6-Mes

(a) Synthesis of 6-Mes via the open system procedure (route(i))

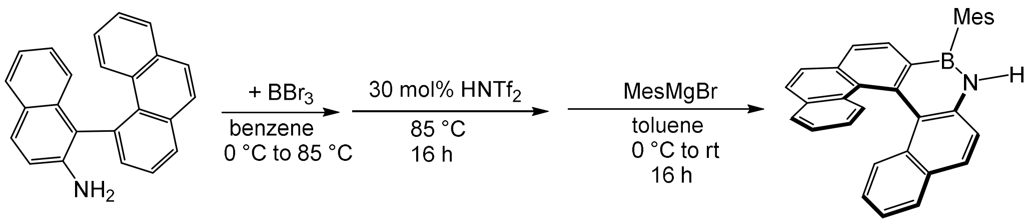

The preparation procedure of **6-Mes** via route (i) is the same as that of **1-Mes** (S3.3.1(a)) except that only 150 mg of the amine precursor (0.5 mmol) was used (and all other reagents were reduced accordingly). **6-Mes** was obtained as a white solid after workup (139 mg, 61%).

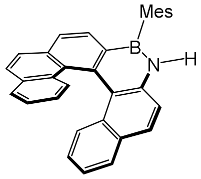

6-Mes

^{1}H NMR (500 MHz, $CDCl_3$) δ = 7.93 – 7.82 (m, 6H), 7.80 (d, *J* = 7.9 Hz, 1H), 7.77 (d, *J* = 8.0 Hz, 1H), 7.69 (d, *J* = 8.5 Hz, 1H), 7.50 (d, *J* = 8.6 Hz, 1H), 7.35 (d, *J* = 8.7 Hz, 1H), 7.22 (t, *J* = 7.6 Hz, 1H), 7.12 (t, *J* = 7.4 Hz, 1H), 7.01 (s, 1H), 6.98 (s, 1H), 6.75 – 6.66 (m, 2H), 2.42 (s, 3H), 2.26 (s, 3H), 2.12 (s, 3H).

^{11}B NMR (160 MHz, $CDCl_3$) δ = 39.05.

^{13}C{^{1}H} NMR (126 MHz, $CDCl_3$) δ = 140.7, 140.6, 137.8, 136.9, 134.9, 134.4, 132.0, 131.7, 130.8, 130.6, 129.5, 129.4, 128.6, 128.0, 127.7, 127.7, 127.5, 127.3, 127.2, 126.7, 126.6, 126.5, 125.6, 125.2, 124.5, 123.4, 119.8, 118.7, 23.1, 23.0, 21.3.

HRMS (ESI): Calcd for $C_{33}H_{27}NB^+$ $[M+H]^+$: 448.2231, found: 448.2240.

(b) Synthesis of 6-Mes with PhTMS and $HNTf_2$ (route(ii))

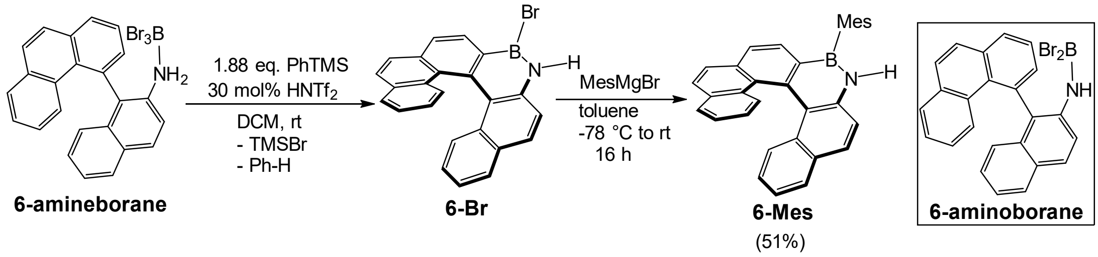

The preparation procedure of **6-Mes** via route (ii) is similar to that of **1-Mes** (S3.3.1(b)). Compound **6-Br** (0.16 mmol) was generated in-situ with a total of 1.88 eq. PhTMS added in multiple portions (NMR yield: 82%) (See Figure S14-15 for in-situ NMR studies). The formation of **6-Br** was confirmed by converting it into **6-Mes** (isolated yield: 55%). The NMR data are in agreement with that obtained from the open system procedure.

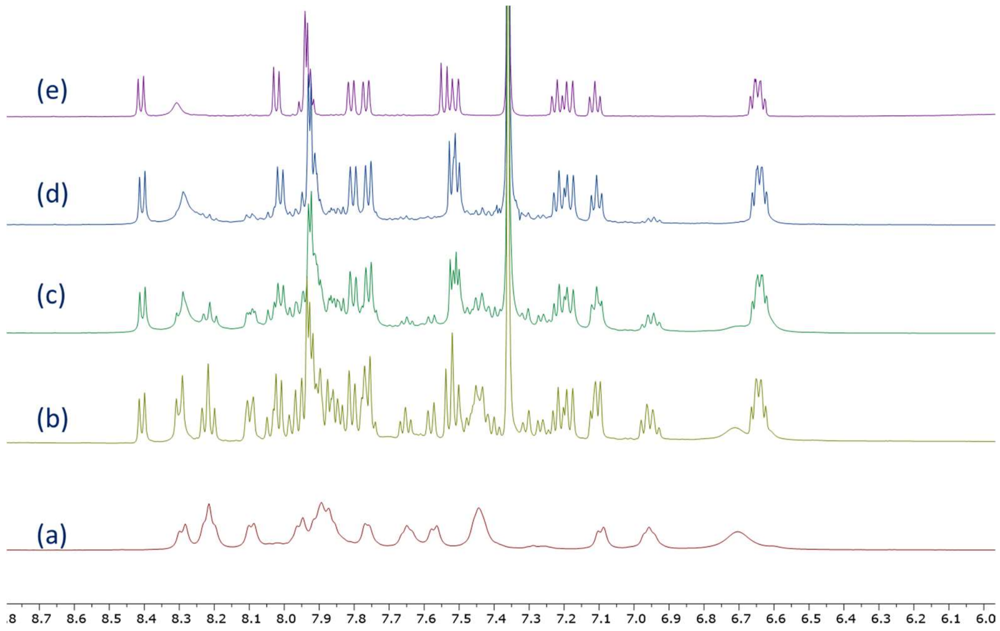

Figure S14. In-situ ^{1}H NMR spectra for the dehydrobromination and borylation of **6-amineborane** in dichloromethane: (a) **6-amineborane**; (b) 96 hours after the addition of 1 eq. of PhTMS and 30 mol% $HNTf_2$; (c) = (b) + 0.5 eq. PhTMS, 24 hours; (d) = (c) + 0.25 eq. PhTMS, 72 hours; (e) = (b) + 0.125 eq. PhTMS, 24 hours. (During the reaction, some **6-Br** precipitated out of the solution, more DCM was added at this stage to obtain a homogeneous solution).

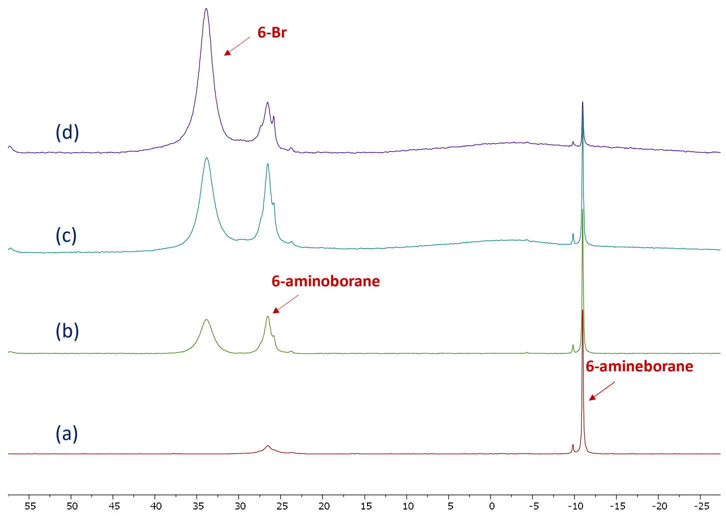

Figure S15. In-situ ^{11}B NMR spectra for the dehydrobromination and borylation reactions of **6-amineborane** in dichloromethane: (a) **6-amineborane**; (b) 96 hours after the addition of 1 eq. of PhTMS and 30 mol% $HNTf_2$; (c) = (b) + 0.5 eq. PhTMS, 24 hours; (d) = (c) + 0.25 eq. PhTMS, 72 hours; (e) = (d) + 0.125 eq. PhTMS, 24 hours. ^{11}B NMR spectrum after the last addition was not collected.

S3.3.3 Synthesis of 7-Mes

(a) Synthesis of 7-Mes via the open system procedure (route(i))

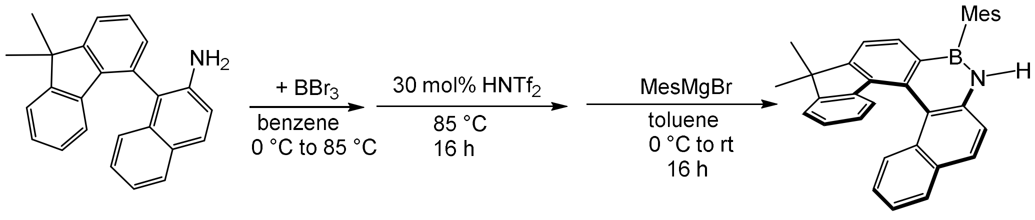

7-Mes

The preparation procedure of **7-Mes** via route (i) is the same as that of **1-Mes** (S3.3.1(a)) except that only 335 mg of the amine precursor (1 mmol) was used (and all other reagents were reduced accordingly). **7-Mes** was obtained as a white solid after workup (310 mg, 67%).

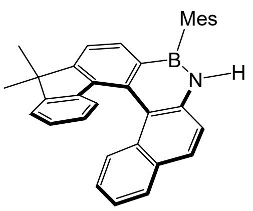

7-Mes

^{1}H NMR (500 MHz, $CDCl_3$) δ = ^{1}H NMR (500 MHz, $CDCl_3$) δ = 8.00 – 7.97 (dd, *J* = 8.7, 4.6 Hz, 3H); 7.84 (d, *J* = 7.5 Hz, 1H), 7.73 (s, 1H), 7.63 (d, *J* = 7.5 Hz, 1H), 7.50 – 7.47 (m, 2H), 7.43 – 7.35 (m, 1H), 7.23 – 7.13 (m, 2H), 7.00 (s, 2H), 6.77 (t, *J* = 7.1 Hz, 1H), 6.38 (d, *J* = 7.9 Hz, 1H), 2.43 (s, 3H), 2.24 (s, 3H), 2.15 (s, 3H), 1.83 (s, 3H), 1.58 (s, 3H).

^{11}B NMR (160 MHz, $CDCl_3$) δ= 40.30.

^{13}C{^{1}H} NMR (126 MHz, $CDCl_3$) δ = 158.1, 153.2, 140.6, 140.6, 140.0, 137.9, 137.7, 136.1, 134.6, 133.6, 131.1, 129.3, 129.2, 128.4, 127.9, 127.2, 127.1, 126.2, 126.2, 125.2, 125.1, 123.7, 121.6, 120.4, 119.6, 117.1, 46.7, 28.8, 26.4, 23.0, 23.0, 21.3.

HRMS (ESI): Calcd for $C_{34}H_{31}NB^+$ $[M+H]^+$: 464.2544, found: 464.2526.

(b) Synthesis of 7-Mes with PhTMS and $HNTf_2$ (route(ii))

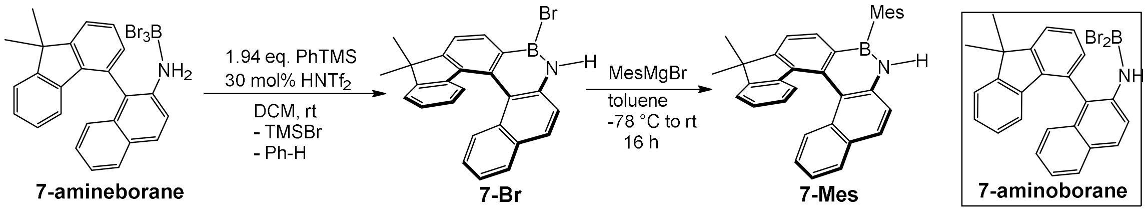

The preparation procedure of **7-Mes** via route (ii) is similar to that of **1-Mes** (S3.3.1(b)). Compound **7-Br** (0.16 mmol) was generated in-situ with 1.94 eq. PhTMS (NMR yield: 67%) (See Figure S16-17 for in-situ NMR studies). The formation of **7-Br** was confirmed by converting it into **7-Mes** (isolated yield 47%). The NMR data are in agreement with that obtained from the open system procedure.

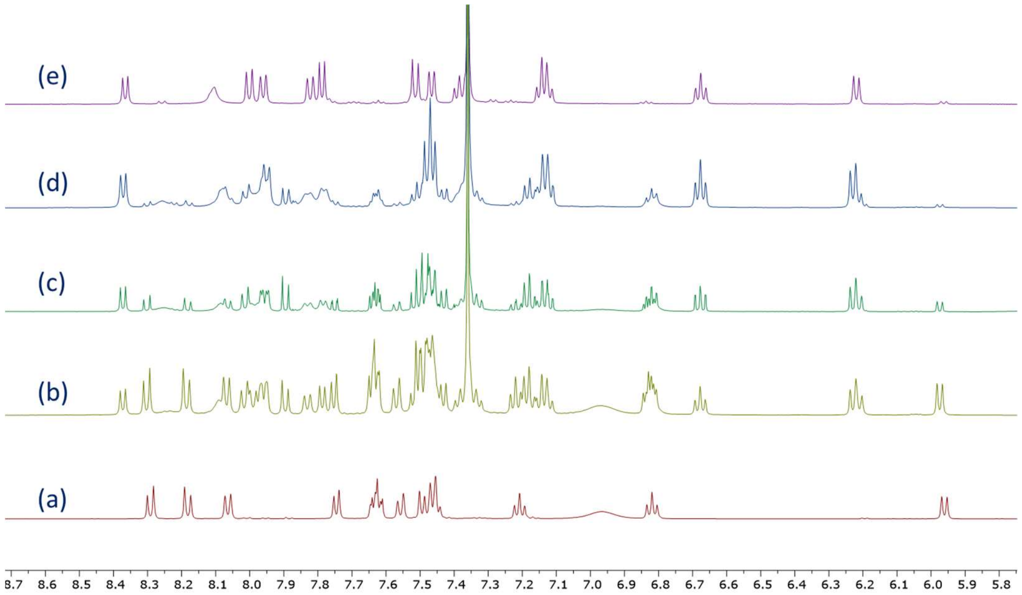

Figure S16. In-situ ^{1}H NMR spectra for the dehydrobromination and borylation of **7-amineborane** in dichloromethane: (a) **7-amineborane**; (b) 1 eq. PhTMS, 30 mol% $HNTf_2$, 72 hours; (c) = (b) + 0.35 eq. PhTMS, rt, 24h; (d) = (c) + 0.35 eq. PhTMS, rt, 24h; (e) = (d) + 0.25 eq. PhTMS, rt, 48h. During the reaction, some **7-Br** precipitated out of the solution, more DCM was added to obtain a homogeneous solution.

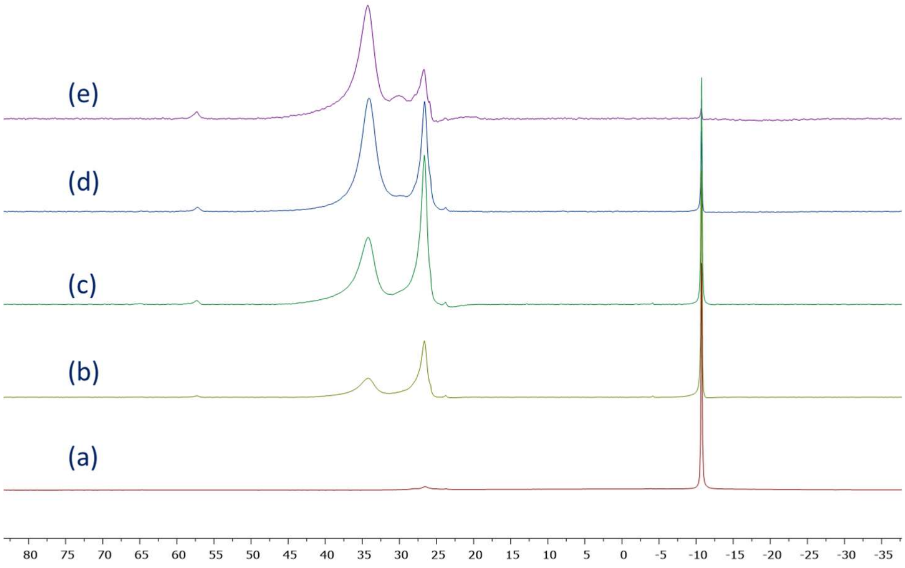

Figure S17. In-situ ^{1}H NMR spectra for the dehydrobromination and borylation of **7-amineborane** in dichloromethane: (a) **7-amineborane**; (b) 1 eq. PhTMS, 30 mol% $HNTf_2$, 72 hours; (c) = (b) + 0.35 eq. PhTMS, 24h; (d) = (c) + 0.35 eq. PhTMS, 24h; (e) = (d) + 0.25 eq. PhTMS, 48h.

(c) Attempted synthesis of 7-Br with excess BBr_3

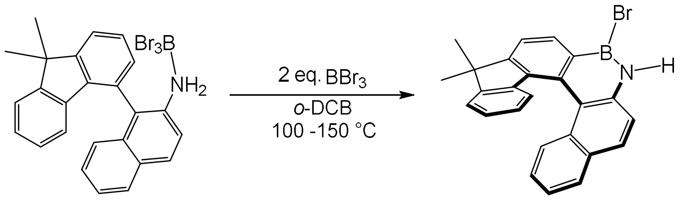

To a J-Young NMR tube charged with **7-amineborane** (29 mg, 0.05 mmol) in 1 mL *o*DCB was added BBr_3 (0.5 mL, 0.2 M in *o*DCB). A homogeneous solution was obtained, and the sample was kept at room temperature for 48 hours. NMR studies indicate no **7-Br** formed at all. The sample was heated to 100 °C for 24 hours, only a trace amount of **7-Br** formed (< 5%) based on the ^{1}H NMR spectrum. The sample was then heated to 150 °C for 24 hours and roughly 25 % **7-Br** (**7-amineborane** : **16-Br** = 1: 3) formed. Further heating at 150 °C for another 48 hours (72 hours in total at 150 °C) didn't change the ratio of **7-amineborane** to **7-Br**.At same time, undesired by-products were observed.

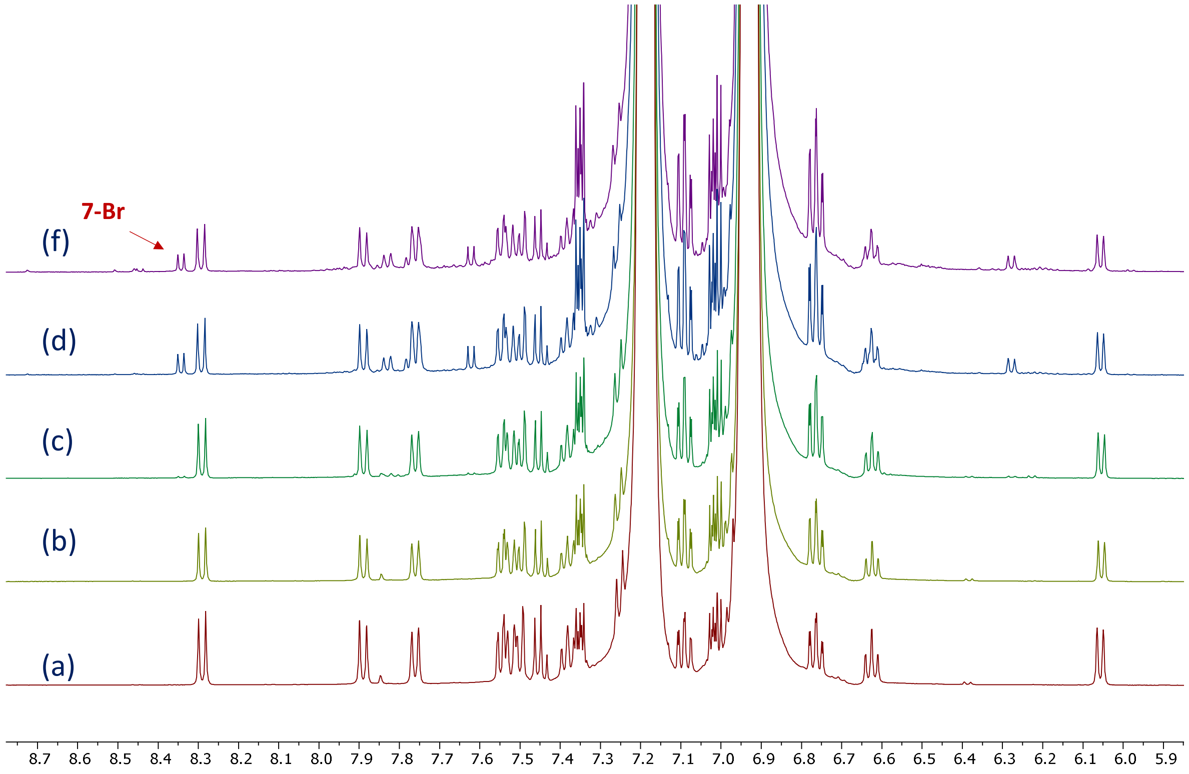

Figure S18. In-situ ^{1}H NMR spectra for the borylation reaction of **7-amineborane** with excess BBr_3 in *o*DCB: (a) the mixture of **7-amineborane** and 2 eq. BBr_3; (b) rt, 48 hours; (c) 100 °C, 24 hours; (d) 150 °C, 24 hours; (e) 150 °C, 72 hours.

S3.3.4 Derivatization of 1-Mes

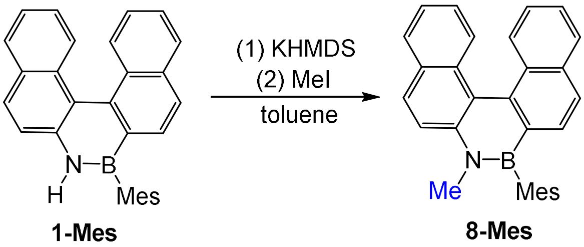

To a J-Young NMR tube charged with **1-Mes** (40 mg, 0.1 mmol) and potassium bis(trimethylsilyl)amide (KHMDS) (30 mg, 0.15 mmol) was added 2 mL toluene. The resulting yellow suspension was stirred at room temperature for 1 hour. Then MeI (12 µL, 0.2 mmol) was added and the suspension turned yellow. The reaction was stirred overnight and quenched with 1 M HCl aqueous solution. The product was extracted with ethyl acetate and the organic layer was dried over Na_2SO_4 and concentrated in *vacuo*. The crude product was purified by column chromatography on silica gel (hexane: DCM 100:1 to 5:1, Rf = 0.41 in hexane: DCM 5:1). The desired product was obtained as a light-yellow solid (17 mg, 41%).

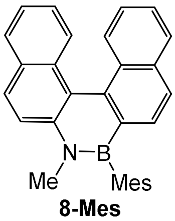

^{1}H NMR (500 MHz, $CDCl_3$) δ = 8.06 – 8.03 (m, 2H), 7.98 (d, *J* = 8.7 Hz, 1H), 7.78 (d, *J* = 8.1 Hz, 1H), 7.96 – 7.92 (m, 3H), 7.59 (d, *J* = 8.1 Hz, 1H), 7.52 (t, *J* = 7.4 Hz, 1H), 7.52 (t, *J* = 7.4 Hz, 1H), 7.24 – 7.20 (m, 2H), 6.97 (s, 1H), 6.96 (s, 1H), 3.64 (s, 3H), 2.41 (s, 3H), 2.12 (s, 3H), 1.95 (s, 3H).

^{11}B NMR (160 MHz, $CDCl_3$) δ = 40.56.

^{13}C{^{1}H} NMR (126 MHz, $CDCl_3$) δ = 140.5, 140.1, 139.7, 137.40, 137.35, 135.1, 132.1, 130.9, 130.5, 129.7, 129.2, 129.0, 128.8, 128.1, 127.7, 127.32, 127.28, 126.7, 126.6, 124.8, 124.2, 124.1, 119.0, 115.7, 36.6, 22.7, 22.6, 21.5.

HRMS (ESI): Calcd for $C_{30}H_{27}NB^+$ $[M+H]^+$: 412.2231, found: 412.2217.

S4. Brief substrates scope assessment

General procedure 3:

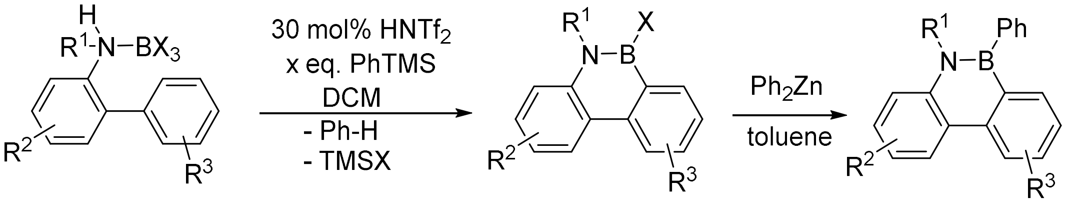

To a solution of an amine-borane adduct **ArN(R)H-BX$_3$** (R = H, Me; X = Cl, Br) (0.1-0.2 mmol, 1 eq.) in dichloromethane (1-2 mL) was added PhTMS (1 eq.) and HNTf$_2$ (30 mol%). The sample was kept at room temperature and the reaction progress was monitored by the NMR spectroscopy over time. At first, all PhTMS was consumed to produce an aminoborane **Ar(R)N-BX$_2$**, which in the presence of HNTf$_2$ was converted into an azaborine and an amine-borane adduct **ArN(R)H-BX$_3$** in a ca. 1:1 ratio. More PhTMS was added once the amino-borane was fully consumed, which can be easily determined by ^{11}B NMR spectroscopy. This process was repeated until all the amine-borane adduct was converted into the desired B-X azaborine product (X = Cl, Br). The NMR yield was determined by adding cyclohexane as an internal standard. For some substrates, the product precipitates out of the solution when the reactions complete. More solvent was added to get a homogeneous solution before adding the internal standard. Once the "NMR yield" was determined, the reaction mixture was transferred to an ampoule and the solvent was removed under vacuum. The obtained solid was dissolved in toluene. To the solution was added Ph$_2$Zn (x eq., 0.2 M in toluene) at 0 °C. The solution was allowed to gradually warm to room temperature and was stirred overnight. The reaction was then quenched with 1 M HCl aqueous solution. The product was extracted with Et$_2$O. The organic layer was dried over Na$_2$SO$_4$ and concentrated in *vacuo*. The crude product was purified by column chromatography on silica gel (hexane: DCM 100:1 to 4:1).

Synthesis of 9-Ph

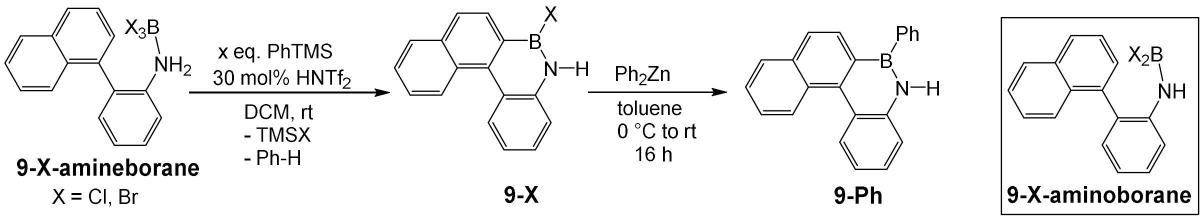

Compound **9-X** was generated in-situ via the **general procedure 3** (NMR yield: **9-Cl** 67%, **9-Br** 87%) (See Figure S19-22 for in-situ NMR studies). The formation of **9-X** was confirmed by converting it into **9-Ph** via **general procedure 4** (isolated yield: from **9-Cl** 36%, from **9-Br** 68%).

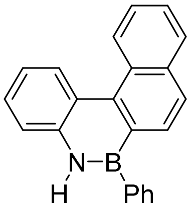

9-Ph

^{1}H NMR (500 MHz, $CDCl_3$) δ = 9.03 – 8.94 (m, 1H), 8.74 (dd, *J* = 8.3, 1.2 Hz, 1H), 8.17 (d, *J* = 8.2 Hz, 1H), 8.05 – 7.98 (m, 1H), 7.89 (s, 1H), 7.88 – 7.85 (m, 1H), 7.82 (m, 2H), 7.67 -7.61 (m, 2H), 7.57 – 7.47 (m, 4H), 7.42 (dd, *J* = 8.0, 1.3 Hz, 1H), 7.33 (ddd, *J* = 8.3, 7.0, 1.4 Hz, 1H).

^{11}B NMR (160 MHz, $CDCl_3$) δ = 36.95.

^{13}C{^{1}H} NMR (126 MHz, $CDCl_3$) δ = 139.9, 139.2, 136.2, 133.5, 131.2, 130.4, 129.8, 128.8, 128.6, 128.3, 128.2, 127.9, 127.0, 126.5, 125.7, 123.7, 121.0, 119.0.

HRMS (ESI): Calcd for $C_{22}H_{17}NB^+$ $[M+H]^+$: 306.1449, found: 306.1458.

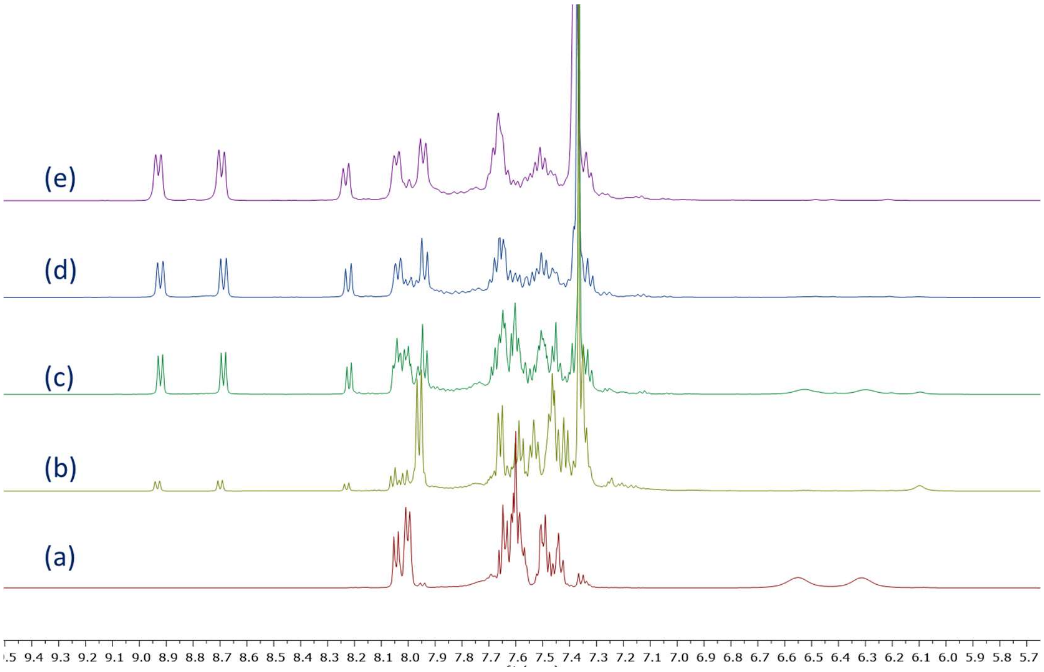

Figure S19. In-situ ^{1}H NMR spectra for the dehydrochlorination and borylation of **9-Cl-amineborane** in dichloromethane: (a) **9-Cl-amineborane**; (b) 1 eq. PhTMS, 30 mol% $HNTf_2$, 6 hours; (c) 192 hours; (d) = (c) + 0.5 eq. PhTMS, 72 h; (e) = (d) + 0.25 eq. PhTMS, 14 days.

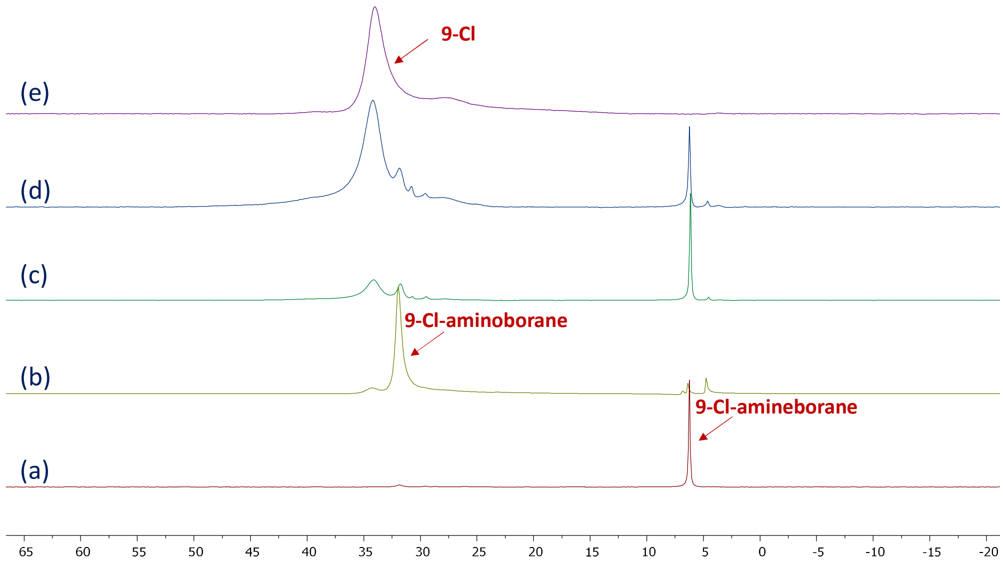

Figure S20. In-situ ^{11}B NMR spectra for the dehydrochlorination and borylation of **9-Cl-amineborane** in dichloromethane: (a) **9-Cl-amineborane**; (b) 1 eq. PhTMS, 30 mol% $HNTf_2$, 6 hours; (c) 192 hours; (d) = (c) + 0.5 eq. PhTMS, 72 h; (e) = (d) + 0.25 eq. PhTMS, 14 days.

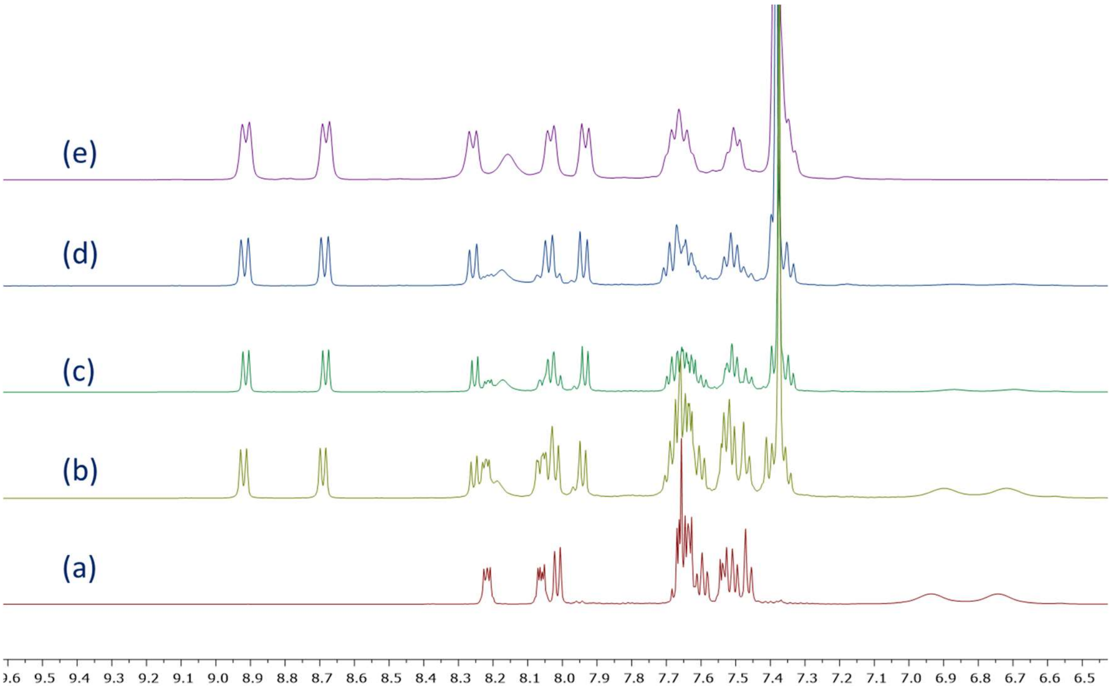

Figure S21. In-situ ^{1}H NMR spectra for the dehydrobromination and borylation of **9-Br-amineborane** dichloromethane: (a) **9-Br-amineborane**; (b) 1 eq. PhTMS, 30 mol% $HNTf_2$, 20 hours; (c) = (b) + 0.5 eq. PhTMS, 12 hours; (d) = (c) + 0.25 eq. PhTMS, 12 h; (e) = (d) + 0.25 eq. PhTMS, 12 hours.

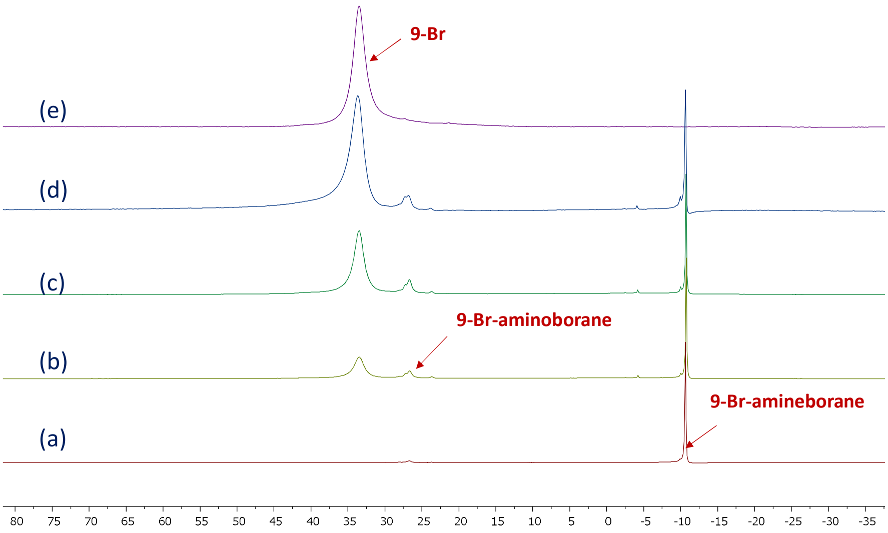

Figure S22. In-situ ^{11}B NMR spectra for the dehydrobromination and borylation of **9-Br-amineborane** dichloromethane: (a) **9-Br-amineborane**; (b) 1 eq. PhTMS, 30 mol% $HNTf_2$, 20 hours; (c) = (b) + 0.5 eq. PhTMS, rt, 12 hours; (d) = (c) + 0.25 eq. PhTMS, rt, 12 h; (e) = (d) + 0.25 eq. PhTMS, rt, 12 hours.

Synthesis of 10-Ph

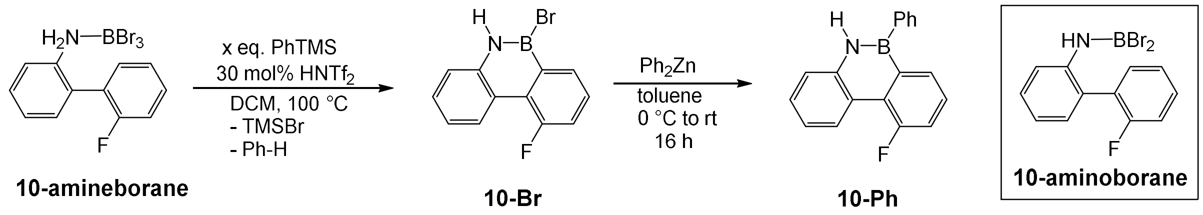

Compound **10-Br** was generated in-situ via a procedure similar to the **general procedure 3** except that the reaction was performed at 100 °C (heating block temperature). NMR yield: 67% (See Figure S23- 24 for in situ NMR studies). The formation of **10-Br** was confirmed by converting it into **10-Ph** (isolated yield: 49%).

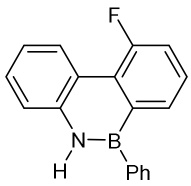

10-Ph

^{1}H{^{19}F} NMR ^{1}H NMR (400 MHz, $CDCl_3$) δ = 8.95 (d, J = 8.3 Hz, 1H), 8.05 (dd, J = 6.3, 2.5 Hz, 1H), 7.80 – 7.78 (m, 2H), 7.74 (s, 1H), 7.56 – 7.46 (m, 6H), 7.34 (ddd, J = 8.4, 7.2, 1.3 Hz, 1H), 7.29 (dd, J = 8.0, 1.2 Hz, 1H).

^{11}B NMR (160 MHz, $CDCl_3$) δ = 36.82.

^{19}F{^{1}H} NMR (376 MHz, $CDCl_3$) δ = -109.25.

^{13}C{^{1}H} NMR (126 MHz, $CDCl_3$) δ = 161.3 (d, $J_{C\text{-}F}$ = 254.7 Hz), 139.1, 133.3, 132.4 (d, $J_{C\text{-}F}$ = 3.4 Hz), 129.3 (d, J = 25.9 Hz), 129.0, 128.5 (d, J = 2.3 Hz), 128.2, 127.3 (d, $J_{C\text{-}F}$ = 4.6 Hz), 127.1 (d, $J_{C\text{-}F}$ = 8.8 Hz), 122.2 (d, $J_{C\text{-}F}$ = 2.7 Hz), 121.4 (d, $J_{C\text{-}F}$ = 5.7 Hz), 119.1, 118.8 (d, $J_{C\text{-}F}$ = 25.4 Hz).

HRMS (ESI): Calcd for $C_{18}H_{14}NBF^{+}$ $[M+H]^{+}$: 274.1198, found: 274.1213.

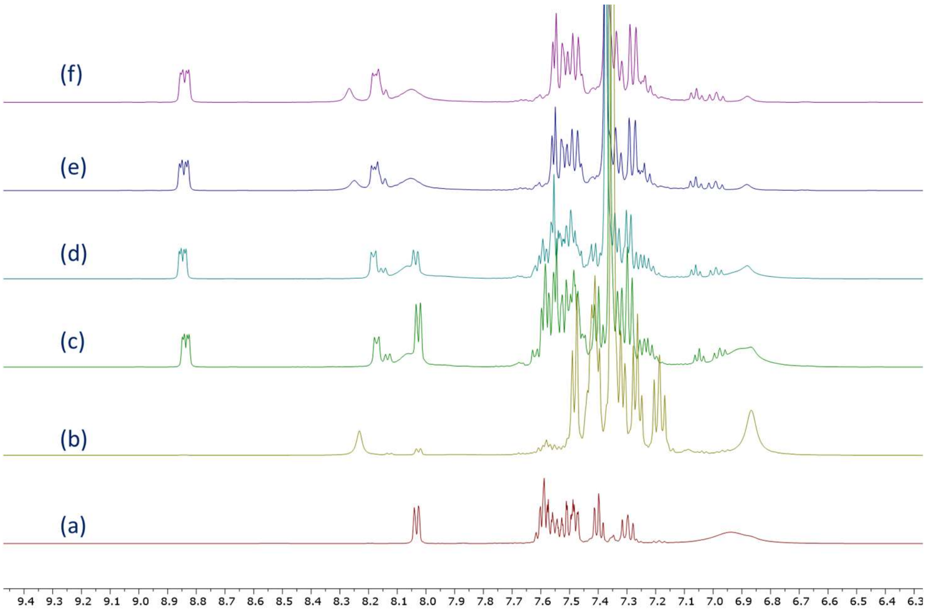

Figure S23. In-situ ^{1}H NMR spectra for the dehydrobromination and borylation of **10-amineborane** in dichloromethane: (a) **10-amineborane**; (b) 12 hours after the addition of 1 eq. of PhTMS and 30 mol% $HNTf_2$, only dehydrobromination occurred at this stage; (c) heated at 100 °C for 24 hours; (d) = (c) + 0.4 eq. PhTMS, 100 °C, 16 hours; (e) = (d) + 0.25 eq. PhTMS, 100 °C, 20 hours; (f) = (e) + 0.25 eq. PhTMS, 100 °C, 16 hours;

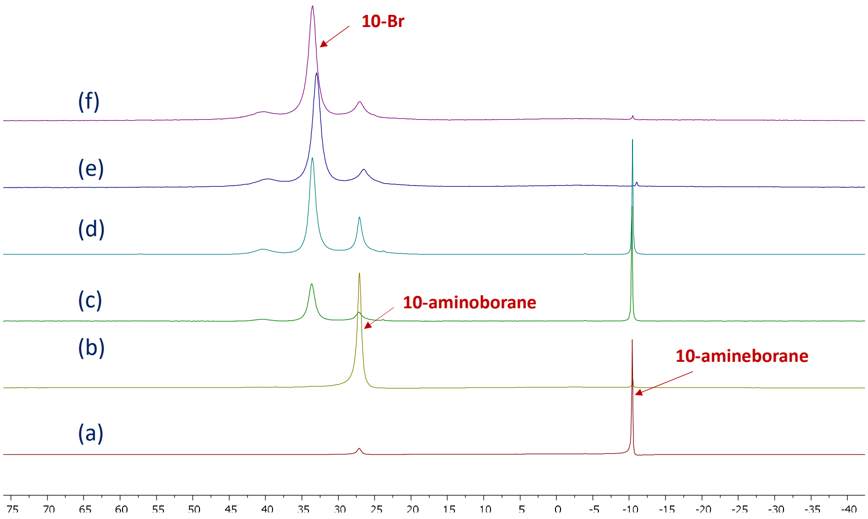

Figure S24. In-situ ^{1}H NMR spectra for the dehydrobromination and borylation of **10-amineborane** in dichloromethane: (a) **10-amineborane**; (b) 12 hours after the addition of 1 eq. of PhTMS and 30 mol% $HNTf_2$, (c) heated at 100 °C for 24 hours; (d) = (c) + 0.4 eq. PhTMS, 100 °C, 16 hours; (e) = (d) + 0.25 eq. PhTMS, 100 °C, 20 hours; (f) = (e) + 0.25 eq. PhTMS, 100 °C, 16 hours;

Synthesis of 11-Ph

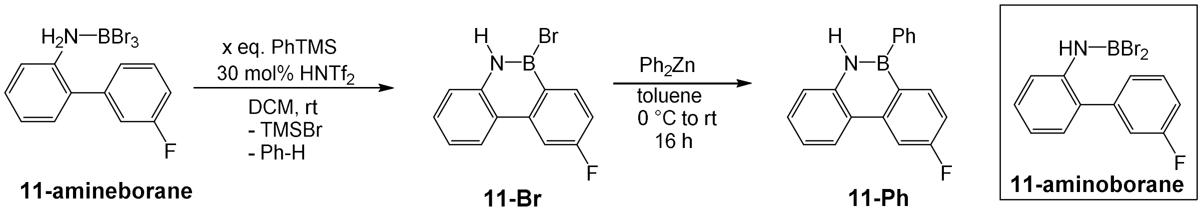

Compound **11-Br** was generated in-situ via the **general procedure 3** (NMR yield: 91%) (See Figure S25-26 for in-situ NMR studies). The formation of **11-Br** was confirmed by converting it into **11-Ph** (isolated yield: 65%).

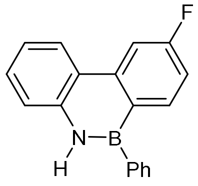

11-Ph

^{1}H{^{19}F} NMR ^{1}H NMR (400 MHz, $CDCl_3$): 8.34 (d, J = 8.1 Hz, 1H), 8.23 (d, J = 8.5 Hz, 1H), 8.13 (d, J = 2.3 Hz, 1H), 7.80 – 7.77 (m, 2H), 7.68 (s, 1H), 7.54 – 7.47 (m, 4H), 7.33 – 7.28 (m, 2H), 7.23 (dd, J = 8.5, 2.4 Hz, 1H).

^{11}B NMR (160 MHz, $CDCl_3$) δ = 36.81.

^{19}F{^{1}H} NMR (376 MHz, $CDCl_3$) δ = -108.07.

^{13}C{^{1}H} NMR (126 MHz, $CDCl_3$) δ = 165.2 (d, $J_{C\text{-}F}$ = 249.1 Hz), 142.0 (d, $J_{C\text{-}F}$ = 8.4 Hz), 139.1, 139.0 (d, $J_{C\text{-}F}$ = 8.9 Hz), 133.3, 129.0, 128.9, 128.3, 124.3, 122.9 (d, $J_{C\text{-}F}$ = 3.6 Hz), 122.0, 119.4, 114.4 (d, $J_{C\text{-}F}$ = 21.0 Hz), 108.4 (d, $J_{C\text{-}F}$ = 21.4 Hz).

HRMS (ESI): Calcd for $C_{18}H_{14}NBF^+$ $[M+H]^+$: 274.1198, found: 274.1200.

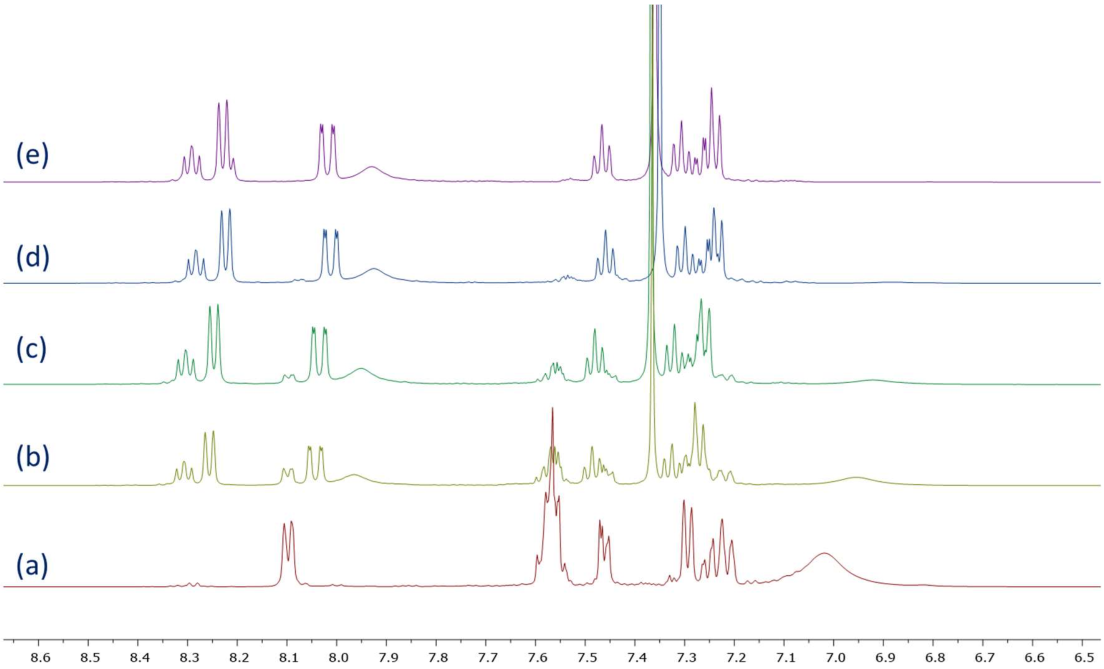

Figure S25. In-situ ^{1}H NMR spectra for the dehydrobromination and borylation of **11-amineborane** in dichloromethane: (a) **11-amineborane**; b) 1 eq. PhTMS, 30 mol% $HNTf_2$, 3 hours; (c) = (b) + 0.5 eq. PhTMS, 2 hours; (d) = (c) + 0.25 eq. PhTMS, 1 hour; (e) = (d) + 0.25 eq. PhTMS, 1 hour.

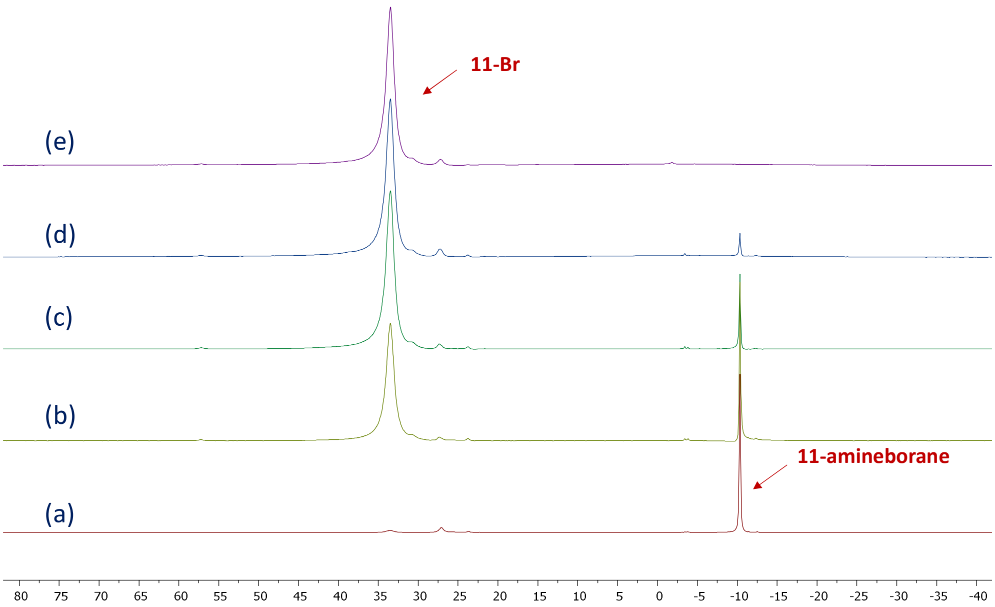

Figure S26. In-situ ^{11}B NMR spectra for the dehydrobromination and borylation of **11-amineborane** in dichloromethane: (a) **11-amineborane**; b) 1 eq. PhTMS, 30 mol% $HNTf_2$, 3 hours; (c) = (b) + 0.5 eq. PhTMS, 2 hours; (d) = (c) + 0.25 eq. PhTMS, 1 hour; (e) = (d) + 0.25 eq. PhTMS, 1 hour.

Synthesis of 12-Ph

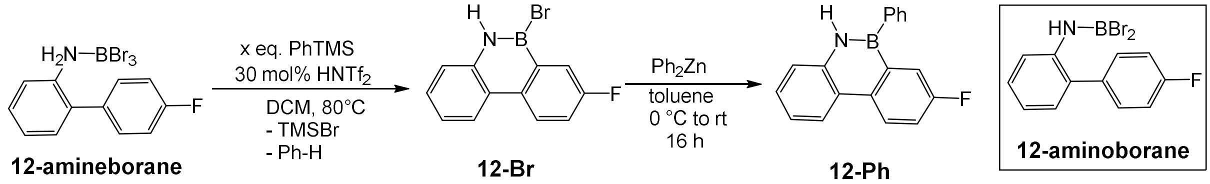

Compound **12-Br** was generated in-situ via a procedure similar to the **general procedure 3** except that the reaction was performed at 80 °C (heating block temperature). NMR yield: 78% (See Figure S27-28 for in-situ NMR studies). The formation of **12-Br** was confirmed by converting it to **12-Ph** (isolated yield: 35%).

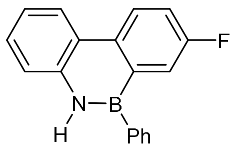

12-Ph

^{1}**H** NMR (400 MHz, $CDCl_3$) δ = 8.50 (d, J = 9.0 Hz, 1H), 8.42 – 8.35 (m, 1H), 7.87 (d, J = 2.9 Hz, 1H), 7.82 – 7.77 (m, 2H), 7.75 (s, 1H), 7.57 – 7.51 (m, 3H), 7.51 – 7.42 (m, 2H), 7.38 – 7.27 (m, 2H).

^{11}**B** NMR (128 MHz, $CDCl_3$) δ = 36.85.

^{19}**F{^{1}H}** NMR (376 MHz, $CDCl_3$) δ = -115.71.

^{13}**C{^{1}H}** NMR (126 MHz, $CDCl_3$) δ = 161.44 (d, J_{C-F} = 247.1 Hz), 138.36, 135.60 (d, J_{C-F} = 2.4 Hz), 133.23, 129.15, 128.37, 128.08, 124.82 (d, J_{C-F} = 7.5 Hz), 124.02, 123.18, 122.18, 120.92 (d, J_{C-F} = 18.4 Hz), 119.39, 119.20 (d, J_{C-F} = 22.9 Hz).

HRMS (ESI): Calcd for $C_{18}H_{13}NBFNa^{+}$ $[M+Na]^{+}$: 296.1032, found: 296.1027.

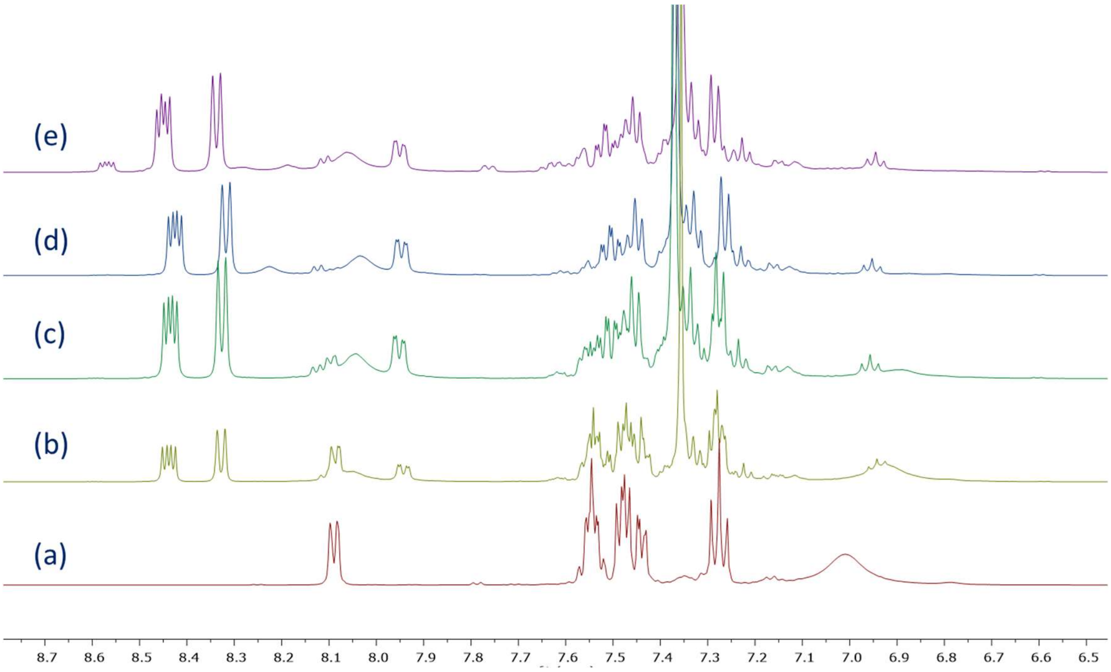

Figure S27. In-situ ^{1}H NMR spectra for the dehydrobromination and borylation of **12-amineborane** in DCM: (a) **12-amineborane**; (b) 1 eq. PhTMS, 30 mol% $HNTf_2$,80 °C, 6 hours; (c) = (b) + 0.5 eq. PhTMS, 80 °C, 6 hours; (d) = (c) + 0.25 eq. PhTMS, 80 °C, 5 hours; (e) = (d) + 0.25 eq. PhTMS, 80 °C, 4 hours.

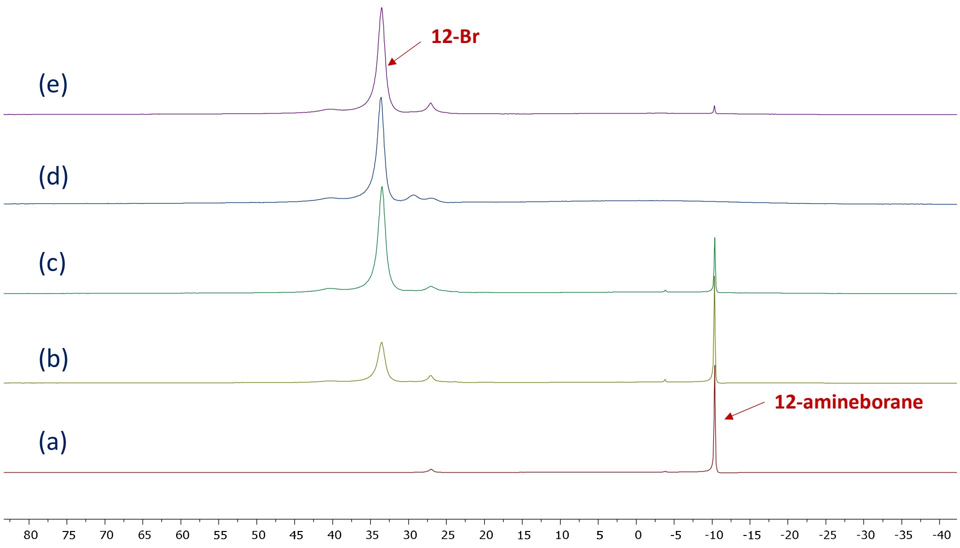

Figure S28. In-situ ^{11}B NMR spectra for the dehydrobromination and borylation of **12-amineborane** in dichloromethane: (a) **12-amineborane**; (b) 1 eq. PhTMS, 30 mol% $HNTf_2$, 80 °C, 6 hours; (c) = (b) + 0.5 eq. PhTMS, 80 °C, 6 hours; (d) = (c) + 0.25 eq. PhTMS, 80 °C, 5 hours; (e) = (d) + 0.25 eq. PhTMS, 80 °C, 4 hours.

Synthesis of 13-Ph

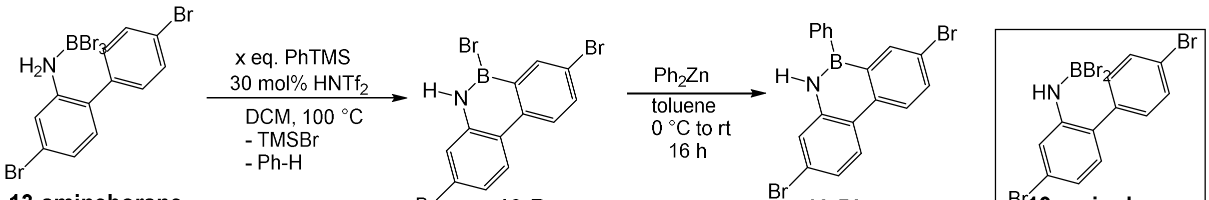

Compound **13-Br** was generated in-situ via a procedure similar to the **general procedure 3** except that the reaction was performed at 100 °C (heating block temperature) (See Figure S29-30 for in-situ NMR studies). The formation of **13-Br** was confirmed by converting it into **13-Ph** (isolated yield: 51%).

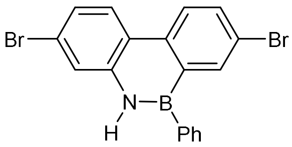

13-Ph

^{1}H NMR (500 MHz, $CDCl_3$) δ = 8.29 (d, *J* = 2.2 Hz, 1H), 8.25 (d, *J* = 8.8 Hz, 1H), 8.19 (d, *J* = 8.7 Hz, 1H), 7.83 (dd, *J* = 8.7, 2.3 Hz, 1H), 7.77 – 7.70 (m, 2H), 7.63 (s, 1H), 7.55 - 7.37 (m, 3H), 7.43 (d, *J* = 2.1 Hz, 1H), 7.38 (dd, *J* = 8.6, 2.0 Hz, 1H).

^{11}B NMR (160 MHz, $CDCl_3$) δ = 37.09.

^{13}C{^{1}H} NMR (126 MHz, $CDCl_3$) δ = 139.7, 138.7, 137.3, 134.4, 133.2, 129.4, 128.5, 125.5, 125.3, 124.3, 122.2, 122.00, 121.96, 121.4.

HRMS (ESI): Calcd for $C_{18}H_{12}NBBr_2Na^+$ $[M+Na]^+$: 433.9322, found: 433.9342.

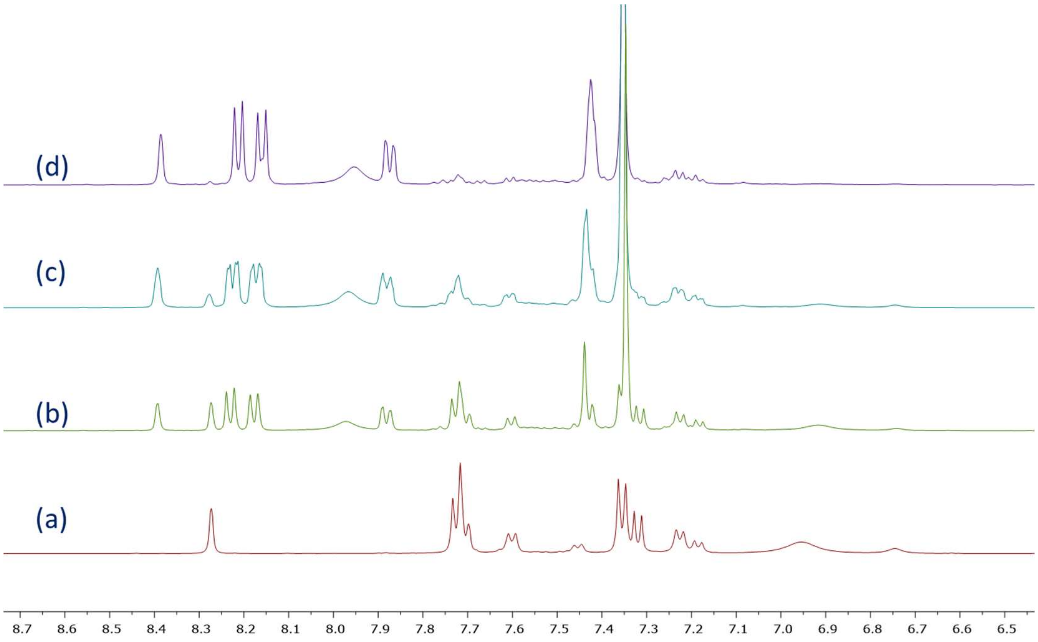

Figure S29. In-situ ^{1}H NMR spectra for the dehydrobromination and borylation of **13-amineborane** in dichloromethane: (a) **13-amineborane**; (b) 1 eq. PhTMS, 30 mol% $HNTf_2$, 100 °C, 35 hours; (c) = (b) + 0.5 eq. PhTMS, 100 °C, 22 hours; (d) = (c) + 0.25 eq. PhTMS, 100 °C, 22 hours.

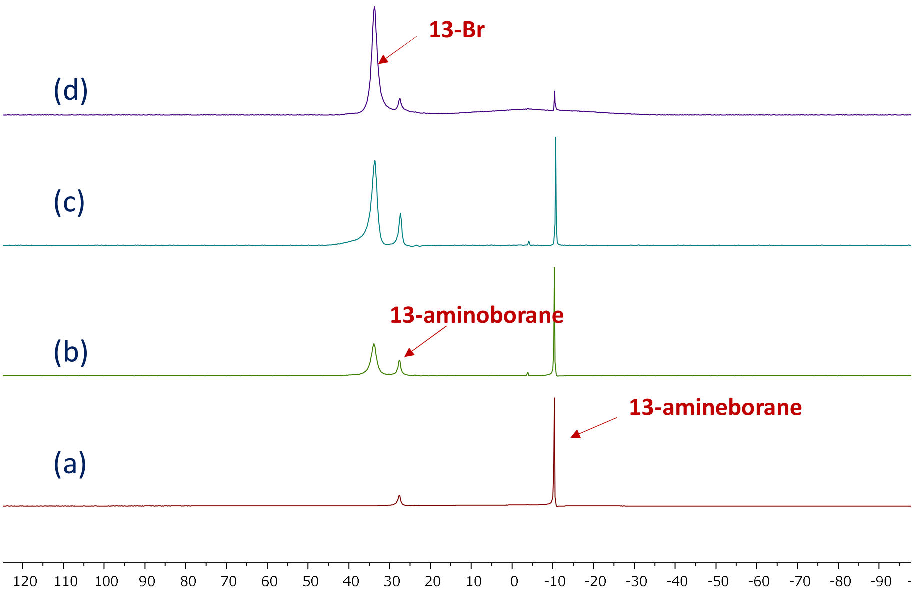

Figure S30. In-situ ^{11}B NMR spectra for the dehydrobromination and borylation of **13-amineborane** in dichloromethane: (a) **13-amineborane**; b) 1 eq. PhTMS, 30 mol% $HNTf_2$, 100 °C, 35 hours; (c) = (b) + 0.5 eq. PhTMS, 100 °C, 22 hours; (d) = (c) + 0.25 eq. PhTMS, 100 °C, 22 hours.

Synthesis of 14-Ph

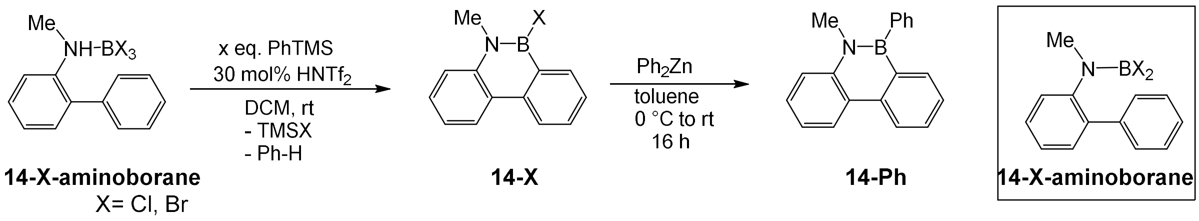

Compound **14-X** (X= Cl, Br) was generated in-situ via the **general procedure 3** (See Figure S31-34 for in-situ NMR studies). The formation of **14-X** was confirmed by converting it into **14-Ph** (isolated yield: from **14-Cl** 24%, from **9-Br** 64%).

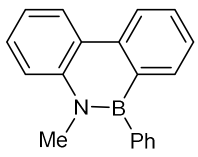

17-Ph

^{1}H NMR (500 MHz, $CDCl_3$) δ = 8.58 (d, *J* = 8.0 Hz, 1H), 8.51 (d, *J* = 8.1 Hz, 1H), 7.76 -7.71 (m, 2H), 7.67 (d, *J* = 8.4 Hz, 1H), 7.60 – 7.56 (m, 3H), 7.52 -7.37 (m, 5H), 3.62 (s, 3H).

^{11}B NMR (160 MHz, $CDCl_3$) δ = 39.22.

^{13}C{^{1}H} NMR (126 MHz, $CDCl_3$) δ = 141.5, 138.7, 137.2, 132.6, 131.0, 128.4, 127.8, 127.6, 126.1, 125.0, 124.3, 121.8, 121.7, 116.1, 37.7.

HRMS (ESI): Calcd for $C_{19}H_{17}NB^{+}$ $[M+Na]^{+}$: 270.1449, found: 270.1456.

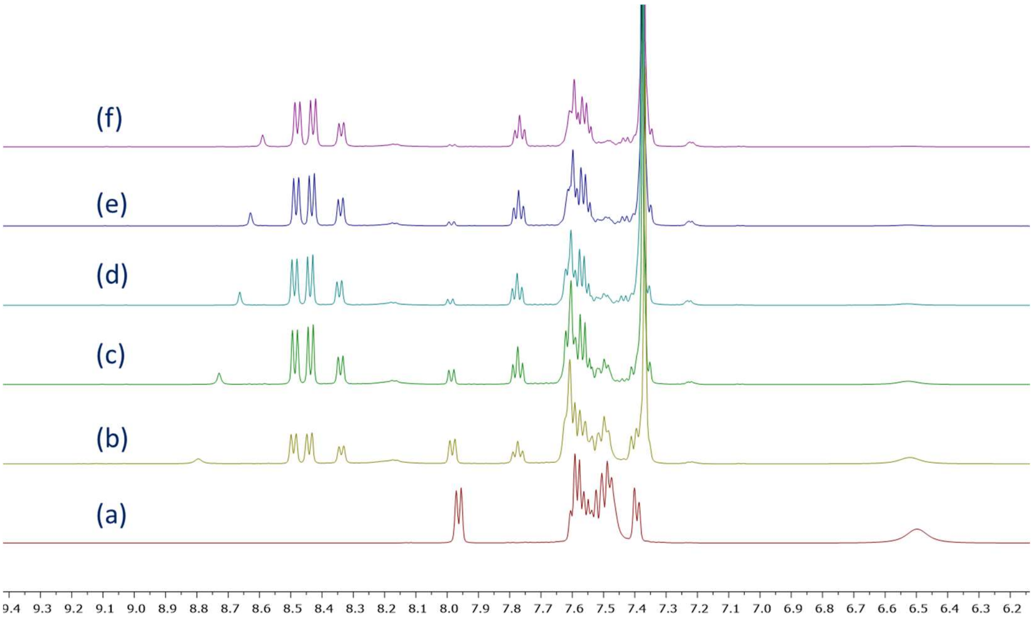

Figure S31. In-situ ^{1}H NMR spectra for the dehydrobromination and borylation of **14-Cl-amineborane** in DCM: (a) **14-Cl-amineborane**; (b) 1 eq. PhTMS, 30 mol% $HNTf_2$, 6 days; (c) = (b) + 0.5 eq. PhTMS, 4 days; (d) = (c) + 0.2 eq. PhTMS, 24 hours; (e) = (d) + 0.2 eq. PhTMS, 24 hours; (f) = (e) + 0.1 eq. PhTMS, 24 hours.

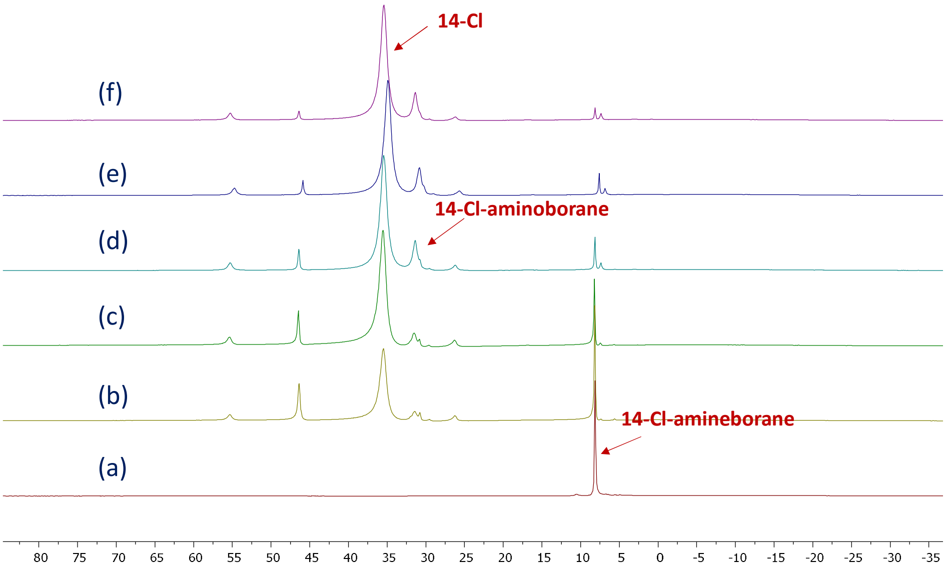

Figure S32. In-situ ^{11}B NMR spectra for the dehydrobromination and borylation of **14-Cl-amineborane** in DCM: (a) **14-Cl-amineborane**; (b) 1 eq. PhTMS, 30 mol% $HNTf_2$, 6 days; (c) = (b) + 0.5 eq. PhTMS, 4 days; (d) = (c) + 0.2 eq. PhTMS, 24 hours; (e) = (d) + 0.2 eq. PhTMS, 24 hours; (f) = (e) + 0.1 eq. PhTMS, 24 hours.

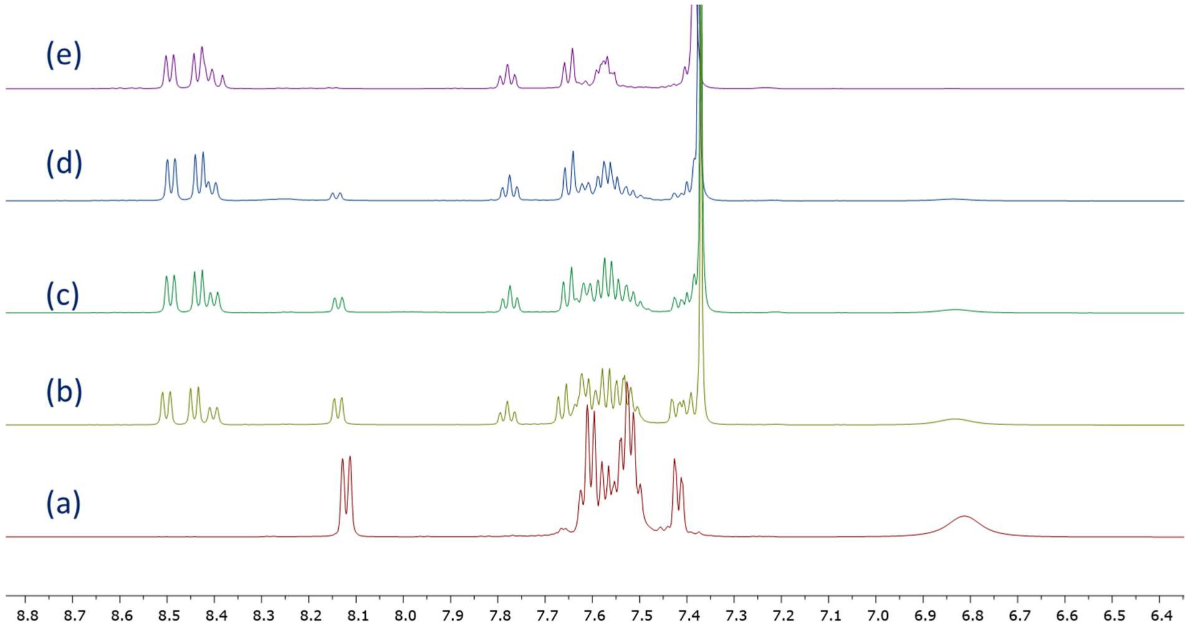

Figure S33. In-situ ^{1}H NMR spectra for the dehydrobromination and borylation of **14-Br-amineborane** in dichloromethane: (a) **14-Br-amineborane**; (b) 1 eq. PhTMS, 30 mol% $HNTf_2$, 24 hours; (c) = (b) + 0.5 eq. PhTMS, 12 hours; (d) = (c) + 0.25 eq. PhTMS, 6 hours; (e) = (d) + 0.25 eq. PhTMS, 6 hours.

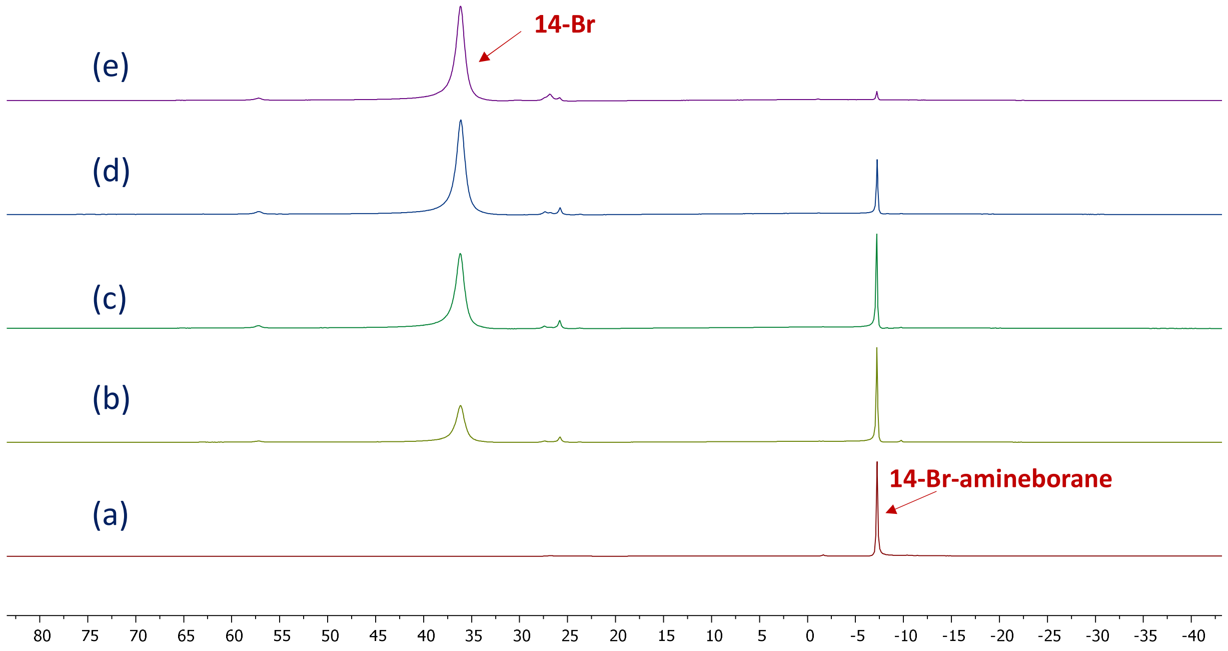

Figure S34. In-situ ^{11}B NMR spectra for the dehydrobromination and borylation of **14-Br-amineborane** in dichloromethane: (a) **14-Br-amineborane**; (b) 1 eq. PhTMS, 30 mol% $HNTf_2$, 24 hours; (c) = (b) + 0.5 eq. PhTMS, 12 hours; (d) = (c) + 0.25 eq. PhTMS, 6 hours; (e) = (d) + 0.25 eq. PhTMS, 6 hours.

S3.3.5 Dehydrochlorination of 15

(a) Attempted dehydrochlorination of 15 under thermal conditions

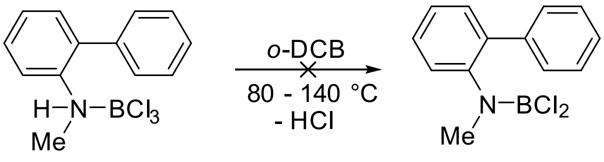

0.36 mL BCl_3 (1 M in heptane, 0.36 mmol) was added slowly to a solution of *N*-methyl-[1,1'-biphenyl]-2-amine (49 mg, 0.3 mmol) in *o*-DCB (5 mL). The mixture was stirred at room temperature for 30 minutes and 0.5 mL of the solution was taken for NMR spectroscopy. A major resonance at 8.1 ppm was observed which indicates the formation of the amine-borane adduct **15**. The reaction mixture was then heated at various temperatures under a N_2 flow. During the reaction, small portions of the mixture were taken for NMR studies. However, no dehydrochlorination product **16** (δ_{11B} = 31.7 ppm) was generated based on the ^{11}B NMR spectra.

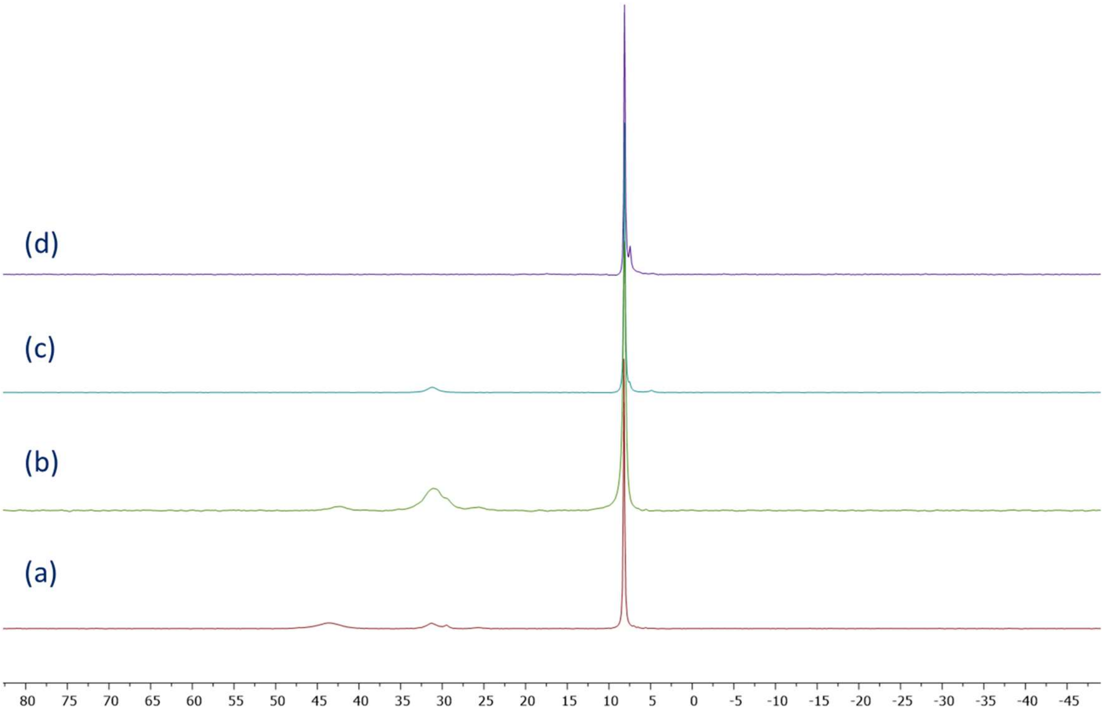

Figure S35. In-situ ^{11}B NMR spectra in *o*DCB showing no dehydrochlorination of **15** upon heating. (a) **15**; (b) 80 °C, 3 hours; (c) 100 °C, 16 hours; (d) 140 °C, 3 hours.

(b) Catalysed dehydrochlorination of 8-Cl with 40 mol% $HNTf_2$ and PhTMS

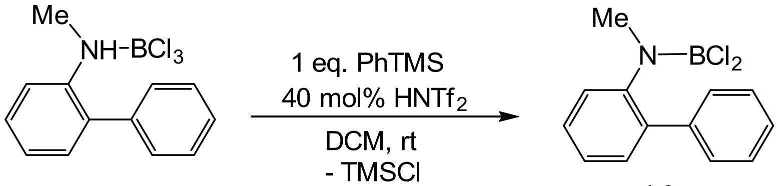

15 **16**

To a J-Young NMR tube loaded with **15** (0.1 mmol) and PhTMS (0.1 mmol) in 0.5 mL DCM was added $HNTf_2$ (14 mg, 0.04 mmol). The sample was kept at room temperature and the reaction progress was monitored by NMR spectroscopy over time. After 7 hours, almost all the amineborane adduct **15** was converted into **16**.

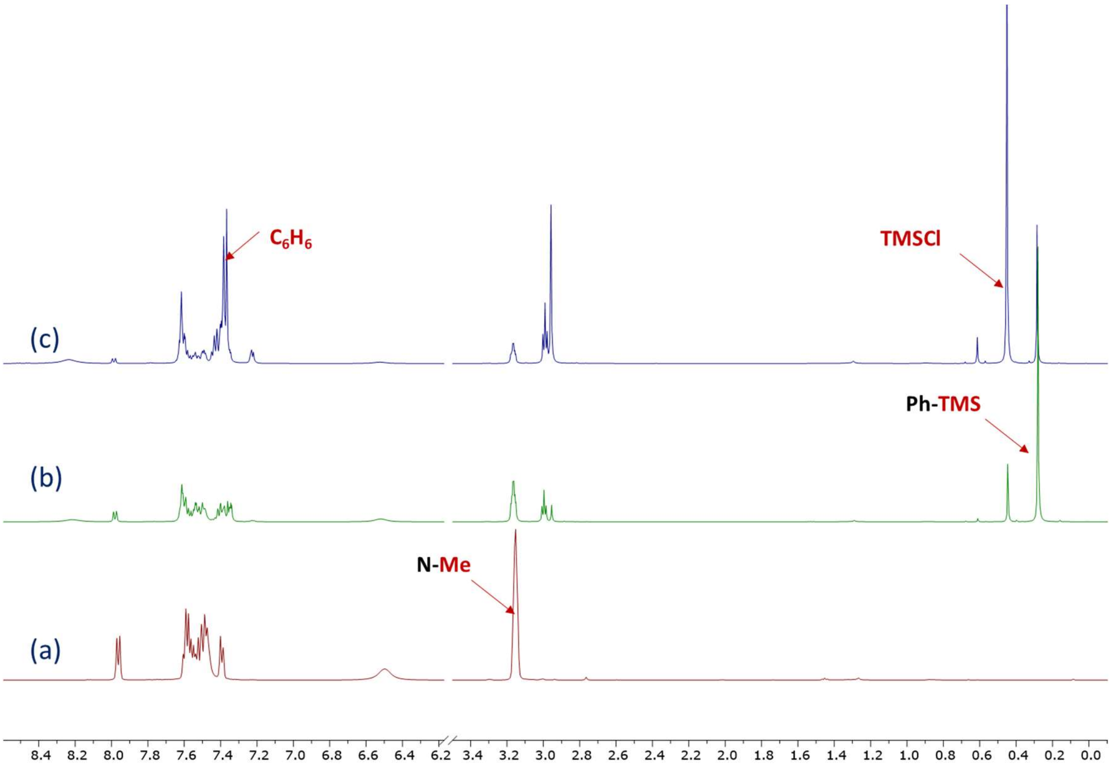

Figure S36. In-situ ^{1}H NMR spectra for the dehydrochlorination of **15** with 40 mol% $HNTf_2$ and 1 eq. PhTMS. (a) **15**; (b) rt, 30 minutes; (c) rt, 7 hours.

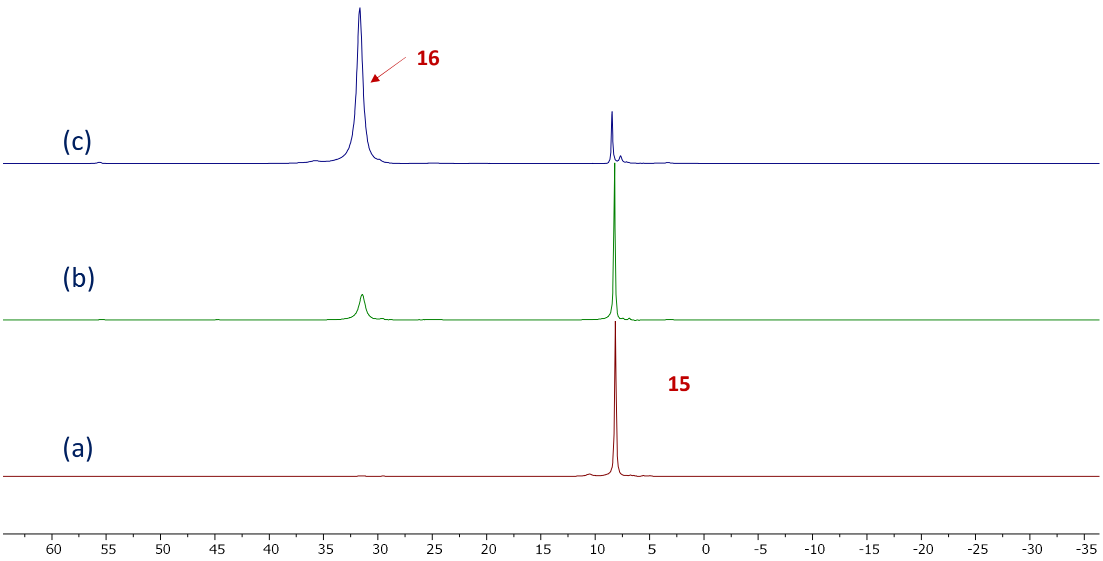

Figure S37. In-situ ^{11}B spectra for the dehydrochlorination of **15** with 40 mol% $HNTf_2$ and 1 eq. PhTMS. (a) **15**; (b) rt, 30 minutes; (c) rt, 7 hours.

S5. Resolution of (*rac*)-6-Mes and probing of racemisation barrier for 1-Mes and 6-Mes

Resolution of (*rac*)-6-Mes

Analytical HPLC

The purity of samples was verified by analytical HPLC using a column with a chiral stationary phase from Dr. Maisch GmbH (ReproSil Chiral-MIX, 250 × 4.6 mm, particle size = 5 µm). The samples of (*rac*)-**6-Mes**, (*P*)-**6-Mes** and (*M*)-**6-Mes** were dissolved in *n*-hexane/CH_2Cl_2 (87:13), filtered through a syringe filter prior to injection and eluted in the same solvent mixture at 20 °C with a flow rate of 1 mL min^{-1}.

Semi-preparative HPLC

Chiral resolution of (*rac*)-**6-Mes** into its enantiomers (*P*)-**6-Mes** and (*M*)-**6-Mes** was carried out on a column with a chiral stationary phase from Dr. Maisch GmbH (ReproSil Chiral-MIX, 250 × 20 mm, particle size = 5 µm). A sample of (*rac*)-**6-Mes** was dissolved in *n*-hexane/CH_2Cl_2 (87:13) filtered, injected on the column and eluted with *n*-hexane/CH_2Cl_2 (87:13) at room temperature with a flow rate of 7 mL min^{-1}. The separation of the enantiomers was achieved by using the recycling mode of the HPLC system.

Enantiomer (*M*)-**6-Mes** was isolated as the first fraction in enantiomercially pure form. (*P*)-**6-Mes** was collected as the second fraction with > 99% ee.

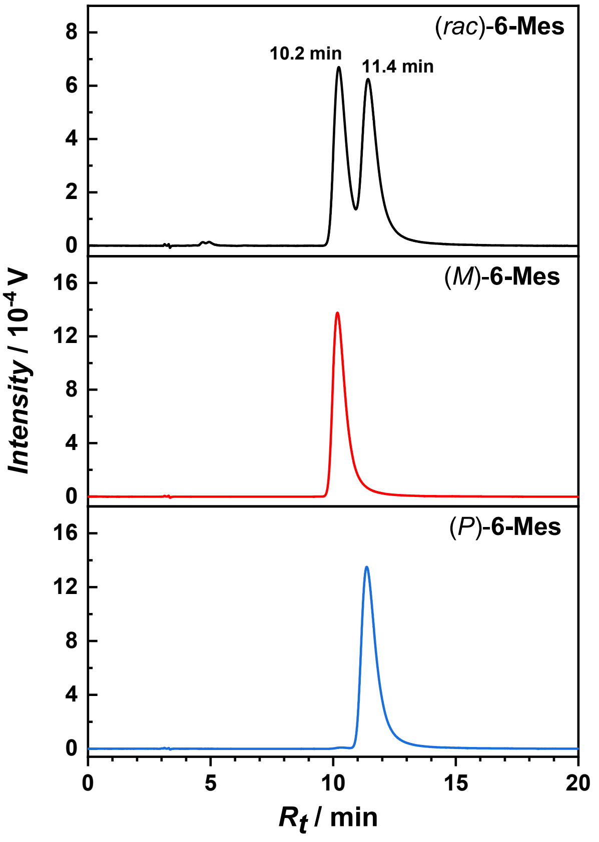

Figure S38. Analytical HPLC chromatograms of (*rac*)-**6-Mes** before and (*M*)-**6-Mes** and (*P*)-**6-Mes** after separation (c = ~0.1 mg/mL).

Verification of the Configurational Stability of (*M*)-6-Mes

For the investigation of the racemization process of compound **6-Mes**, an enantiopure sample of (*M*)-**6-Mes** (0.23 mg) was dissolved in 1,2-dichlorobenzene (0.50 mL) and heated in an oil bath. Aliquots (10 µL) were injected into an HPLC-system and could be compared qualitatively (λ = 312 nm) due to the fact that an ideal baseline separation of the respective enantiomers could not be achieved under the previously described conditions. The compound proved to be chemically stable as there was no decomposition observed even after stirring at elevated temperatures for > 2.5 d. The small peak at 3 to 4 min, that is observable in each chromatogram, corresponds to the solvent. Samples were taken after stirring the compound at 130 °C for 2 h, then at 140 °C for 2 h and afterwards at 150 °C for 16 h (Figure S38, left graph). While for the first two measurements no formation of the (*P*)-enantiomer was observed, a barely visible shoulder indicating the isomerization process could be observed after stirring the sample at 150 °C

overnight. To verify this observation, two separate samples of (*M*)-**6-Mes** (0.15 mg) were dissolved in 1,2-dichlorobenzene (0.70 mL) and heated at 140 °C and 150 °C for 64 h, respectively (Figure S38, right graph). After that time, formation of the corresponding (*P*)-enantiomer was observed in the chromatograms of both samples, although the racemization process is significantly faster at 150 °C than at 140 °C. Thus, the compound is configurationally stable at room temperature and the racemization process is extremely slow even for prolonged stirring at temperatures as high as 140 °C.

While the insufficient baseline separation did not allow to determine the Gibbs free energy of activation by time-dependent HPLC measurements, we attempted to estimate the composition of the samples obtained after heating at 140 °C and 150 °C for 64 h. To this end, the peaks were deconvoluted to give ca. 95% and ca. 89% of the (*M*)-enantiomer (please note that the estimation is not precise). Similar composition of the samples should be expected for carbo[6]helicenes under the same conditions, as calculated based on the published thermodynamic parameters (E_a and $\Delta S^{\ddagger}$) of activation.[8]

[8] (a) R. H. Martin, M. J. Marchant, *Tetrahedron* 1974, ***30***, 347; (b) R. H. Janke, G. Haufe, E.-U. Würthwein, J. H. Borkent, *J. Am. Chem. Soc.* 1996, ***118***, 6031.

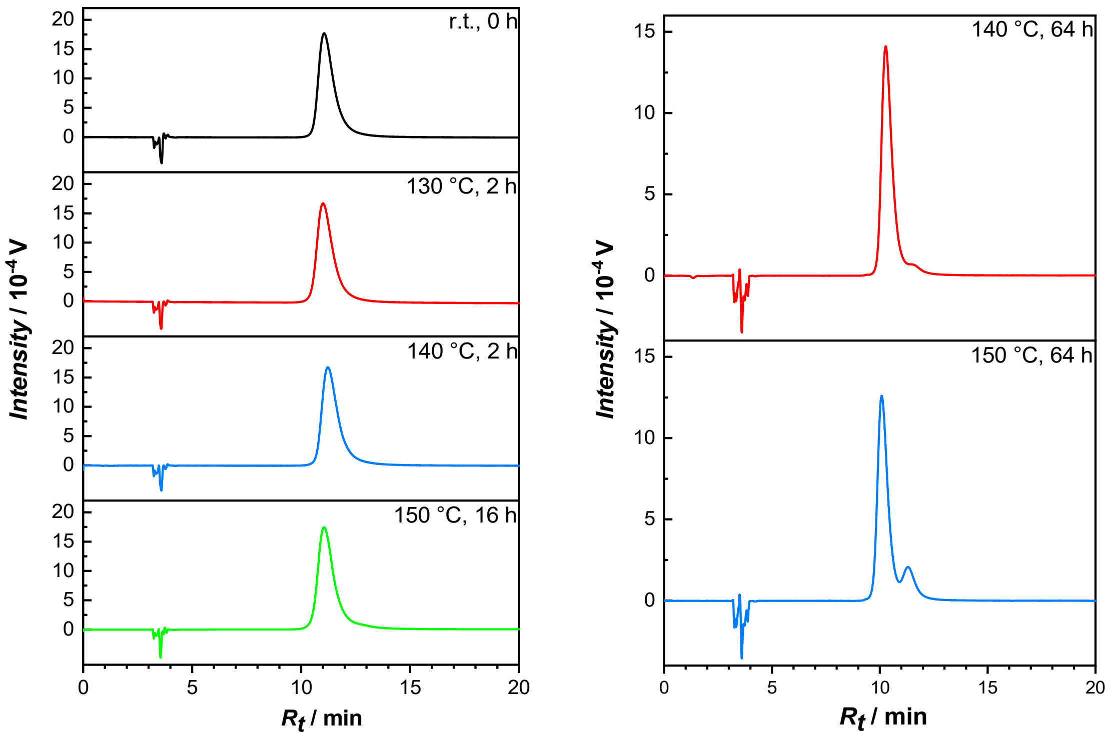

Figure S39. Analytical HPLC chromatograms of samples (*M*)-**6-Mes** a) before and after heating in an oil bath at 130 °C for 2 h, then at 140 °C for 2 h (total 4 h), and at 150 °C for 16 h (total 20 h); b) after heating in an oil bath at 140 °C for 64 h and at 150 °C for 64 h.

Probing the racemisation barrier for 1-Mes

Both enantiomers of **1-Mes** were separated by HPLC on an Agilent 1100 Series system using a Daicel Chiralpak AD-H analytical column (n-hexane : i-PrOH = 97 : 3; 1 mL/min; UV detection). Under optimised conditions, one trial run was performed immediately before the attempted separation to assess the retention times of both isomers. Then, the eluate was collected manually in fractions of 0.5 mL from 5 min before elution of the first isomer to 5 min past elution of the second isomer directly after the column (no detector / fraction sampler was used). Fractions containing **1-Mes** were detected using a UV lamp (λ = 365 nm). The first three luminescent fractions were combined and the solvent was evaporated at room temperature using a stream of N_2 until the volume was about 100 µL. Enantiomeric purity was proven by the absence of the band for the second isomer in an analytical HPLC run. The sample was then kept at room temperature (ca. 20°C) in hexane and aliquots were withdrawn at certain time points.

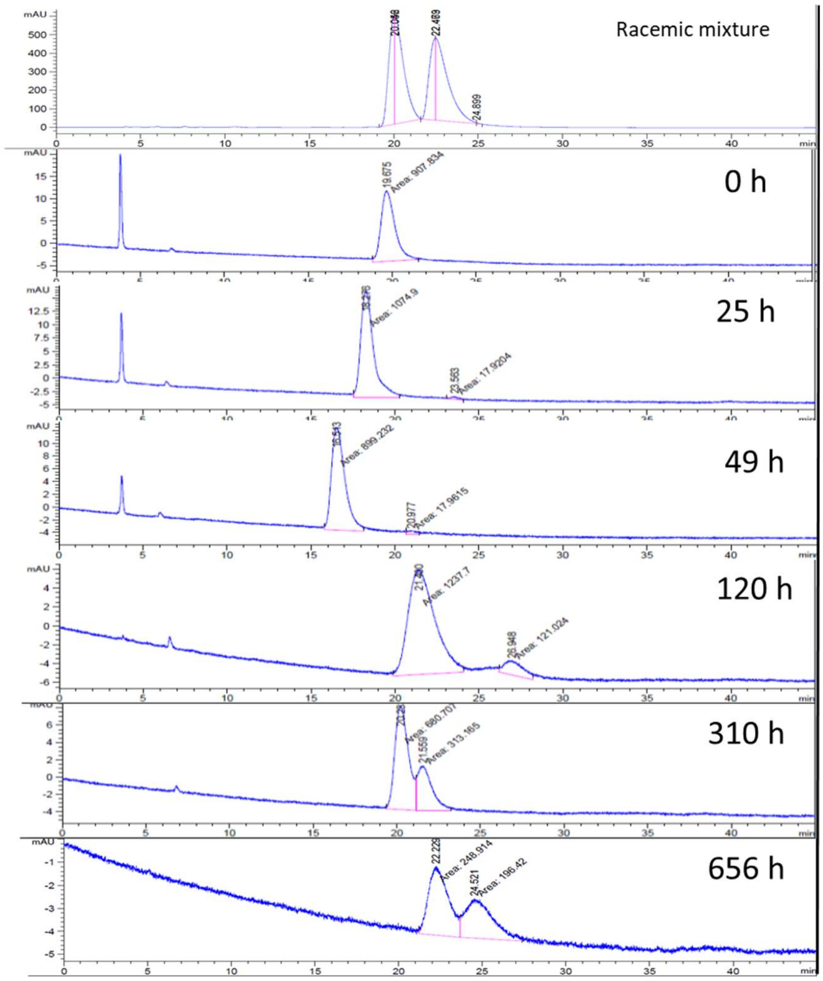

Figure S40. Top, chromatogram of racemate, all other traces show the racemisation of one enantiomer.

S6. Crystal structures

Single crystals of **1-Br** was obtained from the reaction mixture of **3.3.1 (a)**. The reaction mixture was concentrated and was kept at room temperature. Single crystals were obtained after a few days. Single crystals of **6-Mes** and **7-Mes** crystallised from a mixture of hexane and dichloromethane by slow evaporation.

Suitable crystals of **1-Br** and **7-Mes** were selected and mounted on a **Xcalibur, Eos** diffractometer. The crystals were kept at 120.00(10) K during data collection. A suitable crystal of **15-Mes** with dimensions 0.37 × 0.25 × 0.03 mm^3 was selected and mounted on a MITIGEN holder in Paratone oil. on a Rigaku Oxford Diffraction SuperNova diffractometer. The crystal was kept at a steady T = 120.00(10) K during data collection.

The structure was solved with the ShelXT[9] solution program using dual methods and by using Olex2[10] as the graphical interface. The model was refined with ShelXL[11] using full matrix least squares minimisation on $\boldsymbol{F}^2$.

[9] Sheldrick, G.M. . *Acta Cryst.* 2015, A71, 3-8.

[10] Dolomanov, O.V., Bourhis, L.J., Gildea, R.J, Howard, J.A.K. & Puschmann, H. *J. Appl. Cryst.* **2009**, *42*, 339-341.

[11] Sheldrick, G.M. . *Acta Cryst.* 2015, A71, 3-8.

Table S1. Crystal data and structure refinement for **1-Br**, **7-Mes** and **6-Mes**.

	**1-Br**	**7-Mes**	**6-Mes**
CCDC NO	**2084378**	**2084379**	**2084380**
Empirical formula	$C_{20}H_{13}BNBr$	$C_{34}H_{30}BN$	$C_{33}H_{26}BN$
Formula weight	358.03	463.4	447.36
Temperature/K	120.00(10)	120.1(4)	120.00(10)
Crystal system	orthorhombic	monoclinic	triclinic
Space group	$Pca2_1$	$P2_1/c$	P-1
a/Å	22.9580(7)	12.0461(6)	6.6409(2)
b/Å	9.4271(3)	17.1985(5)	12.0504(4)
c/Å	7.0146(3)	13.0700(6)	15.9138(6)
α/°	90	90	78.061(3)
β/°	90	109.463(5)	78.902(3)
γ/°	90	90	79.901(3)
Volume/$Å^3$	1518.15(9)	2553.0(2)	1210.41(8)
Z	4	4	2
$\rho_{calc}g/cm^3$	1.566	1.206	1.227
μ/mm^{-1}	2.704	0.068	0.528
F(000)	720	984	472
Crystal size/mm^3	0.232 × 0.197 × 0.11	0.46 × 0.101 × 0.05	0.368 × 0.251 × 0.033
Radiation	Mo Kα (λ = 0.71073)	Mo Kα (λ = 0.71073)	Cu Kα (λ = 1.54184)
2Θ range for data collection/°	6.808 to 58.688	6.812 to 58.97	7.574 to 153.368
Index ranges	$-31 \leq h \leq 30$, $-12 \leq k \leq 12$, $-9 \leq l \leq 9$	$-15 \leq h \leq 16$, $-23 \leq k \leq 22$, $-17 \leq l \leq 16$	$-8 \leq h \leq 8$, $-15 \leq k \leq 15$, $-19 \leq l \leq 20$
Reflections collected	31855	55926	19449
Independent reflections	3769 [R_{int} = 0.0582, R_{sigma} = 0.0431]	6564 [R_{int} = 0.0827, R_{sigma} = 0.0768]	9193 [R_{int} = 0.0614, R_{sigma} = 0.0223]
Data/restraints/parameters	3769/1/208	6564/0/334	9193/64/592
Goodness-of-fit on F^2	1.035	1.017	1.053
Final R indexes [I>=2σ (I)]	R_1 = 0.0353, wR_2 = 0.0624	R_1 = 0.0623, wR_2 = 0.1182	R_1 = 0.0571, wR_2 = 0.1596
Final R indexes [all data]	R_1 = 0.0544, wR_2 = 0.0679	R_1 = 0.1324, wR_2 = 0.1424	R_1 = 0.0683, wR_2 = 0.1660
Largest diff. peak/hole / e $Å^{-3}$	0.38/-0.34	0.20/-0.20	0.18/-0.21

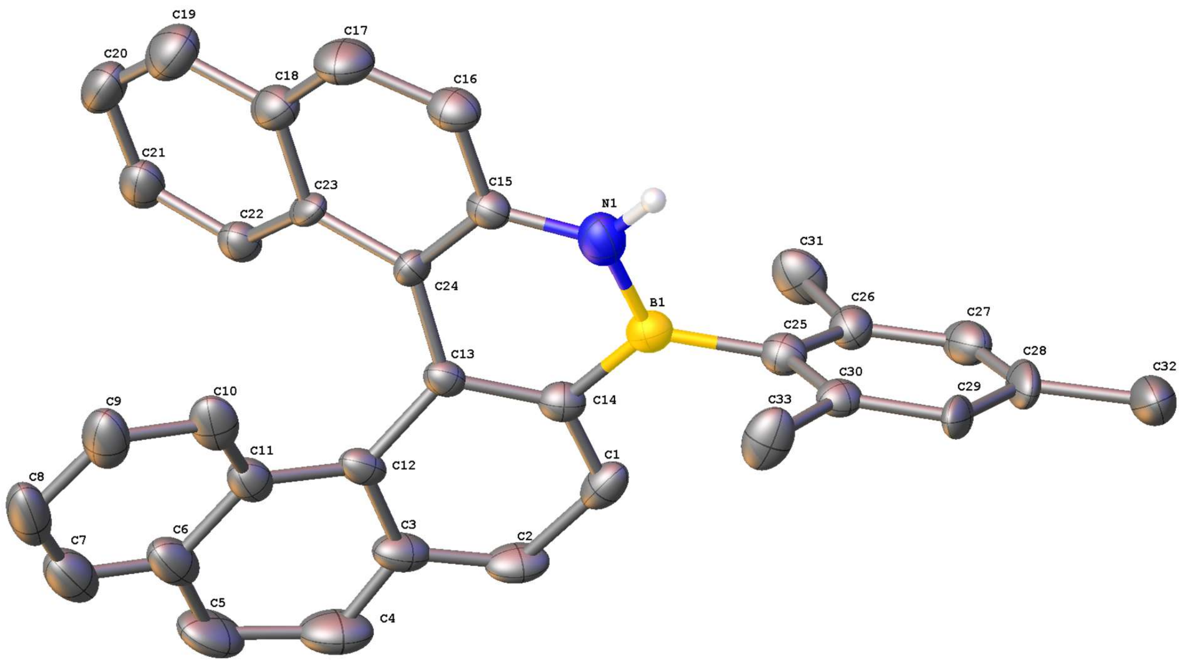

Figure S41: One of the two disorder components in **6-Mes**. Displacement ellipsoids are at the 50% probability level. C-bound H atoms are not shown.

S6. Electrochemical studies

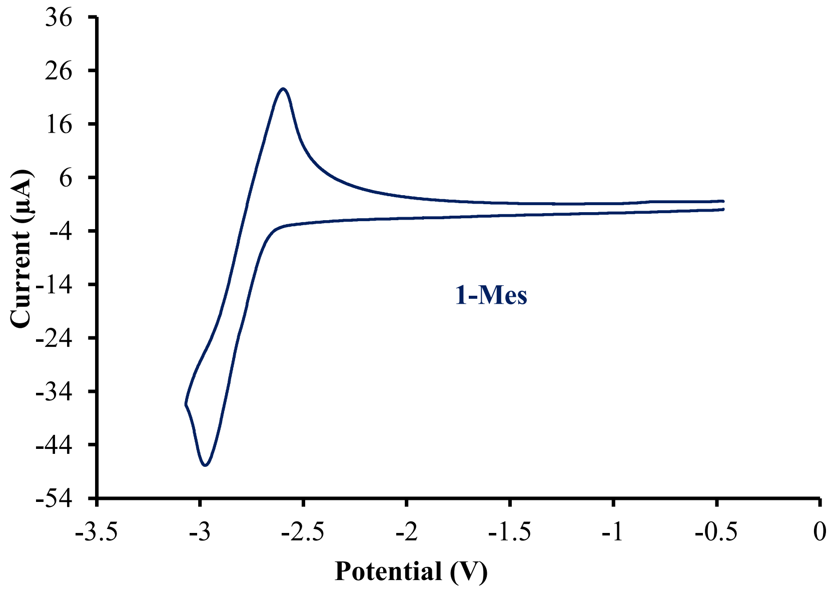

Figure S42. Cyclic voltammetry diagram of **1-Mes**. Measured in THF (1 mM), with [nBu4N][PF6] (0.1 M) as the supporting electrolyte at a scan rate of 100 mV/s (E_{peak} = -2.972 V).

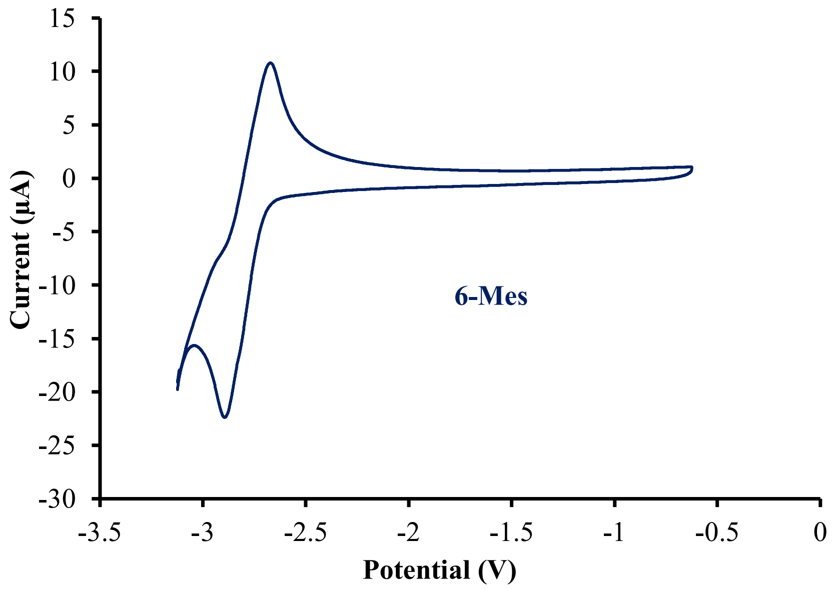

Figure S43. Cyclic voltammetry diagram of **6-Mes**. Measured in THF (1 mM), with [nBu4N][PF6] (0.1 M) as the supporting electrolyte at a scan rate of 100 mV/s (E_{peak} = -2.885 V)..

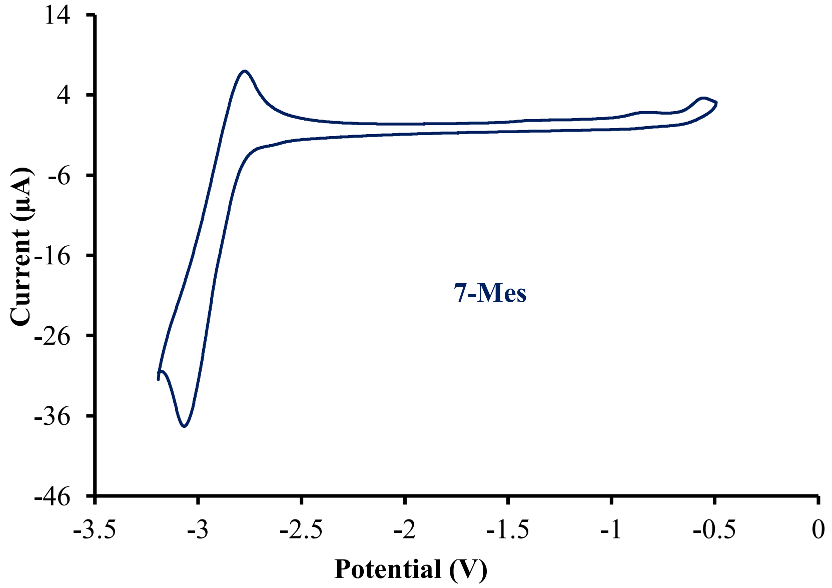

Figure S44. Cyclic voltammetry diagram of **7-Mes**. Measured in THF (1 mM), with [nBu4N][PF6] (0.1 M) as the supporting electrolyte at a scan rate of 100 mV/s (E_{peak} = -3.057 V)..

S7. NMR spectra

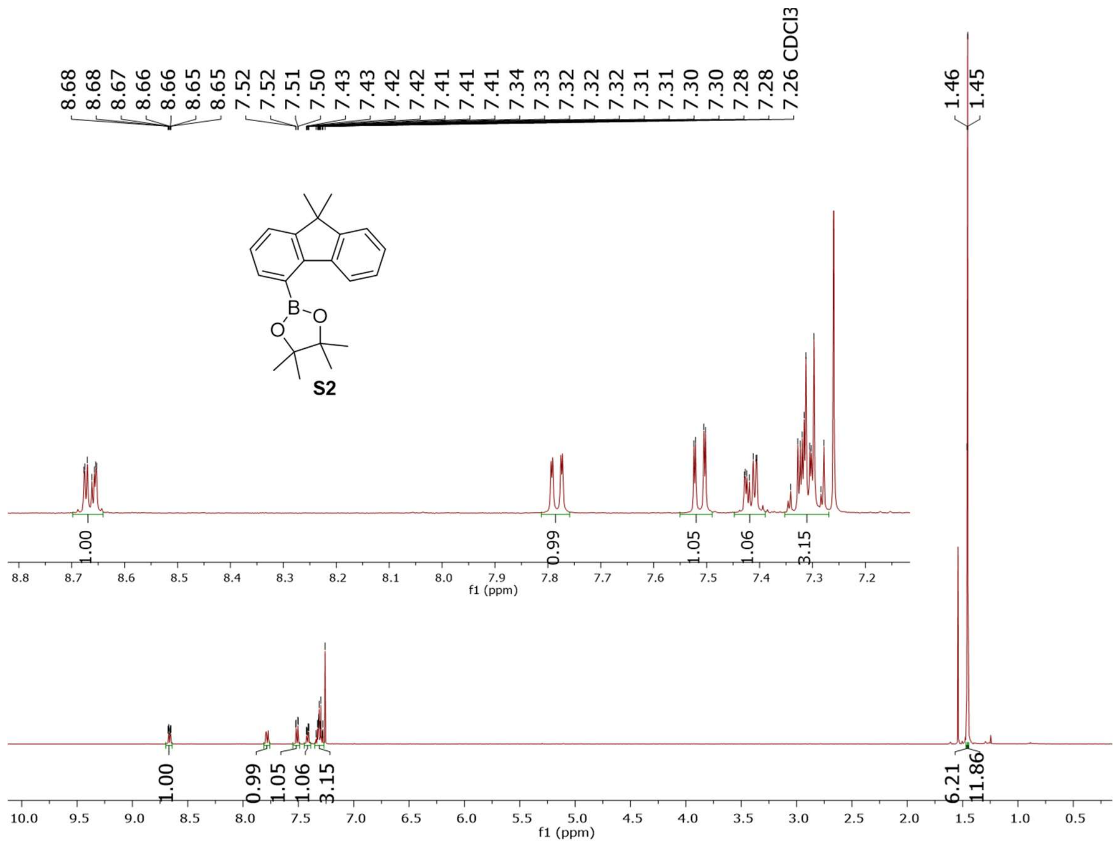

Figure S45. ^{1}H NMR spectrum of **S2** in $CDCl_3$.

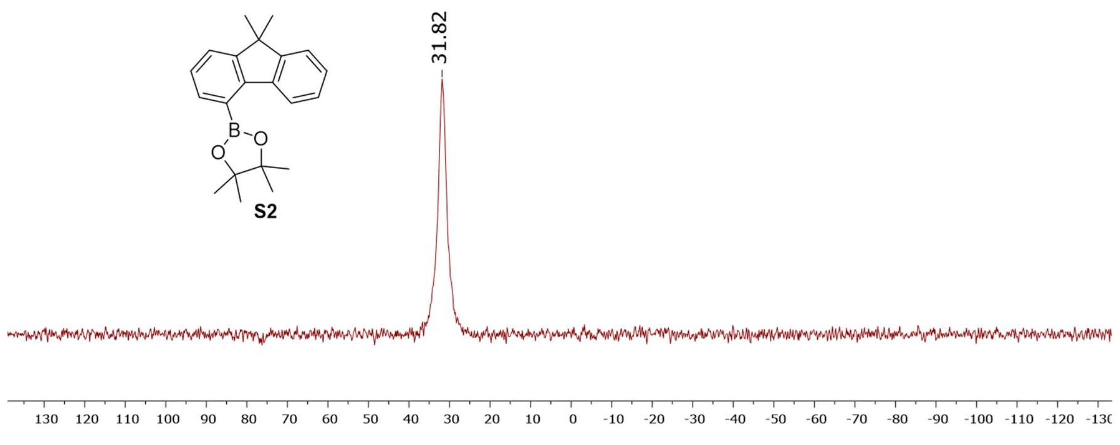

Figure S46. ^{11}B NMR spectrum of **S2** in $CDCl_3$.

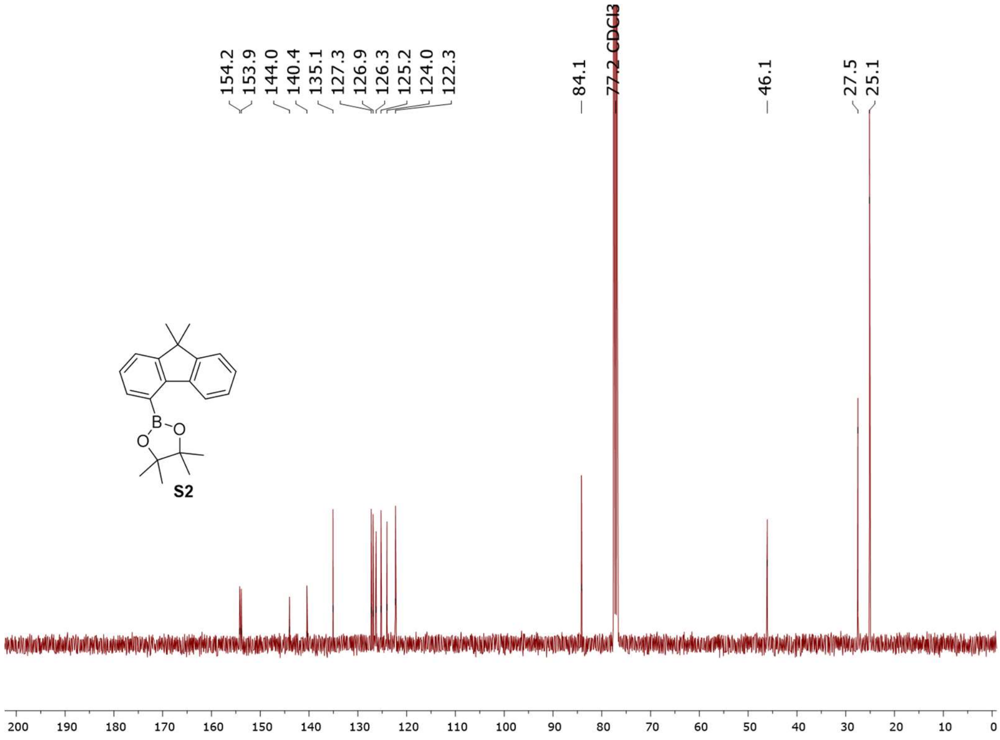

Figure S47. $^{13}C\{^{1}H\}$ NMR spectrum of **S2** in $CDCl_3$.

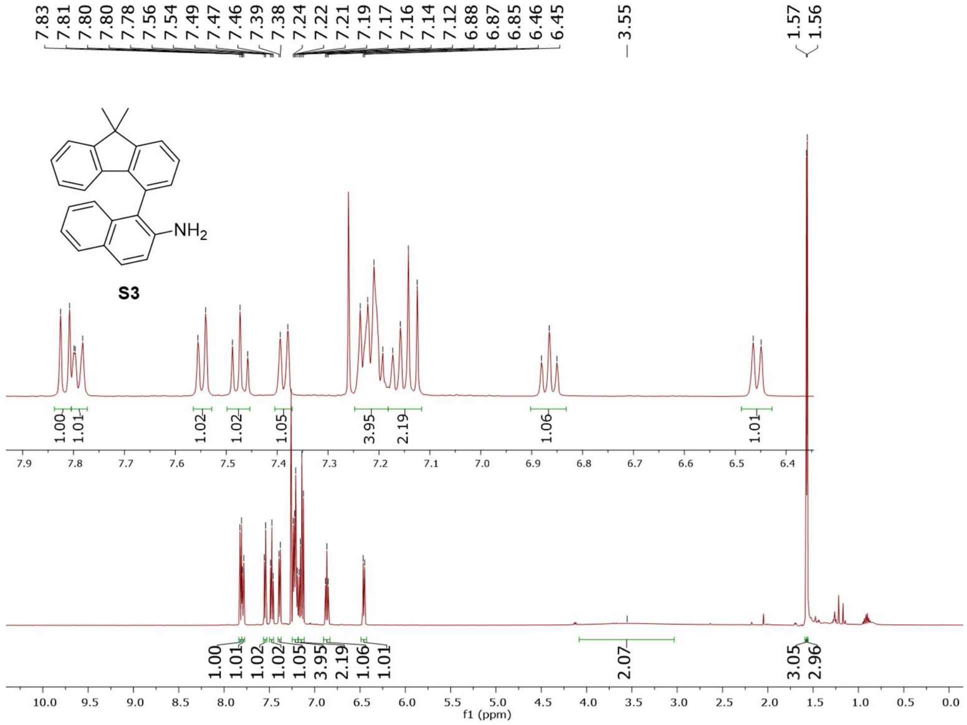

Figure S48. ^{1}H NMR spectrum of **S3** in $CDCl_3$.

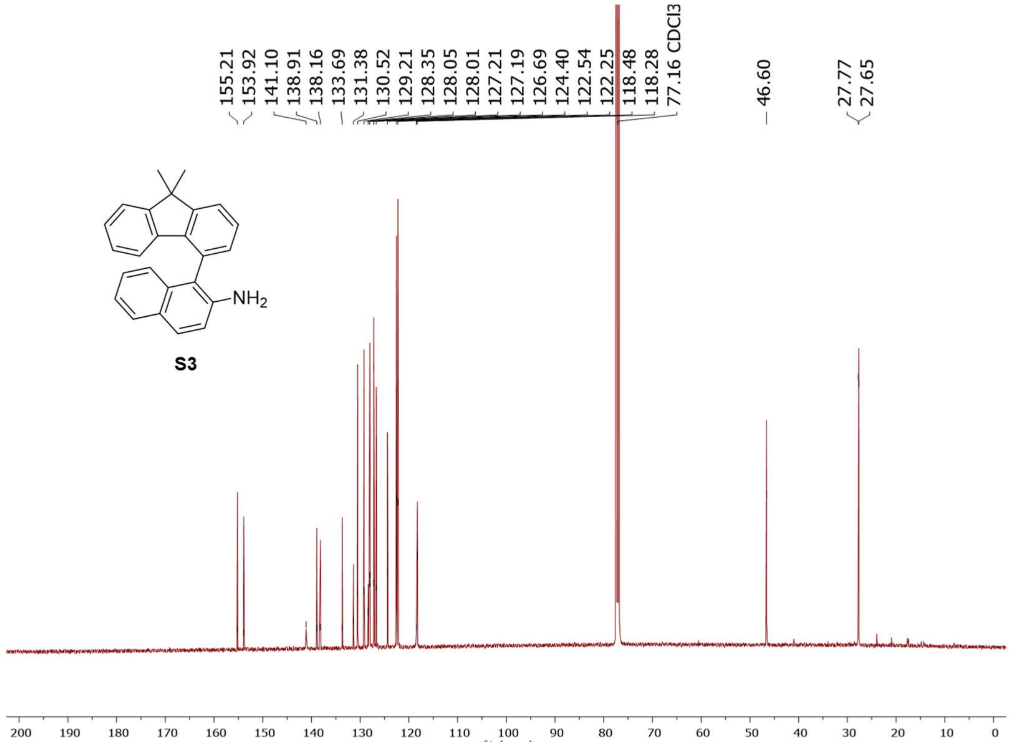

Figure S49. $^{13}C\{^1H\}$ NMR spectrum of **S3** in $CDCl_3$.

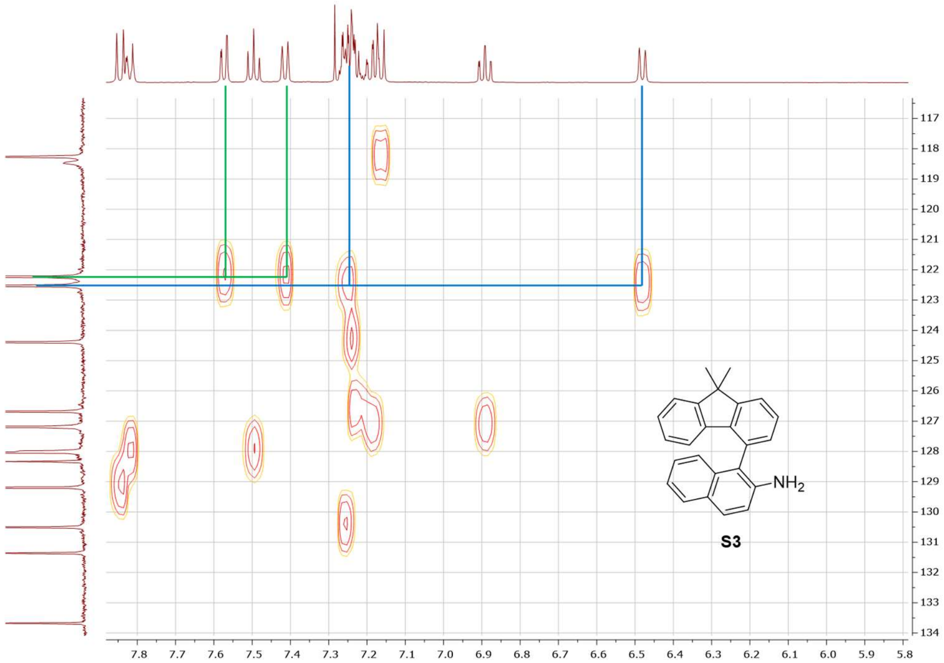

Figure S50. HSQC spectrum of **S3** in $CDCl_3$.The spectrum clearly indicates that two carbon resonances overlap.

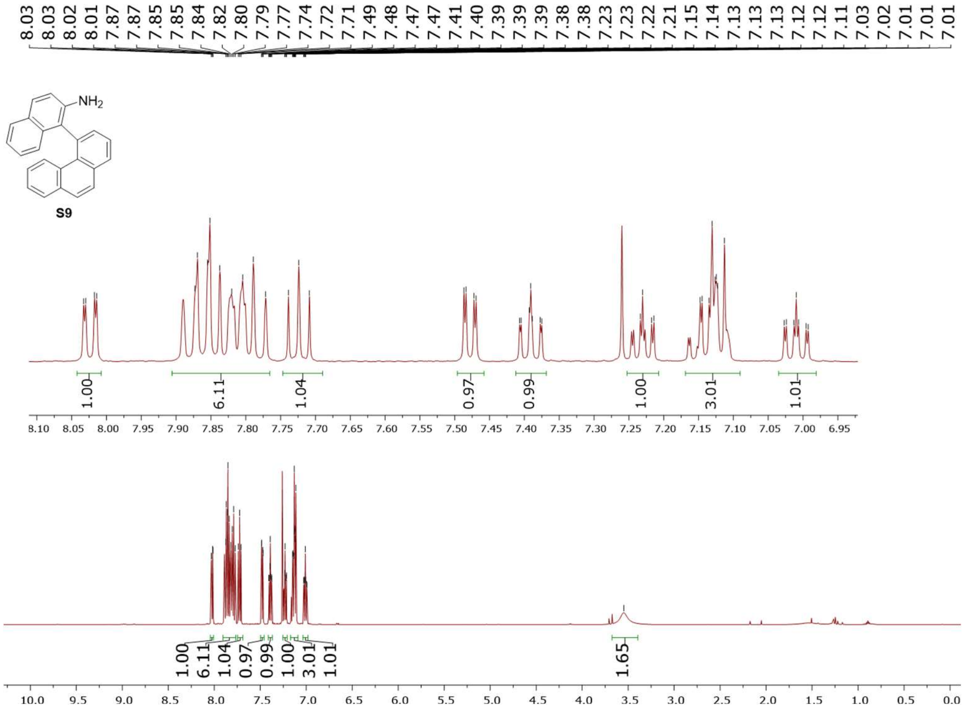

Figure S51. ^{1}H NMR spectrum of **S9** in $CDCl_3$.

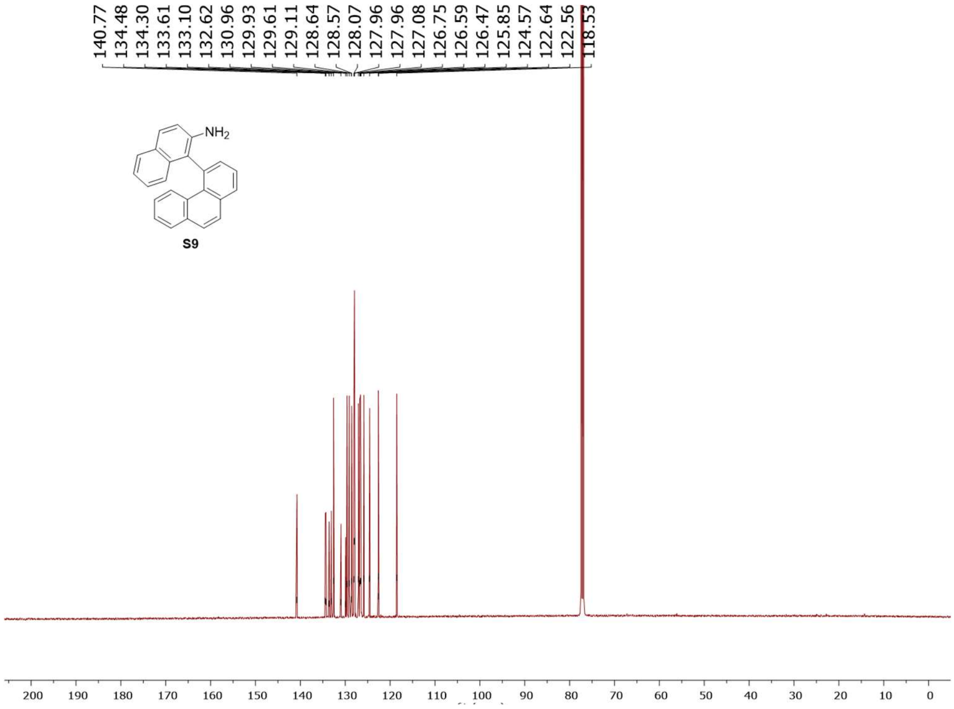

Figure S52. $^{13}C\{^{1}H\}$ NMR spectrum of **S9** in $CDCl_3$.

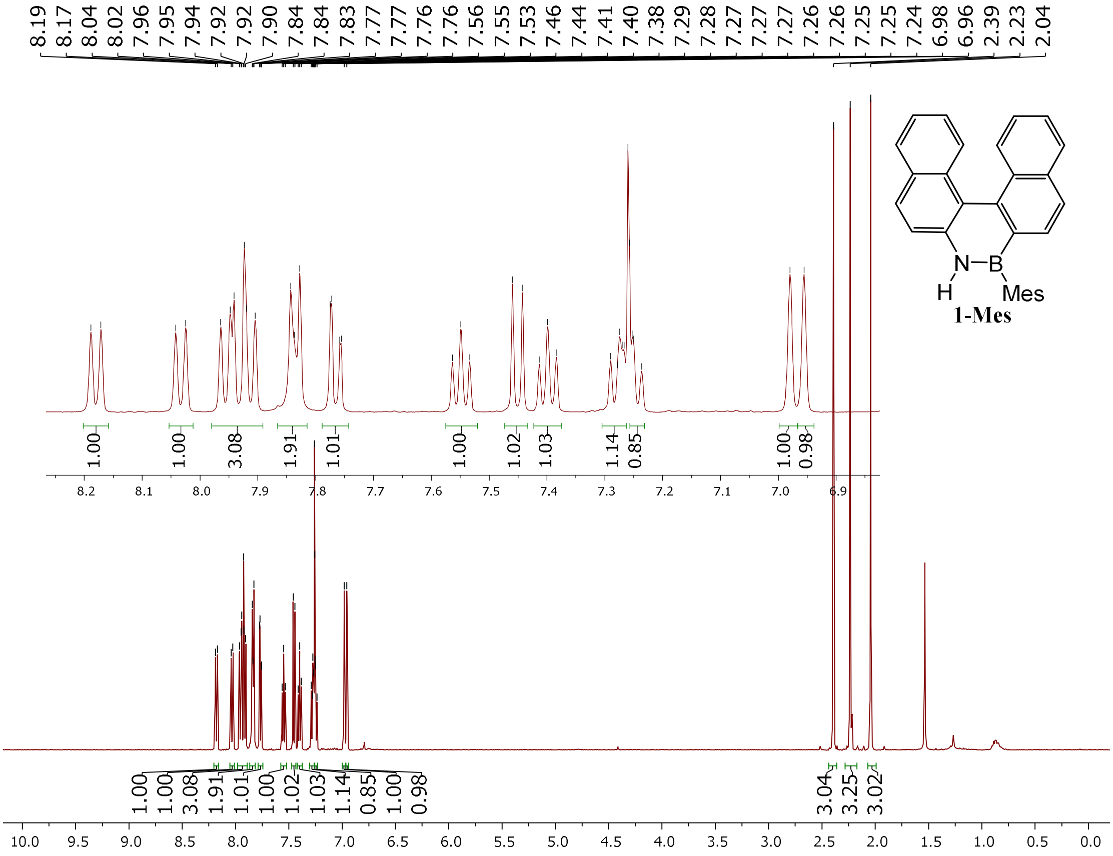

Figure S53. ^{1}H NMR spectrum of **1-Mes** in $CDCl_3$.

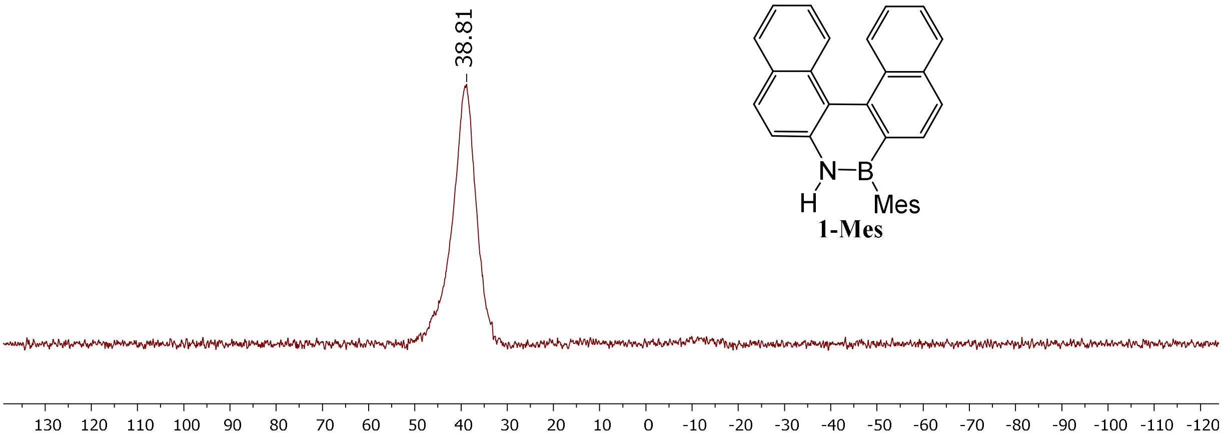

Figure S54. ^{11}B NMR spectrum of **1-Mes** in $CDCl_3$.

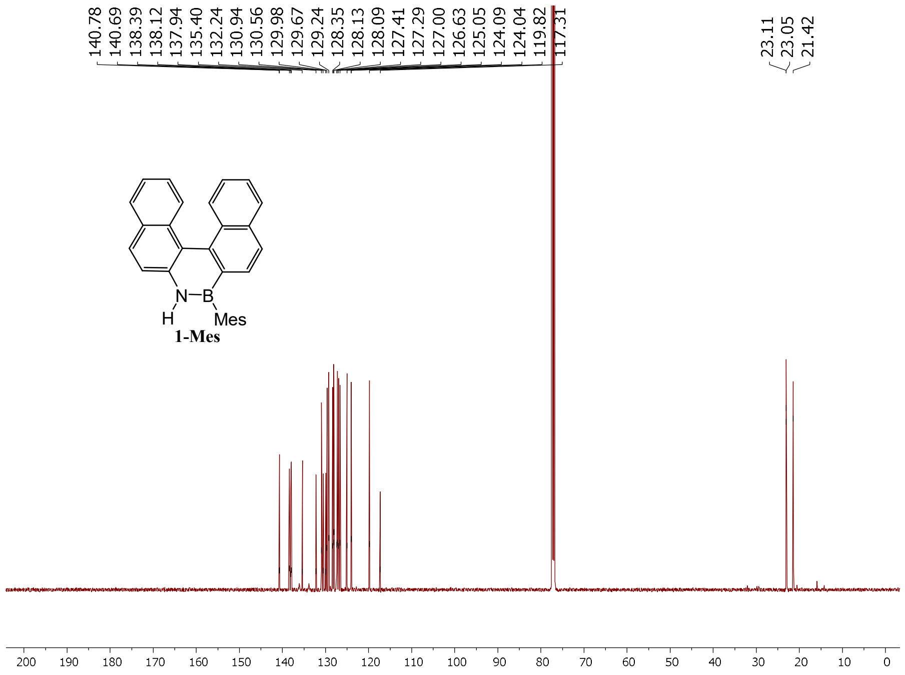

Figure S55. $^{13}C\{^1H\}$ NMR spectrum of **1-Mes** in $CDCl_3$.

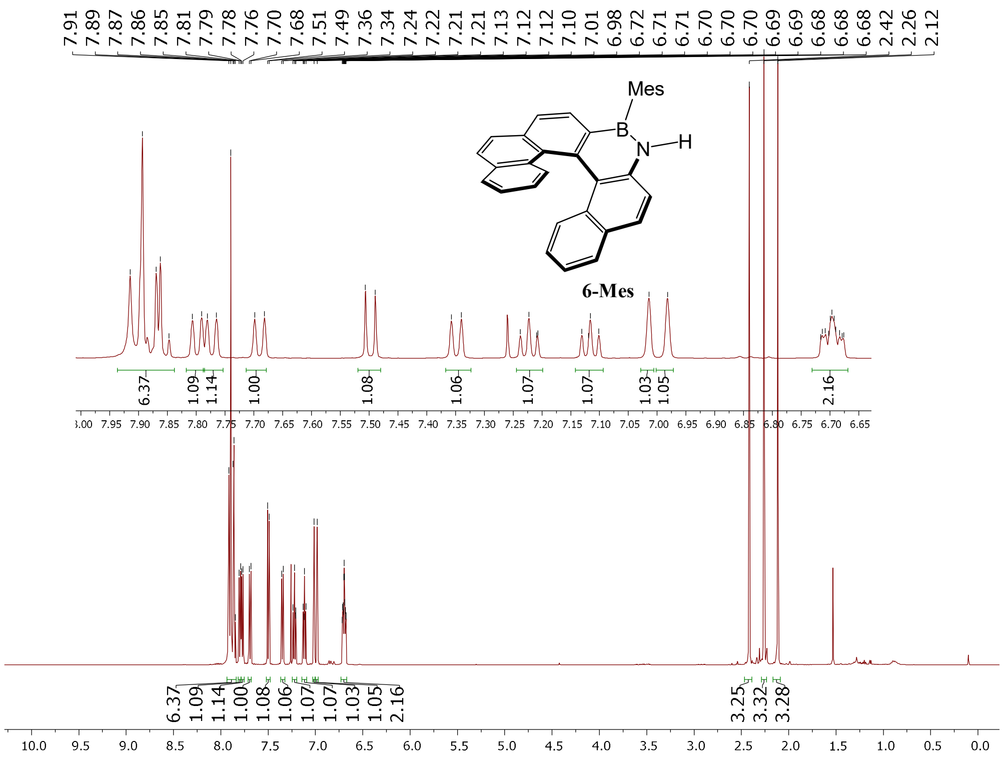

Figure S56. ^1H NMR spectrum of **6-Mes** in $CDCl_3$.

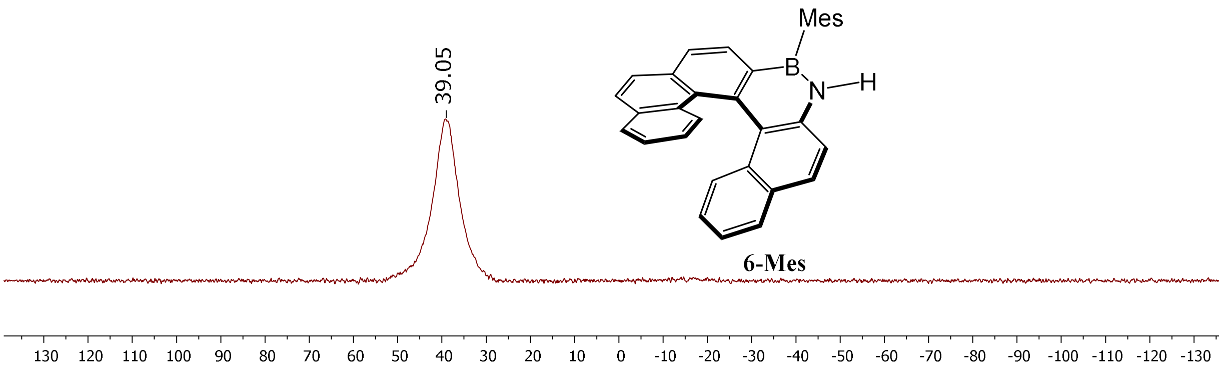

Figure S57. ^{11}B NMR spectrum of **6-Mes** in $CDCl_3$.

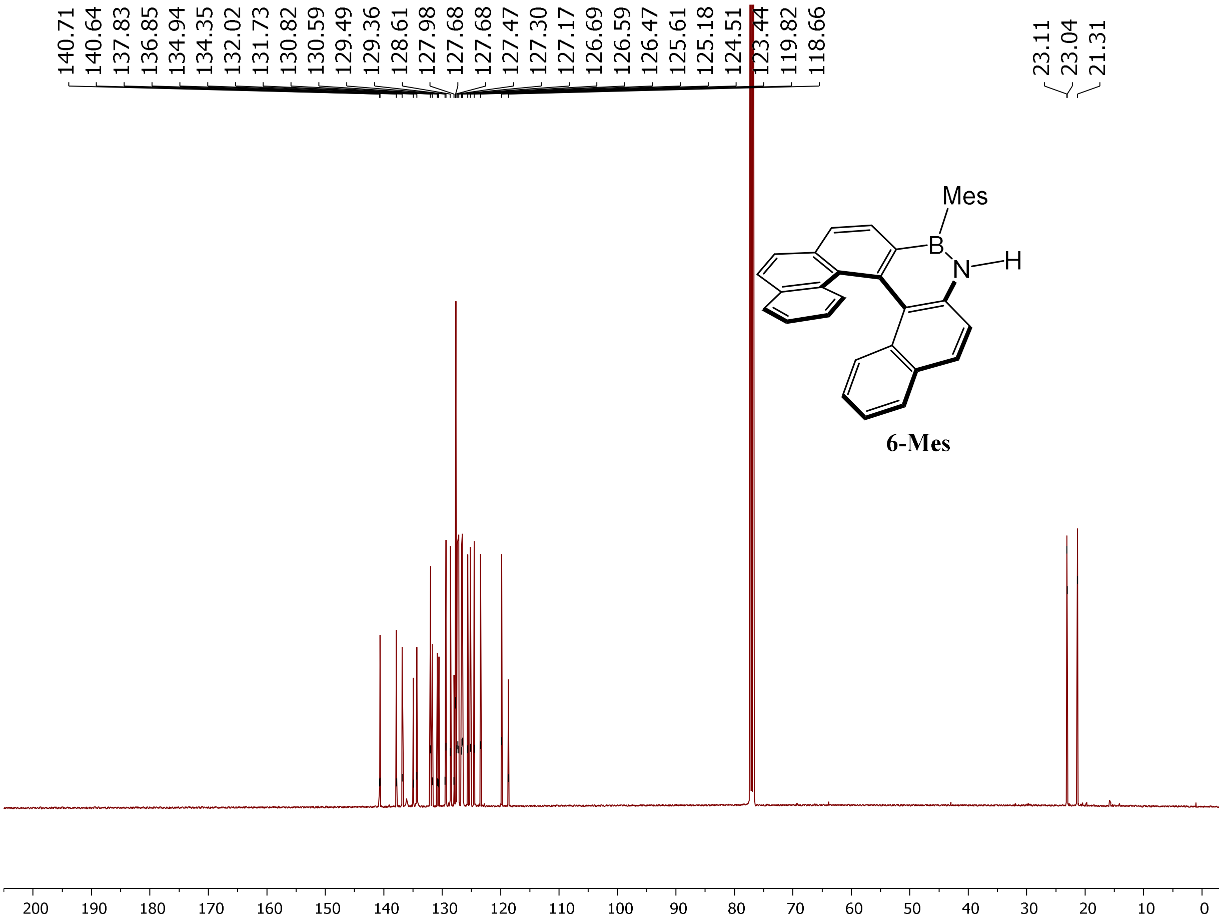

Figure S58. $^{13}C\{^1H\}$ NMR spectrum of **6-Mes** in $CDCl_3$.

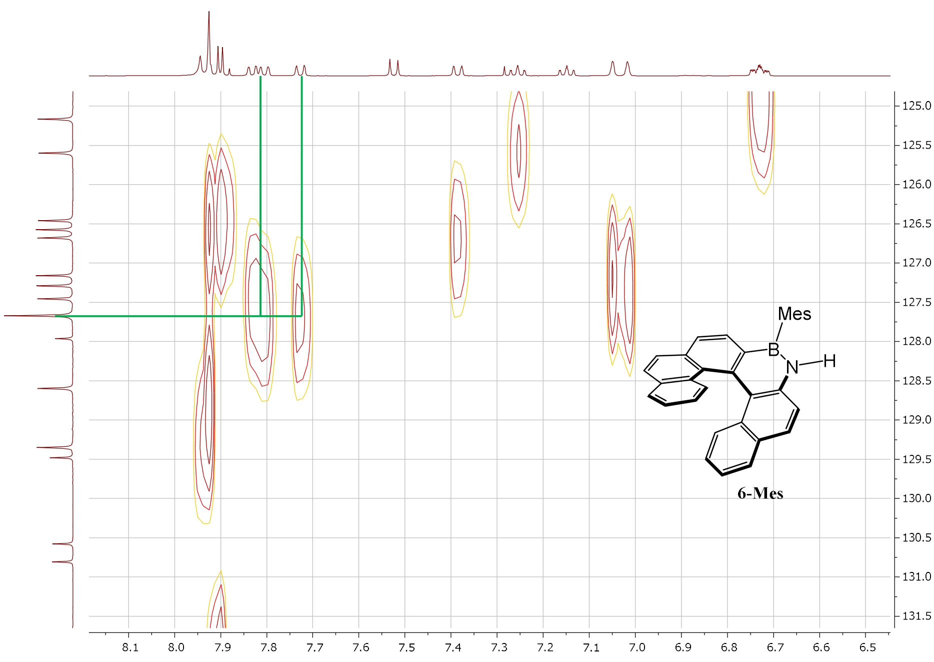

Figure S59. HSQC spectrum of **6-Mes** in $CDCl_3$.The spectrum indicates two carbon resonances overlap.

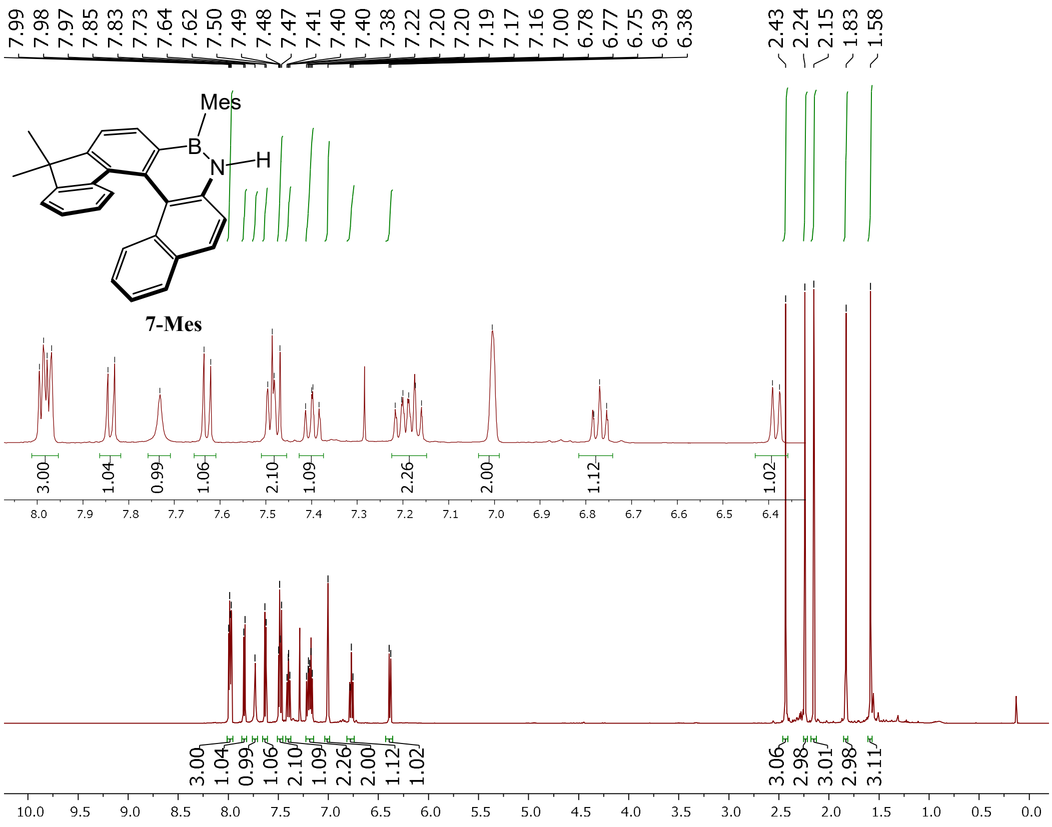

Figure S60. ^1H NMR spectrum of **7-Mes** in $CDCl_3$.

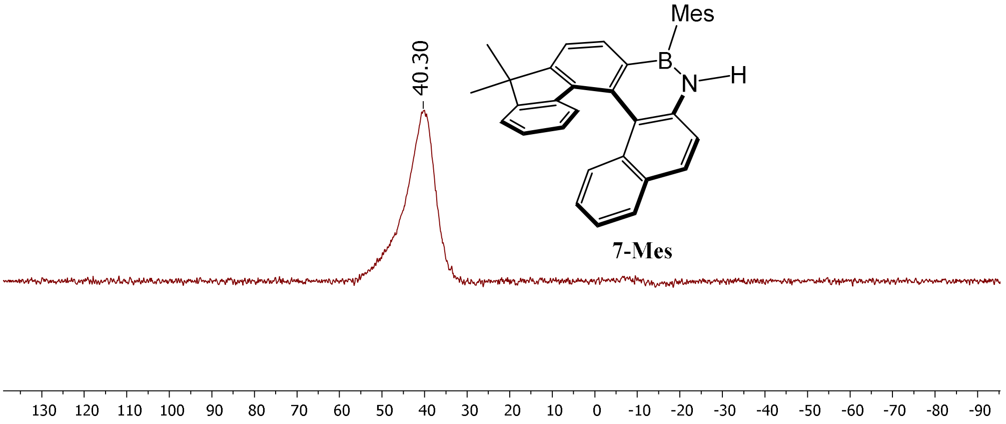

Figure S61. ^{11}B NMR spectrum of **7-Mes** in $CDCl_3$.

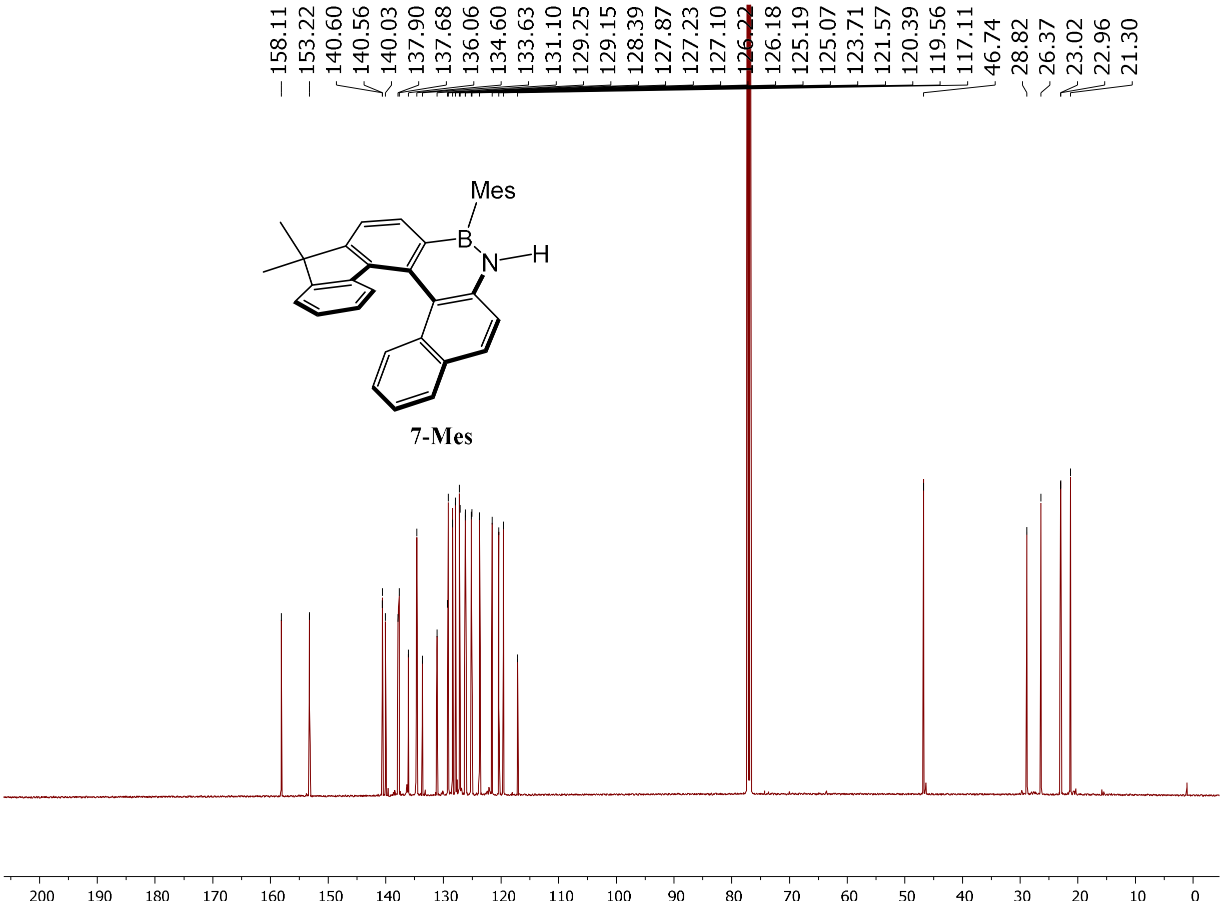

Figure S62. $^{13}C\{^{1}H\}$ NMR spectrum of **7-Mes** in $CDCl_3$.

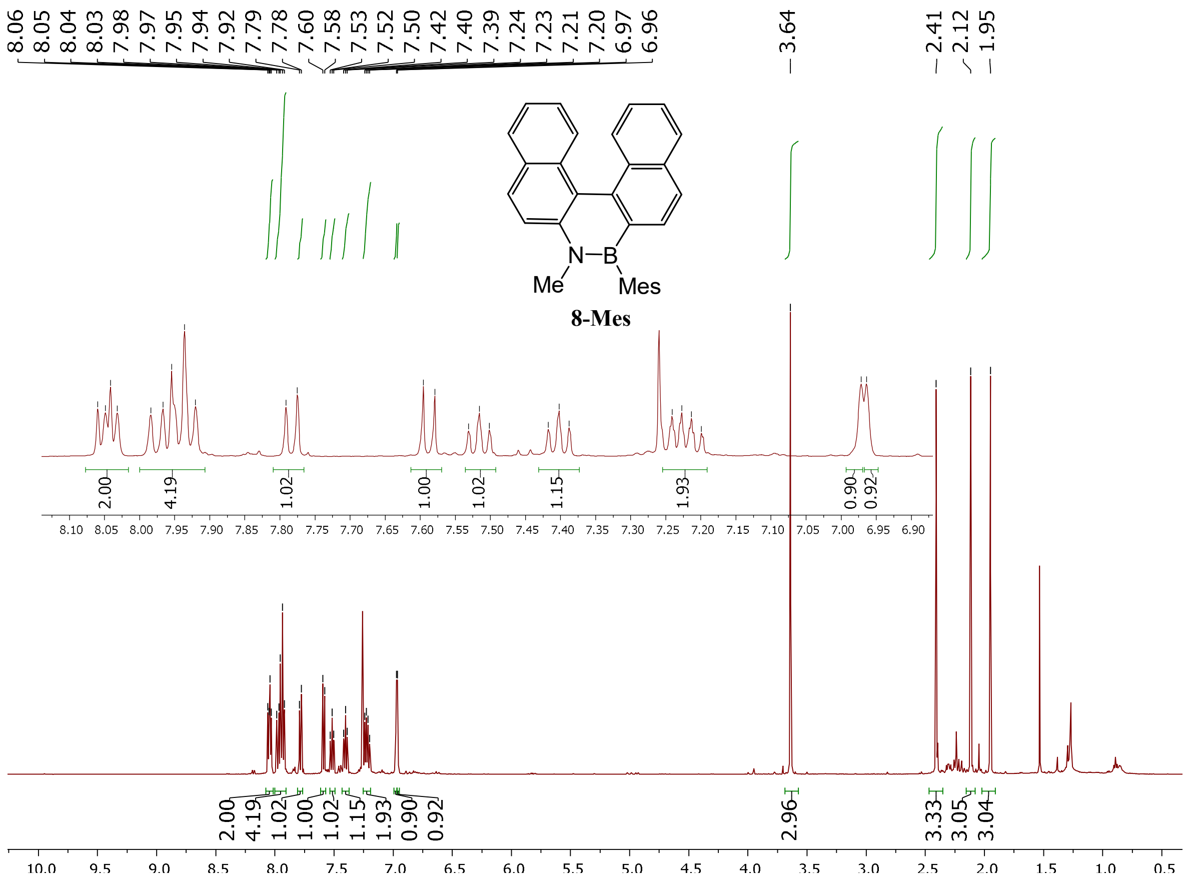

Figure S63. ^{1}H NMR spectrum of **8-Mes** in $CDCl_3$.

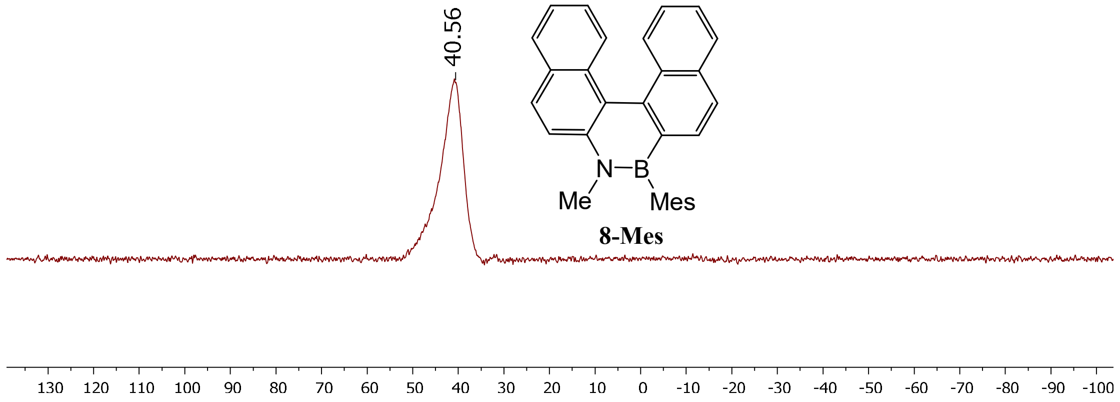

Figure S64. ^{11}B NMR spectrum of **8-Mes** in $CDCl_3$.

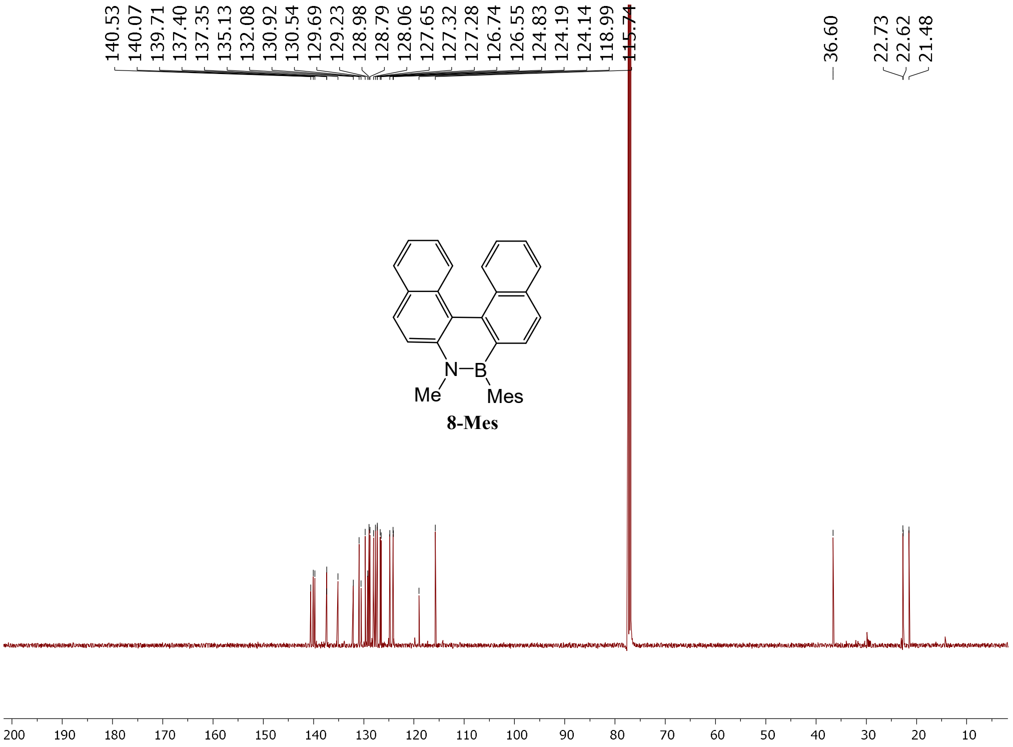

Figure S65. $^{13}C\{^{1}H\}$ NMR spectrum of **8-Mes** in $CDCl_3$.

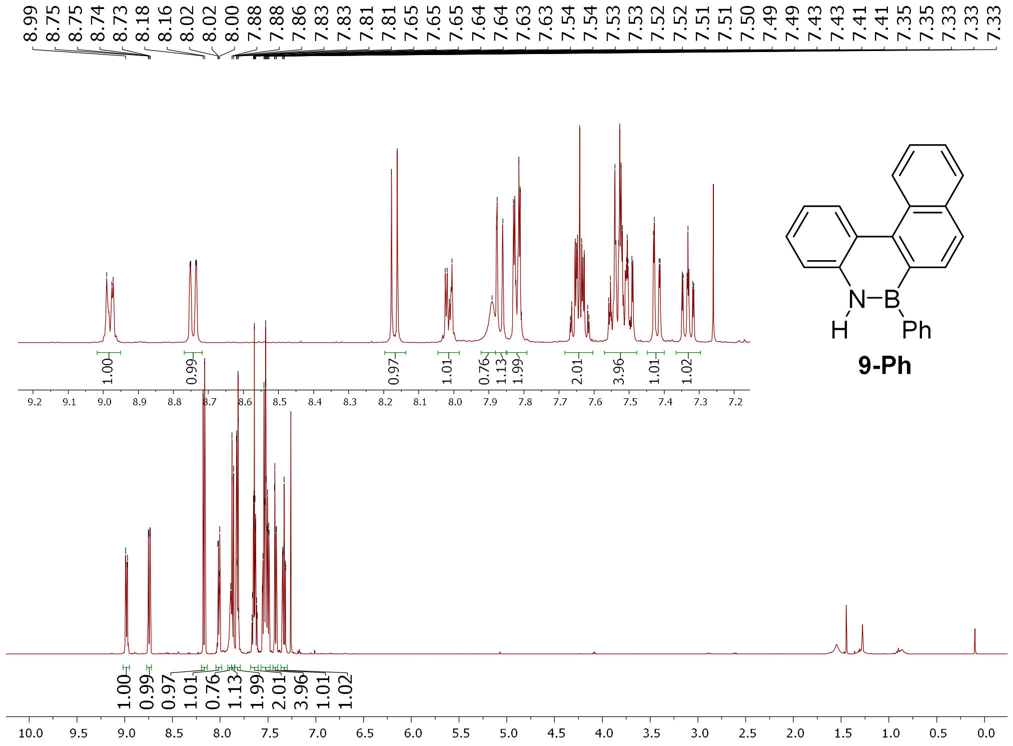

Figure S66. ^{1}H NMR spectrum of **9-Ph** in $CDCl_3$.

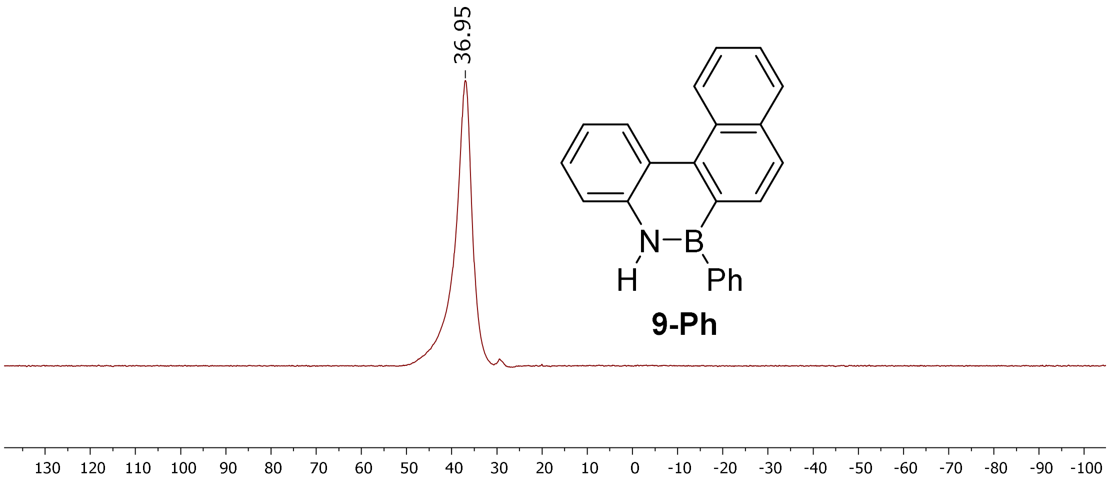

Figure S67. ^{11}B NMR spectrum of **9-Ph** in $CDCl_3$.

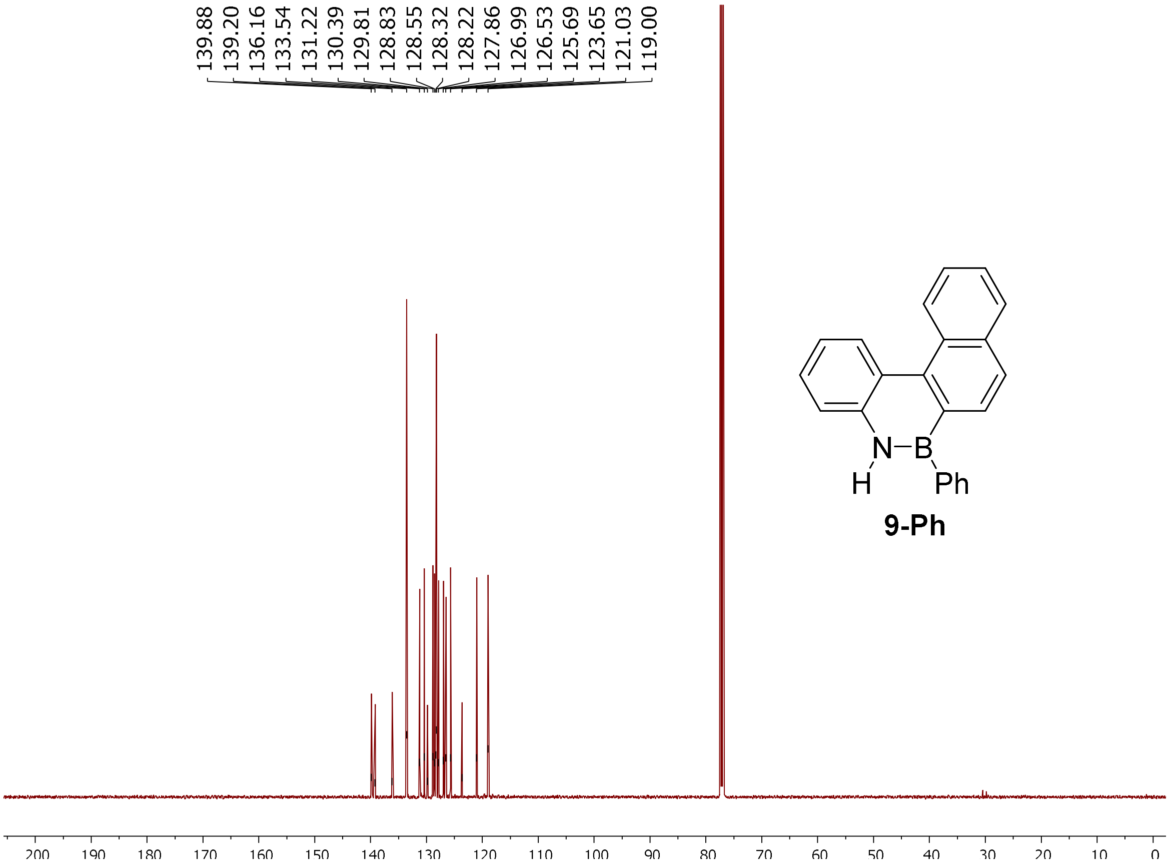

Figure S68. ^{13}C{^{1}H} NMR spectrum of **9-Ph** in $CDCl_3$.

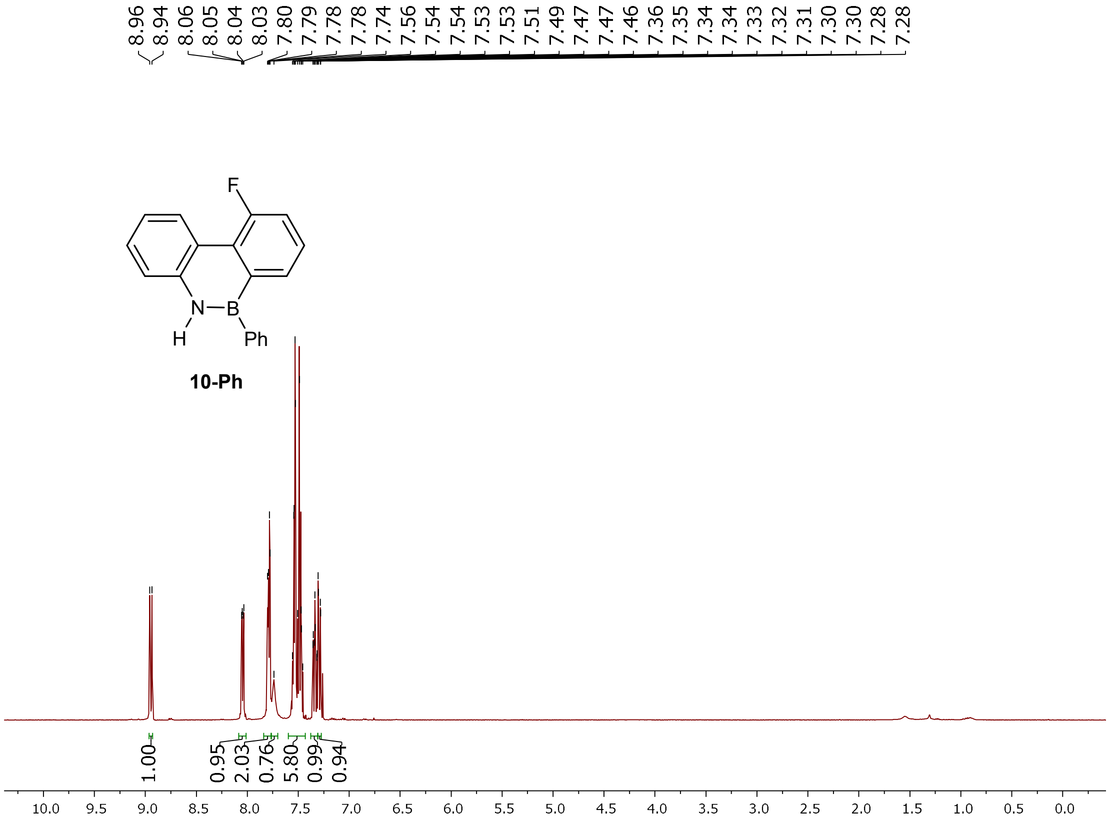

Figure S69. $^{1}H\{^{19}F\}$ NMR spectrum of **10-Ph** in $CDCl_3$.

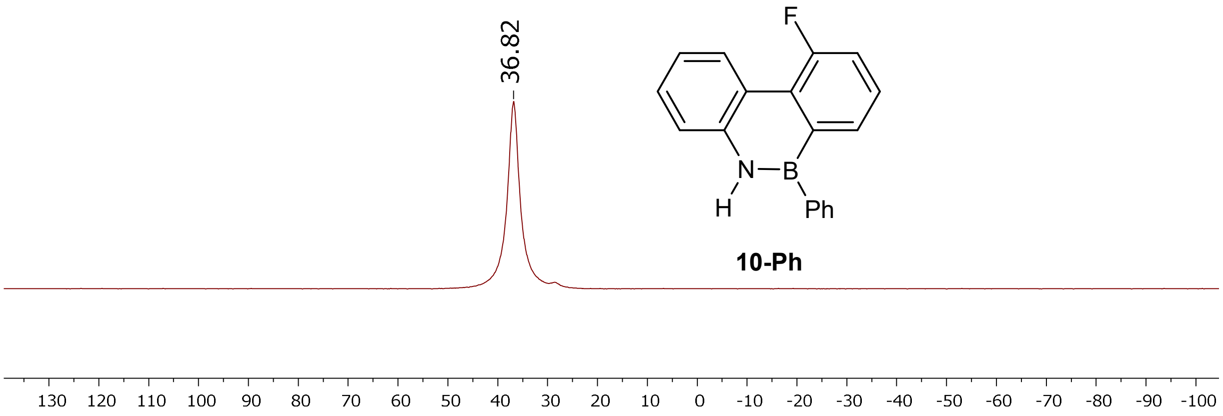

Figure S70. ^{11}B NMR spectrum of **10-Ph** in $CDCl_3$.

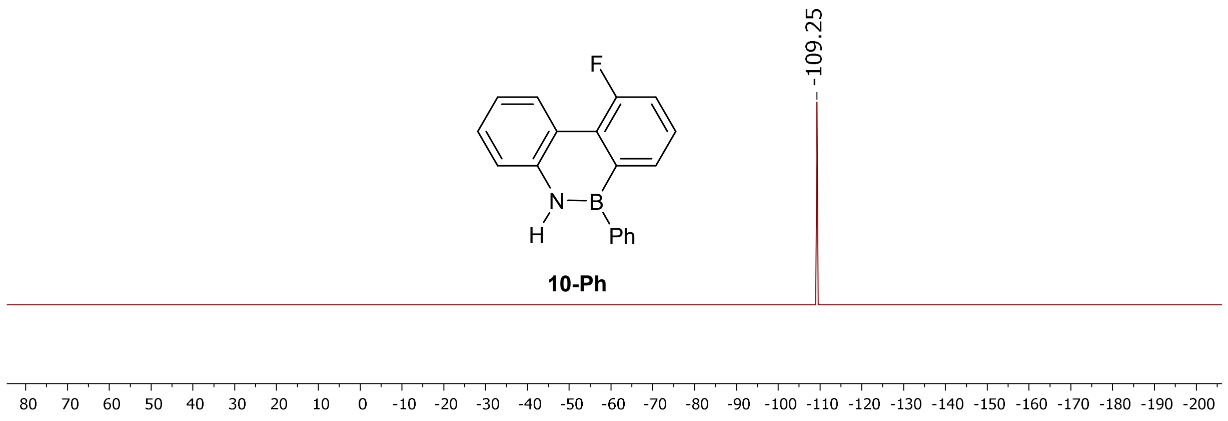

Figure S71. $^{19}F\{^{1}H\}$ NMR spectrum of **10-Ph** in $CDCl_3$.

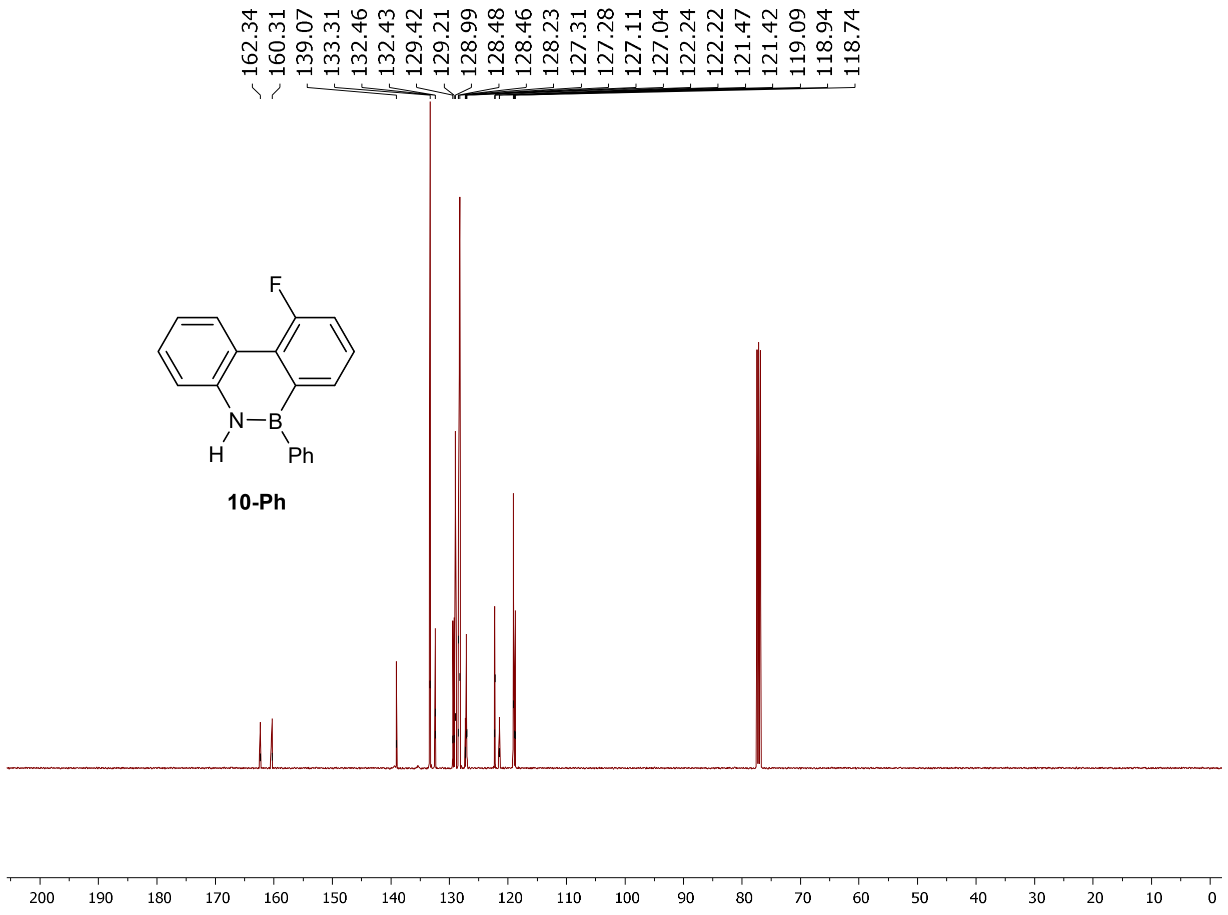

Figure S72. $^{13}C\{^{1}H\}$ NMR spectrum of **10-Ph** in $CDCl_3$.

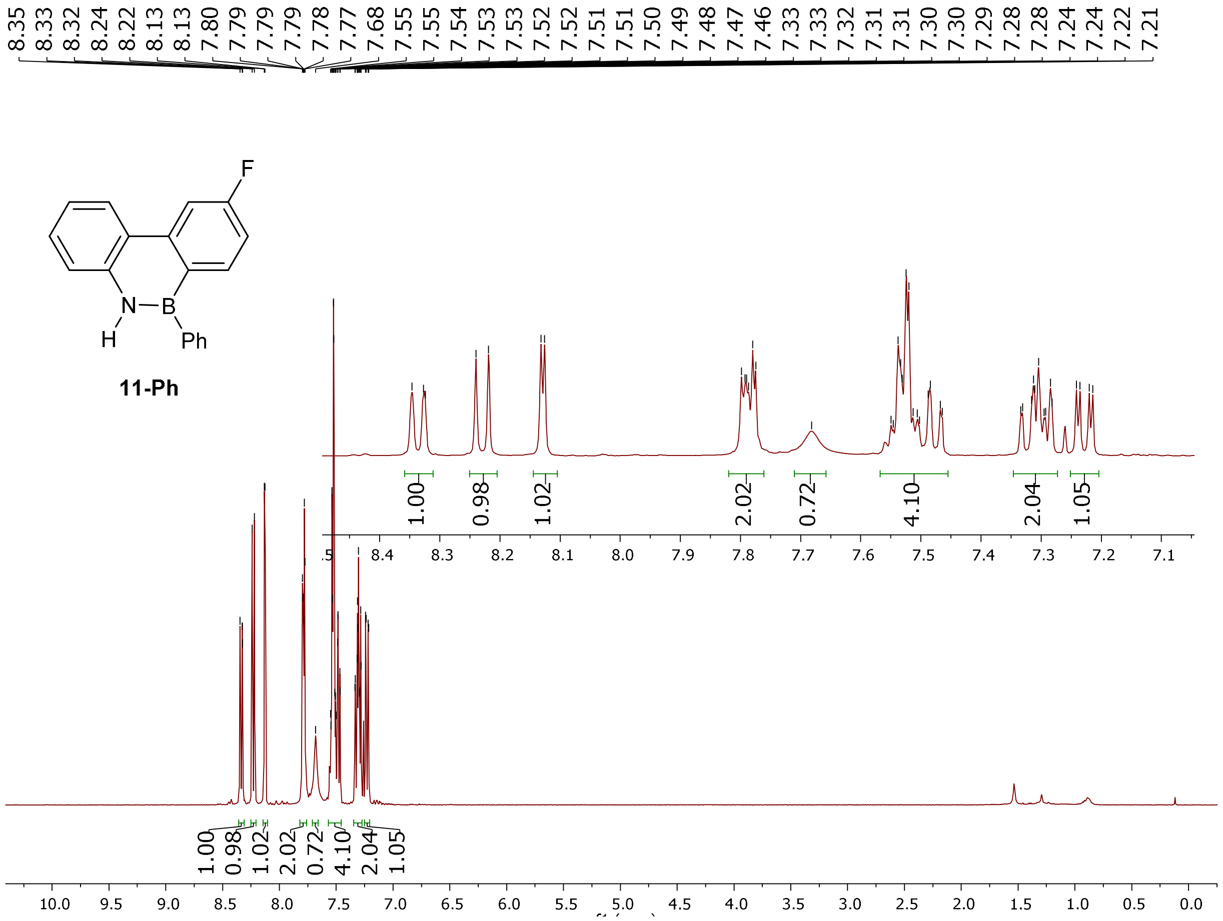

Figure S73. $^{1}H\{^{19}F\}$ NMR spectrum of **11-Ph** in $CDCl_3$.

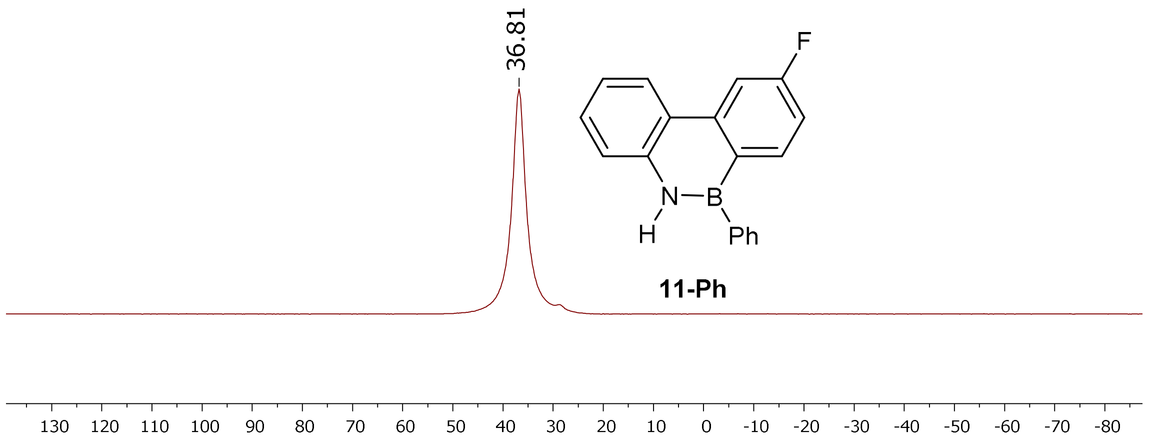

Figure S74. ^{11}B NMR spectrum of **11-Ph** in $CDCl_3$.

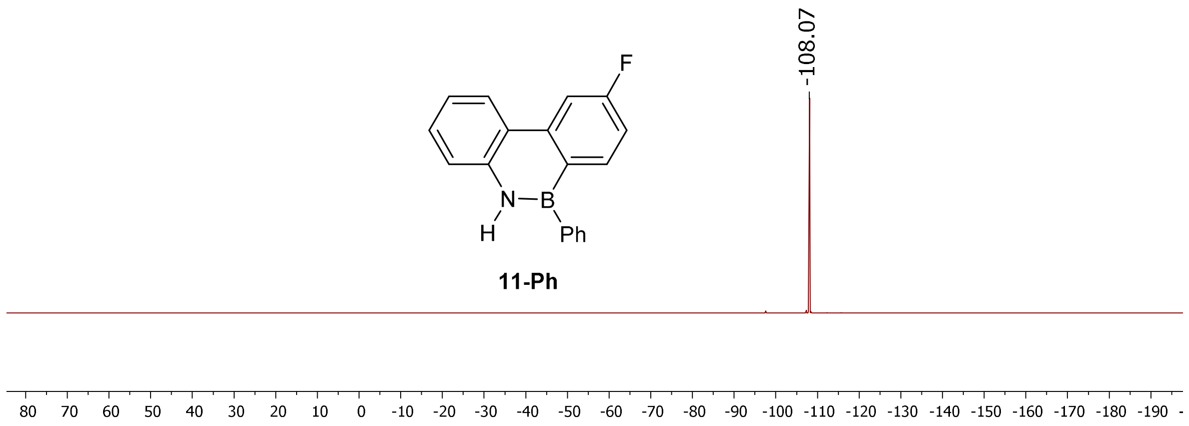

Figure S75. $^{19}F\{^1H\}$ NMR spectrum of **11-Ph** in $CDCl_3$.

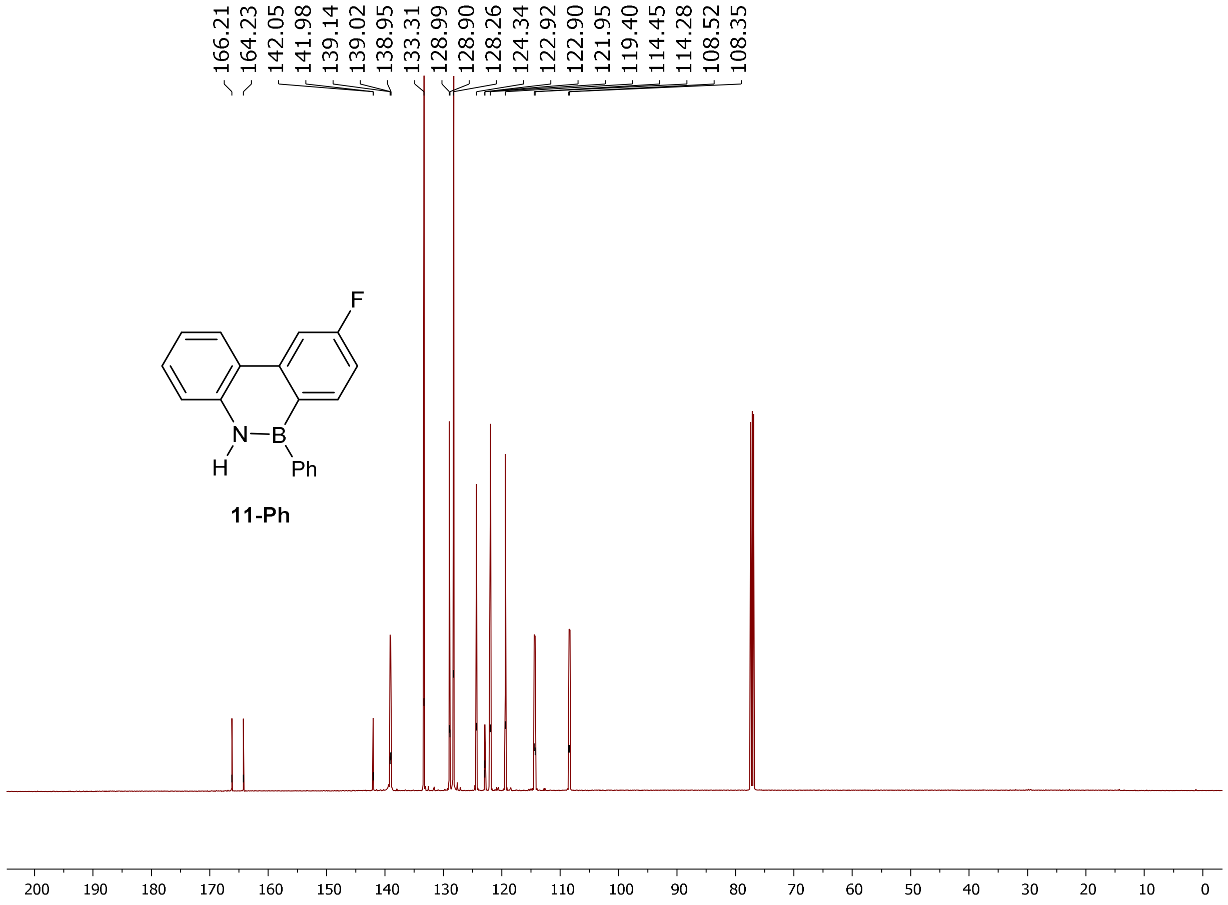

Figure S76. $^{13}C\{^1H\}$ NMR spectrum of **11-Ph** in $CDCl_3$.

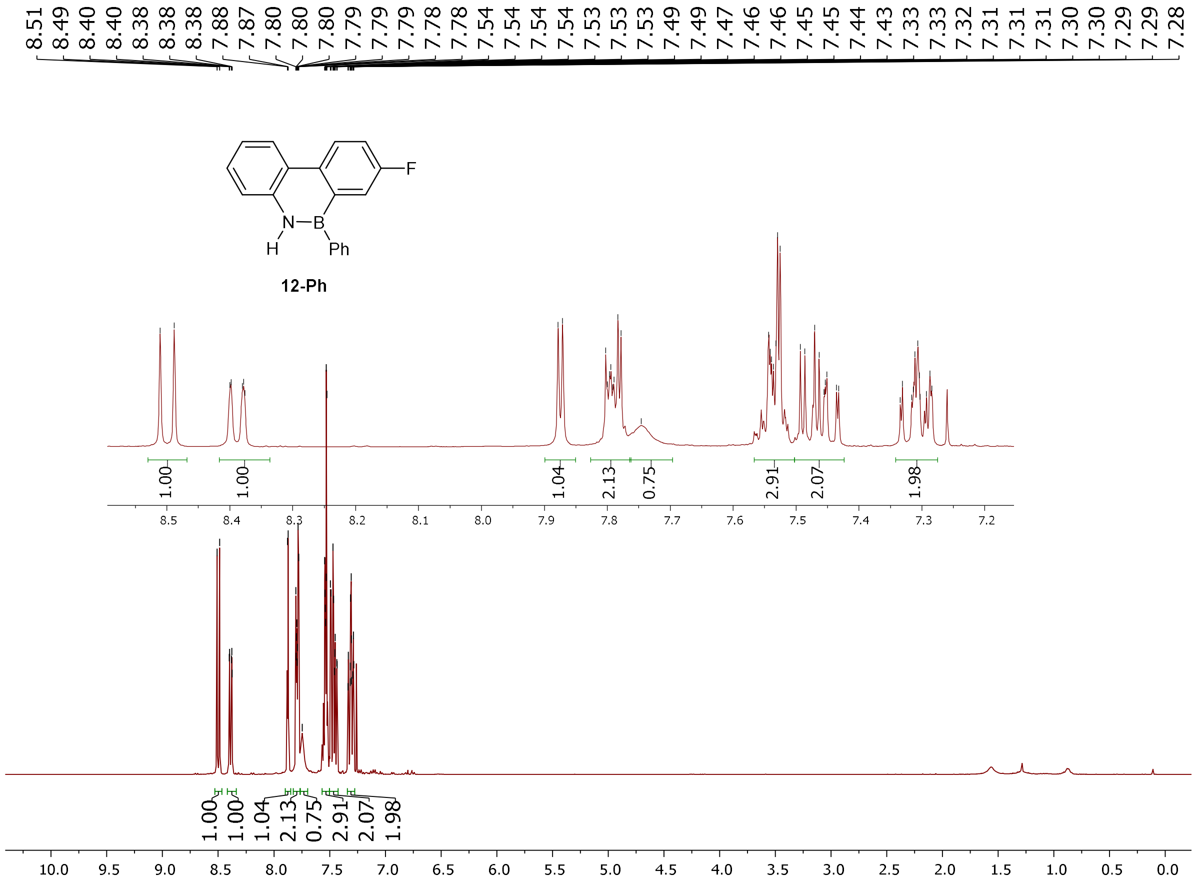

Figure S77. $^{1}H\{^{19}F\}$ NMR spectrum of **12-Ph** in $CDCl_3$.

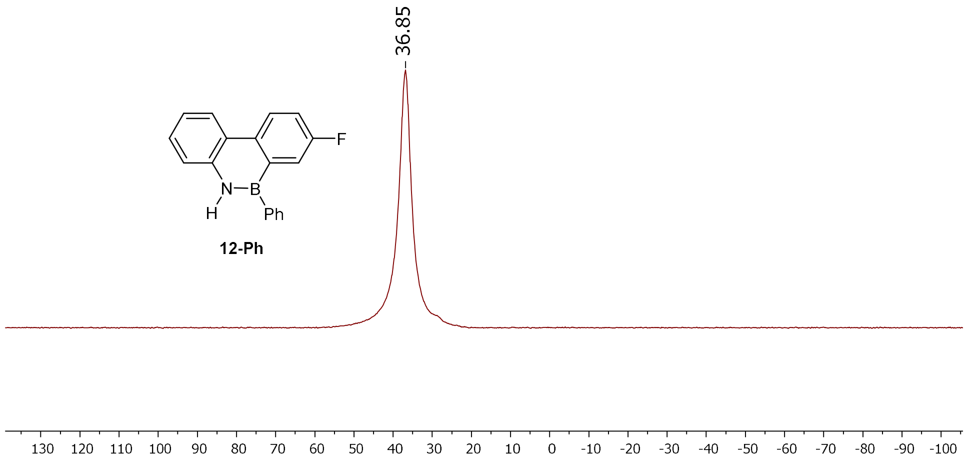

Figure S78. ^{11}B NMR spectrum of **12-Ph** in $CDCl_3$.

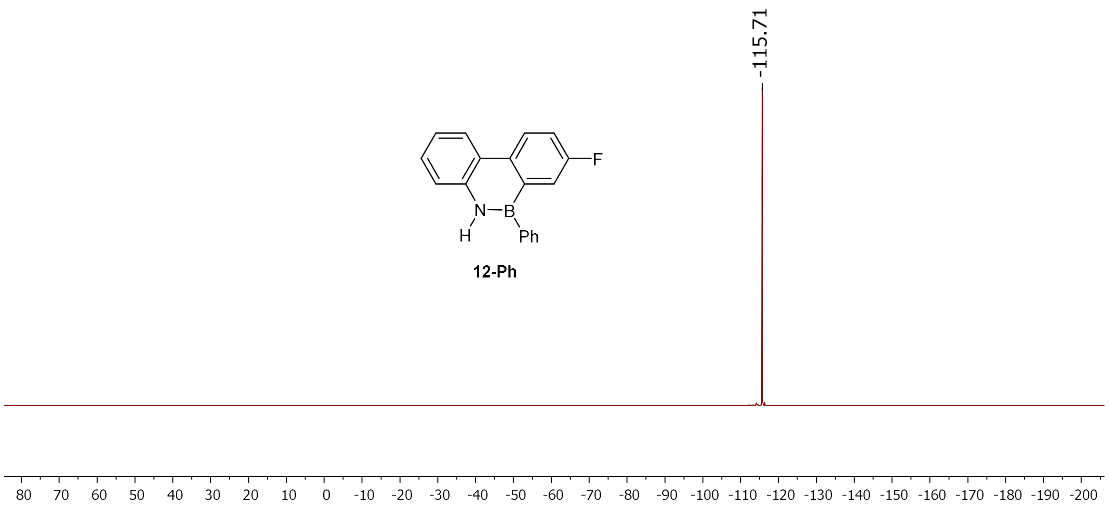

Figure S79. $^{19}F\{^{1}H\}$ NMR spectrum of **12-Ph** in $CDCl_3$.

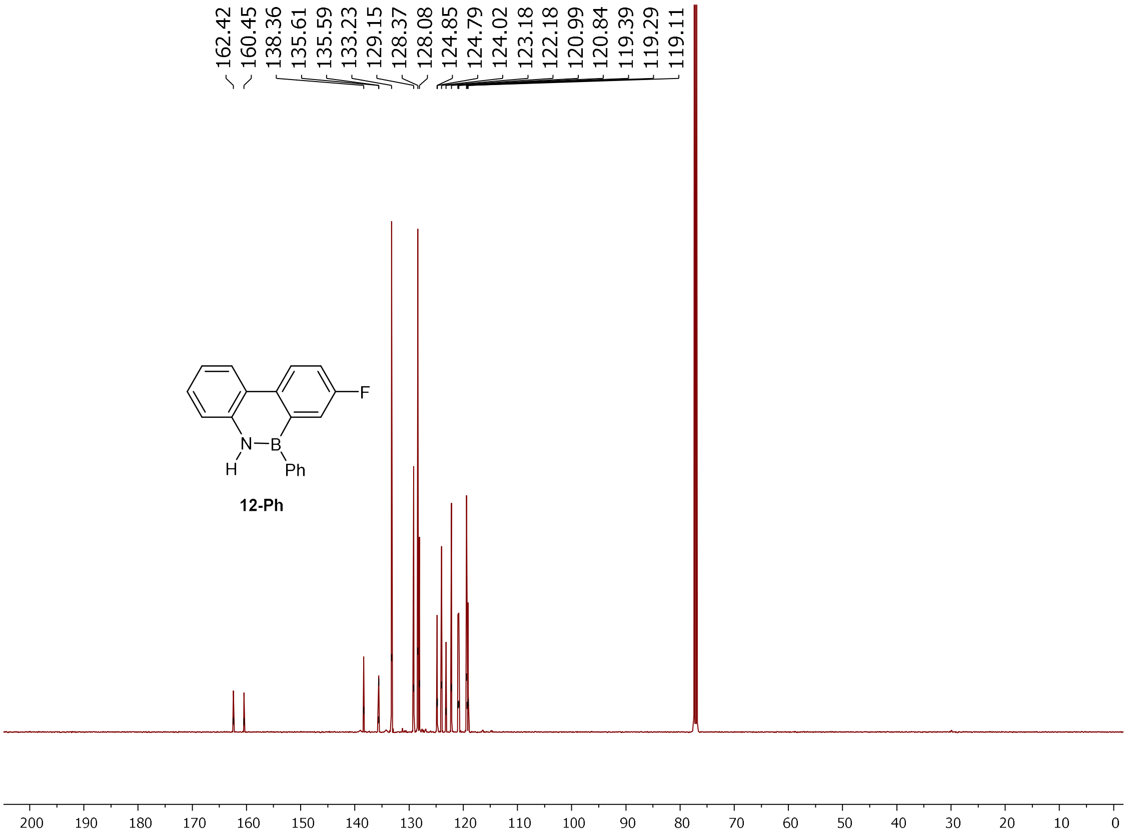

Figure S80. $^{13}C\{^{1}H\}$ NMR spectrum of **12-Ph** in $CDCl_3$.

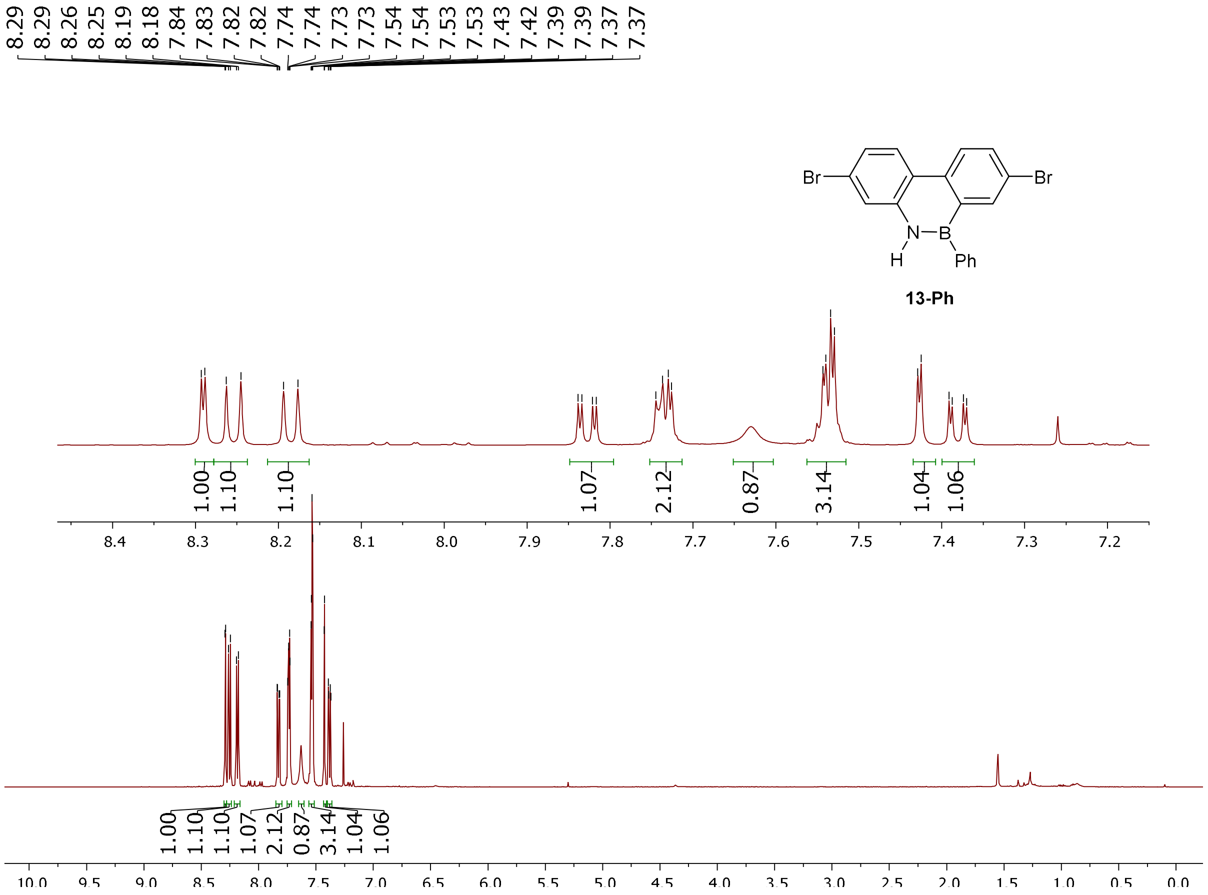

Figure S81. ^{1}H NMR spectrum of **13-Ph** in $CDCl_3$.

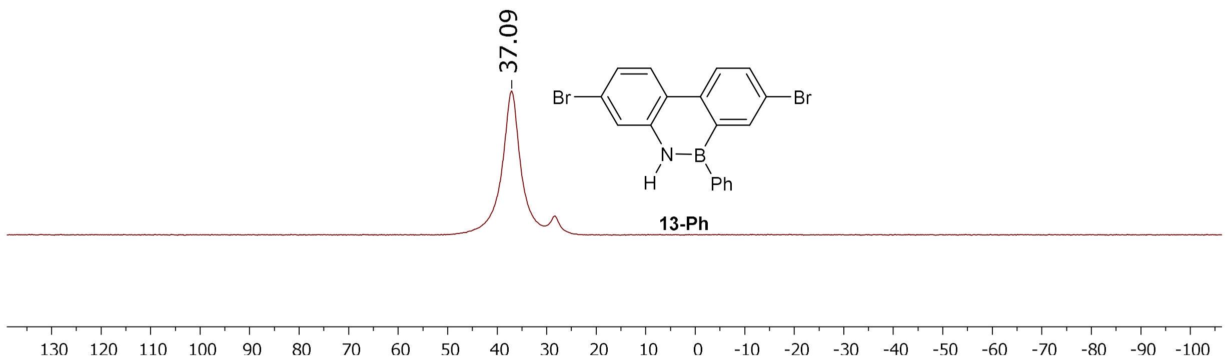

Figure S82. ^{11}B NMR spectrum of **13-Ph** in $CDCl_3$.

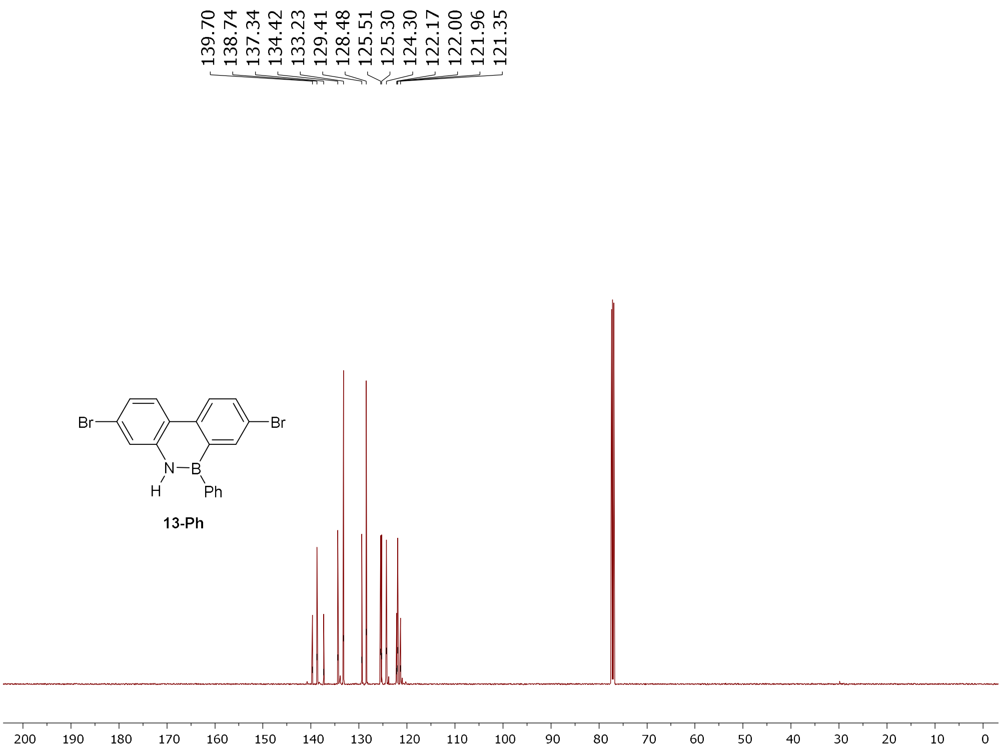

Figure S83. $^{13}C\{^{1}H\}$ NMR spectrum of **13-Ph** in $CDCl_3$.

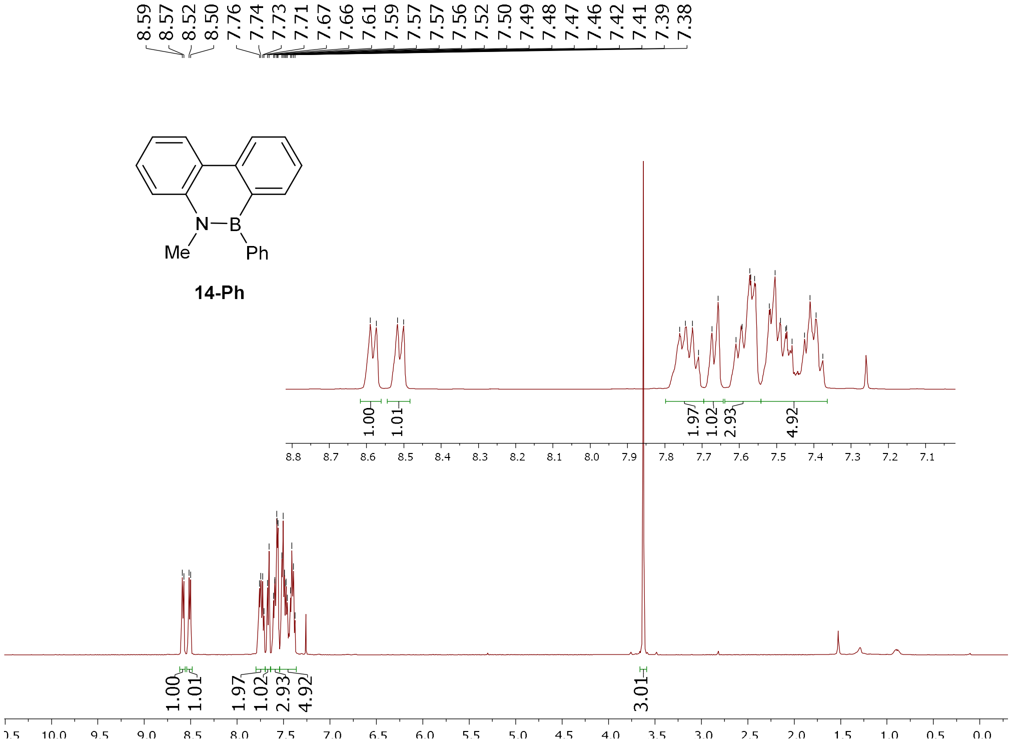

Figure S84. ^{1}H NMR spectrum of **14-Ph** in $CDCl_3$.

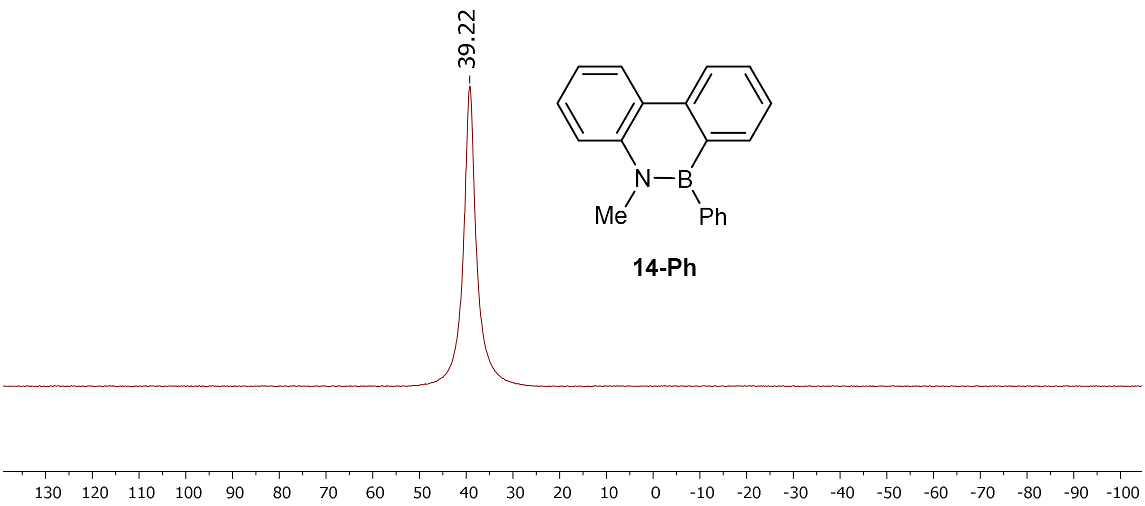

Figure S85. ^{11}B NMR spectrum of **14-Ph** in $CDCl_3$.

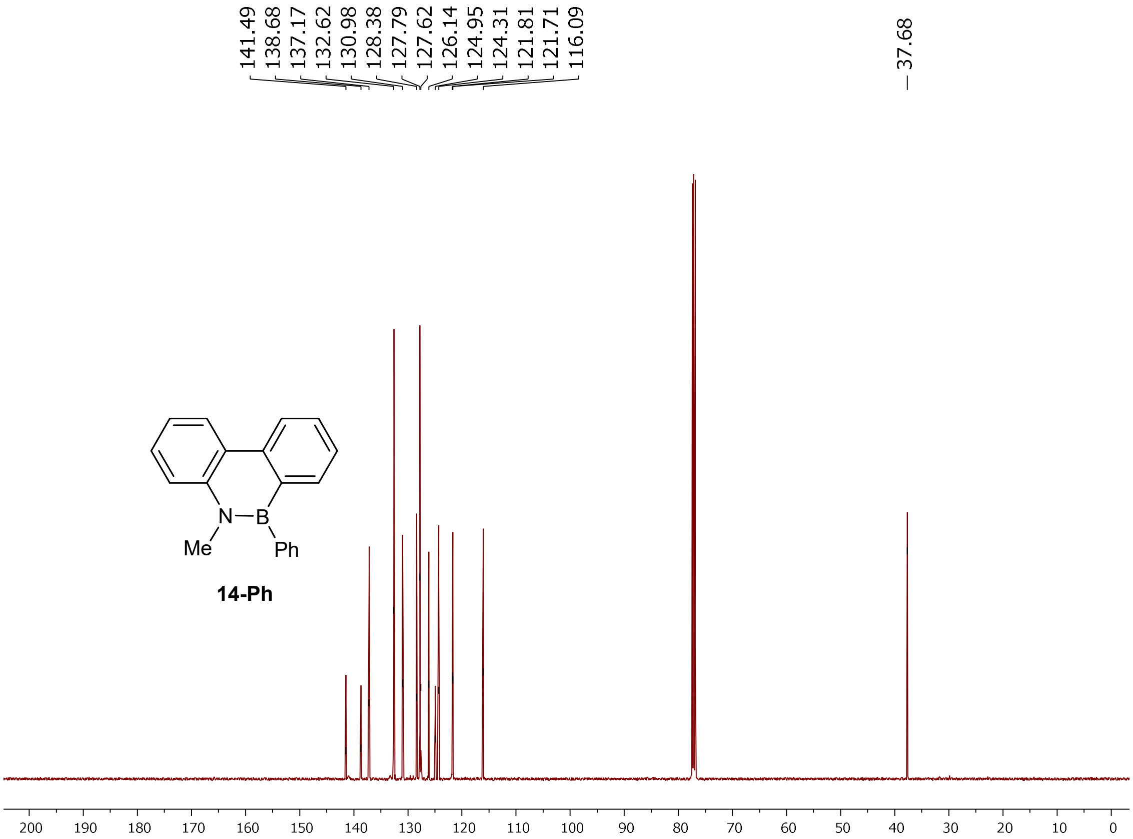

Figure S86. $^{13}C\{^{1}H\}$ NMR spectrum of **14-Ph** in $CDCl_3$.

S8. DFT calculations

All of the calculations were performed using either the Gaussian09 or the Gaussian16 series of programs.[12] For racemisation energy barrier calculations, geometry optimisations were carried out with the PBE0(D3)/6-31G(d) level of theory,[13,14,15] single point energy calculations were performed at the PBE0/def2-TZVP level. This method was chosen to be directly comparable to racemisation energies previously calculated by Merino *et al.*[16]

Strain energy calculations were performed at the B3LYP[17]/6-31g(d) level using the method developed by Itami *et al*,[18] with the functional/basis set chosen to permit direct comparison to previous calculations.

---

[12] (a) Gaussian 09, Revision E.01, Frisch, M. J.; Trucks, G. W.; Schlegel, H. B.; Scuseria, G. E.; Robb, M. A.; Cheeseman, J. R.; Scalmani, G.; Barone, V.; Mennucci, B.; Petersson, G. A.; Nakatsuji, H.; Caricato, M.; Li, X.; Hratchian, H. P.; Izmaylov, A. F.; Bloino, J.; Zheng, G.; Sonnenberg, J. L.; Hada, M.; Ehara, M.; Toyota, K.; Fukuda, R.; Hasegawa, J.; Ishida, M.; Nakajima, T.; Honda, Y.; Kitao, O.; Nakai, H.; Vreven, T.; Montgomery, J. A., Jr.; Peralta, J. E.; Ogliaro, F.; Bearpark, M.; Heyd, J. J.; Brothers, E.; Kudin, K. N.; Staroverov, V. N.; Kobayashi, R.; Normand, J.; Raghavachari, K.; Rendell, A.; Burant, J. C.; Iyengar, S. S.; Tomasi, J.; Cossi, M.; Rega, N.; Millam, J. M.; Klene, M.; Knox, J. E.; Cross, J. B.; Bakken, V.; Adamo, C.; Jaramillo, J.; Gomperts, R.; Stratmann, R. E.; Yazyev, O.; Austin, A. J.; Cammi, R.; Pomelli, C.; Ochterski, J. W.; Martin, R. L.; Morokuma, K.; Zakrzewski, V. G.; Voth, G. A.; Salvador, P.; Dannenberg, J. J.; Dapprich, S.; Daniels, A. D.; Farkas, Ö.; Foresman, J. B.; Ortiz, J. V.; Cioslowski, J.; Fox, D. J. Gaussian, Inc., Wallingford CT, **2009**. (b) Gaussian 16, Revision A.03, M. J. Frisch, G. W. Trucks, H. B. Schlegel, G. E. Scuseria, M. A. Robb, J. R. Cheeseman, G. Scalmani, V. Barone, G. A. Petersson, H. Nakatsuji, X. Li, M. Caricato, A. V. Marenich, J. Bloino, B. G. Janesko, R. Gomperts, B. Mennucci, H. P. Hratchian, J. V. Ortiz, A. F. Izmaylov, J. L. Sonnenberg, D. Williams-Young, F. Ding, F. Lipparini, F. Egidi, J. Goings, B. Peng, A. Petrone, T. Henderson, D. Ranasinghe, V. G. Zakrzewski, J. Gao, N. Rega, G. Zheng, W. Liang, M. Hada, M. Ehara, K. Toyota, R. Fukuda, J. Hasegawa, M. Ishida, T. Nakajima, Y. Honda, O. Kitao, H. Nakai, T. Vreven, K. Throssell, J. A. Montgomery, Jr., J. E. Peralta, F. Ogliaro, M. J. Bearpark, J. J. Heyd, E. N. Brothers, K. N. Kudin, V. N. Staroverov, T. A. Keith, R. Kobayashi, J. Normand, K. Raghavachari, A. P. Rendell, J. C. Burant, S. S. Iyengar, J. Tomasi, M. Cossi, J. M. Millam, M. Klene, C. Adamo, R. Cammi, J. W. Ochterski, R. L. Martin, K. Morokuma, O. Farkas, J. B. Foresman, and D. J. Fox, Gaussian, Inc., Wallingford CT, **2016**.

[13] Petersson, G. A., Bennett, A., Tensfeldt, T. G., Al-Laham, M. A., Shirley, W. A. and Mantzaris, J. *J. Chem. Phys.*, **1988**, *89*, 2193–2218

[14] Adamo, C. and Barone, V. *J. Chem. Phys.,* **1999**, *110*(13), 6158–6170

[15] Grimme, S., Antony, J., Ehrlich, S. and Krieg, H. *J. Chem. Phys.*, **2010**, *132*(15), 1–19.

[16] Barroso, J., Cabellos, J. L., Pan, S., Murillo, F., Zarate, X., Fernandez-Herrera, M. A. and Merino, G. *Chem Commun.*, **2017**, *54*(2), 188–191.

[17] (a) Becke, A. D. *J. Chem. Phys.* **1993**, *98*, 5648-5652; (b) Burke, K; Perdew, J. P.; Yang, W. Electronic Density Functional Theory: Recent Progress and New Directions, (Ed: J. F. Dobson; G. Vignale, M. P. Das), Springer, Heildelberg, **1998**.

[18] Watanabe, K., Segawa, Y. and Itami, K. *Chem. Commun.*, **2020**, *56*(95), 15044–15047.

For reaction energy change calculations, geometry optimisations were performed with the M062X[19]/6-311+G(d,p) level of theory. Solvent effects of the dichloromethane were introduced using the self consistent field approach , by means of the integral equation formalism polarisable continuum model (IEFPCM).[20] For ion pairs, the anions and cations were calculated separately.

For electronic transitions and related calculations, B3LYP as a functional and def2-TZVP[21] as a basis set with Grimme′s D3BJ dispersion correction[22] were used for geometry optimisation. Dichloromethane was used as a solvent for calculations (PCM model). Time-dependent (TD)-DFT calculations were carried out on the optimized structures employing a hybrid exchange-correlation functional using the Coulomb-attenuating method (CAM-B3LYP)[23] with Grimme′s D3BJ dispersion correction and def2-TZVP as a basis set. Dichloromethane was used as a solvent for calculations (PCM model).

Nucleus independent chemical shifts (NICS) were evaluated by using the gauge invariant atomic orbital (GIAO) method[24] at the GIAO-B3LYP/6-311+G(d,p)//B3LYP/6-31G(d) (optimisation) level.

All geometry optimizations were full, with no restrictions. Stationary points located in the potential energy surface were characterized as minima (no imaginary frequencies) or as transition states (one and only one imaginary frequency) by vibrational analysis. The transition state was further confirmed by IRC calculations.

The CD spectrum of **(*M*)-6-Mes** were simulated using the GaussView 5[25] visualization software package. The half-widths of 1600 cm^{-1} were assumed for a proper simulation of the CD spectrum.

---

[19] Zhao, Y. and Truhlar, D. G. *Theor. Chem. Acc.* 2008, **120**, 215-241.

[20] Mennucci B., Cancès E. and Tomasi J., *J. Phys. Chem. B* 1997, **101**, 10506-10517.

[21] a) Schäfer, A., Huber, C. and Ahlrichs, R. *J. Chem. Phys.* 1994, **100**, 5829-5835; b) Weigend, F. and Ahlrichs, R. *Phys. Chem. Chem. Phys.* 2005, **7**, 3297-3305; c) Weigend, F. *Phys. Chem. Chem. Phys.* 2006, **8**, 1057-1065.

[22] Grimme, S., Ehrlich, S. and Goerigk, L. *J. Comput. Chem.* 2011, **32**, 1456-1465.

[23] Yanai, T., Tew, D. P. and Handy, N. C. *Chem. Phys. Lett.* 2004, **393**, 51-57.

[24] a) Dichtfield, R. *Mol. Phys.* 1974, 2**7**, 789-807; b) b) Wolinski, K.; Hinton, J. F. and Pulay, P. *J. Am. Chem. Soc.* 1990, **112**, 8251–8260.

[25] GaussView, Version 5, Dennington, R., Keith, T. and J. Millam, Semichem Inc., Shawnee Mission KS, 2009.

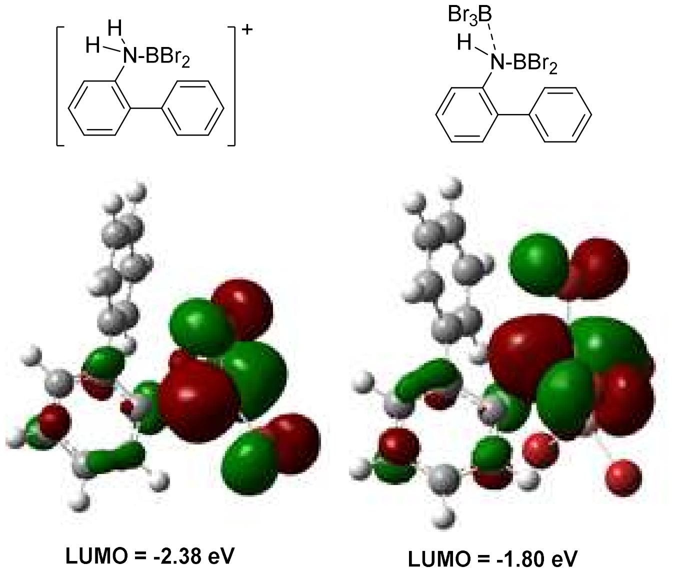

Figure S87. LUMO of, left, borenium cation formed on protonation of an aminoborane, right, a borenium equivalent formed on addition of BBr_3.

Scheme S1. Energy change on protonation with $HNTf_2$ and BBr_3 activation of aminoboranes (kcal/mol).

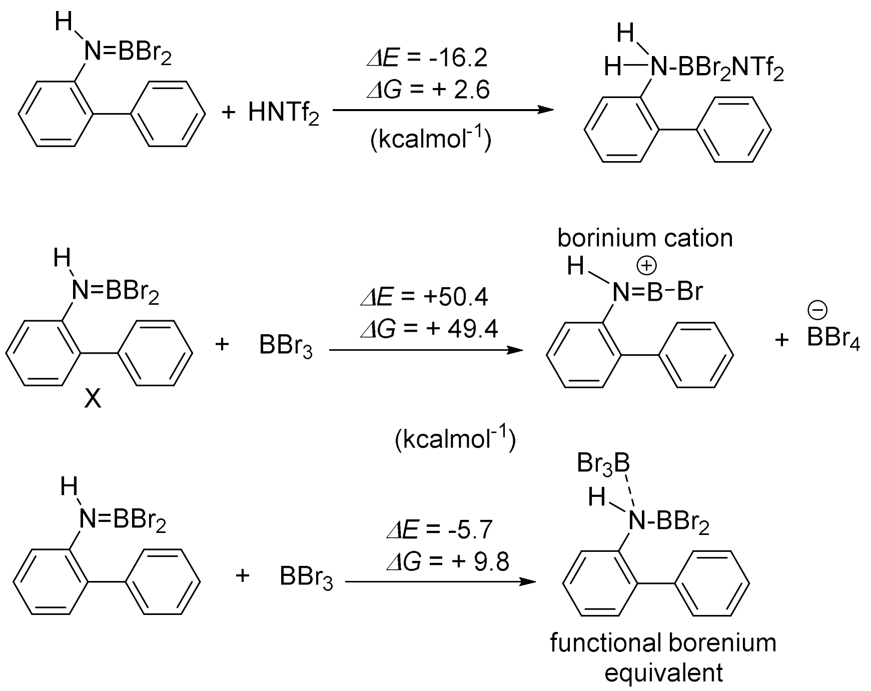

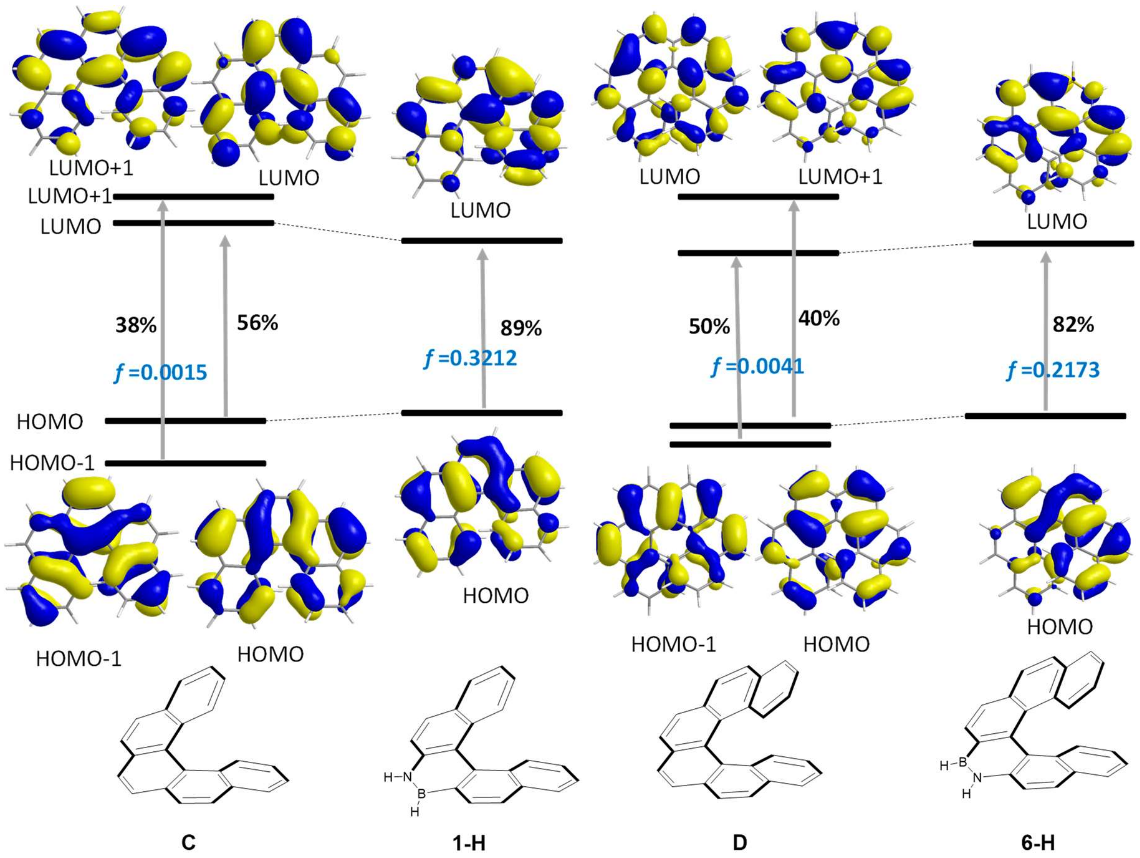

Figure S88. Molecular orbital diagrams (*iso* = 0.03) and the major compositions of $S_0 \rightarrow S_1$ transitions along with the corresponding oscillator strength *f*.

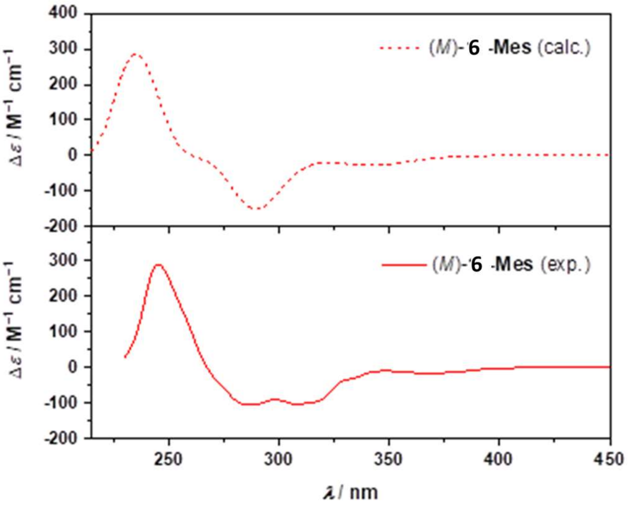

Figure S89. The simulated (dashed line; CAM-B3LYP-D3BJ/def2-TZVP, CH_2Cl_2, PCM model) and experimental (solid line; CH_2Cl_2; $c = 2.5 \times 10^{-5}$ M) ECD spectra of (*M*)-**6-Mes**.

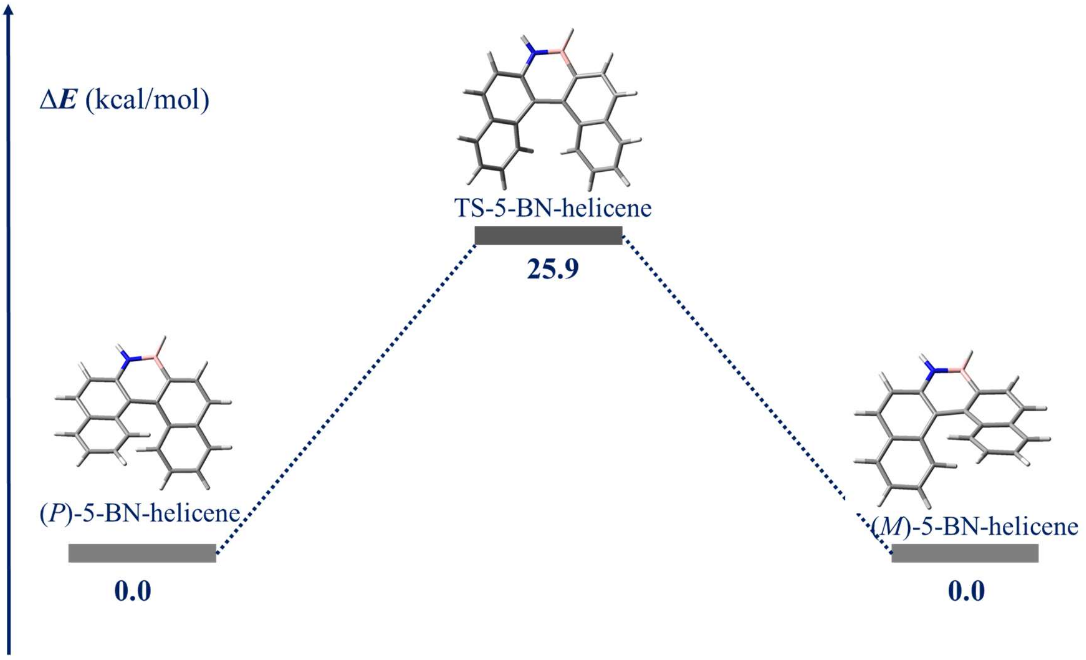

Figure S90. *P*-*M* interconversion pathway and corresponding energy barrier for **[5]-BN-helicene**.

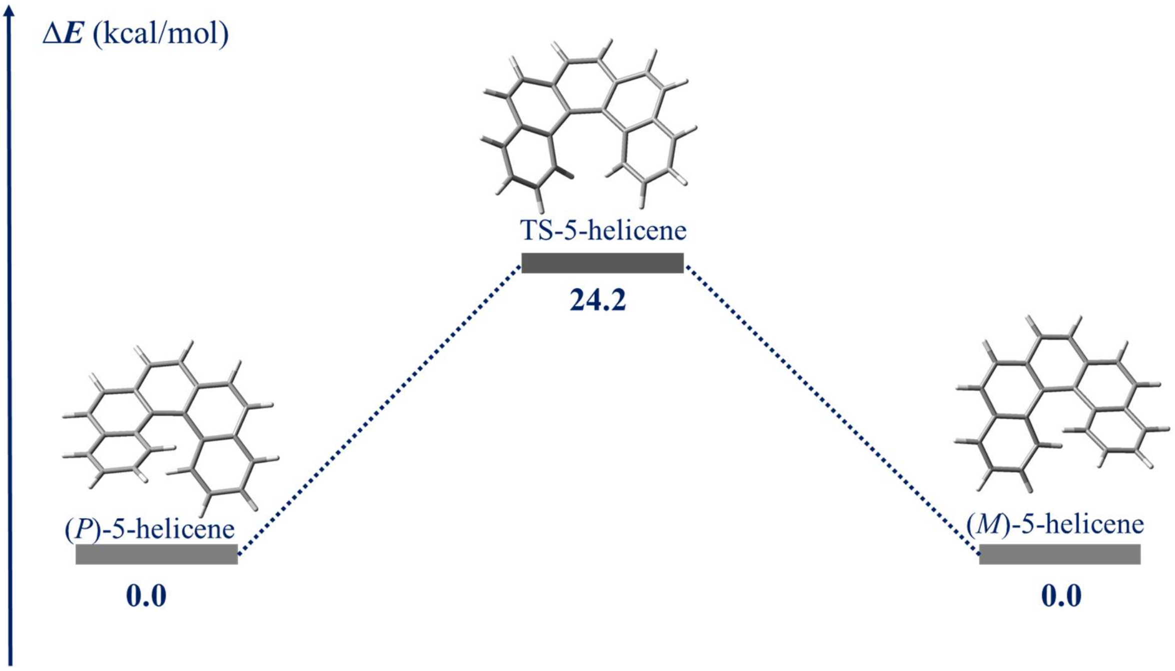

Figure S91. *P*-*M* interconversion pathway and corresponding energy barrier for **[5]-helicene**.

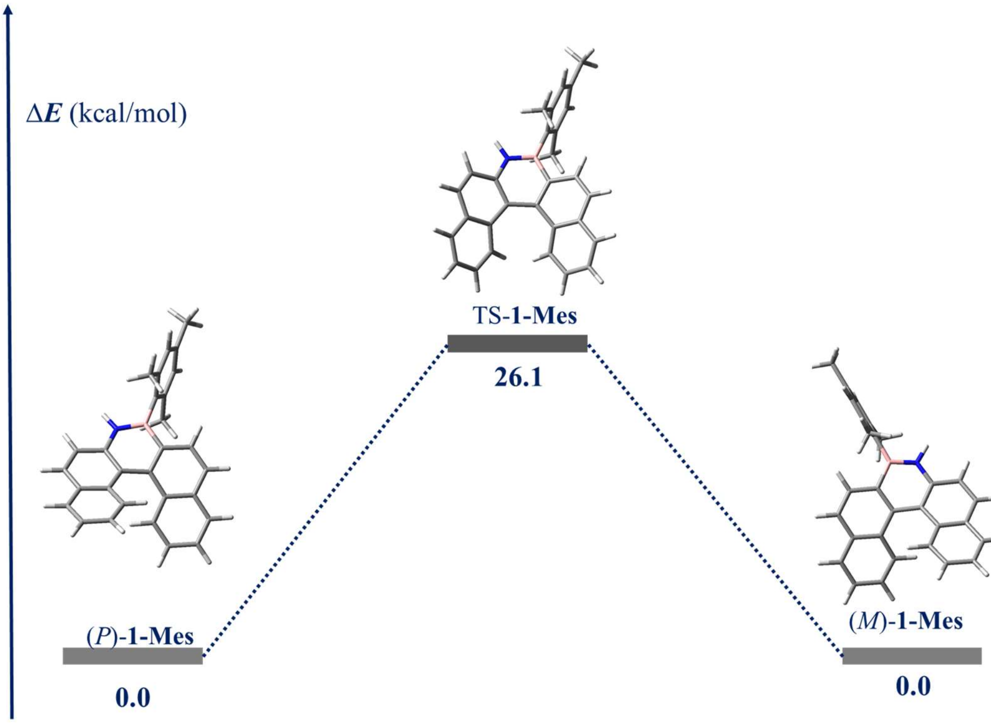

Figure S92. *P*-*M* interconversion pathway and corresponding energy barrier for **1-Mes**.

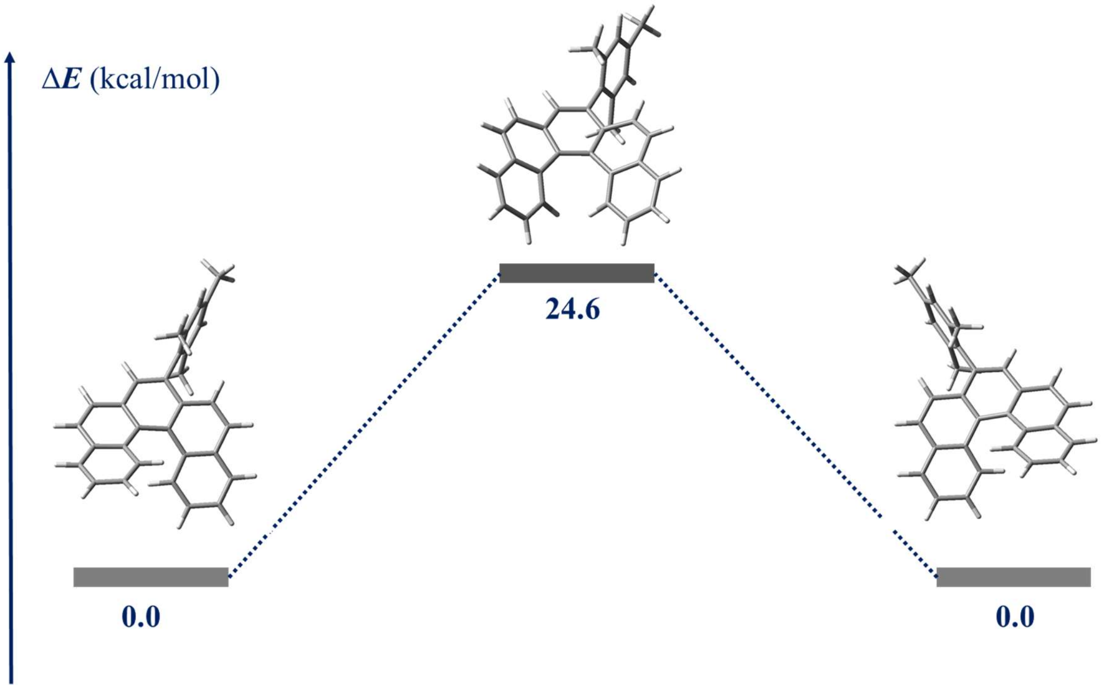

Figure S93. *P*-*M* interconversion pathway and corresponding energy barrier for **[5]-Mes-helicene**.

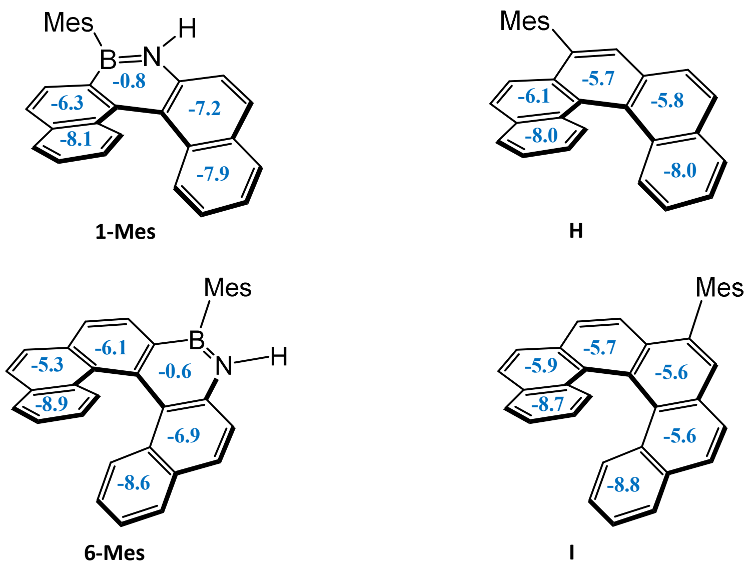

Figure S94. NICS(0) values of **1-Mes**, **6-Mes** and their corresponding carbon analogues.

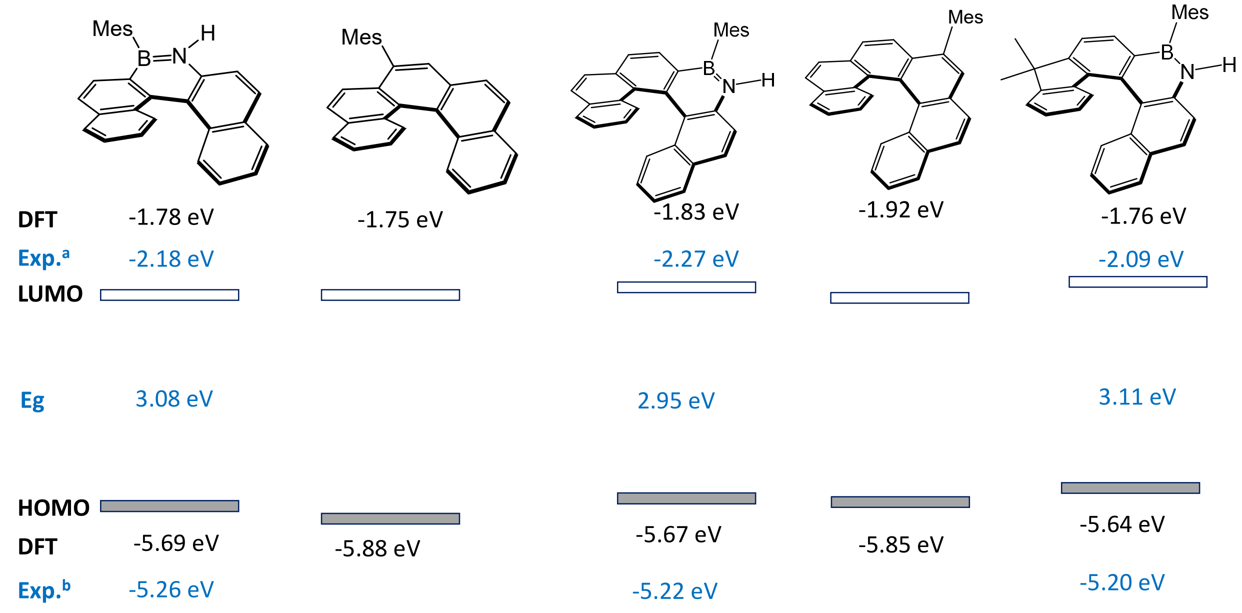

Figure S95. HOMO and LUMO energies of values of **1-Mes**, **6-Mes**, **7-Mes** and the corresponding carbon analogues of **1-Mes** and **6-Mes**. [a.] $E_{LUMO} = -E^{peak} - 5.15$ eV. [b.] The optical energy gap (E_g) was determined at the energy corresponding to the onset of the lowest energy absorption band. [c.] $E_{HOMO} = E_{LUMO} - Eg$.

Scheme S2. Homodesmotic reactions for [5]- and [6]-BN-helicenes and their all carbon analogues (kcal/mol).

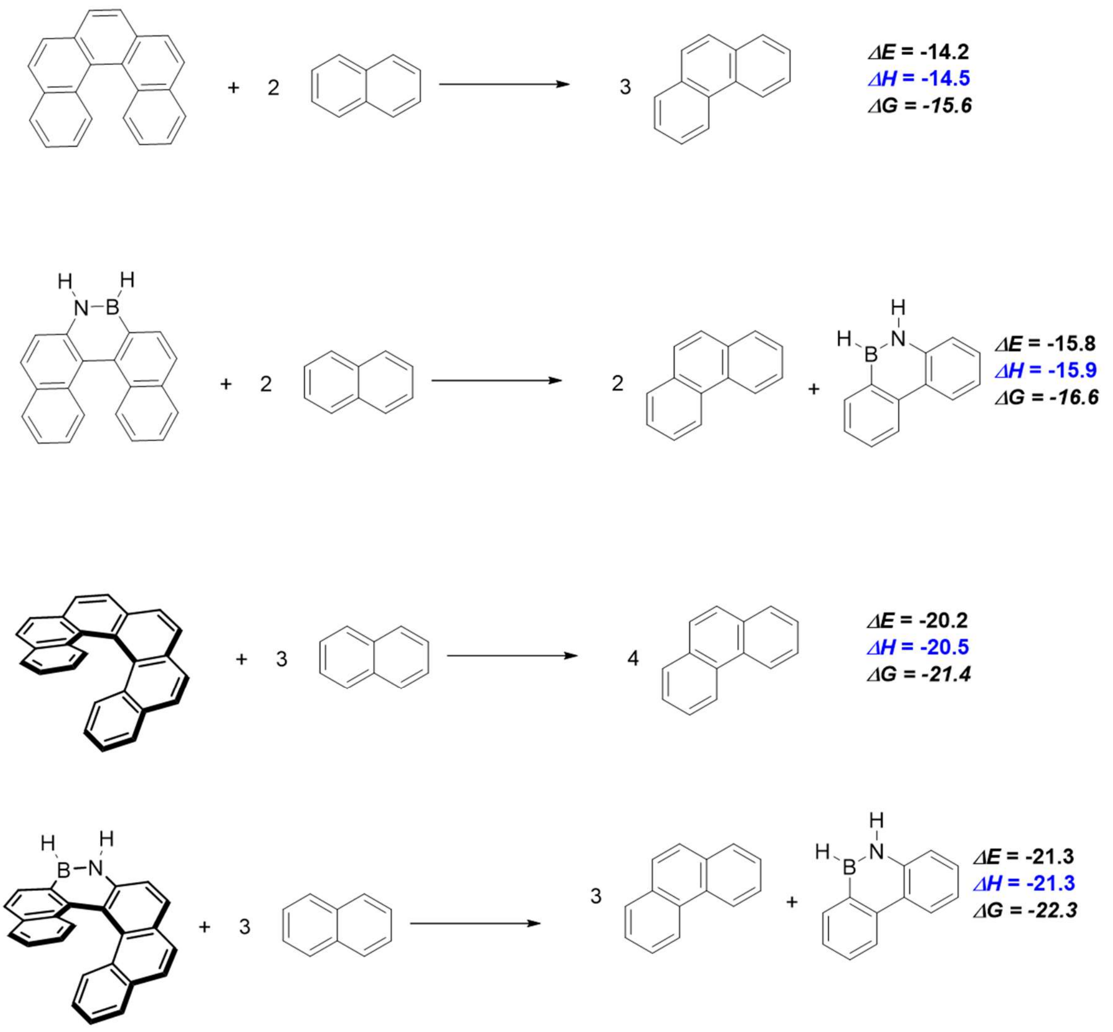

Coordinates

Thermochemistry: M062X/6-311+G(d,p)//PCM(CH_2Cl_2)

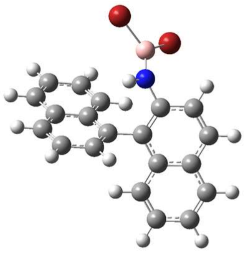

4-Br

H	-0.77340700	0.05190300	-1.79082100
B	-1.85819500	-1.10276200	-0.48639000
N	-0.69074400	-0.70118800	-1.11485300
Br	-3.54626800	-0.37867200	-1.09928100
Br	-1.87213300	-2.33166200	1.00432400
C	0.65602300	-1.02660800	-0.77719200
C	1.54868000	-0.01092200	-0.50398500
C	1.05737000	-2.38383300	-0.76967600
C	2.90223900	-0.34090800	-0.18217700
C	2.34856700	-2.71313700	-0.47021700
H	0.33034500	-3.14606800	-1.02093400
C	3.30086900	-1.70685500	-0.16546500
C	3.86476100	0.64986500	0.15445100
H	2.66314800	-3.75095000	-0.47053300
C	4.64262000	-2.03742500	0.15747500
C	5.15172700	0.29814000	0.47040800
H	3.56938900	1.69196900	0.16549400
C	5.55020400	-1.05926200	0.46676400
H	4.93282900	-3.08276300	0.15886600
H	5.87152400	1.06600600	0.72821900
H	6.57218600	-1.32032300	0.71468500

C	1.09066200	1.40747400	-0.55826800
C	0.18160900	1.91607000	0.42236500
C	1.51273400	2.22308300	-1.58224800
C	-0.28958100	3.25197100	0.29750800
C	-0.27609600	1.13156400	1.51493400
C	1.05592800	3.55713600	-1.69226300
H	2.20277600	1.83062800	-2.32187800
C	-1.21578300	3.75196500	1.25166700
C	0.17018800	4.05788200	-0.77652600
C	-1.17359100	1.63991100	2.41715000
H	0.09584200	0.11986000	1.63233700
H	1.40456200	4.17388400	-2.51180800
C	-1.65420400	2.96457300	2.28293200
H	-1.57164300	4.77115200	1.14519500
H	-0.19576400	5.07563000	-0.85891600
H	-1.51616100	1.02506900	3.24131500
H	-2.36495700	3.35367900	3.00237900

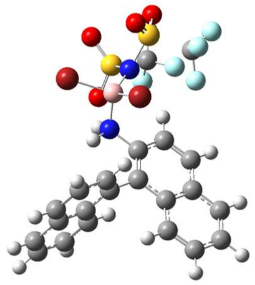

4-Br-HNTf$_2$

C	-2.36886100	0.88179300	-0.11057700
C	-1.07756500	0.69495000	0.31714300
C	-0.25531000	1.75835600	0.74154400
C	-0.74331300	3.03393600	0.71646300
C	-2.06377700	3.29559900	0.27085700
C	-2.88787200	2.21329200	-0.14196400

H	0.75373700	1.56349700	1.07366200
H	-0.12069700	3.86149700	1.03689100
C	-3.23474600	-0.27348000	-0.49360100
C	-3.18953300	-0.78736100	-1.76817500
C	-4.12016600	-0.84177000	0.47689100
C	-4.00163700	-1.88543000	-2.13955600
H	-2.52465800	-0.34140800	-2.49848300
C	-4.93595800	-1.94303200	0.09542000
C	-4.85216200	-2.44938300	-1.22856500
H	-3.94179000	-2.27218800	-3.14936100
H	-5.47652400	-3.29242200	-1.50420100
H	-0.37279000	-0.93664500	1.34856000
H	-1.26872200	-1.30608300	0.02490400
B	0.76607200	-1.05354800	-0.47358400
N	-0.53847000	-0.67127600	0.36877000
Br	0.81674400	-3.07733700	-0.45336100
Br	0.47833300	-0.33441300	-2.31934400
N	2.08686200	-0.47123900	0.16671300
S	3.46994700	-0.21903500	-0.79706900
S	2.44678300	-0.89556400	1.78426700
C	3.19820800	1.53546700	-1.48802500
C	3.25189800	0.67935700	2.49238100
O	3.39082500	-1.96897000	1.89587400
O	3.53343900	-1.12939500	-1.90274000
O	4.56692100	-0.08128500	0.13173100
O	1.15930500	-0.97286500	2.44407600
F	3.08541500	1.47318800	-2.79499000
F	4.27151800	2.23085300	-1.16771600
F	2.13430800	2.10255900	-0.95773700

F	3.09879300	1.69240300	1.65459600
F	4.51739100	0.46754400	2.74244100
F	2.61274300	0.94582200	3.61389800
C	-2.58265800	4.61565500	0.23571300
H	-1.94446400	5.43452700	0.54893700
C	-5.81915300	-2.51246600	1.04952000
H	-6.43491400	-3.35380600	0.75040600
C	-5.89406400	-2.01004700	2.32175100
H	-6.57340700	-2.44922800	3.04247900
C	-3.86474700	4.84928600	-0.18525500
H	-4.25435100	5.85978700	-0.20959200
C	-4.68747500	3.77206000	-0.59056600
H	-5.70055700	3.96827000	-0.92057300
C	-4.21384400	2.48591900	-0.57043100
H	-4.84763500	1.66510300	-0.88460400
C	-5.08525800	-0.91303200	2.70076300
H	-5.15420500	-0.52094300	3.70844200
C	-4.21925700	-0.34307200	1.80377600
H	-3.60543200	0.49943400	2.10256000

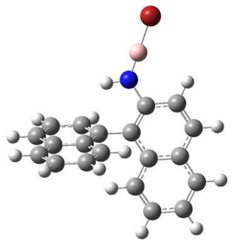

Borinium cation

C	-0.43828500	0.67893800	0.41858700
C	0.90711900	0.85557000	0.20396300
C	1.46854100	2.10167900	-0.15870000
C	0.64862600	3.18013700	-0.31648400

C	-0.75255000	3.06331400	-0.11481700
C	-1.30196300	1.80707900	0.25994800
H	2.53940100	2.19989700	-0.30695100
H	1.06291800	4.14188400	-0.59603900
C	-0.97678600	-0.65723000	0.80244900
C	-1.62622100	-1.47931000	-0.17185700
C	-0.84156500	-1.10031400	2.09856200
C	-2.12972000	-2.74696900	0.23235800
C	-1.34276000	-2.36356500	2.49279800
H	-0.35818900	-0.46243000	2.83125900
C	-1.97313100	-3.16497800	1.58041900
H	-1.22831100	-2.68467500	3.52064400
H	-2.36442100	-4.13289700	1.87436700
H	1.30323100	-1.12636100	0.67677300
B	3.07597600	-0.36335900	0.08225000
N	1.77771300	-0.27963400	0.33971700
Br	4.81725100	-0.48419300	-0.25994300
C	-2.77907700	-3.56994000	-0.72430900
H	-3.15963100	-4.53452000	-0.40641000
C	-2.70245600	1.70576800	0.46589300
H	-3.12826500	0.75295800	0.75762500
C	-2.92310300	-3.15436700	-2.02157800
H	-3.42173000	-3.78820800	-2.74483200
C	-2.42121400	-1.89418200	-2.42375200
H	-2.54060600	-1.57458600	-3.45203800
C	-1.78782400	-1.07631800	-1.52396100
H	-1.40741100	-0.11176800	-1.84080700
C	-3.50968200	2.80200100	0.30062900
H	-4.57745300	2.71324900	0.46066100

C	-2.96277800	4.05033300	-0.07484200
H	-3.61567500	4.90543100	-0.20032300
C	-1.61325800	4.17766300	-0.27593800
H	-1.18297800	5.13114800	-0.56139700

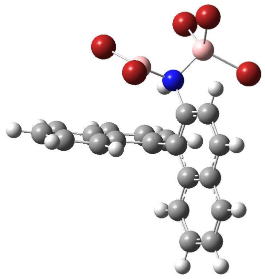

Compound F

C	1.78946400	0.76918600	-0.36711800
C	0.59860600	1.08299600	0.23904400
C	0.43738200	2.21615400	1.06492100
C	1.50826900	3.03521100	1.27849100
C	2.76891300	2.76242800	0.68365800
C	2.91526100	1.62018700	-0.14829500
H	-0.52549500	2.42865600	1.51130800
H	1.40383400	3.91183500	1.90750900
C	1.88756000	-0.46041200	-1.20679400
C	2.15968800	-1.71531100	-0.57805000
C	1.60596500	-0.40697400	-2.55180800
C	2.09355900	-2.89997200	-1.36211300
C	1.58243100	-1.58660200	-3.33461100
H	1.38334800	0.55067000	-3.01086100
C	1.81231500	-2.80261000	-2.75069900
H	1.36079500	-1.51808300	-4.39236500
H	1.77239600	-3.71340400	-3.33826900
H	-0.27948200	-0.38902100	-0.81845600
B	-0.72290900	-0.84773300	1.13160600

N	-0.54758600	0.16940000	0.00594600
Br	-0.38732700	-0.38939200	2.93696100
Br	-1.17726100	-2.62769600	0.67933500
B	-1.95211000	0.85617800	-0.54845600
Br	-1.49241500	2.48266000	-1.62394500
Br	-3.14568700	1.28110900	1.00797900
Br	-2.80653900	-0.51498600	-1.75922200
C	2.45210800	-1.82168300	0.80848700
H	2.53410400	-0.91973100	1.40559200
C	2.63203300	-3.05017200	1.39015600
H	2.84725400	-3.11944300	2.44984400
C	2.53890200	-4.23003000	0.61618700
H	2.67644400	-5.19427800	1.09074800
C	2.28650600	-4.15477400	-0.72935600
H	2.22643500	-5.05550200	-1.33068600
C	3.88895200	3.60315900	0.90458000
H	3.76910400	4.47490500	1.53832000
C	5.09987400	3.31721300	0.32993500
H	5.95079300	3.96440700	0.50529600
C	5.24629800	2.17680700	-0.49314300
H	6.20909000	1.96056200	-0.94040800
C	4.18071100	1.34645500	-0.72887600
H	4.29382100	0.47355100	-1.36132600

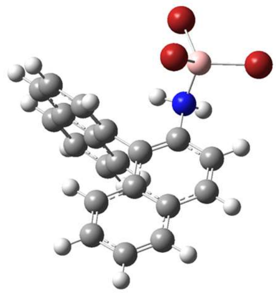

3-Br

C	-1.45478900	-0.60645100	-0.07395700
C	-0.30608300	-0.95130700	-0.74369500
C	-0.11121900	-2.22179400	-1.32510300
C	-1.09615300	-3.16242700	-1.20993400
C	-2.30274700	-2.87324100	-0.52358200
H	0.80889200	-2.44244100	-1.85027200
H	-0.96298000	-4.14483500	-1.64850400
H	1.03102700	0.16562400	-1.83819200
H	0.38985500	0.95346800	-0.56051500
B	2.13422700	-0.15780100	-0.02017100
N	0.76808400	0.04602300	-0.85440700
Br	3.35502800	-1.35453400	-1.08573300
Br	2.91097700	1.70337200	0.12937700
C	-1.62628400	0.75835900	0.50567700
C	-1.93847200	1.86524700	-0.34616200
C	-1.47257500	0.95394900	1.85762600
C	-2.05870900	3.16067500	0.23279900
C	-2.13504900	1.72197200	-1.74760300
C	-1.60596300	2.24064900	2.42756200
H	-1.23869800	0.10648200	2.49161500
C	-2.35427600	4.26757100	-0.60531400
C	-1.88635400	3.31889500	1.63215400
C	-2.41962600	2.81209600	-2.52867100
H	-2.07120400	0.73600000	-2.19506700
H	-1.47618600	2.36564500	3.49557300
C	-2.52720100	4.10046800	-1.95358100
H	-2.44243500	5.25054600	-0.15523000
H	-1.98206700	4.31125800	2.05937300
H	-2.57108000	2.68706200	-3.59429000

H	-2.75322300	4.95180700	-2.58432100
H	-3.17338900	-4.82548800	-0.83337100
H	-3.87794800	-0.31286300	1.12169900
H	-5.62905800	-2.02318600	1.29616200
H	-5.28188800	-4.28799900	0.33380200
C	-2.49239900	-1.58260600	0.04302800
C	-3.33182300	-3.84282300	-0.40261000
C	-4.50143200	-3.54206800	0.24332100
C	-4.69720100	-2.25358700	0.79389500
C	-3.72035300	-1.29707600	0.69764000
Br	1.71727800	-0.92664500	1.77642200

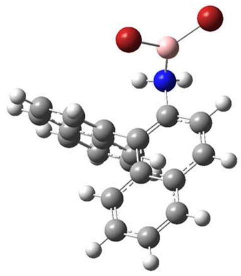

Borenium G

C	0.85258300	0.72154700	-0.02174700
C	-0.34654000	0.72851100	-0.68607900
C	-0.92379200	1.87784000	-1.26192100
C	-0.25566200	3.06551300	-1.15200700
C	0.98987900	3.13811400	-0.47790000
H	-1.86945800	1.81805700	-1.78825400
H	-0.67464700	3.96614500	-1.58516200
H	-1.23691300	-0.80486000	-1.77507400
H	-0.54177800	-1.28994700	-0.35955400
B	-2.46465600	-0.44258900	-0.06243600
N	-1.11285300	-0.54532400	-0.78957100
Br	-4.02111400	-0.43051800	-1.10686400

Br	-2.42642900	-0.32066700	1.80594900
C	1.42079600	-0.53939700	0.53800000
C	2.11201000	-1.44788200	-0.32510300
C	1.29043400	-0.82080100	1.87820800
C	2.63672900	-2.64832900	0.23190400
C	2.30552600	-1.19293700	-1.71041200
C	1.81990100	-2.01224800	2.42417400
H	0.78210000	-0.11389100	2.52435500
C	3.32159800	-3.56036100	-0.61271700
C	2.47327200	-2.90518000	1.61784300
C	2.97486800	-2.09392200	-2.49767400
H	1.93346300	-0.26968700	-2.14103400
H	1.70388800	-2.20974300	3.48251300
C	3.48550600	-3.29230700	-1.94586800
H	3.71685500	-4.47284200	-0.17992800
H	2.88090600	-3.82273600	2.02811800
H	3.12234700	-1.88517800	-3.55048000
H	4.01266900	-3.99268500	-2.58211900
H	1.24847800	5.25974600	-0.78999500
H	3.26065400	1.14864400	1.14916200
H	4.43344500	3.30124400	1.31491700
H	3.42989300	5.36107000	0.35805300
C	1.55744600	1.96009900	0.08450400
C	1.68993600	4.36656600	-0.36277400
C	2.90103500	4.41953900	0.27378000
C	3.47115400	3.24609800	0.82059400
C	2.81921300	2.04426200	0.72947600

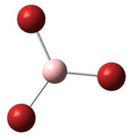

BBr_3

B	0.00000000	0.00000000	0.00172800
Br	0.00000000	0.00000000	1.90493400
Br	0.00000000	1.64583300	-0.95259100
Br	0.00000000	-1.64583300	-0.95259100

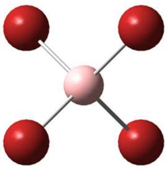

BBr_4

B	0.00000000	0.00000000	0.00000000
Br	1.17182900	1.17182900	1.17182900
Br	-1.17182900	1.17182900	-1.17182900
Br	1.17182900	-1.17182900	-1.17182900
Br	-1.17182900	-1.17182900	1.17182900

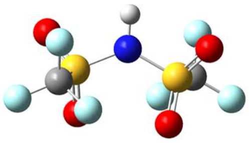

$HNTf_2$

N	-0.00050900	0.00164800	-0.80768700
H	0.00110900	-0.00085000	-1.82803400
S	-1.21972100	-0.88602100	-0.07631400
S	1.22053900	0.88692100	-0.07633900
O	1.66962600	1.83344300	-1.05644900
O	0.81787600	1.24121000	1.25164100
O	-1.66715700	-1.83348400	-1.05635800
O	-0.81691300	-1.23935700	1.25190300

C	2.55460400	-0.40826600	0.07486200
C	-2.55550000	0.40737700	0.07461700
F	2.88229000	-0.83040800	-1.13077800
F	3.59749800	0.14326600	0.66173300
F	2.10478600	-1.41315200	0.79716200
F	-3.59615600	-0.14418800	0.66537200
F	-2.88660500	0.82600500	-1.13127000
F	-2.10544800	1.41467100	0.79350400

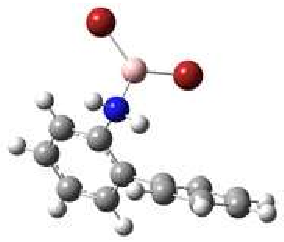

C	1.2537790	1.4846630	0.0085670
C	-0.0465910	1.4307190	-0.4949200
C	-0.9761600	2.4433090	-0.3112740
C	-0.6012420	3.5686250	0.4118300
C	0.6801190	3.6499910	0.9466540
C	1.5935210	2.6222900	0.7448790
H	-1.9743530	2.3591300	-0.7257390
H	-1.3120100	4.3715450	0.5572490
H	0.9733990	4.5224080	1.5172340
H	2.5978680	2.6979850	1.1446230
C	2.2430860	0.3974800	-0.2146040
C	2.8310780	-0.2447310	0.8776260
C	2.5993960	0.0109890	-1.5107950
C	3.7455200	-1.2711780	0.6749110
H	2.5550940	0.0517100	1.8836110
C	3.5109000	-1.0223800	-1.7104720

H	2.1945720	0.5443750	-2.3662910
C	4.0809750	-1.6668060	-0.6178700
H	4.1937890	-1.7671270	1.5272780
H	3.7840090	-1.3100240	-2.7184460
H	4.7923670	-2.4688770	-0.7723120
H	-0.9966590	0.4492240	-2.0630160
H	0.3233490	-0.3525930	-1.4736520
B	-1.3914440	-0.6247190	-0.2521890
N	-0.5040920	0.1963090	-1.2001940
Br	-3.2515030	-0.4722520	-0.4388350
Br	-0.5236920	-1.6924960	1.0195370

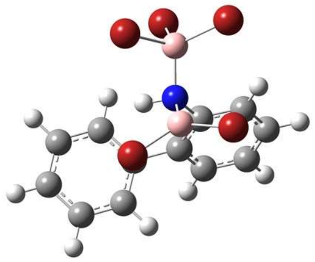

C	-1.6979660	-1.2728020	1.3954120
C	-0.3791580	-0.8037290	1.3448380
C	0.4995740	-0.9740770	2.4060960
C	0.0714830	-1.6464040	3.5427250
C	-1.2310450	-2.1279870	3.6190150
C	-2.1035610	-1.9359500	2.5570280
H	1.5069850	-0.5875490	2.3488440
H	0.7595730	-1.7880800	4.3663580
H	-1.5673680	-2.6518420	4.5052970
H	-3.1198710	-2.3085970	2.6099890
C	-2.6787410	-1.0473320	0.2966960
C	-3.8339010	-0.3056580	0.5582430
C	-2.4651520	-1.5448680	-0.9927630

C	-4.7450970	-0.0429430	-0.4574960
H	-4.0021890	0.0834670	1.5565110
C	-3.3750450	-1.2717670	-2.0108770
H	-1.5975930	-2.1649980	-1.1982030
C	-4.5122840	-0.5161480	-1.7464490
H	-5.6329260	0.5403220	-0.2440370
H	-3.1966940	-1.6603720	-3.0063000
H	-5.2190710	-0.3039100	-2.5395690
H	-0.6471820	-0.2906390	-0.5840670
B	-0.1500560	1.4369740	0.3564950
N	0.0557810	-0.0564740	0.1313590
Br	0.8469430	2.3936930	1.6365870
Br	-1.5434840	2.2772490	-0.6147910
B	1.4167650	-0.5348580	-0.6472580
Br	1.2765590	-2.5295160	-0.8575670
Br	3.1231850	-0.0318500	0.2633910
Br	1.2881600	0.4034210	-2.4377720

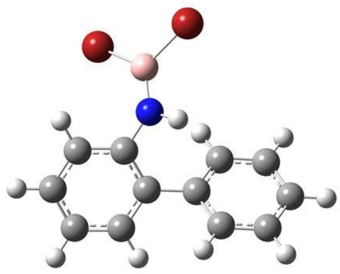

C	1.8625310	1.0936310	-0.1219530
C	0.5226820	1.2450290	-0.5107950
C	-0.0028980	2.5116130	-0.7547190
C	0.7877400	3.6413350	-0.5879610
C	2.1180610	3.5074690	-0.2036190
C	2.6467680	2.2417230	0.0160160

H	-1.0258510	2.6036280	-1.0970070
H	0.3672750	4.6219300	-0.7753750
H	2.7424570	4.3836770	-0.0785240
H	3.6811620	2.1307090	0.3216090
C	2.4644180	-0.2444030	0.1255040
C	1.8891290	-1.1443680	1.0294640
C	3.6446140	-0.6063290	-0.5295000
C	2.4834670	-2.3780650	1.2706900
H	0.9828370	-0.8679800	1.5572140
C	4.2365100	-1.8428300	-0.2910760
H	4.0926200	0.0833960	-1.2367230
C	3.6568390	-2.7316520	0.6089270
H	2.0326120	-3.0621020	1.9800780
H	5.1477620	-2.1121070	-0.8118630
H	4.1167860	-3.6950090	0.7948780
H	0.2126980	-0.6643040	-1.1993160
B	-1.5447900	-0.2188970	-0.2506070
N	-0.2790650	0.0854720	-0.7227990
Br	-2.3046200	-1.9464990	-0.6996520
Br	-2.5677260	0.9555110	0.8868470

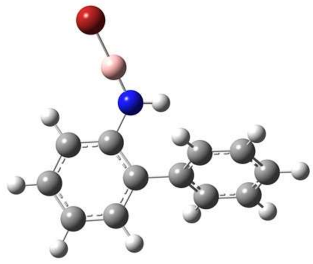

C	-1.3148650	1.0619990	0.0758050
C	0.0608690	1.0401420	-0.1691520
C	0.8321230	2.1967700	-0.1629220
C	0.2283400	3.4145940	0.1158340

C	-1.1414250	3.4693270	0.3552380
C	-1.8999440	2.3062790	0.3252910
H	1.8895090	2.1559310	-0.4039310
H	0.8256160	4.3173660	0.1257540
H	-1.6194800	4.4185710	0.5620620
H	-2.9658220	2.3456070	0.5174300
C	-2.1423790	-0.1717610	0.0551260
C	-3.2670410	-0.2395660	-0.7725500
C	-1.8311730	-1.2619600	0.8750600
C	-4.0603360	-1.3811160	-0.7862870
H	-3.5121000	0.6024030	-1.4104810
C	-2.6272270	-2.4029250	0.8595510
H	-0.9801100	-1.2048810	1.5458430
C	-3.7406380	-2.4650390	0.0271790
H	-4.9275050	-1.4243140	-1.4342080
H	-2.3821650	-3.2378430	1.5048230
H	-4.3601210	-3.3537620	0.0158440
H	0.0612500	-0.9477850	-0.8269750
B	1.9676200	-0.5346610	-0.2831460
N	0.6924260	-0.2226910	-0.4715860
Br	3.6773720	-0.9382750	-0.0076680

Racemisation barrier: PBE0/def2tzvp// PBE0-D3/6-31g(d)

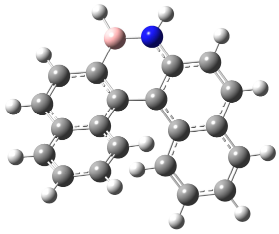

[5]-BN-helicene

C	1.3891250	1.9830950	0.3528680
C	0.7204760	0.7857990	0.0492350
C	-0.7368560	0.8114130	-0.0848440
C	-1.3553390	2.0285170	-0.4277440
C	-1.5851170	-0.3253560	0.1988070
C	-2.9596120	-0.2749260	-0.1845670
C	-3.4983680	0.9206740	-0.7309870
C	-2.7334470	2.0502660	-0.7747720
C	2.7611530	1.9850920	0.7146100
C	3.4958580	0.8381190	0.6725210
C	2.9282660	-0.3549660	0.1547930
C	1.5488040	-0.3717290	-0.2107060
C	3.7343550	-1.4959630	-0.0697350
C	3.2317420	-2.6043470	-0.7055850
C	1.9003290	-2.5886610	-1.1645130
C	1.0859870	-1.5062690	-0.9251030
C	-1.1556910	-1.4486650	0.9503830
C	-1.9984660	-2.5026550	1.2130640
C	-3.3236150	-2.4935250	0.7341180
C	-3.7931490	-1.3933200	0.0598290
H	-1.0384170	4.4298410	-0.3789030
H	1.2789560	3.9970790	0.5422970
H	-4.5436300	0.9362480	-1.0308770

H	-3.1711160	2.9976070	-1.0803800
H	3.2160670	2.9230130	1.0269840
H	4.5413620	0.8409170	0.9703400
H	4.7748740	-1.4634630	0.2457680
H	3.8621230	-3.4707840	-0.8847870
H	1.5114600	-3.4344940	-1.7250250
H	0.0732810	-1.5123800	-1.3100540
H	-0.1458660	-1.4661600	1.3433690
H	-1.6407500	-3.3427580	1.8019470
H	-3.9784720	-3.3386830	0.9284050
H	-4.8289970	-1.3474740	-0.2685770
N	0.7235270	3.1908000	0.2890760
B	-0.5828340	3.3320650	-0.2268140

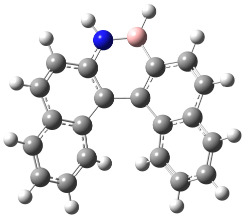

TS-[5]-BN-helicene

C	1.6742160	-0.3802720	-0.2223050
C	1.3758110	2.0248320	-0.3000910
C	3.3822700	1.1460270	0.7291400
H	4.3324550	1.2795570	1.2402420
C	-1.6382490	-0.4173940	-0.2072560
C	-3.3944570	1.0236670	0.7562800
C	-0.7394390	0.7349110	-0.3556300
C	-2.8835250	-0.2713840	0.4883220
C	2.4055110	-2.7097930	-0.5105050
H	2.2591750	-3.6666160	-1.0041790
C	0.7519740	0.7539620	-0.3748090

C	-2.7208190	2.1048590	0.2941470
C	3.7286570	-1.2622730	0.8399310
H	4.6160240	-1.0595820	1.4351780
C	-1.4065340	1.9790160	-0.2353750
C	1.5536010	-1.6650930	-0.7999610
H	0.8410550	-1.8221920	-1.5875440
C	-2.2718000	-2.7697790	-0.5369700
H	-2.0722140	-3.7135450	-1.0373610
C	-1.4552490	-1.6878860	-0.7954060
H	-0.7166230	-1.8037110	-1.5642520
C	-3.6839460	-1.3945480	0.7927870
H	-4.5919870	-1.2314970	1.3689750
C	2.6966100	2.1926590	0.1986590
H	3.1010910	3.2009000	0.2403300
C	2.9022330	-0.1678520	0.4918510
C	-3.3682360	-2.6480690	0.3264060
H	-3.9948460	-3.5046020	0.5573280
C	3.4708190	-2.5313810	0.3833200
H	4.1222630	-3.3605170	0.6448370
H	-1.3939150	3.9774100	-0.5290210
H	-3.1284660	3.1065510	0.4131860
H	-4.3523610	1.1282280	1.2586690
N	-0.7810940	3.1728850	-0.5169270
B	0.6127180	3.3091630	-0.5716240
H	1.1057260	4.3883470	-0.7349970

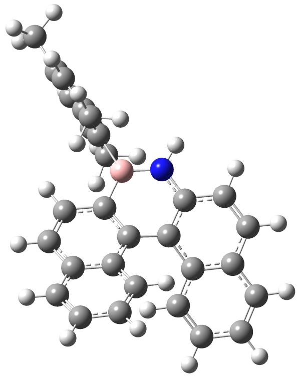

1-Mes

C	0.5879690	-1.7790380	-0.7091730
C	1.4617120	-0.7948860	-0.2202230
C	0.9344620	0.5497940	0.0188990
C	-0.4416720	0.6938120	0.2709230
C	1.7374010	1.7494840	-0.0729900
C	1.1964100	2.9824990	0.4022130
C	-0.1490620	3.0361310	0.8545070
C	-0.9538450	1.9416000	0.7195530
C	1.0759060	-3.0300360	-1.1673990
C	2.3946190	-3.3518540	-1.0449610
C	3.2804640	-2.4853850	-0.3535940
C	2.8035040	-1.2223200	0.1094750
C	4.6023350	-2.8947730	-0.0583160
C	5.4212240	-2.1282050	0.7336530
C	4.9226730	-0.9299530	1.2808040
C	3.6541810	-0.4922830	0.9786650
C	2.9959650	1.7945080	-0.7251100
C	3.7208950	2.9596920	-0.8087000
C	3.2270100	4.1502850	-0.2393080
C	1.9833920	4.1583280	0.3427800
H	-1.3270540	-2.3065090	-1.1231730

H	-0.5399710	3.9791860	1.2296270
H	-2.0146280	2.0034790	0.9522550
H	0.3742270	-3.7288480	-1.6184770
H	2.7672160	-4.3035930	-1.4154140
H	4.9466140	-3.8498420	-0.4490170
H	6.4304420	-2.4571080	0.9650230
H	5.5399070	-0.3474630	1.9594880
H	3.2890150	0.4213620	1.4322790
H	3.3844900	0.8935820	-1.1854260
H	4.6764930	2.9620110	-1.3257950
H	3.8142140	5.0631600	-0.2917870
H	1.5649320	5.0807390	0.7390380
N	-0.7726150	-1.5529240	-0.7368280
B	-1.4022170	-0.4337280	-0.1382020
C	-2.9703580	-0.3714850	-0.0435570
C	-3.6337320	-0.9673000	1.0445890
C	-3.7275920	0.2961170	-1.0235920
C	-5.0240030	-0.8996430	1.1318280
C	-5.1170900	0.3449740	-0.9106980
C	-5.7846540	-0.2512560	0.1590180
H	-5.5267560	-1.3601010	1.9813630
H	-5.6941340	0.8646990	-1.6744890
C	-3.0388600	0.9673100	-2.1834570
H	-2.3766330	1.7709680	-1.8382710
H	-2.4112010	0.2618130	-2.7411860
H	-3.7597860	1.4015300	-2.8830750
C	-2.8401810	-1.6644420	2.1194990
H	-2.2897500	-2.5251170	1.7200320
H	-2.0937570	-0.9921970	2.5608500

H	-3.4852600	-2.0261270	2.9262340
C	-7.2853040	-0.2164560	0.2484070
H	-7.7314250	-1.0799830	-0.2618870
H	-7.6260190	-0.2424250	1.2887450
H	-7.6943990	0.6845700	-0.2205190

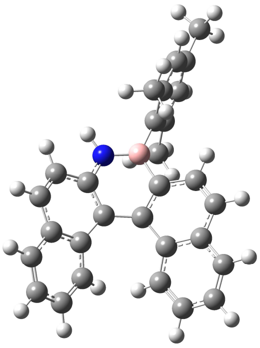

TS-1-Mes

C	-1.6934180	1.8290740	0.0563750
C	0.4454790	0.6940660	-0.0831980
C	0.2068740	2.8456790	-1.1681680
H	0.6033030	3.6665040	-1.7607390
C	-2.8964350	-1.2465060	0.2884980
C	-2.2883960	-3.4282930	-0.6917190
C	-1.4930220	-0.8151030	0.2575010
C	-3.2803120	-2.4817920	-0.3305210
C	-3.5686350	3.3497800	0.5192120
H	-4.4507300	3.5697890	1.1143510
C	-0.9446970	0.5690930	0.1642230
C	-0.9929780	-3.1723360	-0.3863700
C	-1.9216460	4.0254380	-1.0623930
H	-1.4939300	4.7617800	-1.7389240
C	-0.5868410	-1.8851490	0.0622690
C	-2.8612470	2.1922660	0.7656060
H	-3.1641990	1.6074880	1.6135150

C	-5.2642130	-0.9917290	0.8989780
H	-6.0111440	-0.4556440	1.4783350
C	-3.9422380	-0.6068400	0.9893350
H	-3.6992730	0.1448720	1.7150650
C	-4.6406480	-2.8377010	-0.4638450
H	-4.8763160	-3.7602070	-0.9897530
C	1.0029530	1.8501970	-0.6957500
H	2.0769970	1.8672860	-0.8640310
C	-1.1524070	2.8757830	-0.7648520
C	-5.6386870	-2.0832980	0.1046680
H	-6.6815690	-2.3700060	0.0055970
C	-3.1364070	4.2516370	-0.4635710
H	-3.7103030	5.1463450	-0.6881400
H	1.3101810	-2.5761780	0.1742580
H	-0.2188330	-3.9127210	-0.5767090
H	-2.5869860	-4.3739720	-1.1361180
N	0.7729610	-1.7182500	0.1847720
B	1.4147590	-0.4686210	0.1230280
C	2.9839380	-0.3793770	0.1544750
C	3.6513710	0.0195140	1.3270350
C	3.7401060	-0.6782090	-0.9940330
C	5.0434290	0.0951810	1.3412080
C	5.1317480	-0.5872990	-0.9512930
C	5.8027030	-0.2089240	0.2110680
H	5.5497670	0.4051520	2.2543920
H	5.7072150	-0.8120340	-1.8484130
C	2.8602880	0.3700680	2.5601150
H	2.1592060	1.1891850	2.3591240
H	2.2606520	-0.4809060	2.9063420

H	3.5123870	0.6768990	3.3837530
C	3.0508590	-1.0753940	-2.2746390
H	2.5452700	-2.0444710	-2.1807830
H	2.2803660	-0.3466750	-2.5549740
H	3.7604060	-1.1520000	-3.1042970
C	7.3046050	-0.1497330	0.2521060
H	7.6568810	0.6144030	0.9529390
H	7.7286650	-1.1088470	0.5767160
H	7.7247110	0.0742370	-0.7339700

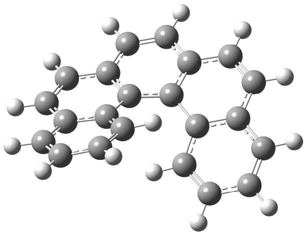

[5]-helicene

C	0.1629870	0.6614480	3.2414740
C	-0.1629870	-0.6614480	3.2414740
C	-0.2339210	-1.3827340	2.0202820
C	-0.0058680	-0.7218330	0.7877230
C	0.0058680	0.7218330	0.7877230
C	0.2339210	1.3827340	2.0202820
C	-0.2906150	1.5581350	-0.3634410
C	-0.0058680	2.9529080	-0.3071210
C	0.4534610	3.5320780	0.9123770
C	0.5000840	2.7851130	2.0458670
C	-0.5000840	-2.7851130	2.0458670
C	-0.4534610	-3.5320780	0.9123770
C	0.0058680	-2.9529080	-0.3071210
C	0.2906150	-1.5581350	-0.3634410
C	0.2673560	-3.7691470	-1.4304550

C	0.8705340	-3.2568730	-2.5552440
C	1.2592930	-1.9056870	-2.5734170
C	0.9779370	-1.0827100	-1.5055960
C	-0.9779370	1.0827100	-1.5055960
C	-1.2592930	1.9056870	-2.5734170
C	-0.8705340	3.2568730	-2.5552440
C	-0.2673560	3.7691470	-1.4304550
H	0.3110530	1.1999190	4.1743390
H	-0.3110530	-1.1999190	4.1743390
H	0.6835320	4.5945410	0.9338480
H	0.7484620	3.2415120	3.0011510
H	-0.7484620	-3.2415120	3.0011510
H	-0.6835320	-4.5945410	0.9338480
H	0.0127490	-4.8250810	-1.3732440
H	1.0789660	-3.8974810	-3.4077440
H	1.7962220	-1.5066870	-3.4296550
H	1.3070490	-0.0506870	-1.5333900
H	-1.3070490	0.0506870	-1.5333900
H	-1.7962220	1.5066870	-3.4296550
H	-1.0789660	3.8974810	-3.4077440
H	-0.0127490	4.8250810	-1.3732440

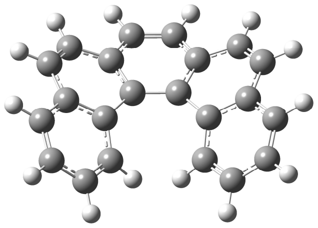

TS-[5]-helicene

C	1.6604560	-0.4070140	-0.2070150
C	1.3785370	2.0053140	-0.2672030
C	3.4047560	1.1013420	0.7011730

H	4.3727420	1.2246420	1.1800520
C	-1.6604540	-0.4070190	-0.2070120
C	-0.6784060	3.2110570	-0.5010700
C	0.6784000	3.2110580	-0.5010630
C	-3.4047640	1.1013380	0.7011510
C	-0.7341110	0.7325410	-0.3443260
C	-2.9042780	-0.2135260	0.4781200
C	2.3528430	-2.7471620	-0.5032950
H	2.1756020	-3.7039450	-0.9868590
C	0.7341090	0.7325440	-0.3443230
C	-2.7191930	2.1562650	0.2069310
C	3.7288810	-1.3110750	0.8018940
H	4.6356570	-1.1171110	1.3704530
C	-1.3785420	2.0053110	-0.2672140
C	1.5087920	-1.6879230	-0.7771200
H	0.7736060	-1.8368300	-1.5439490
C	-2.3528310	-2.7471730	-0.5032710
H	-2.1755870	-3.7039580	-0.9868290
C	-1.5087800	-1.6879340	-0.7771010
H	-0.7735910	-1.8368450	-1.5439270
C	-3.7288810	-1.3110790	0.8018970
H	-4.6356600	-1.1171140	1.3704510
C	2.7191830	2.1562700	0.2069550
H	3.1239880	3.1633940	0.2657690
C	2.9042760	-0.2135220	0.4781250
C	-3.4428200	-2.5818200	0.3573140
H	-4.0913410	-3.4176940	0.6039580
C	3.4428250	-2.5818130	0.3572980
H	4.0913450	-3.4176870	0.6039380

H	1.2401850	4.1396240	-0.5619430
H	-1.2401920	4.1396210	-0.5619530
H	-3.1240000	3.1633900	0.2657380
H	-4.3727530	1.2246390	1.1800240

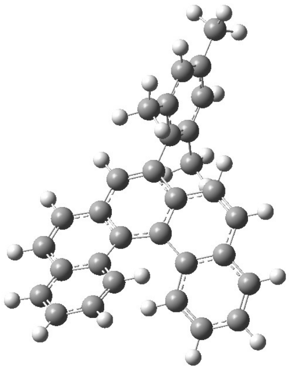

[5]-Mes-helicene

C	-0.62089600	-1.84973500	0.65924800
C	-1.46245700	-0.80308300	0.21383000
C	-0.87147900	0.50221000	0.04482700
C	0.53457100	0.58747900	-0.11595100
C	-1.60687300	1.75368100	0.11853300
C	-0.98521500	2.95920400	-0.31473400
C	0.38460800	2.94059400	-0.70658600
C	1.12824900	1.81480200	-0.54421000
C	-1.18016100	-3.09529200	1.07646800
C	-2.51256600	-3.33781600	0.97194200
C	-3.36120700	-2.40162400	0.31040500
C	-2.83112400	-1.15317100	-0.12586000
C	-4.69824400	-2.74176200	0.00563300
C	-5.47866400	-1.92273400	-0.77648800
C	-4.92507300	-0.74295700	-1.30458000
C	-3.63695000	-0.37224500	-0.98828000
C	-2.87908900	1.86316200	0.72929700

C	-3.54359100	3.06723100	0.80092300
C	-2.96755100	4.23365500	0.26655100
C	-1.70176600	4.17618800	-0.26788100
H	0.84232700	3.86205500	-1.05844500
H	2.19652400	1.82297100	-0.74036900
H	-0.50913000	-3.84650200	1.48619600
H	-2.93717100	-4.27828100	1.31473700
H	-5.08887400	-3.68712500	0.37551700
H	-6.50137900	-2.20156600	-1.01492600
H	-5.51081600	-0.12193800	-1.97679200
H	-3.22492900	0.53007600	-1.42444400
H	-3.33322200	0.98239700	1.16778200
H	-4.51505400	3.11436800	1.28533000
H	-3.50385600	5.17763200	0.30992100
H	-1.21485500	5.07775600	-0.63278900
C	2.84968900	-0.44676900	0.05089400
C	3.46356500	-0.83660400	-1.15055500
C	3.62476400	0.03355300	1.11919100
C	4.85135200	-0.75229400	-1.25919400
C	5.00998700	0.10041900	0.97152700
C	5.64295500	-0.29221800	-0.20739500
H	5.32625400	-1.05196800	-2.19207300
H	5.61055600	0.47373000	1.79950600
C	2.96893200	0.48351700	2.39560900
H	2.26854900	1.30685800	2.21070800
H	2.38728700	-0.32437000	2.85358100
H	3.71179200	0.82501600	3.12240900
C	2.63244000	-1.32240100	-2.30606900
H	2.06286900	-2.21986900	-2.03920600

H	1.89800500	-0.56724500	-2.61106100
H	3.25800400	-1.55877600	-3.17182900
C	7.14053200	-0.24280900	-0.33275200
H	7.59472500	-1.18614500	-0.00321200
H	7.45110500	-0.07728900	-1.36957600
H	7.56848400	0.55604300	0.28170800
H	1.41320900	-2.51190400	0.97530000
C	0.78712800	-1.68097800	0.65709400
C	1.36906500	-0.53719100	0.18390100

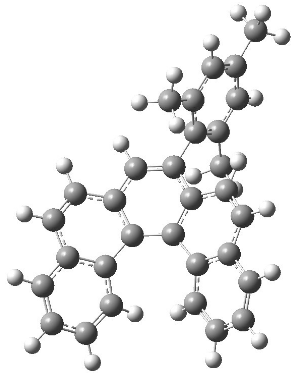

TS-[5]-Mes-helicene

C	-1.55074800	1.82837400	0.02468000
C	0.52951200	0.57013400	-0.07525000
C	0.42198600	2.73746700	-1.15637300
H	0.87760900	3.53701000	-1.73501600
C	-2.92640200	-1.17098100	0.29693500
C	-2.37731400	-3.41153800	-0.61032400
C	-1.49835800	-0.80482400	0.26972400
C	-3.33707700	-2.40118100	-0.31301300
C	-3.35810000	3.43806500	0.45070100
H	-4.24453800	3.69482500	1.02428200
C	-0.88183400	0.52075900	0.15070400
C	-1.08843300	-3.21784300	-0.25105600

C	-1.64043700	4.03596000	-1.08365100
H	-1.15850900	4.75353000	-1.74379300
C	-0.62388000	-1.92350400	0.13988500
C	-2.71167200	2.24437300	0.70832600
H	-3.06168200	1.66976300	1.54358300
C	-5.29615900	-0.81782300	0.84575900
H	-6.03662500	-0.24595200	1.39833200
C	-3.96057200	-0.48429500	0.96634500
H	-3.70933800	0.26532500	1.69202400
C	-4.70473900	-2.70138500	-0.48186500
H	-4.96447400	-3.62157300	-1.00029000
C	1.15544100	1.70259200	-0.68650700
H	2.22580800	1.65408700	-0.86027800
C	-0.94616600	2.84598400	-0.78106800
C	-5.68848600	-1.89969300	0.05119600
H	-6.73856000	-2.14704700	-0.07584600
C	-2.85876500	4.32236200	-0.51121900
H	-3.37960900	5.24715500	-0.74255900
H	1.38964100	-2.68072100	0.30085000
H	-0.35030400	-4.00815800	-0.36189100
H	-2.71605600	-4.35496100	-1.03063000
C	2.85456200	-0.44515900	0.15540300
C	3.50490100	0.02680800	1.30817000
C	3.60008000	-0.80840200	-0.97875700
C	4.89652700	0.10872800	1.31336500
C	4.99061700	-0.70530300	-0.93478000
C	5.65840700	-0.25677600	0.20382700
H	5.39932900	0.47152500	2.20841700
H	5.56719600	-0.97881500	-1.81699600

C	2.71115400	0.44239600	2.51606700
H	2.06982200	1.30303500	2.29191800
H	2.04863300	-0.36265100	2.85358500
H	3.36925900	0.71640400	3.34583100
C	2.91238700	-1.27321200	-2.23385800
H	2.42923100	-2.24704100	-2.09445100
H	2.12341000	-0.57477800	-2.53626800
H	3.62379900	-1.36581700	-3.05982600
C	7.15987600	-0.18906000	0.24303300
H	7.50809800	0.62232400	0.89058500
H	7.58592500	-1.12235500	0.63309000
H	7.58068600	-0.03140000	-0.75532100
C	1.37030100	-0.56164100	0.14673200
C	0.77693300	-1.78366600	0.24531300

Strain energy b3lyp/6-31(d)

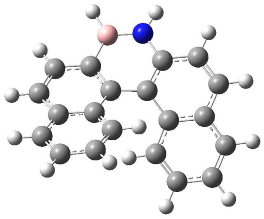

[5]-BN-helicene

C	1.39171300	1.98230300	0.36094700
C	0.72391300	0.77921800	0.05348300
C	-0.74131400	0.80517800	-0.08664600
C	-1.35780900	2.02982300	-0.43211600
C	-1.60103300	-0.33238200	0.19463000
C	-2.98112800	-0.27262300	-0.19151900

C	-3.51128800	0.92803000	-0.74405700
C	-2.73765900	2.05494300	-0.78886300
C	2.76563000	1.99204300	0.72837800
C	3.51010900	0.84803700	0.68431600
C	2.95032500	-0.35074800	0.16146800
C	1.56459700	-0.37862500	-0.20558500
C	3.76960600	-1.48619900	-0.06839900
C	3.27850500	-2.59970500	-0.71122900
C	1.94297000	-2.59708100	-1.16805700
C	1.11443900	-1.52205200	-0.92211600
C	-1.18434900	-1.46415500	0.94872200
C	-2.04053600	-2.51099500	1.21660400
C	-3.36918000	-2.49019800	0.73841500
C	-3.82729200	-1.38556600	0.05705200
H	-1.03774700	4.43013800	-0.38922800
H	1.27838200	4.00334900	0.54325200
H	-4.55536300	0.94969000	-1.04739300
H	-3.17000000	3.00308800	-1.09909900
H	3.21265600	2.93293200	1.04294100
H	4.55519600	0.85737000	0.98304600
H	4.80966100	-1.44385700	0.24710500
H	3.91833600	-3.45828200	-0.89512000
H	1.56267600	-3.44571100	-1.73043800
H	0.10238200	-1.54168100	-1.30500600
H	-0.17601000	-1.49453000	1.34150600
H	-1.69108900	-3.35315300	1.80773600
H	-4.03267600	-3.32774100	0.93661200
H	-4.86225000	-1.33050900	-0.27239500
N	0.72154500	3.19540400	0.29115400

B	-0.58525600	3.33511700	-0.23083400

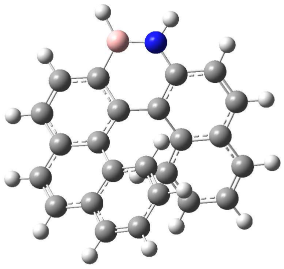

[6]-BN-helicene

C	-2.83174800	-1.17584600	0.25117300
C	-1.58029800	-0.53145900	0.52257100
C	-1.28990600	0.76202500	-0.07379900
C	-2.37524300	1.44772500	-0.65339600
C	-3.60495200	0.78786700	-0.92989800
C	-3.81023600	-0.50206300	-0.53233400
C	0.01465900	1.44494700	0.02026600
C	-0.00289300	2.86471100	0.07998000
C	1.29940000	0.78036800	0.06527600
C	2.39259700	1.50218600	0.63209500
C	2.28080200	2.89145300	0.89427300
C	1.14647100	3.56659200	0.52046200
C	-3.11273200	-2.44825200	0.81124000
C	-2.22569700	-3.05971300	1.66815100
C	-1.02756800	-2.39608800	2.00585300
C	-0.71444700	-1.17410500	1.44727200
C	1.59271200	-0.52716000	-0.51938700
C	2.84869700	-1.15259800	-0.24412900
C	3.83441300	-0.45811200	0.52874200
C	3.63277900	0.83309900	0.90179500
C	0.73492100	-1.17755100	-1.44031400

C	3.14202600	-2.42231700	-0.79560100
C	1.05742800	-2.40260700	-1.99365500
C	2.25949600	-3.04917500	-1.65113500
H	-4.38246500	1.33702400	-1.45710100
H	-4.74648600	-1.00659500	-0.75696000
H	-3.09325100	3.20335600	-1.38940000
H	3.13559500	3.41456600	1.31613600
H	1.10219600	4.65084000	0.58562700
H	-4.06256600	-2.91985800	0.56928500
H	-2.45568500	-4.02911800	2.10149800
H	-0.34329800	-2.84684700	2.71968900
H	0.20296000	-0.68313600	1.74405700
H	4.77319700	-0.96083200	0.74855400
H	4.41440300	1.39290800	1.40955200
H	-0.18681400	-0.69546300	-1.73891600
H	4.09685900	-2.88346200	-0.55348300
H	0.37649800	-2.86439000	-2.70353000
H	2.50110500	-4.01892400	-2.07765000
B	-1.21520200	3.61323200	-0.48007600
N	-2.27764300	2.80306300	-0.94139900
H	-1.27683600	4.79876500	-0.62019000

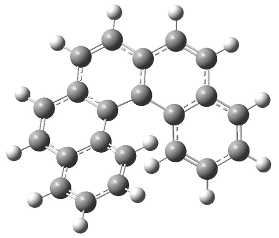

[5]-helicene

C	0.16490700	0.66232900	3.24472300
C	-0.16490700	-0.66232900	3.24472300

C	-0.23843900	-1.38616700	2.02093700
C	-0.00734000	-0.72568400	0.78144700
C	0.00734000	0.72568400	0.78144700
C	0.23843900	1.38616700	2.02093700
C	-0.29036400	1.57347700	-0.37006900
C	-0.00734000	2.97482400	-0.30356300
C	0.45698700	3.54733700	0.92132600
C	0.50841100	2.79162600	2.05206300
C	-0.50841100	-2.79162600	2.05206300
C	-0.45698700	-3.54733700	0.92132600
C	0.00734000	-2.97482400	-0.30356300
C	0.29036400	-1.57347700	-0.37006900
C	0.27565200	-3.80277500	-1.42165700
C	0.88446000	-3.30005600	-2.55166000
C	1.26802100	-1.94432700	-2.58223000
C	0.97854100	-1.10878400	-1.52133700
C	-0.97854100	1.10878400	-1.52133700
C	-1.26802100	1.94432700	-2.58223000
C	-0.88446000	3.30005600	-2.55166000
C	-0.27565200	3.80277500	-1.42165700
H	0.31413300	1.20038100	4.17748900
H	-0.31413300	-1.20038100	4.17748900
H	0.68650400	4.60966600	0.94890300
H	0.76010000	3.24216700	3.00911700
H	-0.76010000	-3.24216700	3.00911700
H	-0.68650400	-4.60966600	0.94890300
H	0.02305700	-4.85852000	-1.35489600
H	1.09866300	-3.94881600	-3.39658200
H	1.80570000	-1.55194600	-3.44118400

H	1.30378500	-0.07722100	-1.56272400
H	-1.30378500	0.07722100	-1.56272400
H	-1.80570000	1.55194600	-3.44118400
H	-1.09866300	3.94881600	-3.39658200
H	-0.02305700	4.85852000	-1.35489600

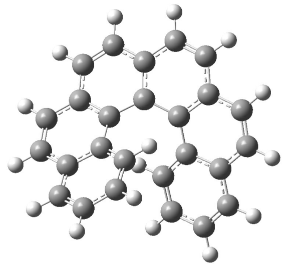

[6]-helicene

C	-2.85248600	-1.16636200	0.20856300
C	-1.59471400	-0.54682400	0.49583800
C	-1.29618500	0.76421700	-0.07045000
C	-2.38608200	1.49546000	-0.61779200
C	-3.62810000	0.84082200	-0.89802600
C	-3.83525900	-0.45884800	-0.55164800
C	0.00011300	1.42167400	0.00005800
C	0.00030400	2.85080100	-0.00014000
C	1.29622300	0.76410300	0.07048200
C	2.38620900	1.49530300	0.61790700
C	2.27311000	2.89884000	0.83591600
C	1.15155100	3.56631300	0.43816100
C	-3.14670200	-2.44587900	0.73956000
C	-2.26748900	-3.08491000	1.58789800
C	-1.06644400	-2.44255700	1.94662300
C	-0.74071700	-1.21140000	1.41187500
C	1.59457600	-0.54703500	-0.49587000
C	2.85228900	-1.16663900	-0.20857300

C	3.83512500	-0.45920200	0.55173100
C	3.62812900	0.84040300	0.89827100
C	0.74055500	-1.21153100	-1.41190900
C	3.14643100	-2.44612100	-0.73956300
C	1.06620400	-2.44275300	-1.94666800
C	2.26718200	-3.08511500	-1.58795500
H	-4.40853900	1.41481000	-1.39173200
H	-4.77539300	-0.95415400	-0.78162300
H	-3.12770200	3.43444200	-1.24108900
H	3.12784600	3.43426400	1.24147300
H	1.10083900	4.65130500	0.48418900
H	-4.10087900	-2.90292700	0.48739900
H	-2.51036800	-4.06089700	1.99919600
H	-0.39037000	-2.91365800	2.65494000
H	0.17892300	-0.73297100	1.72248800
H	4.77523000	-0.95462800	0.78158200
H	4.40858000	1.41426400	1.39210200
H	-0.17901700	-0.73306700	-1.72258000
H	4.10055500	-2.90329800	-0.48743600
H	0.39006200	-2.91382900	-2.65493700
H	2.51009700	-4.06111000	-1.99921800
H	-1.10032500	4.65150800	-0.48401300
C	-1.15104700	3.56649800	-0.43842400
C	-2.27269800	2.89906600	-0.83599100

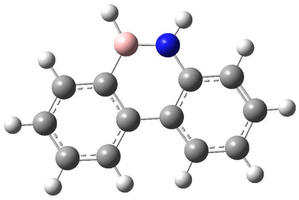

BN-phenanthrene

C	3.60038100	-0.27567600	0.00004300
C	2.86532400	0.90008400	0.00019300
C	1.45561100	0.89037700	0.00015100
C	0.76973800	-0.35759500	-0.00000600
C	1.53413500	-1.54459300	-0.00022200
C	2.92106900	-1.50249400	-0.00019200
C	-0.70307500	-0.37585100	0.00002500
C	-1.43403300	0.84231800	-0.00009500
C	-2.83982800	0.83178500	-0.00014600
H	-3.37181300	1.78126000	-0.00026000
C	-3.54090800	-0.36322200	-0.00002600
C	-2.83992000	-1.57600100	0.00017400
C	-1.45304400	-1.57097800	0.00020200
H	4.68681500	-0.25173900	0.00007800
H	3.37756700	1.85956000	0.00032000
H	1.04878600	-2.51468200	-0.00047600
H	3.48322700	-2.43316300	-0.00036300
H	-1.36015000	2.87516200	-0.00025100
H	-4.62730000	-0.35468700	-0.00006200
H	-3.37754400	-2.51974200	0.00032200
H	-0.93533600	-2.52375400	0.00041800
B	0.64779300	2.19533500	0.00008500
H	1.12552600	3.29152800	0.00014900
N	-0.75735000	2.06066600	-0.00013000

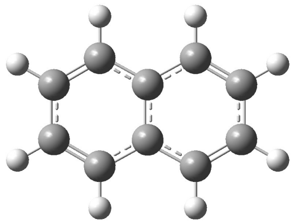

Naphthalene

C	2.43369500	-0.70845100	-0.00000100
C	1.24482600	-1.40245000	0.00000300
C	0.00001400	-0.71689300	0.00000000
C	-0.00001600	0.71688800	0.00000000
C	1.24480300	1.40245400	0.00000200
C	2.43367900	0.70847300	-0.00000200
H	-1.24211700	-2.49014700	0.00000600
H	3.37839400	-1.24557800	-0.00000400
H	1.24215600	-2.49013500	0.00000100
C	-1.24480700	-1.40246100	0.00000000
C	-1.24482900	1.40245600	0.00000000
H	1.24212300	2.49014000	0.00000000
H	3.37837300	1.24560700	-0.00000500
C	-2.43369100	0.70845400	0.00000000
C	-2.43367500	-0.70847000	-0.00000300
H	-1.24214600	2.49013900	0.00000400
H	-3.37840100	1.24556500	-0.00000100
H	-3.37837400	-1.24559700	-0.00000100

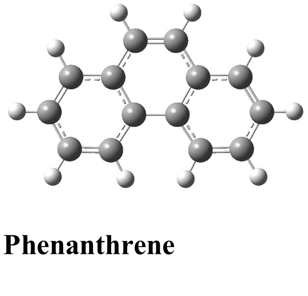

Phenanthrene

C	3.56188400	-0.29599100	0.00004400
C	2.83728700	0.87925200	0.00013200
C	1.42285200	0.86575600	0.00007300
C	0.72893800	-0.38114500	-0.00001900
C	1.50065400	-1.56703700	-0.00016000
C	2.88322200	-1.52911200	-0.00012900
C	0.67975200	2.09374300	0.00005400
C	-0.72893900	-0.38114300	0.00000700
C	-1.42285100	0.86575500	-0.00007900
C	-0.67975300	2.09374300	-0.00007000
C	-2.83728900	0.87925200	-0.00011400
H	-3.34753800	1.83974700	-0.00020000
C	-3.56188300	-0.29599000	-0.00002400
C	-2.88322000	-1.52911300	0.00013100
C	-1.50065500	-1.56703900	0.00014900
H	1.23275800	3.03003600	0.00012100
H	4.64814600	-0.27146200	0.00009100
H	3.34753700	1.83974600	0.00022900
H	1.00750300	-2.53318500	-0.00035000
H	3.44731400	-2.45793200	-0.00026100
H	-1.23275800	3.03003600	-0.00014300
H	-4.64814600	-0.27146700	-0.00004700
H	-3.44731900	-2.45792900	0.00025700
H	-1.00749500	-2.53318100	0.00032500

Electronic transitions: B3LYP(D3BJ)/ def2-TZVP

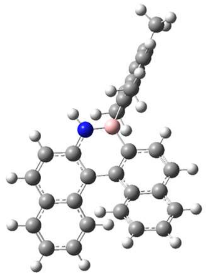

1-Mes

C	-0.60436200	-1.78678300	-0.70799800
C	-1.46652400	-0.79405800	-0.22312300
C	-0.92725200	0.54615800	0.00366800
C	0.44876000	0.68102900	0.25177600
C	-1.72128500	1.74896400	-0.09546000
C	-1.17449600	2.98037700	0.37405900
C	0.16962500	3.02522900	0.82671400
C	0.96543800	1.92675000	0.69605400
C	-1.10352800	-3.03769900	-1.14945900
C	-2.42057200	-3.34896400	-1.01269300
C	-3.29567700	-2.47022400	-0.32467100
C	-2.80823500	-1.20554600	0.12061000
C	-4.61623500	-2.86710100	-0.01247000
C	-5.42055600	-2.08891800	0.77968300
C	-4.91019900	-0.88942900	1.30966500
C	-3.64531200	-0.46278000	0.98847300
C	-2.97553300	1.79786500	-0.75049300
C	-3.69038600	2.96507900	-0.84675200
C	-3.19222000	4.15497100	-0.28232400
C	-1.95376500	4.15965000	0.30520500
H	1.30064800	-2.33588000	-1.12274900
H	0.56540200	3.96266600	1.19789000

H	2.02120500	1.98796300	0.92946400
H	-0.41113400	-3.74153800	-1.59536000
H	-2.80082200	-4.29809600	-1.36854900
H	-4.96961000	-3.81967000	-0.38831900
H	-6.42540200	-2.40751600	1.02394200
H	-5.51571800	-0.29994000	1.98612500
H	-3.27613600	0.45161200	1.42452800
H	-3.37068900	0.90068700	-1.20019100
H	-4.63975200	2.97205400	-1.36631200
H	-3.77216400	5.06677100	-0.34310600
H	-1.53412500	5.07851000	0.69606400
B	1.39625000	-0.45688500	-0.15366600
N	0.75914800	-1.57319000	-0.74244500
C	2.96112300	-0.39363500	-0.05023800
C	3.60976900	-0.88581200	1.09435000
C	3.73182300	0.18273200	-1.07318600
C	4.99688100	-0.80425500	1.19648400
C	5.11778900	0.24914800	-0.94407200
C	5.77044100	-0.24323400	0.18336500
H	5.48465700	-1.18206300	2.08869100
H	5.70131600	0.70128300	-1.73887200
C	3.06562500	0.73791600	-2.30700300
H	2.54261500	-0.04418100	-2.86274100
H	2.32038300	1.49360700	-2.04737400
H	3.79317600	1.19623000	-2.97643800
C	2.80639000	-1.49556600	2.21537300
H	2.03057800	-0.80955400	2.56468800
H	2.29862800	-2.40689000	1.88934900
H	3.44013300	-1.75027600	3.06457600

C	7.27051900	-0.19218500	0.29335300
H	7.58982800	-0.12105900	1.33385100
H	7.72356900	-1.09544300	-0.12552600
H	7.67985100	0.65942000	-0.25151800

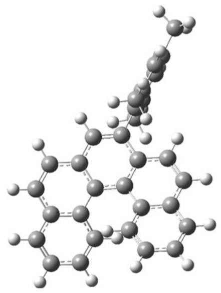

H

C	-0.62343600	-1.84964900	-0.65557100
C	-1.46251100	-0.80213100	-0.21220000
C	-0.87086500	0.50125300	-0.04922600
C	0.53436200	0.58587200	0.11033800
C	-1.60631500	1.75041100	-0.13021100
C	-0.98552700	2.95749800	0.29695900
C	0.38326600	2.94002300	0.68759700
C	1.12462100	1.81517100	0.53188600
C	-1.18543600	-3.09476700	-1.06719600
C	-2.51522000	-3.33531600	-0.95700200
C	-3.36095100	-2.39706700	-0.29728500
C	-2.82885400	-1.14846600	0.13323400
C	-4.69636100	-2.73416800	0.01365400
C	-5.47011000	-1.91404800	0.79704300
C	-4.91319600	-0.73490900	1.32021400
C	-3.62963900	-0.36597600	0.99521000
C	-2.87552100	1.85655000	-0.74260300
C	-3.53807700	3.05786300	-0.82492500
C	-2.96465000	4.22613000	-0.29478600

C	-1.70254100	4.17279700	0.24274100
H	1.40262800	-2.51238600	-0.96954900
H	0.83912900	3.85925000	1.03387600
H	2.18769500	1.82963600	0.72750600
H	-0.51944100	-3.84550500	-1.47407000
H	-2.94028800	-4.27232500	-1.29452500
H	-5.08879900	-3.67569200	-0.35072800
H	-6.48802500	-2.18923300	1.04036700
H	-5.49383500	-0.11567400	1.99162800
H	-3.22049500	0.53547100	1.42275900
H	-3.33027300	0.97800200	-1.17173000
H	-4.50423100	3.10353800	-1.31049900
H	-3.50008500	5.16525800	-0.34426900
H	-1.21960100	5.07305700	0.60262100
C	2.84587900	-0.45063000	-0.04994700
C	3.45316200	-0.79512300	1.16522900
C	3.62786300	-0.01367400	-1.12881000
C	4.83921300	-0.70764200	1.27914500
C	5.01042700	0.05931300	-0.97649800
C	5.63628800	-0.28688300	0.21845400
H	5.30579700	-0.97169400	2.22150400
H	5.61244600	0.40080300	-1.81114200
C	2.98882500	0.38475900	-2.43233900
H	2.45123100	-0.45113900	-2.88421700
H	2.26042600	1.18564900	-2.28758900
H	3.73950400	0.73100400	-3.14170900
C	2.62289800	-1.24122200	2.33898500
H	1.89365700	-0.47816400	2.62051700
H	2.05631000	-2.14462000	2.10435300

H	3.25217200	-1.44670700	3.20409300
C	7.13428300	-0.22968500	0.34938600
H	7.43717000	-0.11321700	1.39033100
H	7.59356600	-1.14897300	-0.02504100
H	7.55131600	0.59830000	-0.22547000
C	1.36523100	-0.54015200	-0.18630900
C	0.78323200	-1.68190500	-0.65333400

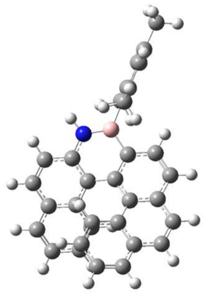

6-Mes

C	0.09495000	1.98733200	0.58980500
C	0.94803000	-0.53878300	-0.41136000
C	-0.82900600	1.10418300	0.01957600
C	-0.42629500	-0.28460300	-0.21320100
C	-0.29696000	3.28281200	1.01266300
C	-1.55821900	3.73701600	0.78314900
C	-2.47296100	2.96148600	0.02456600
C	-2.09123800	1.65812000	-0.40915000
H	2.00675600	2.30112000	1.18791900
H	0.43143400	3.90246600	1.52152900
H	-1.86023300	4.71792700	1.12766900
C	-3.73130100	3.48773500	-0.34551400
C	-2.96157300	0.99659200	-1.30674600

C	-4.16568100	1.54259900	-1.67692700
C	-4.57575200	2.78872900	-1.16970000
H	-4.00579400	4.47009100	0.01937800
H	-2.66968600	0.04675900	-1.72467100
H	-4.80185100	1.00849500	-2.37092100
H	-5.53509600	3.20213700	-1.45198900
C	0.43843400	-2.71254700	-1.33593000
C	1.35731200	-1.76636600	-0.97465200
C	-0.91355100	-2.56195800	-0.95346000
C	-1.33602500	-1.39618600	-0.26355300
C	-1.85349400	-3.60125800	-1.23830300
C	-3.12749700	-3.53899100	-0.78461100
C	-3.53296600	-2.48903900	0.09414100
C	-2.62365500	-1.44205800	0.40744800
H	0.73693700	-3.61676500	-1.85173700
H	2.41391600	-1.93268400	-1.14313900
H	-1.51805500	-4.44228400	-1.83229300
H	-3.84067700	-4.31765700	-1.02517900
C	-4.80362800	-2.52017800	0.70487900
C	-2.99605400	-0.54102000	1.42669000
C	-4.22884800	-0.60864600	2.03337900
C	-5.15801000	-1.58768900	1.65012300
H	-5.48878300	-3.31368000	0.43239600
H	-2.29619800	0.21364500	1.74750800

H	-4.47957500	0.09756000	2.81420500
H	-6.13333600	-1.62491200	2.11755400
C	3.52490000	0.21866300	0.15086500
C	4.12928400	-0.53612300	1.16902000
C	4.32697300	0.73173300	-0.88231600
C	5.50549100	-0.75716900	1.14522000
C	5.69898100	0.49183700	-0.88161400
C	6.30822800	-0.25364000	0.12529400
H	5.96093900	-1.33724200	1.94051900
H	6.30641200	0.89487800	-1.68496000
B	1.97464300	0.46229600	0.13917200
N	1.41848300	1.61779100	0.73363200
C	3.29624900	-1.11500000	2.28490000
H	2.74625000	-0.33626200	2.81830700
H	2.55367500	-1.81726100	1.89778600
H	3.91665200	-1.64518300	3.00714800
C	3.70461500	1.53867500	-1.99416500
H	2.89908200	0.98352300	-2.48167600
H	3.26530300	2.46517000	-1.61606500
H	4.44129300	1.80146100	-2.75300500
C	7.78705000	-0.53122500	0.09237700
H	8.17200300	-0.75089300	1.08865800
H	8.00800200	-1.39410100	-0.54247800
H	8.34132900	0.31747100	-0.31110800

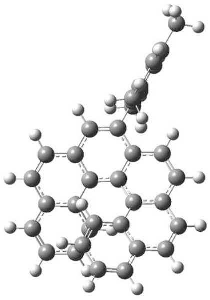

I

C	0.09867400	2.04944500	0.58348400
C	1.01828800	-0.46466100	-0.25043100
C	-0.82008100	1.12953300	0.03403800
C	-0.38087500	-0.23340100	-0.14649100
C	-0.34018600	3.34631200	0.98623400
C	-1.61493900	3.75390500	0.76821000
C	-2.51312700	2.93841400	0.01927600
C	-2.10181000	1.64359700	-0.40490600
H	2.15488200	2.43393800	1.10726900
H	0.37386800	3.99904900	1.47281700
H	-1.94980100	4.72932700	1.09829700
C	-3.78021800	3.42989300	-0.36116000
C	-2.94172600	0.95852300	-1.30920800
C	-4.15727900	1.47182900	-1.69394900
C	-4.60073300	2.70587800	-1.19116900
H	-4.08302400	4.40697700	-0.00487600
H	-2.61925900	0.01603300	-1.72162300
H	-4.77299400	0.92110800	-2.39311600

H	-5.56758300	3.09569300	-1.48165800
C	0.60974900	-2.65169900	-1.19638200
C	1.48471800	-1.68878100	-0.80039800
C	-0.76970400	-2.53993100	-0.88303900
C	-1.25194200	-1.38288400	-0.23075600
C	-1.65769200	-3.61448400	-1.18801700
C	-2.95150600	-3.59107900	-0.78395000
C	-3.41993700	-2.54689900	0.06611700
C	-2.55566500	-1.46619400	0.39851900
H	0.96077300	-3.55105100	-1.68655800
H	2.54922600	-1.82363800	-0.93034600
H	-1.26999900	-4.45164700	-1.75505000
H	-3.62880700	-4.39593100	-1.04091500
C	-4.71006000	-2.61196700	0.63447700
C	-2.99091300	-0.57017000	1.39855200
C	-4.23887900	-0.67349300	1.96534900
C	-5.12475300	-1.68422600	1.55817100
H	-5.36025400	-3.42903000	0.34656100
H	-2.32603500	0.20769800	1.73793400
H	-4.53655900	0.02794300	2.73385300
H	-6.11356100	-1.74829200	1.99323600
C	3.41261900	0.26091000	0.15924400
C	4.03522000	-0.39931400	1.22648200
C	4.16549600	0.66989500	-0.95162300

C	5.40859300	-0.63319000	1.16925700
C	5.53396100	0.41566100	-0.97315000
C	6.17524200	-0.23600900	0.07821100
H	5.88874500	-1.14022600	1.99849400
H	6.11302700	0.73393600	-1.83295300
C	3.24023500	-0.85625100	2.42034200
H	2.75229900	-0.01647100	2.91899900
H	2.44942000	-1.54992900	2.12668300
H	3.88136700	-1.35809400	3.14402500
C	3.50555900	1.36838700	-2.11053300
H	2.68393100	0.77344200	-2.51516900
H	3.07836300	2.32604300	-1.80603300
H	4.22103500	1.55275100	-2.91096800
C	7.65150200	-0.52228400	0.01916700
H	8.04684500	-0.76953100	1.00440600
H	7.85905700	-1.36738600	-0.64295000
H	8.20593300	0.33423900	-0.36806100
C	1.94753400	0.52766500	0.19748500
C	1.46948600	1.70424400	0.69383300

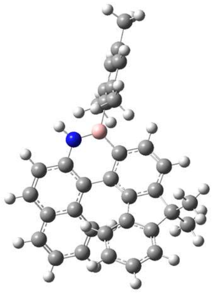

7-Mes

C	-0.37634700	2.26103100	-0.64639900
C	-0.96568600	-0.36205000	0.32551900
C	0.64262800	1.46666400	-0.10835600
C	0.38197200	0.04534500	0.11622800
C	-0.11875300	3.59038400	-1.06376800
C	1.09746000	4.16375700	-0.85049900
C	2.09491200	3.47566000	-0.11441300
C	1.84896500	2.13426500	0.30613200
H	-0.91253100	4.14225800	-1.55269700
H	1.29549700	5.17318700	-1.18833600
C	3.29636200	4.11985900	0.25756600
C	2.78128000	1.55367000	1.19826400
C	3.92287100	2.21651500	1.57574900
C	4.20536100	3.50169900	1.07832500
H	3.47163100	5.12830800	-0.09719300
H	2.58304200	0.57445800	1.60420800
H	4.60997800	1.74461500	2.26628300
H	5.11764600	4.00735000	1.36641000

C	-0.23542300	-2.59041800	0.97219700
C	-1.24325700	-1.66174700	0.77274700
C	1.05348700	-2.25229700	0.58372000
C	1.36627500	-0.97231400	0.10202800
C	3.26769800	-2.30142200	-0.18968900
C	2.73203000	-1.02953600	-0.45571200
H	-0.46144500	-3.58066000	1.34702400
H	-2.27629200	-1.94039800	0.94084800
C	4.53503200	-2.64762000	-0.62487900
C	3.46148900	-0.12690900	-1.22894600
C	4.72679400	-0.48557900	-1.68203100
C	5.27219100	-1.72835000	-1.36946900
H	4.94604000	-3.62611000	-0.40862900
H	3.05803900	0.84005700	-1.48557100
H	5.29408700	0.21414800	-2.28234900
H	6.26274300	-1.98736400	-1.72068200
C	-3.62157600	0.19136700	-0.12731500
C	-4.20426600	-0.58333200	-1.14428900
C	-4.42128100	0.61441700	0.94690600
C	-5.55725000	-0.90956900	-1.07865300
C	-5.77112300	0.27028600	0.98702400
C	-6.35985200	-0.48907000	-0.02065500
H	-5.99463600	-1.50908500	-1.86985400
H	-6.37568300	0.59932600	1.82530200

C	2.26300300	-3.17080100	0.54195800
C	1.97223300	-4.46553500	-0.23162400
H	2.87186000	-5.07868500	-0.30171400
H	1.20363700	-5.05113000	0.27480300
H	1.62711400	-4.24376400	-1.24197300
C	2.73906800	-3.50251300	1.96853600
H	1.97586500	-4.06972200	2.50392400
H	3.64968700	-4.10269200	1.93347000
H	2.94854900	-2.59092600	2.52931000
H	-2.33064000	2.40533600	-1.16698700
C	-3.82031400	1.43330100	2.06184200
H	-2.96327600	0.92351900	2.50918400
H	-3.45828400	2.39742500	1.69624600
H	-4.54847200	1.62516400	2.84965400
C	-3.37001600	-1.07559800	-2.30011400
H	-2.87334700	-0.24967300	-2.81428200
H	-2.58358200	-1.75267000	-1.95750200
H	-3.98013000	-1.60914500	-3.02865100
C	-7.82643000	-0.82477700	0.01784900
H	-8.40960100	-0.09298100	-0.54862900
H	-8.02141300	-1.80421900	-0.42128100
H	-8.20666600	-0.82463100	1.03992100
B	-2.09570200	0.55839500	-0.16095700
N	-1.66544600	1.77057600	-0.75002100

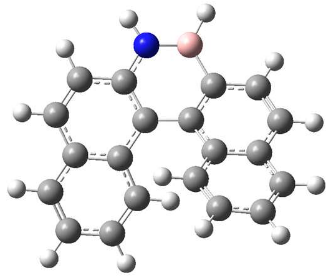

1-H

C	-1.39023900	1.97932100	0.35601600
C	-0.72053000	0.78536600	0.05107500
C	0.73490300	0.81331200	-0.08289000
C	1.35167900	2.02931300	-0.42803400
C	1.58245800	-0.32156700	0.20166100
C	2.95438300	-0.27486000	-0.18755700
C	3.49087200	0.91883600	-0.73695700
C	2.72798100	2.04689600	-0.78062500
C	-2.76136500	1.97880000	0.71545000
C	-3.49223400	0.83293100	0.67046200
C	-2.92265400	-0.35895300	0.15382000
C	-1.54367300	-0.37407800	-0.21022000
C	-3.72499300	-1.50069800	-0.07271400
C	-3.22026000	-2.60475800	-0.71007500
C	-1.89028800	-2.58583000	-1.16879600
C	-1.07950100	-1.50477700	-0.92520000
C	1.15569800	-1.43903000	0.95905200
C	1.99617800	-2.49064100	1.22376000

C	3.31738300	-2.48726900	0.73710500
C	3.78576400	-1.39378600	0.05614100
H	-1.28351100	3.99273400	0.54507300
H	4.53029200	0.93319800	-1.04098500
H	3.16674000	2.98798200	-1.08952100
H	-3.21467800	2.91292200	1.02433900
H	-4.53383700	0.83327800	0.96563400
H	-4.76121600	-1.47176300	0.24160700
H	-3.84551600	-3.46930600	-0.89031800
H	-1.50263600	-3.42712600	-1.72881600
H	-0.07005900	-1.51285500	-1.30407800
H	0.15126600	-1.45689600	1.35148300
H	1.64285900	-3.32420000	1.81671800
H	3.96742500	-3.33067900	0.93074300
H	4.81556400	-1.35300600	-0.27698800
B	0.57717400	3.32932800	-0.22468900
H	1.03410800	4.41837600	-0.38039200
N	-0.72743500	3.18993300	0.29001700

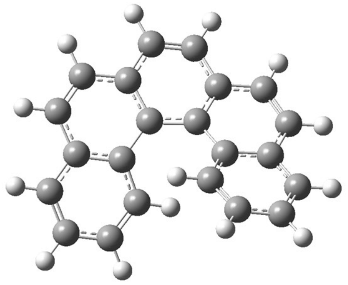

C

C	-1.36367800	2.01827400	0.32462800
C	-0.71910000	0.78752700	0.05250900
C	0.71919100	0.78739300	-0.05254600
C	1.36377900	2.01826300	-0.32462200
C	1.57003800	-0.36299300	0.19214500
C	2.94279500	-0.30833200	-0.18196200
C	3.49123000	0.90962900	-0.67789200
C	2.74497600	2.04159300	-0.68004000
C	-2.74473000	2.04161400	0.68043000
C	-3.49109400	0.90961500	0.67846700
C	-2.94279700	-0.30821200	0.18220700
C	-1.57008100	-0.36280900	-0.19221400
C	-3.77160400	-1.43204400	-0.02785700
C	-3.29841500	-2.55156200	-0.66624600
C	-1.97694600	-2.56603700	-1.14344000
C	-1.14015600	-1.50080200	-0.91109100
C	1.13996300	-1.50108700	0.91080700
C	1.97672100	-2.56636500	1.14312100
C	3.29825400	-2.55178700	0.66614800
C	3.77155300	-1.43215400	0.02802300
H	-1.17313900	4.16737300	0.38822800
H	4.53224100	0.92994200	-0.97515900
H	3.18396300	2.99226100	-0.95620400
H	-3.18366100	2.99218300	0.95697400

H	-4.53201900	0.92984800	0.97601200
H	-4.80423100	-1.37855600	0.29464700
H	-3.94638600	-3.40187300	-0.83410400
H	-1.61574600	-3.41686600	-1.70643500
H	-0.13538200	-1.53031800	-1.30137200
H	0.13518200	-1.53057300	1.30098900
H	1.61545800	-3.41732300	1.70587700
H	3.94625800	-3.40206600	0.83403600
H	4.80424600	-1.37864300	-0.29428000
H	1.17354100	4.16716500	-0.38824800
C	0.64821800	3.23823900	-0.20568800
C	-0.64817200	3.23827900	0.20495300

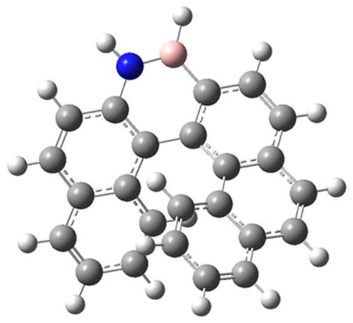

6-H

C	2.38034700	1.45434400	-0.58194000
C	0.00221700	2.88098100	0.07928000
C	1.27929500	0.78276900	-0.03593900
C	-0.01257000	1.46894000	0.02040000
C	3.59601500	0.77321400	-0.84424700

C	3.75821900	-0.52548200	-0.47545700
C	2.75147200	-1.18990100	0.27242900
C	1.52387500	-0.51780100	0.54260400
H	3.13547100	3.20046500	-1.28174500
H	4.39240000	1.31096800	-1.34422600
H	4.67963900	-1.04865300	-0.69743100
C	2.97643800	-2.48395800	0.79389600
C	0.62700100	-1.14660400	1.43723000
C	0.88317800	-2.39094300	1.95803400
C	2.05542400	-3.08670500	1.61213500
H	3.90756100	-2.98186000	0.55194100
H	-0.27317900	-0.63299200	1.73383300
H	0.17652600	-2.83619300	2.64648500
H	2.23979500	-4.07493400	2.01254700
C	-2.30049500	2.89873700	0.81390900
C	-1.16180400	3.57608400	0.47595200
C	-2.39469000	1.51111900	0.56151300
C	-1.28623000	0.80324400	0.02942400
C	-3.62324100	0.82441300	0.81394800
C	-3.78444000	-0.47511000	0.46986300
C	-2.77058000	-1.16117700	-0.26521500
C	-1.53747000	-0.50890600	-0.53995100
H	-3.16629000	3.41688700	1.20705100
H	-1.12740900	4.65675700	0.53977700

H	-4.42500200	1.37378100	1.29150500
H	-4.70963000	-0.99477100	0.68600600
C	-3.01027800	-2.45281300	-0.77805900
C	-0.65026300	-1.14585700	-1.43239900
C	-0.91840400	-2.39242500	-1.94839700
C	-2.09518200	-3.07075800	-1.59627100
H	-3.94686500	-2.93965500	-0.53510400
H	0.25460400	-0.64155600	-1.73105300
H	-0.21662200	-2.84902200	-2.63418900
H	-2.29237400	-4.05918100	-1.99025700
B	1.23682100	3.61906700	-0.43502600
N	2.30790300	2.80730600	-0.85850000
H	1.30491800	4.80104500	-0.56696200

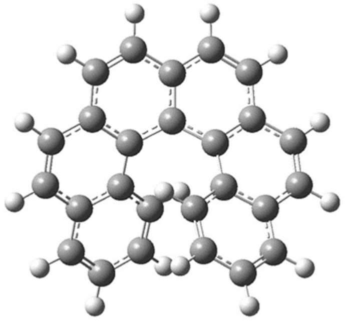

D

C	2.38799200	1.50201000	-0.55158000
C	-0.00033100	2.86458500	-0.00013900
C	1.28424600	0.78375900	-0.03745200
C	-0.00021000	1.44356700	0.00010500

C	3.61859800	0.83011500	-0.81639700
C	3.78737100	-0.47715700	-0.49857500
C	2.77830400	-1.17658000	0.22571200
C	1.54280300	-0.53152100	0.51381900
H	3.15593400	3.43372600	-1.13749700
H	4.41800600	1.39378000	-1.28115900
H	4.71430000	-0.98853300	-0.72607000
C	3.02097400	-2.47677700	0.71838800
C	0.66180900	-1.18280500	1.40393100
C	0.93536500	-2.43428800	1.90181600
C	2.11118600	-3.10706400	1.53145500
H	3.95713900	-2.95849500	0.46391900
H	-0.24099500	-0.68316200	1.71630100
H	0.24022400	-2.90032700	2.58798400
H	2.31136800	-4.10076800	1.91037100
C	-2.29133200	2.90319200	0.76004600
C	-1.16507900	3.57311000	0.39710900
C	-2.38829200	1.50164800	0.55167700
C	-1.28439300	0.78353400	0.03752000
C	-3.61885000	0.82940700	0.81645100
C	-3.78739100	-0.47777300	0.49844800
C	-2.77812100	-1.17707400	-0.22583300
C	-1.54272200	-0.53183700	-0.51383200
H	-3.15629500	3.43329500	1.13815400

H	-1.12246100	4.65416500	0.43984300
H	-4.41832100	1.39295700	1.28124400
H	-4.71426100	-0.98934400	0.72574900
C	-3.02054500	-2.47721500	-0.71855200
C	-0.66145700	-1.18292600	-1.40370700
C	-0.93474500	-2.43452000	-1.90166200
C	-2.11050100	-3.10738100	-1.53154600
H	-3.95664800	-2.95916600	-0.46429600
H	0.24136000	-0.68319000	-1.71586300
H	-0.23934600	-2.90045400	-2.58763700
H	-2.31058900	-4.10111400	-1.91043900
C	1.16435700	3.57330900	-0.39739800
C	2.29080600	2.90372600	-0.75994400
H	1.12153600	4.65437500	-0.43976600

NICS calculation-optimisation: b3lyp/6-31G(d)

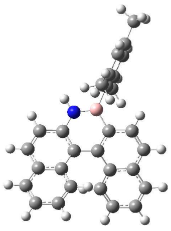

1-Mes

C	0.58116800	-1.78044900	-0.68676000
C	1.47022800	-0.79558000	-0.21367700
C	0.95232100	0.56492900	0.00464900

C	-0.42992200	0.72597400	0.25450600
C	1.76848500	1.76248700	-0.10553700
C	1.23663400	3.01256400	0.35371800
C	-0.10958100	3.08144000	0.81060900
C	-0.92575300	1.98995500	0.69087800
C	1.05001200	-3.04875500	-1.12770400
C	2.36580600	-3.39150100	-0.99881100
C	3.26797300	-2.52717100	-0.31856900
C	2.81214300	-1.24145700	0.12345800
C	4.58408300	-2.95913600	-0.01104200
C	5.41812900	-2.19644200	0.77452900
C	4.94254300	-0.97521100	1.29885700
C	3.68193000	-0.51360700	0.98210700
C	3.03014200	1.79354300	-0.76202600
C	3.76153200	2.95721100	-0.86989700
C	3.27512400	4.16322800	-0.31884100
C	2.03213700	4.18611600	0.27158000
H	-1.34490700	-2.30338300	-1.07267100
H	-0.49266600	4.03194200	1.17465500
H	-1.98383800	2.07031000	0.92631600
H	0.33635900	-3.74243100	-1.56761700
H	2.72245600	-4.35522200	-1.35319800
H	4.90943600	-3.92731900	-0.38505100
H	6.41899900	-2.54326500	1.01608300

H	5.57033100	-0.39418600	1.96924000
H	3.33923500	0.41593200	1.41777200
H	3.41589000	0.88570200	-1.20819500
H	4.71512900	2.94613200	-1.39081500
H	3.86633300	5.07227200	-0.39049800
H	1.61992100	5.11680400	0.65451600
N	-0.78462600	-1.53969300	-0.71070500
B	-1.40741700	-0.40281700	-0.12885900
C	-2.98321200	-0.34380500	-0.02813900
C	-3.63764000	-0.78092200	1.14574500
C	-3.76712000	0.14512300	-1.09771100
C	-5.03311100	-0.73188000	1.22867200
C	-5.16088000	0.17982600	-0.98313600
C	-5.81533800	-0.25863500	0.17154000
H	-5.52137700	-1.06915100	2.14172100
H	-5.75040800	0.56317100	-1.81460200
C	-3.11286800	0.64934500	-2.36842500
H	-2.44258400	1.49421600	-2.16650800
H	-2.50446900	-0.12602200	-2.85016800
H	-3.86088900	0.98333900	-3.09463600
C	-2.83844900	-1.30309400	2.32267100
H	-2.27453400	-2.20767900	2.06104400
H	-2.10528600	-0.56537700	2.67197700
H	-3.48936400	-1.55230900	3.16690600

C	-7.32363400	-0.24261800	0.26507600
H	-7.75759200	-1.17809400	-0.11379900
H	-7.66006100	-0.12682600	1.30111700
H	-7.75392500	0.57449600	-0.32426500

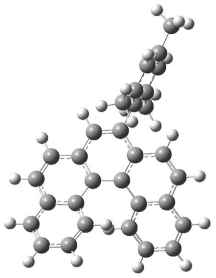

H

C	0.61142900	-1.83946300	-0.65934800
C	1.47127700	-0.80009700	-0.21426900
C	0.89063100	0.51785600	-0.04575000
C	-0.52157700	0.61761100	0.12026600
C	1.63986400	1.76917100	-0.12504000
C	1.02764600	2.98686500	0.30777200
C	-0.34294800	2.97893400	0.70801800
C	-1.09863600	1.85678100	0.55171000
C	1.15206300	-3.09714800	-1.07841700
C	2.48213200	-3.36459200	-0.97024700
C	3.34904600	-2.44134900	-0.30659300
C	2.84096700	-1.17514700	0.12596800
C	4.68100800	-2.81058500	0.00457400
C	5.47937100	-2.00634100	0.78962800

C	4.95039600	-0.80823500	1.30994700
C	3.66911200	-0.40734700	0.98586100
C	2.91465600	1.87171800	-0.74162500
C	3.58625500	3.07544900	-0.82574200
C	3.01843500	4.25140500	-0.29511300
C	1.75349000	4.20254300	0.25024800
H	-0.79193100	3.90465600	1.05980400
H	-2.16435500	1.88027700	0.75420200
H	0.46857400	-3.83671000	-1.48843500
H	2.88998000	-4.31323500	-1.31055200
H	5.05117200	-3.76557600	-0.36133700
H	6.49430400	-2.30820100	1.03347000
H	5.54880100	-0.19727200	1.98041000
H	3.28141900	0.50720300	1.41583500
H	3.36489200	0.98856800	-1.17644500
H	4.55549100	3.11451000	-1.31555100
H	3.55926800	5.19237800	-0.34789100
H	1.27378900	5.10839500	0.61378800
C	-2.86472400	-0.41463600	-0.04088000
C	-3.48106300	-0.77644800	1.17522700
C	-3.65513800	0.01054300	-1.12972600
C	-4.87467400	-0.71129400	1.27865300
C	-5.04524800	0.05873400	-0.98317100
C	-5.67664700	-0.30158300	0.21049000

H	-5.34450400	-0.98901600	2.22045700
H	-5.65006100	0.38970600	-1.82557600
C	-3.02409600	0.42022200	-2.44222600
H	-2.32732500	1.25657300	-2.31049100
H	-2.44961900	-0.39898600	-2.88972200
H	-3.78821200	0.72899700	-3.16227800
C	-2.65873800	-1.22589700	2.36276500
H	-2.07758100	-2.12691800	2.13415900
H	-1.93793300	-0.45820500	2.66879900
H	-3.30064600	-1.44648700	3.22122000
C	-7.18259000	-0.27142900	0.33307500
H	-7.62974700	-1.21319200	-0.01293400
H	-7.49769100	-0.12559400	1.37180700
H	-7.61844700	0.53302100	-0.26945200
C	-1.37362300	-0.50296300	-0.17696300
C	-0.79812500	-1.65385000	-0.65177100
H	-1.42934400	-2.48146500	-0.96721400

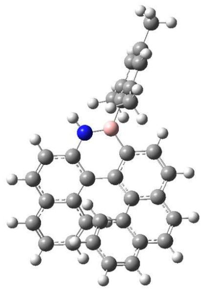

6-Mes

C	0.11742000	1.95926800	0.62894700

C	0.91275700	-0.56941600	-0.44132600
C	-0.83714500	1.10877000	0.04081300
C	-0.46487100	-0.29384100	-0.22651000
C	-0.22832400	3.26745000	1.06877500
C	-1.47001400	3.78057400	0.82488300
C	-2.40766300	3.05002200	0.04241100
C	-2.08104200	1.72337200	-0.39161000
H	2.04670500	2.21262700	1.22517500
H	0.52174100	3.85420300	1.59519400
H	-1.73429000	4.77573400	1.17345800
C	-3.63043700	3.64794300	-0.35620100
C	-2.97761400	1.10780100	-1.30544100
C	-4.14463700	1.72581700	-1.70468200
C	-4.49520100	2.99887100	-1.20820300
H	-3.85804700	4.64773400	0.00682900
H	-2.72940300	0.13993500	-1.72059900
H	-4.79648000	1.22513900	-2.41560900
H	-5.42425600	3.47018500	-1.51664800
C	0.35593000	-2.71472100	-1.42955400
C	1.29700300	-1.79156100	-1.04758100
C	-0.99261300	-2.55739100	-1.02267600
C	-1.39847900	-1.39754900	-0.29664400
C	-1.94158400	-3.59413700	-1.31127000
C	-3.20622200	-3.55153600	-0.81562200

C	-3.59228700	-2.52082900	0.10103300
C	-2.68112800	-1.46124400	0.40307300
H	0.63720500	-3.61316500	-1.97370000
H	2.35315900	-1.97165600	-1.22980600
H	-1.61728800	-4.42288000	-1.93587700
H	-3.92291200	-4.33503600	-1.04998700
C	-4.84198000	-2.58780900	0.76128900
C	-3.04039300	-0.57883100	1.45142300
C	-4.25352200	-0.68341100	2.10603100
C	-5.17908400	-1.67949300	1.74367800
H	-5.52382300	-3.39335500	0.49816200
H	-2.34076900	0.18520700	1.76420200
H	-4.48815900	0.00827600	2.91059100
H	-6.13729100	-1.74812600	2.25147400
C	3.53394200	0.15222600	0.13718700
C	4.16081700	-0.54049700	1.19683500
C	4.33423300	0.63478600	-0.92377900
C	5.54729100	-0.73022800	1.18356300
C	5.71682900	0.42669000	-0.90758400
C	6.34451200	-0.25679400	0.13828200
H	6.01612000	-1.26292800	2.00941500
H	6.31887400	0.80747700	-1.73134600
B	1.97276700	0.39535800	0.12948100
N	1.43662000	1.54776300	0.76290500

C	3.34632600	-1.08678000	2.35206900
H	2.79457100	-0.29462600	2.87324800
H	2.60094800	-1.81480400	2.00835600
H	3.98496700	-1.58614900	3.08778300
C	3.70663400	1.38574000	-2.08102800
H	2.90803500	0.80196400	-2.55529000
H	3.25387200	2.33093600	-1.75471800
H	4.44852500	1.62371300	-2.85012200
C	7.83625100	-0.49856900	0.12256200
H	8.22363900	-0.68188500	1.13039600
H	8.09230900	-1.37422600	-0.48925400
H	8.37765500	0.35642700	-0.29792600

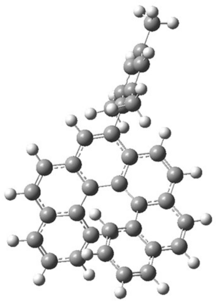

I

C	1.48039600	1.65545500	0.73378200
C	1.94601300	0.48332400	0.19689000
C	0.11143900	2.03019300	0.63121600
C	0.99189700	-0.48330300	-0.28097700
C	-0.83198200	1.13893700	0.05699600
C	-0.41263800	-0.23593700	-0.16081100

C	-0.29242300	3.33643500	1.05700800
C	-1.55301800	3.79416700	0.82555200
C	-2.46969800	3.01967400	0.04760000
C	-2.10231800	1.70513700	-0.38290300
H	2.18020500	2.36608100	1.16741300
H	0.43999200	3.95829600	1.56599300
H	-1.85868300	4.78098500	1.16451100
C	-3.70802400	3.57437400	-0.35765900
C	-2.96487200	1.06160900	-1.30583200
C	-4.15112800	1.63884600	-1.71508500
C	-4.54606000	2.89567500	-1.21698700
H	-3.97218600	4.56643100	0.00153800
H	-2.67816900	0.10408700	-1.72067000
H	-4.77973500	1.11806400	-2.43227400
H	-5.48835800	3.33713300	-1.52989200
C	0.55014200	-2.64111900	-1.30087900
C	1.44209000	-1.69825800	-0.87757000
C	-0.82829100	-2.53018400	-0.96469200
C	-1.30094200	-1.38345100	-0.27085800
C	-1.71950400	-3.60559700	-1.27969100
C	-3.00557800	-3.60854700	-0.83528500
C	-3.46131500	-2.58817700	0.05701500
C	-2.60048700	-1.49274100	0.38468800
H	0.89029900	-3.53211300	-1.82279800

H	2.50820000	-1.84078300	-1.01938900
H	-1.33746000	-4.42681400	-1.88125800
H	-3.68251000	-4.42015800	-1.09069200
C	-4.73209300	-2.69581600	0.67257600
C	-3.02895500	-0.62048900	1.41692900
C	-4.25869100	-0.76712400	2.02877800
C	-5.13600100	-1.79673000	1.63669500
H	-5.37482200	-3.52628600	0.38940700
H	-2.36813000	0.16787800	1.75293100
H	-4.54532000	-0.08379300	2.82352600
H	-6.10877400	-1.89724600	2.11033300
C	3.42052900	0.21138200	0.15415000
C	4.05231000	-0.44836400	1.22823500
C	4.18338200	0.64134100	-0.95310700
C	5.43471600	-0.66145400	1.17748700
C	5.56125800	0.40536700	-0.96356700
C	6.20801100	-0.24563700	0.09136300
H	5.91826400	-1.16691400	2.01135300
H	6.14444300	0.74167400	-1.81913000
C	3.26360700	-0.92235600	2.42897900
H	2.76852400	-0.09041700	2.94327000
H	2.47470200	-1.62675800	2.14018300
H	3.91569500	-1.42451900	3.15027900
C	3.53254800	1.35067500	-2.12014700

H	2.73295300	0.74621700	-2.56454300
H	3.07427400	2.29820100	-1.81354100
H	4.26594400	1.56822400	-2.90273900
C	7.69499000	-0.51117300	0.04307200
H	7.92466800	-1.38174800	-0.58589200
H	8.24166700	0.34108900	-0.37627000
H	8.09846300	-0.71361300	1.04060100
